# Prokaryotic and Eukaryotic Horizontal Transfer of *Sailor* (DD82E), a New Superfamily of *IS630-Tc1-Mariner* DNA Transposons

**DOI:** 10.3390/biology10101005

**Published:** 2021-10-07

**Authors:** Shasha Shi, Mikhail Puzakov, Zhongxia Guan, Kuilin Xiang, Mohamed Diaby, Yali Wang, Saisai Wang, Chengyi Song, Bo Gao

**Affiliations:** 1 College of Animal Science & Technology, Yangzhou University, Yangzhou 225009, China; MX120190668@yzu.edu.cn (S.S.); MX120190652@yzu.edu.cn (Z.G.); xkuilin@outlook.com (K.X.); DH18035@yzu.edu.cn (M.D.); DX120180099@yzu.edu.cn (Y.W.); DX120170084@yzu.edu.cn (S.W.); cysong@yzu.edu.cn (C.S.); 2 A.O. Kovalevsky Institute of Biology of the Southern Seas of RAS, Nakhimov av., 2, 299011 Sevastopol, Russia; puzakov@ngs.ru

**Keywords:** *Tc1/mariner* transposons, DD82E/*Sailor*, horizontal transfer

## Abstract

**Simple Summary:**

Transposable elements, including DNA transposons, play a significant role in genetic material exchanges between prokaryotes and eukaryotes. Comparative profiling of the evolution pattern of DNA transposons between prokaryotes and eukaryotes may identify potential genetic material exchanges between them and provide insights into the evolutionary history of prokaryotic and eukaryotic genomes. The members of the *IS630-Tc1-mariner* (*ITm*) group may represent the most diverse and widely distributed DNA transposons in nature, and the discovery of new members of this group is highly expected based on the increasing availability of genome sequencing data. We discovered a new superfamily (termed *Sailor*) belonging to the *ITm* hyperfamily, which differed from the known superfamilies of *Tc1/mariner*, DDxD/*pogo* and DD34E/*Gambol*, regarding phylogenetic position and catalytic domain. Our data revealed that *Sailor* was distributed in both prokaryotes and eukaryotes and suggested that horizontal transfer (HT) events of *Sailor* may occur from prokaryotic to eukaryotic genomes. Finally, internal transmissions of *Sailor* in prokaryotes and eukaryotes were also detected.

**Abstract:**

Here, a new superfamily of *IS630-Tc1-mariner* (*ITm*) DNA transposons, termed Sailor, is identified, that is characterized by a DD82E catalytic domain and is distinct from all previously known superfamilies of the *ITm* group. Phylogenetic analyses revealed that *Sailor* forms a monophyletic clade with a more intimate link to the clades of *Tc1/mariner* and *DD34E/Gambol*. *Sailor* was detected in both prokaryotes and eukaryotes and invaded a total of 256 species across six kingdoms. *Sailor* is present in nine species of bacteria, two species of plantae, four species of protozoa, 23 species of Chromista, 12 species of Fungi and 206 species of animals. Moreover, *Sailor* is extensively distributed in invertebrates (a total of 206 species from six phyla) but is absent in vertebrates. *Sailor* transposons are 1.38–6.98 kb in total length and encoded transposases of ~676 aa flanked by TIRs with lengths between 18, 1362 and 4 bp (TATA) target-site duplications. Furthermore, our analysis provided strong evidence of *Sailor* transmissions from prokaryotes to eukaryotes and internal transmissions in both. These data update the classification of the *ITm* group and will contribute to the understanding of the evolution of *ITm* transposons and that of their hosts.

## 1. Introduction

Transposable elements (TEs) are DNA sequences capable of integration and movement within the host genome. The proportion of TEs in the genomes of various organisms varies widely (from 3% to 90%) [1,2,3]. As a result of displacements, TEs can change the primary structure of DNA, interfere with the work of genes and change their function, influence the processes of regulation of transcription, and cause chromosomal rearrangements [4,5,6]. TE-mediated mutations are usually classified as insertional mutations [7,8]. Moreover, the epigenetic topology of the eukaryotic genome can change as a result of transpositions [9]. In addition, TE nucleotide sequences can be the source of new genes [10,11]. A large number of stress factors, both intracellular and external, have been shown to affect the induction of TE movements. These include high and low temperature, pH, ultraviolet radiation, magnetic fields, gamma radiation, various chemical compounds, outbreeding, inbreeding, infections, and starvation, among others [4]. A natural consequence of increased stress-induced mutagenesis is a growth in the spectrum of genetic diversity. In turn, this increases the adaptive potential of the population, which can also contribute to speciation [12,13]. Currently, TEs are divided into two classes: retrotransposons (class I) and DNA transposons (class II). Class I includes TEs that encode reverse transcriptase and are transported by an RNA intermediate, as well as their non-autonomous derivatives, whereas Class II combines TEs that use DNA to move their copies to a new position in the genome and can be divided into two subclasses. The first subclass consists of two groups of elements: TIR and Crypton. TIRs are characterized by terminal inverted repeats (TIRs) and the enzyme transposase, through which transposition occurs via a cut-and-paste mechanism. TIR transposons include the *hAT*, *Merlin*, *Mutator*, *PiggyBac*, *PIF-Harbinger*, *IS630-Tc1-mariner* and *Transib* TE groups, among others. Crypton elements use tyrosine recombinase in the transposition mechanism. *Helitron* and *Maverick* are the two main representative elements of the second subclass. These elements are moved using a copy-and-paste mechanism [5,11,14,15]. One of the most widespread groups of cut-and-paste DNA transposons is the *IS630-Tc1-mariner* (*ITm*). Members of this group are present in almost all branches of the tree of life [16]. Autonomous *ITm* transposons usually carry one open reading frame (ORF) encoding the transposase enzyme, flanked by TIRs. *ITm* transposases are characterized by the presence of a paired domain (two HTH motifs) and a DDE/D domain. In addition, structures such as the GRPR-type motif and a nuclear localization signal (NLS) are present [17,18]. The paired domain is in the first half of the amino acid sequence and provides specific binding to TIRs. The second half contains the DDE/D domain, which possesses the catalytic activity required for the excision and insertion of the TE. The GRPR-like motif is located between the two HTH motifs of the paired domain and is supposed to mediate the binding of the paired domain to the target site duplication (TSD), the TA dinucleotide [19], which has been identified in most *Tc1/mariner* superfamilies, such as DD35E/*TR* [20], DD36E/*IC* [21], DD38E/*IT* [22], DD37D/*maT* [23], DD39D/*GT* [23] and DD41D/*VS* [24]. The classification of *ITm* transposons is reliably associated with the length of the peptide chain between the second “D” and the third “E/D” amino acid residues (aa) of the catalytic domain, the so-called DDE/D signature. Nine families are classically considered, and most of their phylogenetic relationships have been updated and are well defined: DD34E/*Tc1* [25], DD34D/*mariner* [26], DD37D/*maT* [23], DD39D/*GT* [23], DD41D/*VS* [24], DD35E/*TR* [20], DD36E/*IC* [21], DD37E/*TRT* [27] and DD38E/*IT* [22]. However, until recently, studies have shown that many groups of *ITm* transposons carry the same DDE/D signatures but have different phylogenetic origins [28,29,30]. In particular, the DD34E/*Gambol* [31] and DDxD/*pogo* [30] transposons, which were designated as the *Tc1/mariner* superfamily, have been shown to form two separate superfamilies with good bootstrap support [30]. Consequently, the rank of the *ITm* group is automatically promoted to hyperfamily, because it includes more than one superfamily. This situation underscores the need to study new groups of *ITm* transposons and their prevalence among living organisms. Here, we identified a new superfamily of *ITm*, termed *Sailor*, that forms an independent superfamily with a distinct DDE domain (DD78-111E) and different phylogenetic positions compared with previous superfamilies (DDxD/*pogo*, DD34E/*Gambol*, *Tc1/mariner*, *Zator* and *TP36*) [25,26,30,31,32]. We also systematically characterized their evolution landscapes (taxonomic distribution and evolution patterns) and structural organization. The current study updated the classification of *ITm* DNA transposons and expanded our knowledge about the diversity of DNA transposons and their contributions to genome evolution in prokaryotes and eukaryotes.

## 2. Materials and Methods

### 2.1. Transposon Mining and Annotation

New transposon sequences were identified by systematically screening the new transposase families as part of the development of genetic manipulation tools using the TE database of RepBase. All *Tc1/mariner* DNA transposon sequences deposited in RepBase were downloaded (Version: 20181026). Transposase-coding sequences were predicted with GENSCAN (http://hollywood.mit.edu/GENSCAN.html, accessed on 15 October 2020). Subsequently, these transposases (>300 aa) were used for phylogenetic and multiple-alignment analyses, to define putative new families. The distinct DD82E domain of transposase was firstly identified in four species including *Lobosporangium transversal* (607 aa), *Locusta migratoria* (461 aa), *Crassostrea gigas* (376 aa), and *Acyrthosiphon pisum* (434 aa), which were deposited in RepBase. Then, these transposase sequences were used as a query to search the genomes deposited in the National Center for Biotechnology Information (NCBI) genome project database (https://blast.ncbi.nlm.nih.gov/Blast, accessed on 25 October 2020) using TBLASTN with an E-value of 1e–100. Subsequently, the best hits were extracted with 2-kb flanking sequences, since most *ITm* families contain TIRs (including untranslated region) less than 2 kb as previous studies reported, such as DD35E/*TR* [20], DD36E/*IC* [21], DD38E/*IT* [22], DD37D/*maT* [23], DD39D/*GT* [23]. The presence of TIRs and TSDs was manually screened in the resulting sequences, to identify potential DNA transposons. Putative sequence contamination was verified further by checking the flanking sequences of transposons, and the transposons located on very short contigs that failed to map to the species genome or lacked flanking sequences were designated as sequence contamination and were excluded from the analysis. The WebLogo server (http://weblogo.berkeley.edu/logo.cgi/, accessed on 10 April 2021) was used to create the logo representation of the TSD sequences [33]. Putative nuclear localization signal (NLS) motifs were predicted using PSORT II, as provided on the PSORT server (http://psort.nibb.ac.jp/, accessed on 25 April 2021), and the secondary structures and motifs of the transposases were predicted using the PSIPRED program (http://bioinf.cs.ucl.ac.uk/psipred/, accessed on 25 April 2021) [34], Pfam (http://pfam.xfam.org/, accessed on 26 April 2021) and HMMER web server (https://www.ebi.ac.uk/Tools/hmmer//search/phmmer, accessed on 28 April 2021). The consensus sequences of each transposon in each genome were reconstructed via the multiple alignment of copies in each genome using the online emboss explorer (http://www.bioinformatics.nl/emboss-explorer/, accessed on 5 April 2021). In cases where there were a few copies of the transposon in the genome, the longest copy in the genome was designated as the representative element for further analysis. The copy number of each transposon in each species was estimated based on the Blast result of the various genome sequences (>40% coverage in length and >80% identity) using consensus or representative sequences of the identified transposon as queries. All identified transposon sequences reported in this paper are deposited as Supplementary Material in FASTA format (Data S1).

### 2.2. Phylogenetic Analysis

The maximum likelihood method was used to infer the phylogenetic tree using the IQ-TREE program with an ultrafast bootstrap value of 1000, based on the alignment of the conserved DDE region of transposases [35]. The best-suited aa substitution model for these data was the LG+I+G4 model according to BIC, which was selected by ModelFinder embedded in the IQ-TREE program [35]. The MAFFT program was used to perform multiple alignments of DDE domains [36]. Reference transposase sequences were obtained from GenBank and the references of DD35E/*TR* [20], DD36E/*IC* [21], DD37E/*TRT* [27], DD38E/*IT* [22], *Zator* and *TP36* [32]. Bacterial transposases from insertion sequence 256 (*IS256*) were chosen as the outgroup.

### 2.3. HT Detection

The pairwise distances between the host genes and the transposons were used to detect horizontal transfer events of *Sailor* transposons. In total, 29 ribosomal proteins (cytoplasmic and organelle) that were identified as universally conserved proteins [37] were evaluated for their conservation and length (Figures S1 and S2), as well as taxonomic distribution of the single genomic copy across domains (Table S1), to select the fit host genes for the HT hypothesis test. The accession numbers and host species are listed in Table S2. The taxonomic distribution of a single genomic copy of host genes across the domains of Bacteria, Archaea and Eucarya (Plantae, Chromista, Protozoa, Eumycota and animals) was evaluated using the online OrthoDB database (https://www.orthodb.org/, accessed on 5 May 2021). Homologous genes between prokaryote and eukaryote species were identified by an online TBlastN or BLASTP search using prokaryote proteins as queries. In addition, in order to obtain accurate results, only species containing intact transposons (full length elements coding transposase >300 aa flanked with TIRs) were selected for HT analysis. Putative HT events between organisms, as detected based on pairwise distances between the various organisms, were determined for *Sailor* and the selected host gene sequences using the MEGA program depending on two multiple alignments [38]. The multiple sequence alignments of the host-gene-coding sequences and transposase-coding sequences were created using the MAFFT program (version 7.481) [36]. Subsequently, comparison distances between the host genes and transposons were calculated using MEGA software (v. 7.0.26) based on two aligned files (pairwise deletion and maximum composite likelihood). Species for which we could not retrieve highly conserved host genes in the NCBI database were excluded from this analysis. The accession numbers and host species are listed in Table S3. The genetic distances between host genes and transposons in each species, as determined using a pairwise comparison, are listed in Tables S4 and S5.

## 3. Results

### 3.1. Discovery of a New Superfamily of ITm DNA Transposons, DD82E/Sailor

**Figure 1:**
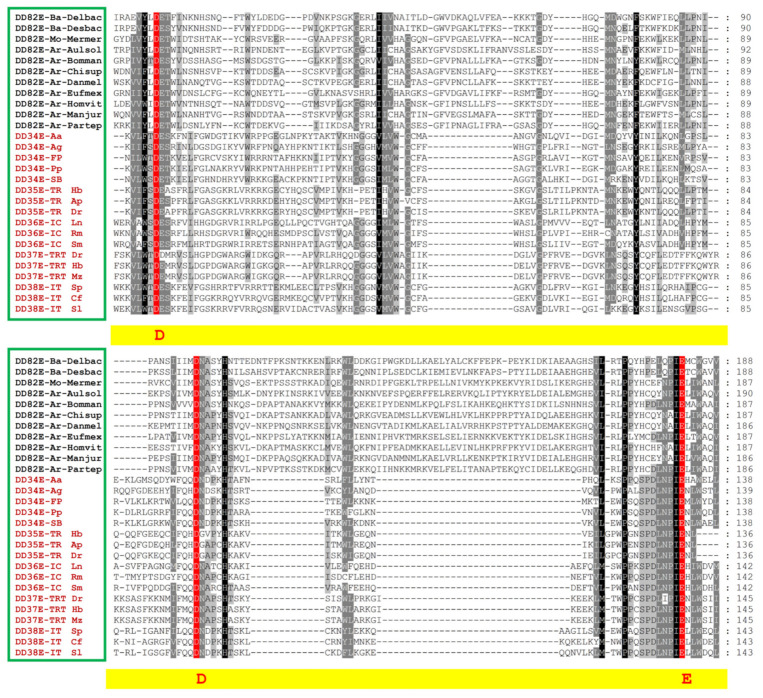
Schematic diagram of the catalytic domains of transposases, which include DD82E/*Sailor*, DD34E/*Gambol*, DD34E/*Tc1*, DD35E/*TR*, DD36E/*IC*, DD37E/*TRT* and DD38E/*IT*.

**Figure 2:**
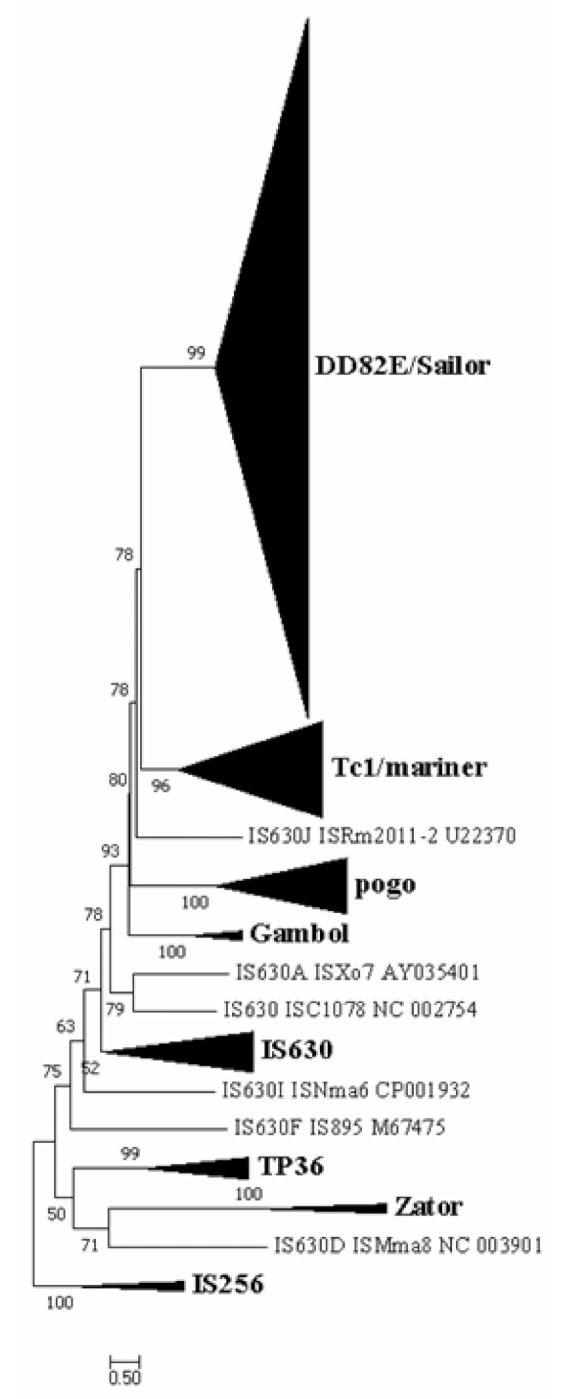
Phylogenetic position of the *Sailor* superfamily. This phylogenetic tree was generated based on DDE domains using the maximum likelihood method in the IQ-TREE program with an ultrafast bootstrap approach (1000 replicates). The reference families and elements included DD34E/*Tc1*, DD34E/*Gambol*, DD35E/*TR*, DD36E/*IC*, DD37E/*TRT*, DD38E/*IT*, DD34D/*mariner*, DD37D/*maT*, DD39D/*GT*, DD41D/*VS*, DDxD/*pogo*, *IS630* transposases, *TP36*, and *Zator*. *IS256* was used as an outgroup. The uncollapsed tree is presented in Figure S3.

We identified a cluster of transposases with a new type of catalytic domain that was distinct from the known transposase superfamilies of the *ITm* group, most of which were represented by DD82E, but with variations between DD78E and DD111E (Figure 1, Table S6 and Data S2). The phylogenetic tree (Figure 2 and Figure S3), which was obtained via maximum likelihood method using the IQ-TREE program [35], demonstrated that DD82E/*Sailor* elements form a well-defined monophyletic clade with a high degree of support (99%). The *Tc1/mariner* transposons were the closest known group to DD82E/*Sailor* on the phylogenetic tree, which were grouped into a separate clade with 99% bootstrap support. Furthermore, the known superfamilies, including DD34E/*Gambol* [31], DDxD/*pogo* [30], *Zator* and *TP36* [32], were identified, and formed separate clades with 100% bootstrap support. The listed groups formed branches separately with very high support (≥99%) for each clade and were “mixed” with *IS630* transposons (Figure 2 and Figure S3), which is possibly related to the independent emergence of these groups from *IS630* during evolution. Overall, the phylogenetic analysis showed that the discovered transposons of *Sailor* with a new type of catalytic domain (mainly represented by DD82E, with variation from DD78E to DD111E) are a new superfamily of the *ITm* infraclass, close to *Tc1/mariner*.

### 3.2. Distribution of DD82E/Sailor in Both Prokaryotes and Eukaryotes

**Figure 3:**
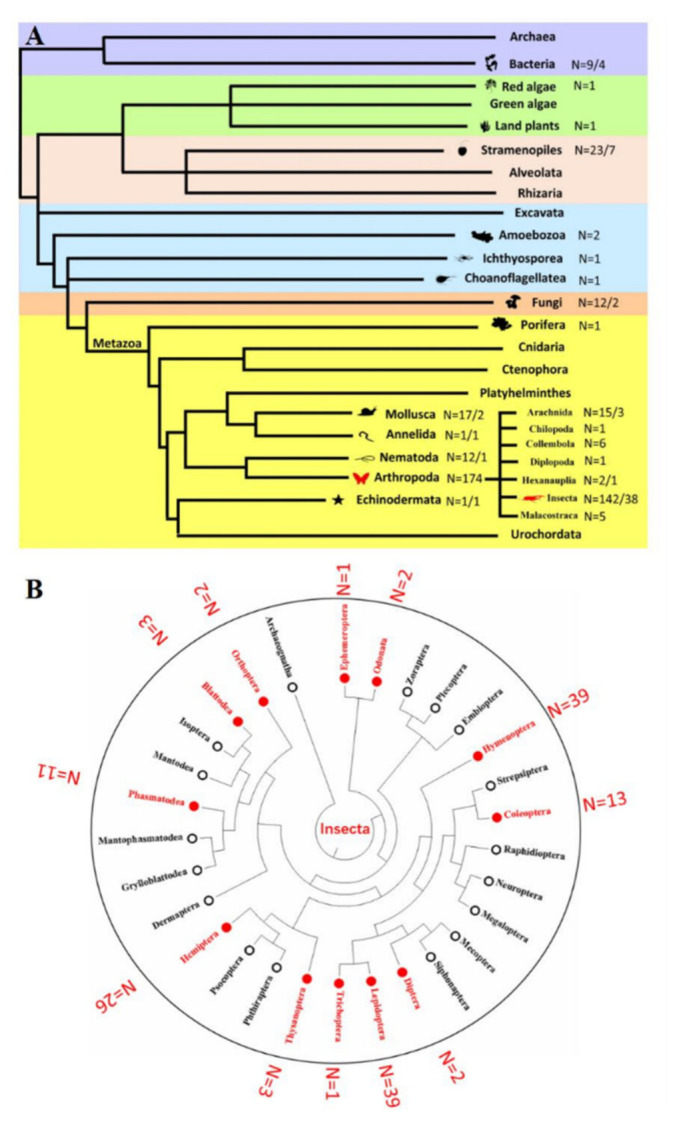
Taxonomic distribution of *Sailor*. (**A**) Taxonomic distribution of *Sailor* elements in the different kingdoms. The numbers next to the animal silhouettes represent the number of *Sailor* elements detected in the species of each lineage. The number after the slash represents the intact *Sailor.* (**B**) Taxonomic distribution of *Sailor* elements in Insecta. The taxonomic tree represents the distribution of the species identified in Insecta in their respective orders. Insecta orders are labelled with a square node and the number of Insecta species is shown outside the circle. The phylogenetic relationships were taken from the TimeTree database (http://timetree.org/, accessed on 5 June 2021) [42].

According to Cavalier-Smith (1998) [39], the tree of life is subdivided into six kingdoms: Bacteria/Archaea, Chromista, Plantae, Protozoa, Fungi and Animals. Representatives of the new superfamily DD82E/*Sailor* were found in six kingdoms (Figure 3 and Table 1 and Table S6). For the species in which DD82E/*Sailor* was identified, the classification, structural characteristics, sequences and genome coordinates in each genome are listed in Table S6. Among Protozoa, only a few cases of the presence of DD82E/*Sailor* were identified: Amoebozoa (two species), Ichthyosporea (one species) and Choanoflagellatea (one species); in plantae, only two cases of the presence of DD82E/*Sailor* were identified: red algae (one species) and land plants (one species). The representation of the new transposons among Bacteria, Chromista (Stramenopiles) and Fungi was slightly higher; DD82E/*Sailor* transposons were found in nine, 23 and 12 species, respectively. The animal kingdom was much richer regarding the representation of DD82E/*Sailor* elements. Of note, although DD82E/*Sailor* transposons were common among invertebrates (206 species), including Porifera, Mollusca, Annelida, Nematoda, Arthropoda and Echinodermata, they were not found in vertebrates. A predominant proportion of elements of this superfamily was found in Protostomia (Mollusca (17 species), Annelida (one species), Nematoda (12 species) and Arthropoda (174 species)). In some species, DD82E/*Sailor* elements were found in the taxa Deuterostomia: Echinodermata (one species) and Porifera (one species). Among arthropods, DD82E/*Sailor* transposons were also not uniformly distributed; a clear predominance of their representation in insects was noted (Figure 3 and Table 1 and Table S6). Uniformity was also not observed within the class of insects, as the studied TEs were found only in 12 out of 29 taxa (Figure 3B). This mosaic distribution along the tree of life indicates a rich evolutionary history woven from elimination events and horizontal transpositions. The latter phenomenon is a fairly common feature of the *ITm* infraclass elements [40,41]. When counting the number of copies of the elements, only sequences with homology to more than 40% of the length of the representative copy and identity of more than 80% were considered. This analysis showed that the overall proliferation of *Sailor* was not significant (Table S6). In most cases, the number of copies ranged from a few to several dozen. However, in some representatives of Mollusca and Arthropoda, the number of copies of *Sailor* exceeded 100. The gastropod Mollusca *Haliotis rubra* (219) and representatives of the order Phasmatodea Arthropoda (83–341), in particular *Timema cristinae* (341), were especially rich in copies of *Sailor*. Furthermore, intact copies of *Sailor* were detected in four species in prokaryotes and in 55 species of 19 orders in eukaryotes, indicating recent insertion into the genome and that some elements may still be active in these species or lineages (Table S6).

### 3.3. Distinct Structural Organization of DD82E/Sailor

**Figure 4:**
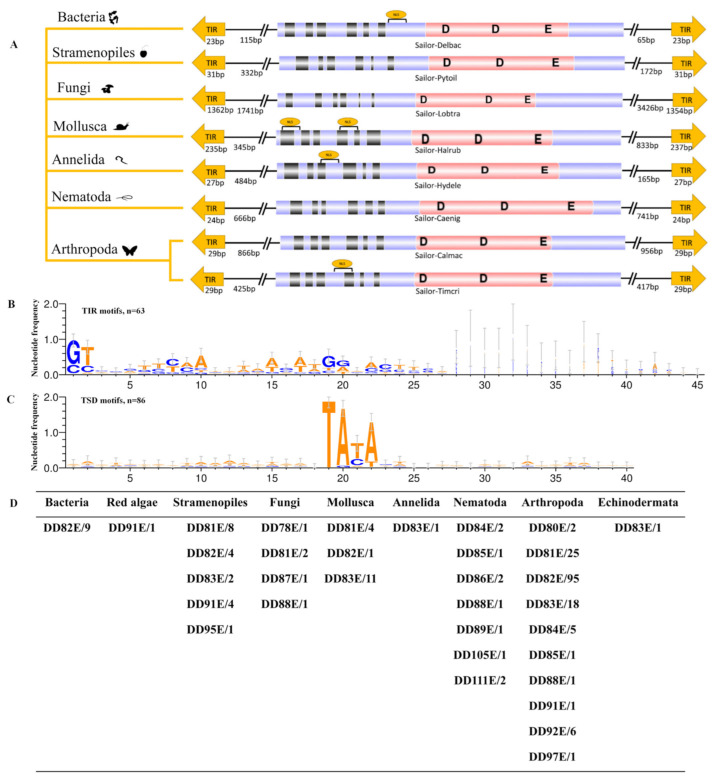
Schematic representation of the structure of *Sailor* transposons. (**A**) Structural organization of *Sailor* elements. The yellow arrows represent *Sailor*, the black rectangles represent HTH motifs, the yellow circle represents the NLS, the pink rectangles represent the catalytic domains, and the blue regions represent transposases (*Sailo*r-Delbac (*Deltaproteobacteria bacterium*), *Sailor*-Phyoil (*Pythium oligandrum*), *Sailor*-Lobtra (*Lobosporangium transversale*), *Sailor*-Halrub (*Haliotis rubra*), *Sailor*-Hydele (*Hydroides elegans*), *Sailor*-Caenig (*Caenorhabditis nigoni*), *Sailor*-Calmac (*Callosobruchus maculatus*), *Sailor*-Timcri (*Timema cristinae*)). (**B**) The WebLogo server (http://weblogo.berkeley.edu/logo.cgi/, accessed on 15 September 2021) was used to create the logo representation of the TIR (≤45 bp) sequences. The value 2 (log2 4) on the y axis stands for maximum possible frequency. (**C**) The WebLogo server (http://weblogo.berkeley.edu/logo.cgi/, accessed on 25 April 2021) was used to create the logo representation of the TSD sequences. (**D**) The table shows the situation of different kingdoms of DDxE, with the number placed after the slash representing the number of DDxE.

The structural organization of *Sailor* transposons retained the features of classic TIR TEs in all groups of organisms (Table S6). The structure of representative *Sailor* transposons is shown in Figure 4A and Figure S4. Full-size *Sailor* exhibited high variability in length (1379–6979 bp). Concomitantly, the elements of Bacteria were not very long (1379–1437 bp), whereas in some representatives of Stramenopiles, Porifera and Arthropoda, the length of *Sailor* exceeded 4 kb, and in Fungi it almost reached 7 kb (Table 1). In general, a length varying from 1.3 to 2.5 kb is typical of the representatives of the *Tc1/mariner* superfamily, whereas a length greater then 4 kb is more common among the DDxD/*pogo* superfamily [24,26,30]. Significant variations in the length of TIRs were also observed: “classic” TIRs (23–57 bp) were detected in most of the studied species, and extra-long TIRs (1362 bp) were found in *Lobosporangium transversale* (Fungi). The latter explains the colossal size of the TE itself (6979 bp). Very long TIRs were observed in representatives of DD41D/*VS* elements [24]. The first two nucleotides of *Sailor* TIRs are usually “GT” and “CC” di-nucleotides, and two conserved motifs (5–10 and 15–25 bp), which may be corresponding to the transposase recognition sequences, were identified for *Sailor* TIRs (Figure 4B). TSDs that predominantly shared *Sailor* contained four TATA nucleotides, in contrast to the classical TA dinucleotide present in *Tc1/mariner* elements (Figure 4C). Full-length *Sailor* elements had ORFs encoding transposases from amino acid residues 323 to 676. The longest amino acid sequences of the enzyme (over 500 amino acid residues) were detected in Stramenopiles, Arthropoda and Fungi (Table 1). In intact transposases, a DNA-binding domain, a catalytic domain and, in some cases, an NLS were observed (Figure 4A and Figure S4). The DNA-binding domain present in representatives of different taxa showed sufficient conservation. Concomitantly, although the catalytic domain had the DD82E signature in an overwhelming majority of cases, it exhibited high variability across taxa (DD78E-DD111E) (Figure 4D). The occurrence of a domain with the DD83E signature was also frequent (Stramenopiles, Mollusca, Annelida, Arthropoda and Echinodermata). *Sailor* transposases with the DD82E signature were not found among red algae, Fungi, Annelida, Nematoda and Echinodermata. Catalytic domains with a spacer between “D” and “E” of more than 90 amino acid residues were found in red algae, Stramenopiles, Nematoda and Arthropoda. Multiple alignment of the amino acid sequence of the catalytic domain of the *Sailor* elements together with DD34E/*Tc1*, DD35E/*TR*, DD36E/*IC*, DD37E/*TRT* and DD38E/*IT* showed that the increase in the length of the spacer between “D” and “E” was apparently caused by two insertion events of 13 and 36 aa (Figure 1). In addition, in the spacer located between “D” and “D”, an insert with a length of 12–15 aa was also detected. In *Sailor* transposons, the region upstream of “E” exhibited noticeable differences from the homologous region of *Tc1* and *Tc1-like* elements, which may indicate a rather long-standing divergence, because this region is conserved in the Tc1/mariner superfamily. These insertion motifs were analyzed by the Pfam and HMMER web server, and we did not find any homology domain, and therefore their function remains unknown.

### 3.4. Evidence of HT Events of DD82E/Sailor between/within Prokaryotes and Eukaryotes

**Figure 5:**
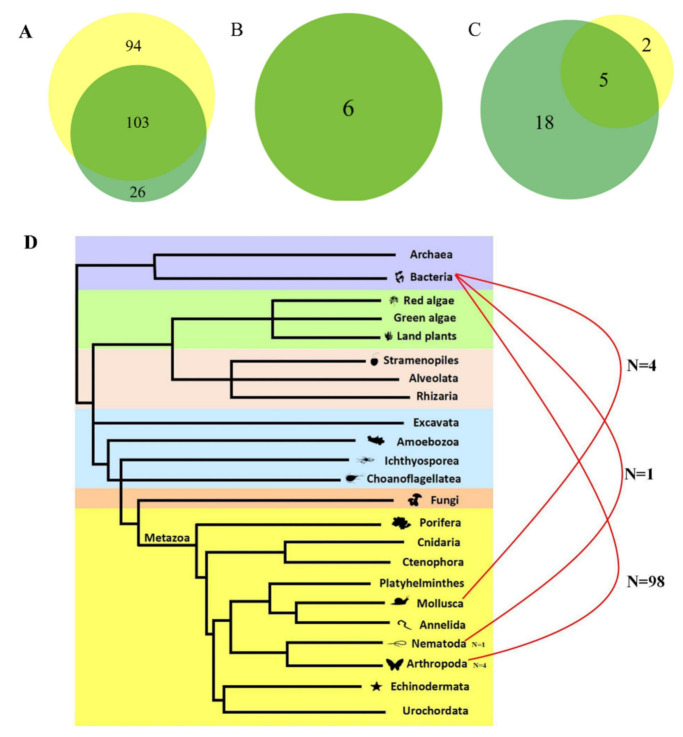
HT events of *Sailor*. (**A**) The two organelle ribosomal proteins (L3 and L4) exhibited prokaryotic to eukaryotic HT events. The yellow color represents the HT characteristic of the organelle ribosomal protein L3, the dark-green color represents HT common to the organelle ribosomal proteins L3 and L4, and the light-green color represents the HT characteristic of the organelle ribosomal protein L4. (**B**) The two cytoplasmic ribosomal proteins (L3 and L4) exhibited HT events in prokaryotic species. The dark-green color represents HT common to the cytoplasmic ribosomal proteins L3 and L4. (**C**) The two cytoplasmic ribosomal proteins (L3 and L4) exhibited HT events in eukaryotic species. The yellow color represents the HT characteristic of the cytoplasm ribosomal protein L3, the dark-green color represents HT common to the cytoplasmic ribosomal proteins L3 and L4, and the light-green color represents the HT characteristic of the cytoplasm ribosomal protein L4. (**D**) HT based on the pairwise genetic distance comparisons of two proteins. The numbers next to the animal silhouettes represent species pairs supporting HT events in the same phylum, and the numbers behind the curve represent species pairs supporting HT events from prokaryotic to eukaryotic species.

Pairwise genetic distance comparisons between the host genes and the transposons were used to identify putative horizontal transfer events of *Sailor* elements. Considering the deep phylogenetic relationships in prokaryotes and eukaryotes, the conservation, protein length and taxonomic distribution of the single genomic copy of 29 ribosomal proteins, designated as the universally conserved genes [37], were evaluated to select the fit host genes for genetic distance calculation in the HT hypothesis test. An analysis of these data suggested that the L3 and L4 ribosomal proteins (cytoplasmic and organelle), which display higher sequence identities and are greater in length (Figures S1 and S2), and a wider taxonomic distribution of the single genomic copy across the three domains of Bacteria, Archaea and Eucarya (Table S1) were fit for the estimation of genetic distance between species as the host genes. The accession numbers and host species are listed in Table S2. The taxonomic distribution of the single genomic copy of the host gene across domains, which was evaluated using the online OrthoDB database (https://www.orthodb.org/, accessed on 5 May 2021), was compared and applied to minimize gene duplication in genomes, for improving the accuracy of genetic distance estimation between species. The genes that were homologous between prokaryote and eukaryote species were identified via online TBlastN or BLASTP search using the prokaryotic ribosomal L3 and L4 proteins as queries; all prokaryote L3 and L4 ribosomal proteins hit eukaryote organelle L3 and L4 ribosomal proteins, respectively. Thus, the prokaryote L3 and L4 ribosomal proteins were compared with eukaryote organelle L3 and L4 ribosomal proteins, to calculate the genetic distance of host genes between prokaryote and eukaryote species, whereas cytoplasmic L3 and L4 ribosomal proteins were used for the calculation of the genetic distance of host genes between eukaryote species. HT was considered to occur when the genetic distances of transposons were lower than those of both L3 and L4 host genes between species. Based on the pairwise genetic distance comparisons, overall, HT events were supported by more than 110 species pairs (Figure 5 and Figure S5 and Table S7), HTs from prokaryotes (four species) to eukaryotes (43 species) were supported by 103 species pairs: most HT events (98 species pairs) were identified between bacteria (four species) and Arthropoda (41 species), HTs between Deltaproteobacteria and arthropods were supported by 77 species pairs, HTs between Gammaproteobacteria and arthropods were supported by 21 species pairs, HT between bacteria and Nematoda, and HT between bacteria and Mollusca were supported by one and four species pairs, respectively (Figure 5A,D and Tables S4 and S7). HT events were also detected between species within prokaryotes (six species pairs), HT events between Gammaproteobacteria and Deltaproteobacteria were supported by three species pairs, HT events within Deltaproteobacteria were supported by three species pairs as well (Figure 5B,D and Tables S5 and S7). In addition, HT events of *Sailor* were detected between eukaryotic species (Figure 5C,D and Tables S5 and S7), including HT between species of Nematoda (one species pair) and HT between species of Arthropoda (four species pairs).

## 4. Discussion

### 4.1. Extensive Distribution of Sailor

The DNA transposons with the DD82E signature described in this work represent a new superfamily of transposons, which we called *Sailor* (Figure 2 and Table S6). This superfamily was phylogenetically closest to the *Tc1/mariner* superfamily and was part of a large group (infraclass) of DNA transposons known as *IS630-Tc1-mariner* (*ITm*) [26,29,30]. Representatives of the *Tc1/mariner* superfamily and the DD34E/*Gambol* and DDxD/*pogo* superfamilies (also referred to as *ITm* transposons) are found only in eukaryotes, whereas representatives of *Sailor* were identified among both eukaryotes and prokaryotes, across the six natural kingdoms (Figure 3). Despite the wide coverage of natural kingdoms, the taxon distribution of *Sailor* was highly mosaic compared with that of *Tc1/mariner* and DDxD/*pogo*. In total, *Sailor* elements were identified in 256 species (Table S6). Concomitantly, intact copies were detected only in a quarter of organisms (four prokaryotes and 55 eukaryotes). The counting of the number of copies showed a rather weak amplification (with few exceptions) of *Sailor* elements. Nevertheless, there were species in which more than 50 intact copies were identified in the genome. These were representatives of the order Coleoptera (Insecta; *Anoplophora glabripennis* and *Callosobruchus maculatus*) (Table S6). A large number of intact copies may indicate that the life cycle of these TEs is undergoing an amplification stage or has recently completed it. The TE life cycle includes the stages of invasion, amplification, diversification, inactivation (degradation) and elimination [43]. However, the life cycle may not always be complete. Variants are possible when TEs are immobilized and cannot proceed to the amplification stage or are weakly amplified and degrade relatively quickly. In addition, TE “resurrection” options are possible: horizontal transfer or intragenomic life-cycle restart [23,44]. Moreover, it is also possible to avoid “death” via the transition from the “wild” to “domestication” state, i.e., co-optation of the TE gene by the host genome [10,45].

### 4.2. Structural Organization of Sailor

The *Tc1/mariner* transposons were closest to the *Sailor* transposons on the phylogenetic tree (Figure 2). The study of the structural organization of *Sailor* elements allowed us to highlight several obvious differences from the representatives of the *Tc1/mariner* superfamily (Figure 1 and Figure 4). The most striking difference was undoubtedly the DDE signature. *Tc1/mariner* elements are characterized by the presence of the DD34-41E/D signature [20,21,22,23,24,27,28,29,30]. Although the *Sailor* superfamily was shown to contain the DD82E catalytic domain, this characteristic varied widely across *Sailor* members (DD78-111E) (Figure 4C). Concomitantly, the occurrence of the DD83E signature was also quite frequent, and the DD82E signature was not found among some taxa. Presumably, the lengthening of the catalytic domain occurred as a result of two independent insertions into the spacer located between “D” and “E” (Figure 1). Increasing the length of the spacer between “D” and “E” did not stop *Sailor* elements from spreading through the tree of life. Moreover, this spread did not reach a wide scale (Figure 3 and Table 1). Another important difference between *Sailor* and *Tc1/mariner* transposons was the TSD modification. Elements of the *Tc1/mariner* superfamily and members of the entire *ITm* infraclass recognize the TA dinucleotide and duplicate it as a result of insertion. This process provides an AT-hook (GRPR-motif) located in the transposase between the first and second triad of α-helices of the DNA-binding domain [19]. The TSDs in the majority of *Sailor* elements had four TATA nucleotides (Figure 4B). It is likely that, in the *Sailor* transposases, the motif that provides the connection with the TSD has also undergone changes. This change in the specificity of the insertion could have influenced the proliferation of *Sailor* transposons, as the number of potential insertion sites was significantly reduced. There were noticeable differences in the amino acid sequence of the conservative loci of the catalytic domain of transposases. For example, the region located around the first “D” in *Sailor* was I/VVYLDET, the consensus amino acid sequence around the second “D” was VIIMDNA, and the consensus sequence around the “E” was HCELNPIEL (Figure 1). By contrast, in *Tc1/mariner*, the corresponding loci are VLWSDES, IFQQDNA/D and SPDLNPIEN. The total length, the length of TIRs and the length of the transposase of *Sailor* elements exhibited a fairly high variability (Table 1); however, this phenomenon is often observed in different groups of the *ITm* infraclass [20,21,22,23,24,27,28,29,30]. The totality of all of these established differences in structural organization suggests that *Sailor* transposons have passed a long independent evolutionary path. In combination with the data of the phylogenetic analysis and the identified cases of HT, it is most likely that *Sailor*, as a separate evolutionary group, was formed in prokaryotes and then spread along the tree of life as a result of HT.

### 4.3. HT Events of DD82E/Sailor between/within Prokaryotes and Eukaryotes

Although a large number of cases of prokaryote-to-eukaryote gene HTs have been identified [46,47,48,49], and prokaryote-to-prokaryote [50] and eukaryote-to-eukaryote cases of TE HT have been reported extensively [20,21,22,51,52,53,54], very few TEs are known to have jumped from prokaryotes to eukaryotes. One likely such event has been reported, in which several bacterial TEs were found embedded in larger genomic fragments that were transferred horizontally from bacteria to eukaryotes [55]. In another study, typical prokaryote-to-eukaryote HT of TEs were characterized, although they occurred at relatively low frequency [47]. The current study revealed that *Sailor* elements are apparently characterized by a high frequency of cases of horizontal transfer from prokaryotes to eukaryotes. In total, HTs were supported by more than 110 species pairs based on the genetic distance comparisons between transposons and the two host genes. HT from prokaryotes to eukaryotes were supported by over 100 species pairs (Figure 5A), and most HT events occurred between bacteria and invertebrate (Arthropoda) species. Whereas, a low frequency of HT was observed within eukaryotes, which may also explain why *Sailor* transposons are more common in invertebrates, but absent in vertebrates. The *Tc1/mariner* superfamily is the record holder among TEs for established HT cases [40,41]. It is likely that *Sailor*, as part of the *ITm* Infraclass, inherited the ability of undergoing frequent HT. Since the discovery of HT, many articles have been published describing this phenomenon in various taxa of eukaryotes (plants, insects, reptiles, mammals and others). It has been shown that these events can occur both between closely related species and distant taxa [40,54,56,57,58,59,60]. To date, about 3000 HT events have been described, with about a third of them being associated with elements of the *Tc1/mariner* superfamily [41]. Despite the abundance of literature on and the high number of detected cases of HT transposons, the mechanism underlying this phenomenon remains unclear. Questions about the probability of generation of adaptive insertions into the recipient’s genome and about their contribution to the evolution of genomes and speciation also remain open. However, the identification of new cases of HT will help expand our knowledge of this phenomenon and bring us closer to solving the issues discussed above. The identification of HT cases between prokaryotes and eukaryotes demonstrates the possibility of exchange of genetic information between two different domains of life. In addition, in the current study, the HT events tended to be underestimated because we applied universally conserved genes, and some host gene annotations were not available. Furthermore, we only used the intact transposons to perform the HT test for accuracy. Thus, we did not define the number of independent HT events of Sailor in this report. The lower number of HTs of Sailor observed in arthropoda using the pairwise genetic distance comparisons based on L3 and transposon compared with those of L4 and transposon was attributed to the lower number of L3 proteins (only three species) vs. L4 proteins (seven species) annotated in this lineage.

## 5. Conclusions

A superfamily (DD82E/*Sailor*) of *ITm* transposons was discovered in this study that displayed a distinct structural organization and phylogenetic position compared with the known groups of *ITm*, including *Tc1/mariner*, DDxD/*pogo* and DD34E/*Gambol*. Moreover, they were distributed in both prokaryotic and eukaryotic organisms, and HT events of these elements may have occurred from prokaryotic to eukaryotic organisms and between different kingdoms. This observation not only improves our understanding of the evolution of the *Sailor* superfamily, but also expands our understanding of the diversity of *ITm* transposons and updates the classification of this group.

## Figures and Tables

**Table 1:** Taxonomic Distribution of *Sailor.*

Taxa Distribution	Number of Species Containing *Sailor*	Number of Species Containing FL *Sailor*	Number of Species Containing Intact *Sailor*	Length of FL *Sailor*	Length of Intact *Sailor*	Tpase Length of Intact *Sailor*	TIR Length of Intact *Sailor*
Bacteria	9	4	4	1379–1437	1379–1437	376–422	23–57
Red algae	1	1	–	3034	–	–	–
Land Plants	1	–	–	–	–	–	–
Stramenopiles	23	8	7	1757–4441	1757–4441	323–507	24–57
Amoebozoa	2	–	–	–	–	–	–
Ichthyosporea	1	–	–	–	–	–	–
Choanoflagellata	1	–	–	–	–	–	–
Fungi	12	2	2	2427–6979	2427–6979	368–607	52–1362
Porifera	1	1	–	4115	–	–	–
Mollusca	17	4	2	2220–2465	2220–2465	338–432	34–237
Annelida	1	1	1	1927	1927	429	27
Nematoda	12	2	1	2754	2757	452	24
Arthropoda	174	65	41	1799–4408	1799–4408	334–676	18–61
Echinodermata	1	1	1	2763	2763	334	28

Description of *Sailor* elements in 14 lineages, including the number of species with these elements, full-length (FL) elements, amino acid (aa) length of the transposase (Tpase), and length of terminal inverse repeats (TIRs).

## Data Availability

The data presented in this study are available on request from the corresponding author.

## Supplementary Materials

The following are available online at https://www.mdpi.com/article/10.3390/biology10101005/s1, Figure S1. Sequence identity of 29 universally conserved ribosomal proteins. (A) Organelle ribosomal proteins, (B) cytoplasmic ribosomal proteins. The original data are listed in Table S8. Figure S2. Sequence length of 29 universally conserved ribosomal proteins. (A) Organelle ribosomal proteins, (B) cytoplasmic ribosomal proteins. The original data are listed in Table S9. Figure S3. Uncollapsed phylogenetic position of the *Sailor* superfamily. Figure S4. Motif prediction for *Sailor* transposases. This analysis was performed using multiple alignment with Bioedit and with modifications in Genedoc. The green box represents the range of the α-helix of all sequences; the red box represents the range of DDE sequences; the red font represents the NLS sequence. (*Sailor*-Delbac: *Deltaproteobacteria bacterium*; *Sailor*-Pytoli: *Pythium oligandrum*; *Sailor*-Lobtra: *Lobosporangium transversal*; *Sailor*-Halrub: *Haliotis rubra*; *Sailor*-Hydele: *Hydroides elegans*; *Sailor*-Caenig: *Caenorhabditis nigoni*; *Sailor*-Calmac: *Callosobruchus maculatus*; *Sailor*-Timcri: *Timema cristinae*) Figure S5. HT events of *Sailor*. (A) The distances were obtained from all possible pairwise comparisons L3 (cytoplasm ribosomal protein L3 in Prokaryotic and organelles ribosomal protein L3 in eukaryotic) and *Sailor.* (B)The distances were obtained from all possible pairwise comparisons L4 (cytoplasm ribosomal protein L4 in Prokaryotic and cytoplasm ribosomal protein L4 in eukaryotic) and *Sailor*. (C) The distances were obtained from all possible pairwise comparisons L3 (cytoplasm ribosomal protein L3) and *Sailor*. (D) The distances were obtained from all possible pairwise comparisons L4 (cytoplasm ribosomal protein L4) and *Sailor*. (E) The distances were obtained from all possible pairwise comparisons L3 (cytoplasm ribosomal protein L3) and *Sailor*. (F) The distances were obtained from all possible pairwise comparisons L4 (cytoplasm ribosomal protein L4) and *Sailor*. Table S1. Number of single copy species, species and genes of 29 universally conserved ribosomal proteins. Table S2. Accession numbers of 29 generally conserved ribosomal proteins. Table S3. Accession numbers of ribosomal proteins of species involved in horizontal transmission. Table S4. Distance between *Sailor* and host genes (from prokaryotic to eukaryotic organisms). Table S5. Distance between *Sailor* and host genes (in prokaryotes and eukaryotes). Table S6. Details of *Sailor*, including species distribution, protein length, TIR, etc. Table S7. HT event statistics of *Sailor*. Table S8. The sequence identity statistics of 29 universally conserved ribosomal proteins. Table S9. The sequence length statistics of 29 universally conserved ribosomal proteins. Data S1. *Sailor* transposons. Data S2. Multiple alignment of catalytic domains including reference families of DD34E/*Gambol*, DD34E/*Tc1*, DD35E/*TR*, DD36E/*IC*, DD37E/*TRT*, and DD38E/*IT*. Data S3. Multiple alignments of the organelle ribosomal protein L3. Data S4. Multiple alignments of the organelle ribosomal protein L4. Data S5. Multiple alignments of the cytoplasmic ribosomal protein L3. Data S6. Multiple alignments of the cytoplasmic ribosomal protein L4. Data S7. Multiple alignment of *Sailor* (organelle ribosomal protein L3). Data S8. Multiple alignment of *Sailor* (organelle ribosomal protein L4). Data S9. Multiple alignment of *Sailor* (cytoplasmic ribosomal protein L3). Data S10. Multiple alignment of *Sailor* (cytoplasmic ribosomal protein L4).
