# Supplementary material for: Prokaryotic and Eukaryotic Horizontal Transfer of Sailor (DD82E), a New Superfamily of IS630-Tc1-Mariner DNA Transposons
Source: Biology (Basel). 2021 Oct 7;10(10):1005. doi: 10.3390/biology10101005 (PMC8533490; doi:10.3390/biology10101005)
Supplement: Supplementary file 1 [file biology-10-01005-s001.zip › Supplementary Material/Supplementary Figure S4.pdf]

```
Sailor-Delbac : MSFQG------------------------------------------------------------KEITYGMKQLVINLRQFND-IERS-----KNNLKAVWAVEQTAKG   44
Sailor-Pytoil : MTSSSGGTGPPKR----------------------------------------------------KRRFRMISHDTAVANLCRTYFE-REAS----DNSRRNLSRPLDRTAEC   57
Sailor-Lobtra : MSSAQFSLIPKELSAEQASRRSESFIYEESNQSMFSQEHENDNPFLISMDEGGLHAPPRLSKTTVAEEEYRLLAHLYAKLSNAPPEFQKWMRQSFGMKECV-QMAKL  106
Sailor-Halrub : M----------------------------------------------------------------KAYSDDAKRLIRNVLNFFK-KESKR---SKPIYKVSDYMKRTTFA   42
Sailor-Hydele : MD-------------------------------------------------------------KAKYNVISSKVKKLVYLVYKYFK-EEKKL---GNTKIPVTNCIERVQDL   47
Sailor-Caenig : MNLEALNTT------------------------------------------------------KLEKKVISSGN-EIINNLLKSME-SIRHLLGHDSELTPFRNPFIAAAAI   56
Sailor-Calmac : MQQGTSVSNT-----------------------------------------------------ASKRHVISTGERNIVKNIYDGVRTRFPE--------RPVKEIVKLCSDL   51
Sailor-Timcri : MESPACYKQL-----------------------------------------------------HSKRKIISSKERAMVLRLYTYFR-KDGT--------CSVTQAAMATAEA   50
```

Helix1 Helix2

```
Sailor-Delbac : LGIGQATVR---RITMAEYNKNQQNIP--GNAPKTRGKPEYIIPQNLQSIVRKYIRSQNLKGQHVSVEEVLQHLLTIS----SDYNFPTTTLWRALNRWGFTYGIGKR  142
Sailor-Pytoil : FDISRQTVM---QIRKKLESGEPFYD------EEERDRAMEVPPSFAGDVRDAIFDMYAAKQHVTLDTLLKKLDKRVTRTSGWKWSRSTLYRLLTD-TMHYTXGKR  154
Sailor-Lobtra : TGVNAHSCL---AAINYSRTGVLKLPNKGVGRGRKAQAK---DKVLQGHITDIIRRTNLGGIPNTLGEVVNELRNEH-----GIRTSRSTVHRYLHASNFYWGKGVR  202
Sailor-Halrub : LGMKKNIVL---KIVKNKPTTNK-------QNLVQKGRHPKLDSFDIRVIRDVIRRLFARNEHLSTRKLKHILAEDH-----DINVSRFMLSKLLLKFGYKYKKVAN  134
Sailor-Hydele : FKISQSTLS---RIVKKCKNGHE-------PRRKCAKRKEKFDSFQKDVIKRALYGFYERNETVTSKRLKIILEKKH-----DIVISKSTLVKMIKSIGFKYKKRSG  139
Sailor-Caenig : VGVHKNIIRFHGTFGSSLKRCVNHLN----GRDRDRYEASKLTIGQQTRIRKEIHSRWANDKPVSVDDMFEWAKSNA-----FFNRGRTSFFRAMRALGFRHRKNNN  154
Sailor-Calmac : TKVSVAIIF---RILKEKKRDVVFVV--KSLKQKRGVKKIVLDGDVKNAIRRKVHSFYLRNELPTIKKLQQSIQNDE----SLPKISRRVLLRTLREINIRYLKRKR  149
Sailor-Timcri : TGVSYKLVI---SIKQEFKTKGKLLTPIKRNNSTQMKRLDKYDSFTQAAIRRKVHSLYRNNELPTLKKIKALIDTDS----DLPNFSLTTLQRLLIDIGFKFIRRKR  150
```

Helix3 Helix1 Helix2 Helix3

```
Sailor-Delbac : RS---ALKERDYVILALRRYL-RQKRLNRN--SDGSFIRAEVYLDETFINKNHSNQFTWYLDEDGP--------------DVNKPSGKGERLII---------VNAI  220
Sailor-Pytoil : RTYYQNLKENVSIAAQRVAYI-KKVQEYRA--EG----RPIFVQDETWVNKNXTPANVWLLDDEGQG--------------GRAVPQGKGERSIV---------CHIG  231
Sailor-Lobtra : RN---FMHDAPHIVQYRLKYLTERFKNLVELDDGLIPMMPEVVFLDESYCHLDHSSKNRWVERDGGIVCESSRQPLLVMFAAFVIYYDRDEQKVVGKFVENSVHIWAA  306
Sailor-Halrub : QK--LVMCERSDIVKIRCNYL-REIKQARE--DG----FEPIVLDETWVNANHTAARQWLPTDPSD--------------GRKIPTGKGERLIV---------LHAG  209
Sailor-Hydele : NR--EVICERSDLVRARANYL-HSIKAFRE--DG----YTIVYTDETWVNACHTAPLQWNPPDPKN--------------ARNIPTGRGRRLIV---------LHAG  214
Sailor-Caenig : NS---VLEERDIVRWRRLYL-GKKEALDA--EG----VMFFALDETWFHDAMAKLFAWQSQYFNMCKKMRMVDPSLPMAGPRKGKLKGKRAIC---------IAVV  242
Sailor-Calmac : NS---ALIERDDIVLWRREYL-RKIRSIRN--AGG---IKIYYLDETWLNEGHTVTRCWQDLNIASRRQAHLEGLST---GLKCPSGKGCRLII---------THIG  235
Sailor-Timcri : NS---LLIDRDDILAWRHRYL-RAIKKHRE--SN----RNIVFTDETWCNSGHTTSKSWMDTTIASSSQARREGLTT---GNKLPSGKGGRVIL---------VHAG  235
```

D

```
Sailor-Delbac : -TLDGWVDKAQLVFEA----KKKTGDYHGQMDWGNFSKWFIEQLLPNIPA-------NSIIIMDNASYHNTTEDN---------------------TFPKSNTKKE  293
Sailor-Pytoil : -GRDGFVDGAKLIFRG--SKALKDSDYHTEMNSSVFLDWMRRKVLPAVPS-------RSVIVIDRATYHTTLTEQT---------------------KPAKSTFRKA  307
Sailor-Lobtra : -NSKASLQGNADHMQPNSQILANDHDYHGHFNHEVFEELFTTVCQSLVKMNLG----PCKIHMDGARYHFHCPD----------------------RKPTAANKKS  385
Sailor-Halrub : -SHRGFLPGCDLVFRS---KSTDGRDYHTEMNSTVFTQWVEEQLLPALPY-------KSIVVMDNAPYHSVRDPNN---------------------RCPTSNTKKS  284
Sailor-Hydele : CAEKGFLDGCDLVFEA---KSRDNRDYHTEMNGEVFLNWLQYQLLPALPP-------KSVVVMDNAPYHSMLAVES---------------------RCPTSGTRKR  290
Sailor-Caenig : -SPSGILPGSILVIISGVNAAEQTEDYHKDVCGSTYETYFK-KVVPLMAAEAAKQNKRAVLLVDNAPYHNKISKKVFRISRQILGNFLGNISRQFSLQPTTGTSKQ  347
Sailor-Calmac : -SESGFVDGGLNVFES-----RKTGDYHEDMDAVVFESWFK-SILTKLEP-------GSVIVLDNASYHSRRLE----------------------ALPTSNWKKA  305
Sailor-Timcri : -NENGFIPGAELIFHG-----KKDDDYHDEMDGKMYEKYFREKLLPNIPS-------NSVIVLDNASIHSTIKE----------------------SIPTKSSTKI  306
```

D H

```
Sailor-Delbac : NLRKWLDDKGIPWG--------KDLLKAELYALCKFFEPK-------PEYKI-DKIAEAACHSILRTPQYHPELQPIEMQWGVVKNYMAKH--CDFTLKKLRNNLPV : 382
Sailor-Pytoil : QLAEWLVAHGVVHNGMRTVDEYMTMTKPELGQLCKENKPK-------PEYEV-AVTAREFDCDVLFLPVGHPELNPIEMVWSYVKSYVAKH-NTDFSLSEVERLSHA : 405
Sailor-Lobtra : EIMEWLISHQFGIPPGSNGD--RTPSKSELLAYIRTLDYQ-------PRYTVWDIALENGCHWIIRTPPYHCELQPIELIWGVIKNSVAYMISGSMAADQLIQMLKE : 483
Sailor-Halrub : DMQQWLTNNNIQFS--------IKATKPQLYSLIKPNKPP-------PTYNI-DNMLRQHGHKVLRLPPYHCDLNPIELIWGDLKSTVARK-NQSFKLNDVKHIVNT : 374
Sailor-Hydele : DMQIWLEDMNIDFD--------PLMKKPELYDVIKANKPL-------PKYQV-DERIRAAGHEVLRLPPYHCNFNPIELIWADLKNTIGLD-NNTFKLDEVKKMVYD : 380
Sailor-Caenig : QLIDWLDEHNVPFD--------RTSKRPVLLAICKEFVNKNGGRSAFVKYEL-DSWAWQQGVQVLRIPPYHPDFNPIEMVWAQMKTHLRNVGAGSDSLEIVIQIFSR : 445
Sailor-Calmac : DIYDWLVSKNIECD--------MTMLKAQLSQLARHHKERY------IKLAV-DEMAREKGFTVLRLPPYHCELNPIELVWAQVKNEVARR-NTTFKLADIRILLEE : 396
Sailor-Timcri : IIQSWLTSKGISWE--------ETMNKMELVEILDKVRGNYE-----RKYII-DEIAKSACHEVLRLPPYHCELNPIEGIWSQIKIFVAQE-NNTFKLNDVHRLLVE : 398
```

P E

```
Sailor-Delbac : AFSQVTSKTCKKLIEKTVTEEN-RYWE----------------------------------------------------EDGEIDT-------NQGVDAK--------- : 422
Sailor-Pytoil : ALDSFDSEAWQRYVDHCVKVER-RYLE---------------------------------------------------------LADDVPLDMD------------- : 441
Sailor-Lobtra : LFRNVQVKTFVSAWKKTLSFAY-RYMQEIRGQTFETAEVQEELEQIFHSIDFSEATFNRLFGIGAARGRYNVLIEPMEDGDIGDEVDEVEEDVDLIPTSRSLAPDEL : 589
Sailor-Halrub : AICDIDIARWHACVEHVKKVEK-EYWL----------------------------------------------------KDGLKEQTVNPVVIRLDTDDSSVEEWSELD : 430
Sailor-Hydele : GFSRITRERWNNCVRHVVDVEEPRYWK----------------------------------------------------SDGL--TPISRVVINLQSDDD--------D : 427
Sailor-Caenig : NRAKIEV------------------------------------------------------------------------------------------------------ : 452
Sailor-Calmac : AIGAVTADNWQKCVSHVKAEEE-KFWK----------------------------------------------------LDPHIDSVIEPVIVNLGSDTTSDSDES--F : 450
Sailor-Timcri : GVRNITKEHWRNHVHHVMEVEA-TMWD----------------------------------------------------VDGLIEKLHDSVVINLKDSSSSSEDDD--S : 452

Sailor-Delbac : ------------------ :  -
Sailor-Pytoil : -----------------D : 442
Sailor-Lobtra : EEDDNSMPASPSARFYRH : 607
Sailor-Halrub : EV---------------- : 432
Sailor-Hydele : DC---------------- : 429
Sailor-Caenig : ------------------ :  -
Sailor-Calmac : DCSSASDDEFFPPHTSATD : 468
Sailor-Timcri : DADLGVVP-------LPD : 463
```
