# Supplementary material for: Prokaryotic and Eukaryotic Horizontal Transfer of Sailor (DD82E), a New Superfamily of IS630-Tc1-Mariner DNA Transposons
Source: Biology (Basel). 2021 Oct 7;10(10):1005. doi: 10.3390/biology10101005 (PMC8533490; doi:10.3390/biology10101005)
Supplement: Supplementary file 1 [file biology-10-01005-s001.zip › Supplementary Material/Supplementary Material.docx]

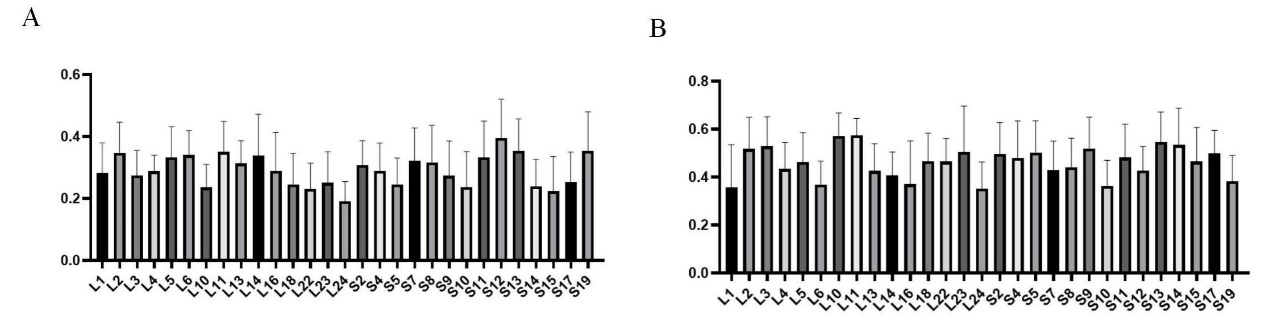


**Supplementary Figure S1.** The sequence identity of 29 universally conserved ribosomal proteins. (A) organelle ribosomal proteins, (B) cytoplasm ribosomal proteins. The original data is listed **Table S8**.


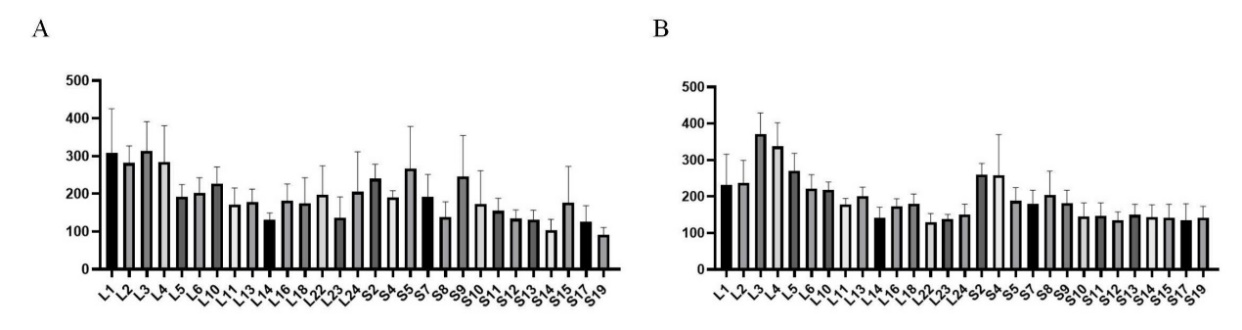
**Supplementary Figure S2.** The sequence length of 29 universally conserved ribosomal proteins. (A) organelle ribosomal proteins, (B) cytoplasm ribosomal proteins. The original data is listed **Table S9**.

**Supplementary Figure S3.** Uncollapsed phylogenetic position of the *Sailor* superfamily.

**Supplementary Figure S4.** Motif prediction for *Sailor* transposases. This analysis was performed using multiple alignment with Bioedit and with modifications in Genedoc. The green box represents the range of the α-helix of all sequences; the red box represents the range of DDE sequences; the red font represents the NLS sequence. (*Sailor*-Delbac: *Deltaproteobacteria bacterium*; *Sailor*-Pytoli: *Pythium oligandrum*; *Sailor*-Lobtra: *Lobosporangium transversal*; *Sailor*-Halrub: *Haliotis rubra*; *Sailor*-Hydele: *Hydroides elegans*; *Sailor*-Caenig: *Caenorhabditis nigoni*; *Sailor*-Calmac: *Callosobruchus maculatus*; *Sailor*-Timcri: *Timema cristinae*)


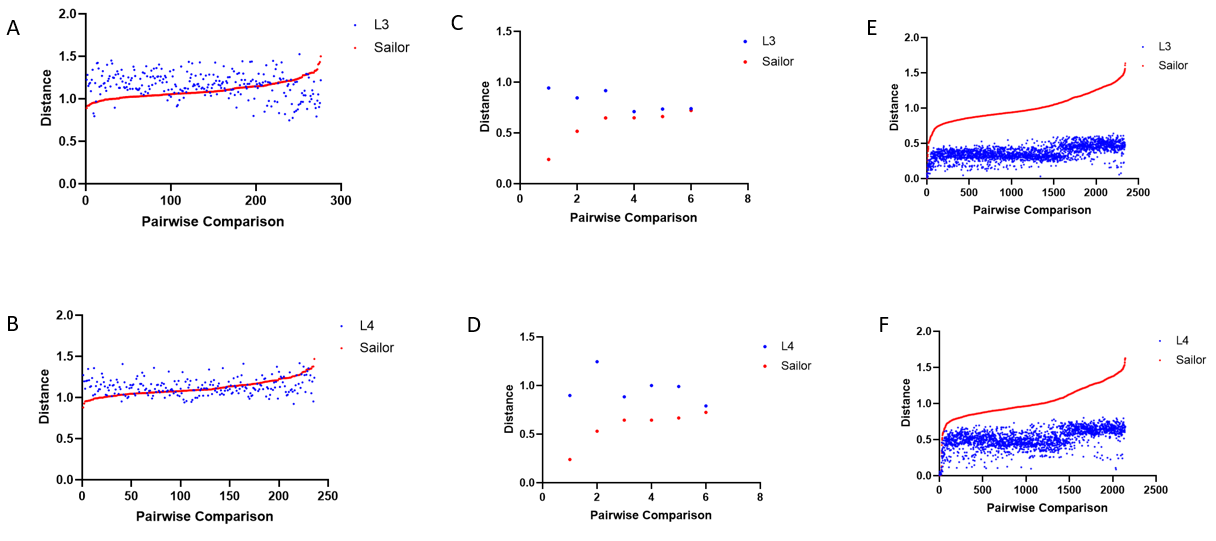


**Supplementary Figure S5.** HT events of *Sailor*. (A) The distances were obtained from all possible pairwise comparisons L3 (cytoplasm ribosomal protein L3 in Prokaryotic and organelles ribosomal protein L3 in eukaryotic) and *Sailor.* (B)The distances were obtained from all possible pairwise comparisons L4 (cytoplasm ribosomal protein L4 in Prokaryotic and cytoplasm ribosomal protein L4 in eukaryotic) and *Sailor*. (C) The distances were obtained from all possible pairwise comparisons L3 (cytoplasm ribosomal protein L3) and *Sailor*. (D) The distances were obtained from all possible pairwise comparisons L4 (cytoplasm ribosomal protein L4) and *Sailor*. (E) The distances were obtained from all possible pairwise comparisons L3 (cytoplasm ribosomal protein L3) and *Sailor*. (F) The distances were obtained from all possible pairwise comparisons L4 (cytoplasm ribosomal protein L4) and *Sailor*.

**Supplementary Table S1.** The number of single copy species, species and genes of 29 universally conserved ribosomal proteins.

| Name | Number of single copy species/species/genes | | | | |
| --- | --- | --- | --- | --- | --- |
|  | Bacteria | | Archaea | Eucarya | |
|  |  |  |  | Organelle | Cytoplasm |
| Ribosomal protein L1 | | 5577/5587/5597 | 397/399/401 | 925/1107/1379 | - |
| Ribosomal protein L2 | | 5527/5535/5543 | 398/400/402 | 113/1259/3230 | - |
| Ribosomal protein L3 | | 5571/5589/5580 | 392/393/394 | 953/1175/1498 | 804/1214/1874 |
| Ribosomal protein L4 | | 5542/5555/5568 | 400/401/402 | 888/1060/1307 | 1054/1255/1597 |
| Ribosomal protein L5 | | 5571/5584/5597 | 390/391/392 | 609/653/718 | 900/1196/1728 |
| Ribosomal protein L6 | | 5553/5563/5574 | 398/400/402 | 537/663/908 | 918/1204/1714 |
| Ribosomal protein L10 | | 5542/5553/5565 | 394/396/398 | 369/388/409 | 836/1189/2079 |
| Ribosomal protein L11 | | 5560/5590/5620 | 397/400/403 | 920/1095/1359 | 966/1230/1772 |
| Ribosomal protein L13 | | 5569/5578/5587 | 396/399/402 | 988/1163/1444 | 974/1216/1653 |
| Ribosomal protein L14 | | 5582/5594/5606 | 395/396/397 | 973/1123/1414 | 976/1198/1637 |
| Ribosomal protein L16 | | 5570/5577/5584 | 397/399/401 | 578/659/846 | 939/1181/1639 |
| Ribosomal protein L18 | | 5522/5532/5542 | 396/398/400 | 476/589/763 | 877/1138/1619 |
| Ribosomal protein L22 | | 5548/5557/5566 | 393/394/397 | 989/959/1148 | 788/1213/1976 |
| Ribosomal protein L23 | | 5548/5559/5570 | 393/394/395 | - | 960/1208/1708 |
| Ribosomal protein L24 | | 5530/5541/5552 | 398/399/400 | 907/1110/1413 | 101/1260/2975 |
| Ribosomal protein S2 | | 5530/5536/5542 | 397/400/404 | 925/1009/1228 | 985/1250/1705 |
| Ribosomal protein S4 | | 5390/5596/5840 | 393/397/401 | - | 902/1244/2067 |
| Ribosomal protein S5 | | 5573/5582/5591 | 395/397/399 | 487/500/514 | 961/1203/1664 |
| Ribosomal protein S7 | | 5581/5591/5601 | 399/400/401 | 886/995/1251 | 948/1206/1648/ |
| Ribosomal protein S8 | | 5578/5591/5604 | 399/401/404 | 586/613/667 | 954/1198/1704 |
| Ribosomal protein S9 | | 5563/5574/5585 | 399/400/401 | 957/1108/1341 | 956/1183/1613 |
| Ribosomal protein S10 | | 5561/5570/5579 | 400/401/402 | 948/1025/1142 | - |
| Ribosomal protein S11 | | 55835593/5603 | 387/389/391 | 873/990/1235 | 949/1216/1715 |
| Ribosomal protein S12 | | 5562/5572/5582 | 399/400/401/ | 919/1070/1384 | 966/1184/1561 |
| Ribosomal protein S13 | | 5578/5587/5596 | 400/402/404 | 517/611/786 | 990/1238/1730 |
| Ribosomal protein S14 | | 4667/5328/6020 | 348/377/406 | - | 936/1014/1130 |
| Ribosomal protein S15 | | 5576/5584/5596 | 395/398/401 | 1054/1147/1308 | 1007/1217/1659 |
| Ribosomal protein S17 | | 5556/5566/5576 | 398/400/412 | 717/859/1395 | 919/1209/1728 |
| Ribosomal protein S19 | | 5539/5546/5553 | 398/399/400 | - | 894/1169/1616 |

**Supplementary Table S2.** Accession numbers of 29 generally conserved ribosomal proteins.

**Supplementary Table S3.** Accession number of ribosomal protein of species involved in horizontal transmission.

**Supplementary Table S4.** The distance between *Sailor* and host genes (from prokaryotic to eukaryotic).

**Supplementary Table S5.** The distance between *Sailor* and host genes (in prokaryotic and eukaryotic).

**Supplementary Table S6.** Details of *Sailo*r include species distribution, protein length, TIR, etc.

**Supplementary Table S7.** HT events statistics of *Sailor*.

| **L3** | | | | | **L4** | | | **L3 and L4** | | |
| --- | --- | --- | --- | --- | --- | --- | --- | --- | --- | --- |
| **Class1** | **Class2** | | **Numbers of**  **Event** | | **Class1** | **Class2** | **Numbers of**  **Event** | **Class1** | **Class2** | **Numbers**  **of**  **Event** |
| **HT events from prokaryotic to eukaryotic statistics** | | | | | | | | | | |
| Bacteria | | Mollusca | | 6 | Bacteria | Stramenopiles | 13 | Bacteria | Nematoda | 1 |
| Bacteria | | Nematoda | | 1 | Bacteria | Mollusca | 8 | Bacteria | Mollusca | 4 |
| Bacteria | | Arthropoda | | 190 | Bacteria | Nematoda | 2 | Bacteria | Arthropoda | 98 |
| - | | - | | - | Bacteria | Arthropoda | 106 | - | - | - |
| **HT events in prokaryotic statistics** | | | | | | | | | | |
| Bacteria | | Bacteria | | 6 | Bacteria | Bacteria | 6 | Bacteria | Bacteria | 6 |
| **HT events in eukaryotic statistics** | | | | | | | | | | |
| Nematoda | | Nematoda | | 1 | Nematoda | Nematoda | 1 | Nematoda | Nematoda | 1 |
| Arthropoda | | Arthropoda | | 6 | Arthropoda | Arthropoda | 23 | Arthropoda | Arthropoda | 4 |

**Supplementary Table S8.** The sequence identity statistics of 29 universally conserved ribosomal proteins.

**Supplementary Table S9.** The Sequence length statistics of 29 universally conserved ribosomal proteins.

**Supplementary Data S1.** *Sailor* transposons.

**Supplementary Data S2.** Multiple alignment of catalytic domains including reference families of DD34E/*Gambol*, DD34E/*Tc1*, DD35E/*TR*, DD36E/*IC*, DD37E/*TRT*, and DD38E/*IT*.

**Supplementary Data S3.** Multiple alignments of the organelle ribosomal protein L3.

**Supplementary Data S4.** Multiple alignments of the organelle ribosomal protein L4.

**Supplementary Data S5.** Multiple alignments of the cytoplasmic ribosomal protein L3.

**Supplementary Data S6.** Multiple alignments of the cytoplasmic ribosomal protein L4.

**Supplementary Data S7.** Multiple alignment of *Sailor* (organelle ribosomal protein L3).

**Supplementary Data S8.** Multiple alignment of *Sailor* (organelle ribosomal protein L4).

**Supplementary Data S9.** Multiple alignment of *Sailor* (cytoplasmic ribosomal protein L3).

**Supplementary Data S10.** Multiple alignment of *Sailor* (cytoplasmic ribosomal protein L4).
