# Supplementary material for: Prokaryotic and Eukaryotic Horizontal Transfer of Sailor (DD82E), a New Superfamily of IS630-Tc1-Mariner DNA Transposons
Source: Biology (Basel). 2021 Oct 7;10(10):1005. doi: 10.3390/biology10101005 (PMC8533490; doi:10.3390/biology10101005)
Supplement: Supplementary file 1 [file biology-10-01005-s001.zip › Supplementary Material/Supplementary Table S4.pdf]

HT of Organelle ribosomal protein L3					HT of Organelle ribosomal protein L4					HT of Organelle ribosomal protein L3 and L4						
Species 1	Species 2	Distance of L3	Distance of Sailor	Difference of Sailor and L3	Species 1	Species 2	Distance of L4	Distance of Sailor	Difference of Sailor and L4	Species 1	Species 2	Distance of L3	Distance of L4	Distance of Sailor	Difference of Sailor and L3	Difference of Sailor and L4
Ba-Delbac	Ar-Acypis	1.2679	1.1770	0.0908	Ba-Delbac	Ar-Acypis	1.1558	1.1551	0.0007	Ba-Delbac	Ar-Acypis	1.2679	1.1558	1.1551	0.1128	0.0007
Ba-Delbac	Ar-Adohon	1.2715	1.0507	0.2207	Ba-Delbac	Ar-Adohon	1.1270	1.0393	0.0877	Ba-Delbac	Ar-Adohon	1.2715	1.1270	1.0393	0.2322	0.0877
Ba-Delbac	Ar-Ampamp	1.0585	1.3081	-0.2496	Ba-Delbac	Ar-Ampamp	1.2835	1.2916	-0.0082	Ba-Delbac	Ar-Amytra	1.3248	1.1228	1.0919	0.2329	0.0309
Ba-Delbac	Ar-Amytra	1.3248	1.0533	0.2715	Ba-Delbac	Ar-Amytra	1.1228	1.0919	0.0309	Ba-Delbac	Ar-Censcu	1.2694	1.2912	0.9990	0.2703	0.2922
Ba-Delbac	Ar-Anogla	1.1308	0.9916	0.1392	Ba-Delbac	Ar-Aptruf	1.2401	1.4688	-0.2287	Ba-Delbac	Ar-Chisup	1.4152	1.1397	0.9901	0.4252	0.1497
Ba-Delbac	Ar-Aptruf	1.2191	1.5007	-0.2816	Ba-Delbac	Ar-Bomman	1.0283	1.0772	-0.0489	Ba-Delbac	Ar-Danmel	1.3628	1.1036	1.0637	0.2991	0.0399
Ba-Delbac	Ar-Aulsol	1.2556	1.1092	0.1464	Ba-Delbac	Ar-Censcu	1.2912	0.9990	0.2922	Ba-Delbac	Ar-Erilan	1.2475	1.2274	1.0495	0.1980	0.1779
Ba-Delbac	Ar-Bomman	1.4359	1.0675	0.3684	Ba-Delbac	Ar-Chisup	1.1397	0.9901	0.1497	Ba-Delbac	Ar-Eufmex	1.4189	1.1383	0.9714	0.4474	0.1669
Ba-Delbac	Ar-Calmac	1.3548	1.1110	0.2437	Ba-Delbac	Ar-Danmel	1.1036	1.0637	0.0399	Ba-Delbac	Ar-Helarm	1.4294	1.1773	1.0678	0.3616	0.1095
Ba-Delbac	Ar-Censcu	1.2694	1.0165	0.2528	Ba-Delbac	Ar-Erilan	1.2274	1.0495	0.1779	Ba-Delbac	Ar-Helzea	1.4179	1.2033	1.0773	0.3405	0.1260
Ba-Delbac	Ar-Chisup	1.4152	0.9992	0.4160	Ba-Delbac	Ar-Eufmex	1.1383	0.9714	0.1669	Ba-Delbac	Ar-Lepdec	1.1984	1.1862	1.1492	0.0492	0.0370
Ba-Delbac	Ar-Danmel	1.3628	1.0423	0.3204	Ba-Delbac	Ar-Eupann	1.0157	1.0179	-0.0022	Ba-Delbac	Ar-Lephet	1.3574	1.1910	0.9908	0.3665	0.2002
Ba-Delbac	Ar-Epacla	1.3317	1.0383	0.2934	Ba-Delbac	Ar-Galmel	1.1056	1.2027	-0.0971	Ba-Delbac	Ar-Limcal	1.4271	1.1560	1.0659	0.3612	0.0902
Ba-Delbac	Ar-Erilan	1.2475	1.0684	0.1791	Ba-Delbac	Ar-Glocon	1.1095	1.1537	-0.0442	Ba-Delbac	Ar-Manjur	1.4014	1.1002	0.9986	0.4028	0.1017
Ba-Delbac	Ar-Eufmex	1.4189	0.9607	0.4582	Ba-Delbac	Ar-Helarm	1.1773	1.0678	0.1095	Ba-Delbac	Ar-Neopin	1.1688	1.2080	1.0161	0.1528	0.1919
Ba-Delbac	Ar-Eupann	1.3802	1.0256	0.3546	Ba-Delbac	Ar-Helzea	1.2033	1.0773	0.1260	Ba-Delbac	Ar-Nillug	1.0969	1.3396	1.0967	0.0002	0.2429
Ba-Delbac	Ar-Eupuro	1.3842	1.0343	0.3500	Ba-Delbac	Ar-Ladful	1.0470	1.1190	-0.0720	Ba-Delbac	Ar-Opebru	1.3434	1.1455	1.0184	0.3249	0.1270
Ba-Delbac	Ar-Galmel	1.3949	1.1587	0.2362	Ba-Delbac	Ar-Lepdec	1.1862	1.1492	0.0370	Ba-Delbac	Ar-Osmbic	1.3558	1.1658	1.0521	0.3037	0.1137
Ba-Delbac	Ar-Glocon	1.2247	1.1456	0.0791	Ba-Delbac	Ar-Lephet	1.1910	0.9908	0.2002	Ba-Delbac	Ar-Partep	1.4044	1.3089	1.0811	0.3232	0.2278
Ba-Delbac	Ar-Helarm	1.4294	1.0758	0.3536	Ba-Delbac	Ar-Limcal	1.1560	1.0659	0.0902	Ba-Delbac	Ar-Pluxyl	1.3261	1.3479	0.9611	0.3650	0.3868
Ba-Delbac	Ar-Helzea	1.4179	1.0700	0.3478	Ba-Delbac	Ar-Lymdis	1.0661	1.1108	-0.0447	Ba-Delbac	Ar-Pseelo	1.3014	1.3033	1.0537	0.2477	0.2496
Ba-Delbac	Ar-Homvit	1.1789	1.0943	0.0846	Ba-Delbac	Ar-Manjur	1.1002	0.9986	0.1017	Ba-Delbac	Ar-Schgra	1.1604	1.2216	1.0843	0.0761	0.1373
Ba-Delbac	Ar-Ladful	1.2542	1.1070	0.1472	Ba-Delbac	Ar-Neopin	1.2080	1.0161	0.1919	Ba-Delbac	Ar-Sipfla	1.1928	1.2344	1.1389	0.0539	0.0954
Ba-Delbac	Ar-Lepdec	1.1984	1.1649	0.0335	Ba-Delbac	Ar-Nillug	1.3396	1.0967	0.2429	Ba-Delbac	Ar-Sogfur	1.4432	1.3577	1.1198	0.3234	0.2379
Ba-Delbac	Ar-Lephet	1.3574	0.9687	0.3886	Ba-Delbac	Ar-Onttau	1.1646	1.1710	-0.0064	Ba-Delbac	Ar-Stedum	1.3541	1.1649	1.0692	0.2849	0.0957
Ba-Delbac	Ar-Limcal	1.4271	1.0877	0.3394	Ba-Delbac	Ar-Opebru	1.1455	1.0184	0.1270	Ba-Delbac	Ar-Temlon	1.2620	1.1005	1.0498	0.2122	0.0507
Ba-Delbac	Ar-Locmig	1.2846	1.0014	0.2832	Ba-Delbac	Ar-Osmbic	1.1658	1.0521	0.1137	Ba-Delbac	Ar-Timbar	1.1714	1.1120	1.0958	0.0756	0.0162
Ba-Delbac	Ar-Manjur	1.4014	0.9921	0.4093	Ba-Delbac	Ar-Partep	1.3089	1.0811	0.2278	Ba-Delbac	Ar-Tutabs	1.2599	1.1196	1.0861	0.1738	0.0335
Ba-Delbac	Ar-Neopin	1.1688	1.0035	0.1653	Ba-Delbac	Ar-Pluxyl	1.3479	0.9611	0.3868	Ba-Delbac	Mo-Halrub	1.2243	1.2062	0.9584	0.2659	0.2478
Ba-Delbac	Ar-Nillug	1.0969	1.0823	0.0146	Ba-Delbac	Ar-Pseelo	1.3033	1.0537	0.2496	Ba-Delbac	Ne-Caenig	1.3115	1.3743	1.2080	0.1035	0.1663
Ba-Delbac	Ar-Onttau	1.4030	1.1839	0.2192	Ba-Delbac	Ar-Schgra	1.2216	1.0843	0.1373	Ba-Desbac	Ar-Adohon	1.3252	1.1117	1.0955	0.2297	0.0162
Ba-Delbac	Ar-Opebru	1.3434	1.0142	0.3292	Ba-Delbac	Ar-Sipfla	1.2344	1.1389	0.0954	Ba-Desbac	Ar-Ampamp	1.5256	1.2363	1.2332	0.2924	0.0031
Ba-Delbac	Ar-Osmbic	1.3558	1.0405	0.3154	Ba-Delbac	Ar-Sogfur	1.3577	1.1198	0.2379	Ba-Desbac	Ar-Bomman	1.1301	1.0369	1.0349	0.0951	0.0020
Ba-Delbac	Ar-Partep	1.4044	1.0426	0.3618	Ba-Delbac	Ar-Stedum	1.1649	1.0692	0.0957	Ba-Desbac	Ar-Chisup	1.1863	1.1595	0.9988	0.1875	0.1606
Ba-Delbac	Ar-Phesol	1.3844	1.0966	0.2878	Ba-Delbac	Ar-Temlon	1.1005	1.0498	0.0507	Ba-Desbac	Ar-Erilan	1.1279	1.1350	1.0078	0.1201	0.1273
Ba-Delbac	Ar-Pluxyl	1.3261	0.9815	0.3447	Ba-Delbac	Ar-Thrpal	1.1838	1.3082	-0.1245	Ba-Desbac	Ar-Lephet	1.0936	1.0349	1.0081	0.0855	0.0268
Ba-Delbac	Ar-Pseelo	1.3014	1.0335	0.2679	Ba-Delbac	Ar-Timbar	1.1120	1.0958	0.0162	Ba-Desbac	Ar-Nillug	1.2885	1.2121	1.1058	0.1827	0.1063
Ba-Delbac	Ar-Schgra	1.1604	1.0713	0.0891	Ba-Delbac	Ar-Timcri	0.9409	1.0827	-0.1418	Ba-Desbac	Ar-Opebru	1.3253	1.0270	1.0087	0.3166	0.0183
Ba-Delbac	Ar-Sipfla	1.1928	1.1696	0.0232	Ba-Delbac	Ar-Timgen	1.0264	1.0477	-0.0213	Ba-Desbac	Ar-Partep	1.1696	1.2154	1.0912	0.0784	0.1242
Ba-Delbac	Ar-Sitmis	1.2698	0.9800	0.2898	Ba-Delbac	Ar-Timmon	0.9976	1.0902	-0.0927	Ba-Desbac	Ar-Pluxyl	1.2598	1.3607	1.0615	0.1983	0.2992
Ba-Delbac	Ar-Sogfur	1.4432	1.1334	0.3098	Ba-Delbac	Ar-Timpop	0.9477	1.0878	-0.1400	Ba-Desbac	Ar-Sogfur	1.2131	1.3156	1.0730	0.1401	0.2425
Ba-Delbac	Ar-Stedum	1.3541	1.0463	0.3077	Ba-Delbac	Ar-Timshe	0.9711	1.0878	-0.1167	Ba-Desbac	Ar-Timbar	1.1757	1.2521	1.1430	0.0327	0.1090
Ba-Delbac	Ar-Stemim	1.3397	1.0566	0.2830	Ba-Delbac	Ar-Timtah	1.0209	1.0920	-0.0710	Ba-Desbac	Ar-Timgen	1.1869	1.1711	1.1132	0.0738	0.0580
Ba-Delbac	Ar-Temlon	1.2620	1.0455	0.2165	Ba-Delbac	Ar-Tutabs	1.1196	1.0861	0.0335	Ba-Desbac	Ar-Timtah	1.1757	1.1433	1.1390	0.0367	0.0044
Ba-Delbac	Ar-Thrpal	1.2952	1.2843	0.0109	Ba-Delbac	Ar-Vantam	1.1236	1.1522	-0.0286	Ba-Desbac	Mo-Halrub	1.4508	1.1506	1.0143	0.4365	0.1363
Ba-Delbac	Ar-Timbar	1.1714	1.0749	0.0965	Ba-Delbac	Fu-Lobtra	1.2709	1.3499	-0.0790	Ba-Legsp.	Ar-Acypis	1.1243	1.1842	1.0957	0.0286	0.0885
Ba-Delbac	Ar-Timcri	1.1224	1.0774	0.0450	Ba-Delbac	Fu-Morsp.	1.2086	1.3438	-0.1353	Ba-Legsp.	Ar-Ampamp	1.4485	1.2330	1.1925	0.2560	0.0405
Ba-Delbac	Ar-Timgen	1.1499	1.0373	0.1126	Ba-Delbac	Mo-Halrub	1.2062	0.9584	0.2478	Ba-Legsp.	Ar-Bomman	1.0552	1.0876	1.0136	0.0416	0.0740
Ba-Delbac	Ar-Timmon	1.1278	1.0850	0.0428	Ba-Delbac	Mo-Mermer	1.3566	0.9875	0.3691	Ba-Legsp.	Ar-Censcu	1.2657	1.2626	1.0538	0.2119	0.2088
Ba-Delbac	Ar-Timpop	1.1353	1.0826	0.0527	Ba-Delbac	Ne-Caeele	1.1913	1.2080	-0.0167	Ba-Legsp.	Ar-Chisup	1.1128	1.1525	1.0946	0.0181	0.0578
Ba-Delbac	Ar-Timshe	1.1666	1.0826	0.0840	Ba-Delbac	Ne-Caenig	1.3743	1.2080	0.1663	Ba-Legsp.	Ar-Ladful	1.1480	1.1127	1.1103	0.0377	0.0024
Ba-Delbac	Ar-Timtah	1.1714	1.0712	0.1003	Ba-Delbac	St-Aphast	1.2705	1.1811	0.0894	Ba-Legsp.	Ar-Limcal	1.2080	1.1078	1.0938	0.1142	0.0139
Ba-Delbac	Ar-Trivap	1.3812	1.0634	0.3178	Ba-Delbac	St-Aphinv	1.1483	1.1367	0.0115	Ba-Legsp.	Ar-Manjur	1.1483	1.1077	1.0783	0.0700	0.0294
Ba-Delbac	Ar-Tutabs	1.2599	1.0424	0.2175	Ba-Delbac	St-Aphste	1.2536	1.2355	0.0180	Ba-Legsp.	Ar-Neopin	1.1456	1.0454	1.0301	0.1155	0.0153
Ba-Delbac	Ar-Vantam	1.4403	1.1426	0.2977	Ba-Delbac	St-Phycam	1.2710	1.2711	-0.0001	Ba-Legsp.	Ar-Nillug	1.1521	1.2113	0.9633	0.1888	0.2480

Ba-Delbac	Fu-Lobtra	0.9038	1.4151	-0.5113
Ba-Delbac	Fu-Morsp.	0.8796	1.2876	-0.4080
Ba-Delbac	Mo-Halrub	1.2243	0.9469	0.2774
Ba-Delbac	Mo-Mermer	0.8299	0.9536	-0.1237
Ba-Delbac	Ne-Caeele	1.4304	1.1981	0.2323
Ba-Delbac	Ne-Caenig	1.3115	1.1981	0.1134
Ba-Delbac	St-Aphast	0.8319	1.1927	-0.3608
Ba-Delbac	St-Aphinv	0.8967	1.1271	-0.2304
Ba-Delbac	St-Aphste	0.7470	1.2115	-0.4645
Ba-Delbac	St-Phycam	0.7759	1.2189	-0.4430
Ba-Delbac	St-Physyr	0.8210	1.2034	-0.3824
Ba-Delbac	St-Phytub	0.7923	1.3390	-0.5467
Ba-Delbac	St-Pilapi	0.9714	1.1796	-0.2082
Ba-Delbac	St-Pytoli	0.7991	1.1461	-0.3470
Ba-Desbac	Ar-Acypis	1.2382	1.2008	0.0375
Ba-Desbac	Ar-Adohon	1.3252	1.1195	0.2057
Ba-Desbac	Ar-Ampamp	1.5256	1.2462	0.2794
Ba-Desbac	Ar-Amytra	1.1055	1.0528	0.0527
Ba-Desbac	Ar-Anogla	1.2327	1.0496	0.1831
Ba-Desbac	Ar-Aptruf	1.0520	1.4063	-0.3543
Ba-Desbac	Ar-Aulsol	1.2945	1.1282	0.1663
Ba-Desbac	Ar-Bomman	1.1301	1.0125	0.1176
Ba-Desbac	Ar-Calmac	1.3506	1.1838	0.1669
Ba-Desbac	Ar-Censcu	0.9864	1.0626	-0.0762
Ba-Desbac	Ar-Chisup	1.1863	0.9892	0.1971
Ba-Desbac	Ar-Danmel	1.1829	1.0876	0.0953
Ba-Desbac	Ar-Epacla	1.0789	1.0664	0.0124
Ba-Desbac	Ar-Erilan	1.1279	1.0170	0.1109
Ba-Desbac	Ar-Eufmex	1.1176	1.0797	0.0379
Ba-Desbac	Ar-Eupann	1.2065	1.1058	0.1007
Ba-Desbac	Ar-Eupuro	1.2232	1.1328	0.0904
Ba-Desbac	Ar-Galmel	1.0994	1.1761	-0.0767
Ba-Desbac	Ar-Glocon	1.2076	1.1418	0.0658
Ba-Desbac	Ar-Helarm	1.1918	1.1526	0.0392
Ba-Desbac	Ar-Helzea	1.1688	1.1514	0.0174
Ba-Desbac	Ar-Homvit	1.3040	1.1293	0.1747
Ba-Desbac	Ar-Ladful	1.2078	1.1474	0.0604
Ba-Desbac	Ar-Lepdec	1.2399	1.1932	0.0467
Ba-Desbac	Ar-Lephet	1.0936	0.9919	0.1017
Ba-Desbac	Ar-Limcal	1.1977	1.0955	0.1022
Ba-Desbac	Ar-Locmig	1.0726	1.0575	0.0151
Ba-Desbac	Ar-Manjur	1.2728	1.0713	0.2015
Ba-Desbac	Ar-Neopin	1.1594	1.0317	0.1277
Ba-Desbac	Ar-Nillug	1.2885	1.0769	0.2117
Ba-Desbac	Ar-Onttau	1.1843	1.1923	-0.0081
Ba-Desbac	Ar-Opebru	1.3253	1.0040	0.3213
Ba-Desbac	Ar-Osmbic	1.1329	1.0778	0.0550
Ba-Desbac	Ar-Partep	1.1696	1.0597	0.1099
Ba-Desbac	Ar-Phesol	1.1289	1.0938	0.0351
Ba-Desbac	Ar-Pluxyl	1.2598	1.0918	0.1679
Ba-Desbac	Ar-Pseelo	1.0898	1.0500	0.0398
Ba-Desbac	Ar-Schgra	1.2979	1.1337	0.1642
Ba-Desbac	Ar-Sipfla	1.1730	1.2112	-0.0383
Ba-Desbac	Ar-Sitmis	1.2394	1.0129	0.2265
Ba-Desbac	Ar-Sogfur	1.2131	1.0890	0.1240
Ba-Desbac	Ar-Stedum	1.0672	1.0623	0.0049
Ba-Desbac	Ar-Stemim	1.0547	1.1328	-0.0782
Ba-Desbac	Ar-Temlon	1.2556	1.0379	0.2177
Ba-Desbac	Ar-Thrpal	1.0959	1.2794	-0.1835

Ba-Delbac	St-Physyr	1.2843	1.2038	0.0805
Ba-Delbac	St-Phytub	1.2783	1.3382	-0.0599
Ba-Delbac	St-Pilapi	1.1015	1.1761	-0.0746
Ba-Delbac	St-Pytoli	1.2159	1.1700	0.0459
Ba-Desbac	Ar-Acypis	1.1035	1.1855	-0.0820
Ba-Desbac	Ar-Adohon	1.1117	1.0955	0.0162
Ba-Desbac	Ar-Ampamp	1.2363	1.2332	0.0031
Ba-Desbac	Ar-Amytra	1.0882	1.0925	-0.0043
Ba-Desbac	Ar-Aptruf	1.1941	1.3743	-0.1802
Ba-Desbac	Ar-Bomman	1.0369	1.0349	0.0020
Ba-Desbac	Ar-Censcu	1.1456	1.0677	0.0779
Ba-Desbac	Ar-Chisup	1.1595	0.9988	0.1606
Ba-Desbac	Ar-Danmel	1.0234	1.0951	-0.0717
Ba-Desbac	Ar-Erilan	1.1350	1.0078	0.1273
Ba-Desbac	Ar-Eufmex	0.9981	1.0917	-0.0936
Ba-Desbac	Ar-Eupann	0.9886	1.0601	-0.0715
Ba-Desbac	Ar-Galmel	1.0118	1.1931	-0.1813
Ba-Desbac	Ar-Glocon	1.0924	1.1679	-0.0755
Ba-Desbac	Ar-Helarm	1.0796	1.1517	-0.0720
Ba-Desbac	Ar-Helzea	1.1182	1.1658	-0.0475
Ba-Desbac	Ar-Ladful	1.0530	1.1446	-0.0917
Ba-Desbac	Ar-Lepdec	1.1080	1.1676	-0.0596
Ba-Desbac	Ar-Lephet	1.0349	1.0081	0.0268
Ba-Desbac	Ar-Limcal	1.0169	1.0778	-0.0609
Ba-Desbac	Ar-Lymdis	1.0336	1.1161	-0.0826
Ba-Desbac	Ar-Manjur	1.0205	1.0604	-0.0399
Ba-Desbac	Ar-Neopin	1.0192	1.0385	-0.0193
Ba-Desbac	Ar-Nillug	1.2121	1.1058	0.1063
Ba-Desbac	Ar-Onttau	1.0329	1.2010	-0.1681
Ba-Desbac	Ar-Opebru	1.0270	1.0087	0.0183
Ba-Desbac	Ar-Osmbic	1.0252	1.0966	-0.0714
Ba-Desbac	Ar-Partep	1.2154	1.0912	0.1242
Ba-Desbac	Ar-Pluxyl	1.3607	1.0615	0.2992
Ba-Desbac	Ar-Pseelo	1.0665	1.0761	-0.0096
Ba-Desbac	Ar-Schgra	1.1142	1.1269	-0.0127
Ba-Desbac	Ar-Sipfla	1.1088	1.1963	-0.0875
Ba-Desbac	Ar-Sogfur	1.3156	1.0730	0.2425
Ba-Desbac	Ar-Stedum	1.1925	1.0834	0.1091
Ba-Desbac	Ar-Temlon	0.9945	1.0397	-0.0452
Ba-Desbac	Ar-Thrpal	1.1656	1.2734	-0.1078
Ba-Desbac	Ar-Timbar	1.2521	1.1430	0.1090
Ba-Desbac	Ar-Timcri	1.0873	1.1325	-0.0451
Ba-Desbac	Ar-Timgen	1.1711	1.1132	0.0580
Ba-Desbac	Ar-Timmon	1.1221	1.1405	-0.0185
Ba-Desbac	Ar-Timpop	1.0610	1.1380	-0.0770
Ba-Desbac	Ar-Timshe	1.0822	1.1427	-0.0606
Ba-Desbac	Ar-Timtah	1.1433	1.1390	0.0044
Ba-Desbac	Ar-Tutabs	1.0786	1.1730	-0.0944
Ba-Desbac	Ar-Vantam	0.9829	1.1994	-0.2166
Ba-Desbac	Fu-Lobtra	1.0517	1.3523	-0.3006
Ba-Desbac	Fu-Morsp.	1.0498	1.2731	-0.2233
Ba-Desbac	Mo-Halrub	1.1506	1.0143	0.1363
Ba-Desbac	Mo-Mermer	1.0259	0.9757	0.0502
Ba-Desbac	Ne-Caeele	1.0934	1.2047	-0.1113
Ba-Desbac	Ne-Caenig	1.1829	1.2047	-0.0217
Ba-Desbac	St-Aphast	1.0277	1.1636	-0.1359
Ba-Desbac	St-Aphinv	1.0481	1.1368	-0.0887
Ba-Desbac	St-Aphste	1.1172	1.2818	-0.1645
Ba-Desbac	St-Phycam	1.1251	1.1942	-0.0691

Ba-Legsp.	Ar-Onttau	1.1926	1.0844	1.0587	0.1339	0.0257
Ba-Legsp.	Ar-Opebru	1.1897	1.0487	1.0199	0.1698	0.0287
Ba-Legsp.	Ar-Pluxyl	1.1379	1.4069	1.0344	0.1034	0.3725
Ba-Legsp.	Ar-Pseelo	1.2041	1.2605	1.0495	0.1546	0.2109
Ba-Legsp.	Ar-Schgra	1.1399	1.1526	1.0199	0.1200	0.1327
Ba-Legsp.	Ar-Temlon	1.1577	1.1396	1.1112	0.0466	0.0285
Ba-Legsp.	Ar-Timbar	1.1252	1.1020	1.0738	0.0514	0.0281
Ba-Legsp.	Ar-Timcri	1.0959	1.0740	1.0712	0.0247	0.0028
Ba-Legsp.	Ar-Timmon	1.0900	1.0864	1.0712	0.0188	0.0152
Ba-Legsp.	Ar-Timtah	1.1252	1.0836	1.0738	0.0514	0.0098
Ba-Legsp.	Ar-Tutabs	1.2351	1.0678	1.0520	0.1830	0.0157
Ba-Legsp.	Mo-Halrub	1.2245	1.2723	0.8795	0.3451	0.3928
Ba-uncDes	Ar-Acypis	1.2739	1.2018	1.0874	0.1865	0.1144
Ba-uncDes	Ar-Adohon	1.1671	1.1085	1.0961	0.0710	0.0124
Ba-uncDes	Ar-Amytra	1.2886	1.1548	1.0980	0.1906	0.0567
Ba-uncDes	Ar-Bomman	1.3108	1.0829	1.0666	0.2443	0.0163
Ba-uncDes	Ar-Chisup	1.2674	1.1857	1.0301	0.2373	0.1556
Ba-uncDes	Ar-Erilan	1.2856	1.1538	1.0868	0.1988	0.0669
Ba-uncDes	Ar-Eufmex	1.1782	1.1969	0.9283	0.2499	0.2685
Ba-uncDes	Ar-Galmel	1.2528	1.0805	1.0582	0.1946	0.0223
Ba-uncDes	Ar-Glocon	1.2189	1.0674	1.0647	0.1542	0.0027
Ba-uncDes	Ar-Helarm	1.2467	1.1071	1.0547	0.1920	0.0524
Ba-uncDes	Ar-Helzea	1.2499	1.0926	1.0541	0.1959	0.0386
Ba-uncDes	Ar-Ladful	1.2641	1.1178	1.0708	0.1933	0.0470
Ba-uncDes	Ar-Lephet	1.1967	1.1594	1.0766	0.1201	0.0828
Ba-uncDes	Ar-Limcal	1.2653	1.1852	1.0356	0.2297	0.1496
Ba-uncDes	Ar-Manjur	1.2687	1.0804	1.0688	0.1999	0.0115
Ba-uncDes	Ar-Neopin	1.1432	1.0386	0.9891	0.1540	0.0495
Ba-uncDes	Ar-Nillug	1.0912	1.1010	0.9974	0.0938	0.1036
Ba-uncDes	Ar-Onttau	1.2440	1.0976	1.0214	0.2227	0.0763
Ba-uncDes	Ar-Partep	1.1814	1.1635	1.0599	0.1216	0.1037
Ba-uncDes	Ar-Pluxyl	1.3419	1.0769	0.9920	0.3499	0.0850
Ba-uncDes	Ar-Pseelo	1.2382	1.2166	1.0227	0.2155	0.1939
Ba-uncDes	Ar-Schgra	1.2106	1.2452	0.9991	0.2115	0.2461
Ba-uncDes	Ar-Sipfla	1.3212	1.1995	1.0750	0.2462	0.1244
Ba-uncDes	Ar-Sogfur	1.1860	1.2005	0.9572	0.2288	0.2433
Ba-uncDes	Ar-Stedum	1.1881	1.2383	0.9739	0.2142	0.2645
Ba-uncDes	Ar-Temlon	1.1753	1.1515	1.0649	0.1104	0.0866
Ba-uncDes	Ar-Timbar	1.1172	1.1527	1.0456	0.0716	0.1071
Ba-uncDes	Ar-Timcri	1.1065	1.1194	1.0456	0.0608	0.0738
Ba-uncDes	Ar-Timgen	1.1288	1.1271	1.0327	0.0962	0.0945
Ba-uncDes	Ar-Timmon	1.1065	1.1493	1.0456	0.0608	0.1037
Ba-uncDes	Ar-Timpop	1.1222	1.0475	1.0463	0.0759	0.0012
Ba-uncDes	Ar-Timshe	1.1137	1.1132	1.0450	0.0687	0.0682
Ba-uncDes	Ar-Timtah	1.1172	1.1593	1.0463	0.0709	0.1131
Ba-uncDes	Ar-Tutabs	1.1800	1.1214	1.0120	0.1680	0.1095
Ba-uncDes	Ar-Vantam	1.2650	1.1447	1.0537	0.2112	0.0909
Ba-uncDes	Mo-Halrub	1.4243	1.1065	0.9526	0.4717	0.1539

Ba-Desbac	Ar-Timbar	1.1757	1.1386	0.0371	Ba-Desbac	St-Physyr	1.0707	1.2585	-0.1878
Ba-Desbac	Ar-Timcri	1.1771	1.1412	0.0359	Ba-Desbac	St-Phytub	1.0684	1.3164	-0.2479
Ba-Desbac	Ar-Timgen	1.1869	1.1062	0.0807	Ba-Desbac	St-Pilapi	1.0190	1.1627	-0.1436
Ba-Desbac	Ar-Timmon	1.1838	1.1494	0.0344	Ba-Desbac	St-Pytoli	1.1532	1.1044	0.0488
Ba-Desbac	Ar-Timpop	1.1463	1.1468	-0.0005	Ba-Legsp.	Ar-Acypis	1.1842	1.0957	0.0885
Ba-Desbac	Ar-Timshe	1.1587	1.1517	0.0070	Ba-Legsp.	Ar-Adohon	1.1935	1.0983	0.0952
Ba-Desbac	Ar-Timtah	1.1757	1.1345	0.0412	Ba-Legsp.	Ar-Ampamp	1.2330	1.1925	0.0405
Ba-Desbac	Ar-Trivap	1.1445	1.1370	0.0076	Ba-Legsp.	Ar-Amytra	1.0272	1.0558	-0.0286
Ba-Desbac	Ar-Tutabs	1.2956	1.1488	0.1467	Ba-Legsp.	Ar-Aptruf	1.1491	1.2664	-0.1173
Ba-Desbac	Ar-Vantam	1.1966	1.2280	-0.0314	Ba-Legsp.	Ar-Bomman	1.0876	1.0136	0.0740
Ba-Desbac	Fu-Lobtra	0.9511	1.4335	-0.4824	Ba-Legsp.	Ar-Censcu	1.2626	1.0538	0.2088
Ba-Desbac	Fu-Morsp.	0.9753	1.1690	-0.1937	Ba-Legsp.	Ar-Chisup	1.1525	1.0946	0.0578
Ba-Desbac	Mo-Halrub	1.4508	0.9978	0.4531	Ba-Legsp.	Ar-Danmel	1.0267	1.0738	-0.0471
Ba-Desbac	Mo-Mermer	0.9695	0.9586	0.0109	Ba-Legsp.	Ar-Erilan	1.0892	1.0898	-0.0006
Ba-Desbac	Ne-Caeele	1.2166	1.2110	0.0056	Ba-Legsp.	Ar-Eufmex	1.0434	1.1008	-0.0574
Ba-Desbac	Ne-Caenig	1.1523	1.2110	-0.0587	Ba-Legsp.	Ar-Eupann	1.0823	1.1227	-0.0405
Ba-Desbac	St-Aphast	1.0329	1.2062	-0.1733	Ba-Legsp.	Ar-Galmel	1.0535	1.0553	-0.0018
Ba-Desbac	St-Aphinv	1.0075	1.1077	-0.1002	Ba-Legsp.	Ar-Glocon	1.2696	1.1507	0.1190
Ba-Desbac	St-Aphste	1.0668	1.2555	-0.1887	Ba-Legsp.	Ar-Helarm	1.0350	1.1224	-0.0874
Ba-Desbac	St-Phycam	1.0701	1.2027	-0.1326	Ba-Legsp.	Ar-Helzea	1.0873	1.1172	-0.0300
Ba-Desbac	St-Physyr	1.0423	1.2792	-0.2369	Ba-Legsp.	Ar-Ladful	1.1127	1.1103	0.0024
Ba-Desbac	St-Phytub	1.0406	1.2823	-0.2416	Ba-Legsp.	Ar-Lepdec	1.1231	1.2074	-0.0843
Ba-Desbac	St-Pilapi	0.9826	1.1402	-0.1576	Ba-Legsp.	Ar-Lephet	1.0952	1.0738	0.0215
Ba-Desbac	St-Pytoli	1.0956	1.1043	-0.0088	Ba-Legsp.	Ar-Limcal	1.1078	1.0938	0.0139
Ba-Legsp.	Ar-Acypis	1.1243	1.0976	0.0267	Ba-Legsp.	Ar-Lymdis	0.9573	1.0834	-0.1261
Ba-Legsp.	Ar-Adohon	1.0715	1.0836	-0.0121	Ba-Legsp.	Ar-Manjur	1.1077	1.0783	0.0294
Ba-Legsp.	Ar-Ampamp	1.4485	1.1770	0.2715	Ba-Legsp.	Ar-Neopin	1.0454	1.0301	0.0153
Ba-Legsp.	Ar-Amytra	1.0683	1.0367	0.0316	Ba-Legsp.	Ar-Nillug	1.2113	0.9633	0.2480
Ba-Legsp.	Ar-Anogla	1.2331	1.0792	0.1539	Ba-Legsp.	Ar-Onttau	1.0844	1.0587	0.0257
Ba-Legsp.	Ar-Aptruf	1.2016	1.3115	-0.1099	Ba-Legsp.	Ar-Opebru	1.0487	1.0199	0.0287
Ba-Legsp.	Ar-Aulsol	1.1162	1.0373	0.0789	Ba-Legsp.	Ar-Osmbic	1.1034	1.1355	-0.0321
Ba-Legsp.	Ar-Bomman	1.0552	0.9763	0.0789	Ba-Legsp.	Ar-Partep	1.1047	1.1492	-0.0445
Ba-Legsp.	Ar-Calmac	1.2260	1.1957	0.0303	Ba-Legsp.	Ar-Pluxyl	1.4069	1.0344	0.3725
Ba-Legsp.	Ar-Censcu	1.2657	1.0364	0.2293	Ba-Legsp.	Ar-Pseelo	1.2605	1.0495	0.2109
Ba-Legsp.	Ar-Chisup	1.1128	1.0558	0.0570	Ba-Legsp.	Ar-Schgra	1.1526	1.0199	0.1327
Ba-Legsp.	Ar-Danmel	1.0249	1.0552	-0.0303	Ba-Legsp.	Ar-Sipfla	1.1275	1.1296	-0.0022
Ba-Legsp.	Ar-Epacla	1.0619	1.0304	0.0315	Ba-Legsp.	Ar-Sogfur	1.4113	1.1511	0.2602
Ba-Legsp.	Ar-Erilan	1.0134	1.0653	-0.0519	Ba-Legsp.	Ar-Stedum	1.1318	1.1509	-0.0191
Ba-Legsp.	Ar-Eufmex	1.1486	1.0711	0.0775	Ba-Legsp.	Ar-Temlon	1.1396	1.1112	0.0285
Ba-Legsp.	Ar-Eupann	1.1273	1.1482	-0.0209	Ba-Legsp.	Ar-Thrpal	1.0680	1.2386	-0.1706
Ba-Legsp.	Ar-Eupuro	1.1099	1.1194	-0.0096	Ba-Legsp.	Ar-Timbar	1.1020	1.0738	0.0281
Ba-Legsp.	Ar-Galmel	1.0477	1.0332	0.0144	Ba-Legsp.	Ar-Timcri	1.0740	1.0712	0.0028
Ba-Legsp.	Ar-Glocon	1.1233	1.1383	-0.0149	Ba-Legsp.	Ar-Timgen	1.0517	1.0803	-0.0286
Ba-Legsp.	Ar-Helarm	1.0549	1.0892	-0.0343	Ba-Legsp.	Ar-Timmon	1.0864	1.0712	0.0152
Ba-Legsp.	Ar-Helzea	1.0356	1.0841	-0.0485	Ba-Legsp.	Ar-Timpop	0.9984	1.0705	-0.0721
Ba-Legsp.	Ar-Homvit	1.1608	1.1314	0.0294	Ba-Legsp.	Ar-Timshe	1.0510	1.0746	-0.0236
Ba-Legsp.	Ar-Ladful	1.1480	1.0756	0.0724	Ba-Legsp.	Ar-Timtah	1.0836	1.0738	0.0098
Ba-Legsp.	Ar-Lepdec	1.1043	1.2116	-0.1073	Ba-Legsp.	Ar-Tutabs	1.0678	1.0520	0.0157
Ba-Legsp.	Ar-Lephet	0.9818	1.0524	-0.0706	Ba-Legsp.	Ar-Vantam	1.0849	1.1090	-0.0241
Ba-Legsp.	Ar-Limcal	1.2080	1.0675	0.1405	Ba-Legsp.	Fu-Lobtra	1.1012	1.2513	-0.1501
Ba-Legsp.	Ar-Locmig	1.1223	1.0264	0.0959	Ba-Legsp.	Fu-Morsp.	1.1287	1.2282	-0.0995
Ba-Legsp.	Ar-Manjur	1.1483	1.0611	0.0872	Ba-Legsp.	Mo-Halrub	1.2723	0.8795	0.3928
Ba-Legsp.	Ar-Neopin	1.1456	0.9976	0.1479	Ba-Legsp.	Mo-Mermer	1.1297	0.9638	0.1659
Ba-Legsp.	Ar-Nillug	1.1521	0.9220	0.2300	Ba-Legsp.	Ne-Caeele	1.3198	1.3417	-0.0219
Ba-Legsp.	Ar-Onttau	1.1926	1.0484	0.1441	Ba-Legsp.	Ne-Caenig	1.3557	1.3417	0.0140
Ba-Legsp.	Ar-Opebru	1.1897	1.0199	0.1698	Ba-Legsp.	St-Aphast	1.1929	1.1353	0.0576
Ba-Legsp.	Ar-Osmbic	1.1084	1.1105	-0.0021	Ba-Legsp.	St-Aphinv	1.1653	1.0528	0.1125
Ba-Legsp.	Ar-Partep	1.0815	1.1291	-0.0477	Ba-Legsp.	St-Aphste	1.3059	1.2431	0.0628
Ba-Legsp.	Ar-Phesol	1.0852	1.0342	0.0510	Ba-Legsp.	St-Phycam	1.2540	1.2290	0.0251

Ba-Legsp.	Ar-Pluxyl	1.1379	1.0170	0.1209	Ba-Legsp.	St-Physyr	1.3119	1.2270	0.0849
Ba-Legsp.	Ar-Pseelo	1.2041	1.0150	0.1891	Ba-Legsp.	St-Phytub	1.2720	1.3138	-0.0418
Ba-Legsp.	Ar-Schgra	1.1399	1.0209	0.1190	Ba-Legsp.	St-Pilapi	1.3759	1.3135	0.0624
Ba-Legsp.	Ar-Sipfla	1.0461	1.1242	-0.0781	Ba-Legsp.	St-Pytoli	1.4177	1.2758	0.1419
Ba-Legsp.	Ar-Sitmis	1.1060	1.0214	0.0846	Ba-uncDes	Ar-Acypis	1.2018	1.0874	0.1144
Ba-Legsp.	Ar-Sogfur	1.1966	1.1257	0.0709	Ba-uncDes	Ar-Adohon	1.1085	1.0961	0.0124
Ba-Legsp.	Ar-Stedum	1.0635	1.1096	-0.0462	Ba-uncDes	Ar-Ampamp	0.9746	1.0818	-0.1072
Ba-Legsp.	Ar-Stemim	1.0425	1.0528	-0.0103	Ba-uncDes	Ar-Amytra	1.1548	1.0980	0.0567
Ba-Legsp.	Ar-Temlon	1.1577	1.0937	0.0641	Ba-uncDes	Ar-Aptruf	1.1851	1.2350	-0.0499
Ba-Legsp.	Ar-Thrpal	1.1709	1.2200	-0.0491	Ba-uncDes	Ar-Bomman	1.0829	1.0666	0.0163
Ba-Legsp.	Ar-Timbar	1.1252	1.0664	0.0587	Ba-uncDes	Ar-Censcu	0.9853	1.1574	-0.1721
Ba-Legsp.	Ar-Timcri	1.0959	1.0638	0.0321	Ba-uncDes	Ar-Chisup	1.1857	1.0301	0.1556
Ba-Legsp.	Ar-Timgen	1.1119	1.0730	0.0389	Ba-uncDes	Ar-Danmel	1.0629	1.0668	-0.0039
Ba-Legsp.	Ar-Timmon	1.0900	1.0638	0.0262	Ba-uncDes	Ar-Erilan	1.1538	1.0868	0.0669
Ba-Legsp.	Ar-Timpop	1.1092	1.0631	0.0461	Ba-uncDes	Ar-Eufmex	1.1969	0.9283	0.2685
Ba-Legsp.	Ar-Timshe	1.1193	1.0672	0.0521	Ba-uncDes	Ar-Eupann	1.0867	1.0898	-0.0032
Ba-Legsp.	Ar-Timtah	1.1252	1.0664	0.0587	Ba-uncDes	Ar-Galmel	1.0805	1.0582	0.0223
Ba-Legsp.	Ar-Trivap	1.1610	1.1524	0.0086	Ba-uncDes	Ar-Glocon	1.0674	1.0647	0.0027
Ba-Legsp.	Ar-Tutabs	1.2351	1.0300	0.2051	Ba-uncDes	Ar-Helarm	1.1071	1.0547	0.0524
Ba-Legsp.	Ar-Vantam	1.0438	1.0797	-0.0359	Ba-uncDes	Ar-Helzea	1.0926	1.0541	0.0386
Ba-Legsp.	Fu-Lobtra	0.9793	1.3484	-0.3691	Ba-uncDes	Ar-Ladful	1.1178	1.0708	0.0470
Ba-Legsp.	Fu-Morsp.	0.8587	1.2299	-0.3712	Ba-uncDes	Ar-Lepdec	1.0355	1.2380	-0.2025
Ba-Legsp.	Mo-Halrub	1.2245	0.8907	0.3339	Ba-uncDes	Ar-Lephet	1.1594	1.0766	0.0828
Ba-Legsp.	Mo-Mermer	0.7947	0.9557	-0.1610	Ba-uncDes	Ar-Limcal	1.1852	1.0356	0.1496
Ba-Legsp.	Ne-Caeele	0.9971	1.3074	-0.3103	Ba-uncDes	Ar-Lymdis	1.1020	1.0328	0.0692
Ba-Legsp.	Ne-Caenig	1.1009	1.3074	-0.2065	Ba-uncDes	Ar-Manjur	1.0804	1.0688	0.0115
Ba-Legsp.	St-Aphast	0.9190	1.1628	-0.2438	Ba-uncDes	Ar-Neopin	1.0386	0.9891	0.0495
Ba-Legsp.	St-Aphinv	0.9129	1.0620	-0.1491	Ba-uncDes	Ar-Nillug	1.1010	0.9974	0.1036
Ba-Legsp.	St-Aphste	0.9645	1.2784	-0.3139	Ba-uncDes	Ar-Onttau	1.0976	1.0214	0.0763
Ba-Legsp.	St-Phycam	1.0029	1.1467	-0.1439	Ba-uncDes	Ar-Opebru	1.0086	1.0422	-0.0336
Ba-Legsp.	St-Physyr	0.9654	1.2503	-0.2849	Ba-uncDes	Ar-Osmbic	1.2256	1.1876	0.0381
Ba-Legsp.	St-Phytub	0.9386	1.3302	-0.3916	Ba-uncDes	Ar-Partep	1.1635	1.0599	0.1037
Ba-Legsp.	St-Pilapi	0.9422	1.2479	-0.3058	Ba-uncDes	Ar-Pluxyl	1.0769	0.9920	0.0850
Ba-Legsp.	St-Pytoli	0.9304	1.2375	-0.3071	Ba-uncDes	Ar-Pseelo	1.2166	1.0227	0.1939
Ba-uncDes	Ar-Acypis	1.2739	1.0808	0.1931	Ba-uncDes	Ar-Schgra	1.2452	0.9991	0.2461
Ba-uncDes	Ar-Adohon	1.1671	1.0852	0.0819	Ba-uncDes	Ar-Sipfla	1.1995	1.0750	0.1244
Ba-uncDes	Ar-Ampamp	1.2840	1.0853	0.1988	Ba-uncDes	Ar-Sogfur	1.2005	0.9572	0.2433
Ba-uncDes	Ar-Amytra	1.2886	1.0699	0.2188	Ba-uncDes	Ar-Stedum	1.2383	0.9739	0.2645
Ba-uncDes	Ar-Anogla	1.2108	1.0927	0.1181	Ba-uncDes	Ar-Temlon	1.1515	1.0649	0.0866
Ba-uncDes	Ar-Aptruf	1.2252	1.2124	0.0128	Ba-uncDes	Ar-Thrpal	1.1456	1.1795	-0.0339
Ba-uncDes	Ar-Aulsol	1.2921	1.0049	0.2872	Ba-uncDes	Ar-Timbar	1.1527	1.0456	0.1071
Ba-uncDes	Ar-Bomman	1.3108	1.0319	0.2790	Ba-uncDes	Ar-Timcri	1.1194	1.0456	0.0738
Ba-uncDes	Ar-Calmac	1.4358	1.1414	0.2944	Ba-uncDes	Ar-Timgen	1.1271	1.0327	0.0945
Ba-uncDes	Ar-Censcu	1.1728	1.1482	0.0246	Ba-uncDes	Ar-Timmon	1.1493	1.0456	0.1037
Ba-uncDes	Ar-Chisup	1.2674	0.9965	0.2709	Ba-uncDes	Ar-Timpop	1.0475	1.0463	0.0012
Ba-uncDes	Ar-Danmel	1.2427	1.0416	0.2011	Ba-uncDes	Ar-Timshe	1.1132	1.0450	0.0682
Ba-uncDes	Ar-Epacla	1.3410	1.0257	0.3153	Ba-uncDes	Ar-Timtah	1.1593	1.0463	0.1131
Ba-uncDes	Ar-Erilan	1.2856	1.0828	0.2028	Ba-uncDes	Ar-Tutabs	1.1214	1.0120	0.1095
Ba-uncDes	Ar-Eufmex	1.1782	0.9225	0.2557	Ba-uncDes	Ar-Vantam	1.1447	1.0537	0.0909
Ba-uncDes	Ar-Eupann	1.2231	1.1347	0.0885	Ba-uncDes	Fu-Lobtra	1.1548	1.1784	-0.0236
Ba-uncDes	Ar-Eupuro	1.2052	1.1567	0.0485	Ba-uncDes	Fu-Morsp.	1.0882	1.2592	-0.1710
Ba-uncDes	Ar-Galmel	1.2528	1.0221	0.2307	Ba-uncDes	Mo-Halrub	1.1065	0.9526	0.1539
Ba-uncDes	Ar-Glocon	1.2189	1.0252	0.1937	Ba-uncDes	Mo-Mermer	1.1870	1.0046	0.1824
Ba-uncDes	Ar-Helarm	1.2467	1.0003	0.2464	Ba-uncDes	Ne-Caeele	1.1374	1.2358	-0.0983
Ba-uncDes	Ar-Helzea	1.2499	0.9997	0.2502	Ba-uncDes	Ne-Caenig	1.0669	1.2358	-0.1689
Ba-uncDes	Ar-Homvit	1.1651	1.0924	0.0727	Ba-uncDes	St-Aphast	1.0099	1.1557	-0.1457
Ba-uncDes	Ar-Ladful	1.2641	1.0404	0.2237	Ba-uncDes	St-Aphinv	1.0524	1.1581	-0.1057
Ba-uncDes	Ar-Lepdec	1.2010	1.2326	-0.0316	Ba-uncDes	St-Aphste	0.9522	1.0336	-0.0814
Ba-uncDes	Ar-Lephet	1.1967	1.0520	0.1446	Ba-uncDes	St-Phycam	0.9870	1.2087	-0.2216

Ba-uncDes	Ar-Limcal	1.2653	1.0425	0.2228	Ba-uncDes	St-Physyr	0.9233	1.2590	-0.3356
Ba-uncDes	Ar-Locmig	1.1542	0.9780	0.1762	Ba-uncDes	St-Phytub	0.9518	1.3766	-0.4249
Ba-uncDes	Ar-Manjur	1.2687	1.0260	0.2427	Ba-uncDes	St-Pilapi	1.0263	1.2365	-0.2102
Ba-uncDes	Ar-Neopin	1.1432	0.9742	0.1690	Ba-uncDes	St-Pytoli	1.0887	1.2434	-0.1546
Ba-uncDes	Ar-Nillug	1.0912	0.9395	0.1516					
Ba-uncDes	Ar-Onttau	1.2440	1.0253	0.2188					
Ba-uncDes	Ar-Opebru	1.2154	1.0451	0.1703					
Ba-uncDes	Ar-Osmbic	1.1811	1.1735	0.0077					
Ba-uncDes	Ar-Partep	1.1814	1.0421	0.1393					
Ba-uncDes	Ar-Phesol	1.1749	1.0222	0.1527					
Ba-uncDes	Ar-Pluxyl	1.3419	0.9508	0.3911					
Ba-uncDes	Ar-Pseelo	1.2382	0.9949	0.2433					
Ba-uncDes	Ar-Schgra	1.2106	0.9928	0.2178					
Ba-uncDes	Ar-Sipfla	1.3212	1.0665	0.2547					
Ba-uncDes	Ar-Sitmis	1.2831	0.9175	0.3656					
Ba-uncDes	Ar-Sogfur	1.1860	0.9585	0.2275					
Ba-uncDes	Ar-Stedum	1.1881	0.9351	0.2530					
Ba-uncDes	Ar-Stemim	1.1643	1.0397	0.1246					
Ba-uncDes	Ar-Temlon	1.1753	1.0754	0.0999					
Ba-uncDes	Ar-Thrpal	1.2885	1.1533	0.1352					
Ba-uncDes	Ar-Timbar	1.1172	1.0247	0.0924					
Ba-uncDes	Ar-Timcri	1.1065	1.0283	0.0781					
Ba-uncDes	Ar-Timgen	1.1288	1.0055	0.1234					
Ba-uncDes	Ar-Timmon	1.1065	1.0283	0.0781					
Ba-uncDes	Ar-Timpop	1.1222	1.0289	0.0932					
Ba-uncDes	Ar-Timshe	1.1137	1.0277	0.0860					
Ba-uncDes	Ar-Timtah	1.1172	1.0254	0.0918					
Ba-uncDes	Ar-Trivap	1.2533	1.0516	0.2016					
Ba-uncDes	Ar-Tutabs	1.1800	0.9822	0.1978					
Ba-uncDes	Ar-Vantam	1.2650	1.0566	0.2084					
Ba-uncDes	Fu-Lobtra	1.0346	1.3023	-0.2678					
Ba-uncDes	Fu-Morsp.	0.9679	1.3016	-0.3336					
Ba-uncDes	Mo-Halrub	1.4243	0.9650	0.4593					
Ba-uncDes	Mo-Mermer	0.8964	1.0009	-0.1045					
Ba-uncDes	Ne-Caeele	1.2871	1.2950	-0.0079					
Ba-uncDes	Ne-Caenig	1.2506	1.2950	-0.0444					
Ba-uncDes	St-Aphast	0.9099	1.1736	-0.2637					
Ba-uncDes	St-Aphinv	0.9628	1.1843	-0.2215					
Ba-uncDes	St-Aphste	0.9272	1.0621	-0.1349					
Ba-uncDes	St-Phycam	0.8490	1.1545	-0.3055					
Ba-uncDes	St-Physyr	0.8750	1.2366	-0.3616					
Ba-uncDes	St-Phytub	0.8915	1.3309	-0.4394					
Ba-uncDes	St-Pilapi	0.9386	1.2160	-0.2774					
Ba-uncDes	St-Pytoli	0.8458	1.2229	-0.3771					
