# Supplementary material for: Prokaryotic and Eukaryotic Horizontal Transfer of Sailor (DD82E), a New Superfamily of IS630-Tc1-Mariner DNA Transposons
Source: Biology (Basel). 2021 Oct 7;10(10):1005. doi: 10.3390/biology10101005 (PMC8533490; doi:10.3390/biology10101005)
Supplement: Supplementary file 1 [file biology-10-01005-s001.zip › Supplementary Material/Supplementary Table S5.pdf]

HT of cytoplasm ribosomal protein L3				
Species 1	Species 2	Distance of L3	Distance of Sailor	Difference of Sailor and L3
Ba-Delbac	Ba-Desbac	0.9431	0.2391	0.7040
Ba-Delbac	Ba-Legsp.	0.9173	0.6476	0.2697
Ba-Delbac	Ba-uncDes	0.7349	0.6621	0.0728
Ba-Desbac	Ba-Legsp.	0.7104	0.6494	0.0610
Ba-Desbac	Ba-uncDes	0.7390	0.7217	0.0173
Ba-Legsp.	Ba-uncDes	0.8450	0.5166	0.3284
Ne-Caeele	Ne-Caenig	0.0745	0.0000	0.0745
Ar-Timcri	Ar-Timpop	0.0470	0.0022	0.0448
Ar-Helarm	Ar-Helzea	0.0089	0.0047	0.0042
Ar-Timgen	Ar-Timpop	0.0527	0.0088	0.0439
Ar-Timcri	Ar-Timgen	0.0427	0.0096	0.0331
Ar-Eupann	Ar-Eupuro	0.0300	0.0752	-0.0452
Ar-Acypis	Ar-Sipfla	0.1270	0.0822	0.0448
Ar-Danmel	Ar-Lymdis	0.1755	0.1472	0.0283
Ar-Pseelo	Ar-Temlon	0.1976	0.2514	-0.0538
Ar-Epacla	Ar-Tutabs	0.1703	0.2696	-0.0993
St-Aphast	St-Aphinv	0.0812	0.3139	-0.2327
Ar-Bomman	Ar-Neopin	0.2585	0.3391	-0.0806
Ar-Acypis	Ar-Sitmis	0.0307	0.3878	-0.3571
Ar-Sipfla	Ar-Sitmis	0.1222	0.4077	-0.2855
Ar-Opebru	Ar-Tutabs	0.1417	0.4126	-0.2709
Ar-Chisup	Ar-Pluxyl	0.1911	0.4253	-0.2342
Ar-Adohon	Ar-Glocon	0.2093	0.4355	-0.2263
Ar-Helzea	Ar-Pluxyl	0.1213	0.4557	-0.3344
Ar-Helarm	Ar-Pluxyl	0.1228	0.4576	-0.3348
Ar-Schgra	Ar-Sitmis	0.0752	0.5030	-0.4278
Ar-Manjur	Ar-Pluxyl	0.1757	0.5095	-0.3338
Ar-Helarm	Ar-Manjur	0.1652	0.5139	-0.3487
Ar-Epacla	Ar-Opebru	0.1870	0.5175	-0.3305
Ar-Anogla	Ar-Lepdec	0.2245	0.5203	-0.2957
Ar-Helzea	Ar-Manjur	0.1658	0.5212	-0.3554
Ar-Adohon	Ar-Lymdis	0.1704	0.5234	-0.3531
Ar-Chisup	Ar-Danmel	0.2110	0.5294	-0.3184
Ar-Adohon	Ar-Danmel	0.1720	0.5318	-0.3598
Ar-Chisup	Ar-Helzea	0.1781	0.5351	-0.3571
Ar-Chisup	Ar-Helarm	0.1770	0.5356	-0.3585
Ar-Lephet	Ar-Pseelo	0.2675	0.5363	-0.2688
Ar-Glocon	Ar-Lymdis	0.2473	0.5379	-0.2906
Ar-Danmel	Ar-Pluxyl	0.1755	0.5448	-0.3694
Ar-Chisup	Ar-Lymdis	0.1945	0.5477	-0.3532
Ar-Danmel	Ar-Glocon	0.2490	0.5502	-0.3012
Ar-Acypis	Ar-Schgra	0.0734	0.5578	-0.4844
Ar-Adohon	Ar-Chisup	0.1954	0.5583	-0.3629
Ar-Lymdis	Ar-Manjur	0.1787	0.5603	-0.3815
Ar-Chisup	Ar-Manjur	0.2144	0.5699	-0.3555
Ar-Danmel	Ar-Manjur	0.1665	0.5763	-0.4098
Ar-Lymdis	Ar-Pluxyl	0.1703	0.5770	-0.4067
Ar-Danmel	Ar-Helarm	0.1563	0.5826	-0.4263
Ar-Lephet	Ar-Temlon	0.2704	0.5878	-0.3174
Ar-Schgra	Ar-Sipfla	0.1218	0.5884	-0.4666
Ar-Erilan	Ar-Pseelo	0.3484	0.5995	-0.2511
Ar-Galmel	Ar-Opebru	0.2104	0.6000	-0.3896
Ar-Danmel	Ar-Helzea	0.1554	0.6007	-0.4453
Ar-Erilan	Ar-Lephet	0.3159	0.6018	-0.2859
Ar-Helzea	Ar-Lymdis	0.1404	0.6039	-0.4635

HT of cytoplasm ribosomal protein L4				
Species 1	Species 2	Distance of L4	Distance of Sailor	Difference of Sailor and L4
Ba-Delbac	Ba-Desbac	0.8979	0.2391	0.6588
Ba-Delbac	Ba-Legsp.	0.9996	0.6439	0.3558
Ba-Delbac	Ba-uncDes	0.9905	0.6665	0.3240
Ba-Desbac	Ba-Legsp.	0.8840	0.6436	0.2404
Ba-Desbac	Ba-uncDes	0.7898	0.7242	0.0656
Ba-Legsp.	Ba-uncDes	1.2461	0.5314	0.7147
Ar-Timbar	Ar-Timgen	0.0223	0.0000	0.0223
Ar-Timgen	Ar-Timtah	0.0233	0.0000	0.0233
Ne-Caeele	Ne-Caenig	0.0576	0.0000	0.0576
Ar-Timbar	Ar-Timtah	0.0050	0.0007	0.0043
Ar-Timmon	Ar-Timpop	0.0111	0.0014	0.0096
Ar-Timcri	Ar-Timpop	0.0101	0.0022	0.0079
Ar-Timcri	Ar-Timmon	0.0030	0.0022	0.0008
Ar-Timpop	Ar-Timshe	0.0040	0.0036	0.0004
Ar-Timmon	Ar-Timshe	0.0101	0.0051	0.0050
Ar-Timcri	Ar-Timshe	0.0091	0.0058	0.0033
Ar-Timgen	Ar-Timpop	0.0473	0.0088	0.0385
Ar-Timgen	Ar-Timmon	0.0452	0.0088	0.0363
Ar-Timpop	Ar-Timtah	0.0421	0.0094	0.0327
Ar-Timmon	Ar-Timtah	0.0410	0.0094	0.0316
Ar-Timcri	Ar-Timgen	0.0441	0.0096	0.0345
Ar-Timbar	Ar-Timpop	0.0421	0.0101	0.0319
Ar-Timcri	Ar-Timtah	0.0379	0.0101	0.0277
Ar-Timbar	Ar-Timmon	0.0420	0.0102	0.0319
Ar-Timbar	Ar-Timcri	0.0389	0.0109	0.0280
Ar-Timgen	Ar-Timshe	0.0431	0.0112	0.0319
Ar-Timshe	Ar-Timtah	0.0379	0.0131	0.0248
Ar-Timbar	Ar-Timshe	0.0379	0.0138	0.0240
Ar-Eupann	Ar-Eupuro	0.0199	0.0752	-0.0553
Ar-Acypis	Ar-Sipfla	0.1118	0.0822	0.0295
Ar-Aulsol	Ar-Schgra	0.0675	0.1316	-0.0641
Ar-Epacla	Ar-Tutabs	0.1834	0.2696	-0.0862
St-Aphast	St-Aphinv	0.0806	0.3151	-0.2345
Ar-Bomman	Ar-Neopin	0.4610	0.3449	0.1161
Ar-Adohon	Ar-Glocon	0.3431	0.4249	-0.0818
Ar-Helarm	Ar-Pluxyl	0.2761	0.4577	-0.1816
Ar-Anogla	Ar-Lepdec	0.2685	0.4960	-0.2275
Ar-Manjur	Ar-Pluxyl	0.2568	0.5076	-0.2508
Ar-Helarm	Ar-Manjur	0.3609	0.5116	-0.1507
Ar-Adohon	Ar-Lymdis	0.2407	0.5247	-0.2840
Ar-Glocon	Ar-Lymdis	0.3761	0.5274	-0.1513
Ar-Lymdis	Ar-Manjur	0.3177	0.5603	-0.2426
Ar-Acypis	Ar-Schgra	0.0598	0.5639	-0.5041
Ar-Acypis	Ar-Aulsol	0.0522	0.5663	-0.5141
Ar-Lymdis	Ar-Pluxyl	0.2112	0.5772	-0.3660
Ar-Aulsol	Ar-Sipfla	0.1274	0.5892	-0.4618
Ar-Schgra	Ar-Sipfla	0.1279	0.5912	-0.4633
Ar-Lephet	Ar-Temlon	0.3325	0.5914	-0.2589
Ar-Erilan	Ar-Lephet	0.4464	0.5995	-0.1531
Ar-Glocon	Ar-Manjur	0.3974	0.6019	-0.2046
Mo-Halrub	Ar-Tutabs	0.4894	0.6036	-0.1142
Ar-Censcu	Ar-Lephet	0.4510	0.6066	-0.1556
Ar-Helarm	Ar-Lymdis	0.2619	0.6066	-0.3448
Ar-Glocon	Ar-Pluxyl	0.3162	0.6088	-0.2926
Ar-Adohon	Ar-Manjur	0.2742	0.6106	-0.3363

HT of cytoplasm ribosomal protein L3 and L4						
Species 1	Species 2	Distance of L3	Distance of L4	Distance of Sailor	Difference of Sailor and L3	Difference of Sailor and L4
Ba-Delbac	Ba-Desbac	0.9431	0.8979	0.2391	0.7040	0.6588
Ba-Delbac	Ba-Legsp.	0.9173	0.9996	0.6476	0.2697	0.3521
Ba-Delbac	Ba-uncDes	0.7349	0.9905	0.6621	0.0728	0.3284
Ba-Desbac	Ba-Legsp.	0.7104	0.8840	0.6494	0.0610	0.2346
Ba-Desbac	Ba-uncDes	0.7390	0.7898	0.7217	0.0173	0.0681
Ba-Legsp.	Ba-uncDes	0.8450	1.2461	0.5166	0.3284	0.7295
Ar-Acypis	Ar-Sipfla	0.1270	0.1118	0.0822	0.0448	0.0296
Ar-Timcri	Ar-Timgen	0.0427	0.0441	0.0096	0.0331	0.0345
Ar-Timcri	Ar-Timpop	0.0470	0.0101	0.0022	0.0448	0.0079
Ar-Timgen	Ar-Timpop	0.0527	0.0473	0.0088	0.0439	0.0385
Ne-Caeele	Ne-Caenig	0.0745	0.0576	0.0000	0.0745	0.0576

Ar-Helarm	Ar-Lymdis	0.1432	0.6044	-0.4612	Ar-Erilan	Ar-Temlon	0.4456	0.6123	-0.1666
Ar-Glocon	Ar-Pluxyl	0.2064	0.6046	-0.3982	Ar-Eufmex	Ar-Partep	0.4509	0.6164	-0.1655
Ar-Censcu	Ar-Lephet	0.3551	0.6052	-0.2501	Ar-Adohon	Ar-Pluxyl	0.1748	0.6182	-0.4434
Ar-Erilan	Ar-Temlon	0.3724	0.6065	-0.2341	Ar-Partep	Ar-Stedum	0.3206	0.6322	-0.3116
Ar-Chisup	Ar-Glocon	0.2816	0.6066	-0.3250	Ar-Adohon	Ar-Helarm	0.2867	0.6350	-0.3483
Ar-Glocon	Ar-Manjur	0.2402	0.6101	-0.3700	Ar-Nillug	Ar-Phesol	0.4536	0.6366	-0.1830
Ar-Adohon	Ar-Manjur	0.1753	0.6144	-0.4391	Ar-Homvit	Ar-Sogfur	0.3113	0.6382	-0.3269
Ar-Adohon	Ar-Pluxyl	0.1238	0.6146	-0.4908	Ar-Aulsol	Ar-Nillug	0.3872	0.6437	-0.2565
Ar-Partep	Ar-Stedum	0.2262	0.6165	-0.3903	Ar-Pluxyl	Ec-Ophspi	0.6151	0.6498	-0.0347
Ar-Limcal	Ar-Pseelo	0.3539	0.6192	-0.2653	Ar-Acypis	Ar-Nillug	0.5210	0.6555	-0.1344
Ar-Eufmex	Ar-Partep	0.3536	0.6194	-0.2658	Ar-Anogla	Ar-Censcu	0.5143	0.6574	-0.1431
Ar-Censcu	Ar-Pseelo	0.3723	0.6253	-0.2530	Ar-Anogla	Ar-Calmac	0.2976	0.6576	-0.3600
Ar-Phesol	Ar-Sitmis	0.3029	0.6255	-0.3226	Ar-Phesol	Ar-Sipfla	0.4231	0.6591	-0.2360
Ar-Nillug	Ar-Sitmis	0.3108	0.6266	-0.3158	Ar-Nillug	Ar-Sipfla	0.5335	0.6618	-0.1283
Ar-Adohon	Ar-Helzea	0.1321	0.6369	-0.5048	Ar-Nillug	Ar-Schgra	0.4853	0.6629	-0.1776
Ar-Nillug	Ar-Phesol	0.3534	0.6371	-0.2837	Ar-Amytra	Ar-Tutabs	0.2196	0.6652	-0.4455
Ar-Adohon	Ar-Helarm	0.1282	0.6394	-0.5111	Ar-Anogla	Ar-Limcal	0.3516	0.6667	-0.3151
Ar-Anogla	Ar-Calmac	0.2219	0.6454	-0.4235	Ar-Galmel	Ar-Tutabs	0.2056	0.6687	-0.4631
Ar-Pluxyl	Ec-Ophspi	0.3489	0.6497	-0.3008	Ar-Eufmex	Ar-Stedum	0.4850	0.6743	-0.1893
Mo-Halrub	Ar-Tutabs	0.3646	0.6573	-0.2926	Ar-Acypis	Ar-Phesol	0.4318	0.6775	-0.2457
Ar-Homvit	Ar-Sogfur	0.2284	0.6576	-0.4292	Mo-Mermer	Ar-Eufmex	0.4825	0.6776	-0.1951
Ar-Anogla	Ar-Censcu	0.3597	0.6590	-0.2993	Ar-Censcu	Ar-Limcal	0.5174	0.6783	-0.1609
Ar-Amytra	Ar-Opebru	0.1728	0.6607	-0.4878	Ar-Glocon	Ar-Helarm	0.4255	0.6789	-0.2533
Ar-Phesol	Ar-Sipfla	0.2885	0.6629	-0.3744	Ar-Erilan	Ar-Osmbic	0.4289	0.6897	-0.2608
Ar-Censcu	Ar-Limcal	0.3915	0.6646	-0.2731	Ar-Lephet	Ar-Limcal	0.4144	0.6904	-0.2759
Ar-Galmel	Ar-Tutabs	0.1931	0.6697	-0.4766	Ar-Pluxyl	Ar-Sogfur	0.4309	0.6987	-0.2678
Ar-Anogla	Ar-Limcal	0.2876	0.6705	-0.3829	Ar-Locmig	Ar-Lymdis	0.4829	0.7001	-0.2172
Ar-Eufmex	Ar-Stedum	0.3515	0.6742	-0.3227	Ar-Anogla	Ar-Lephet	0.4559	0.7024	-0.2465
Ar-Amytra	Ar-Tutabs	0.1624	0.6749	-0.5124	Ar-Anogla	Ar-Eufmex	0.4939	0.7043	-0.2105
Ar-Nillug	Ar-Schgra	0.3186	0.6755	-0.3569	Mo-Limfor	Ar-Pluxyl	0.6220	0.7053	-0.0833
Mo-Mermer	Ar-Eufmex	0.4363	0.6776	-0.2412	Ar-Censcu	Ar-Erilan	0.5373	0.7076	-0.1702
Ar-Lephet	Ar-Limcal	0.3276	0.6781	-0.3505	Ar-Calmac	Ar-Lephet	0.4481	0.7089	-0.2608
Ar-Glocon	Ar-Helzea	0.2122	0.6788	-0.4666	Ar-Helarm	Ec-Ophspi	0.6167	0.7102	-0.0935
Ar-Acypis	Ar-Phesol	0.2984	0.6813	-0.3829	Ar-Osmbic	Ar-Temlon	0.2543	0.7134	-0.4591
Ar-Glocon	Ar-Helarm	0.2153	0.6814	-0.4662	Ar-Neopin	Ar-Tutabs	0.4437	0.7208	-0.2771
Ar-Erilan	Ar-Osmbic	0.3227	0.6881	-0.3654	Mo-Mermer	Ar-Neopin	0.5083	0.7208	-0.2126
Ar-Chisup	Ec-Ophspi	0.3840	0.6941	-0.3100	Ar-Lymdis	Ar-Sogfur	0.4223	0.7235	-0.3012
Ar-Anogla	Ar-Pseelo	0.3366	0.6960	-0.3594	Ar-Manjur	Ar-Sogfur	0.5166	0.7237	-0.2071
Ar-Pluxyl	Ar-Sogfur	0.2487	0.6962	-0.4475	Ar-Lepdec	Ar-Limcal	0.3848	0.7237	-0.3389
Ar-Acypis	Ar-Nillug	0.3131	0.6966	-0.3835	Ar-Homvit	Ar-Pluxyl	0.4744	0.7271	-0.2526
Mo-Limfor	Ar-Pluxyl	0.3576	0.6998	-0.3422	Ar-Homvit	Ar-Trivap	0.4383	0.7281	-0.2898
Ar-Locmig	Ar-Lymdis	0.2970	0.7036	-0.4066	St-Pilapi	St-Pytoli	0.2292	0.7291	-0.4999
Ar-Anogla	Ar-Lephet	0.3113	0.7046	-0.3933	Mo-Halrub	Mo-Limfor	0.3688	0.7293	-0.3605
Ar-Helarm	Ec-Ophspi	0.3602	0.7066	-0.3464	Ar-Calmac	Ar-Censcu	0.5127	0.7297	-0.2170
Ar-Nillug	Ar-Sipfla	0.3386	0.7088	-0.3701	Ar-Anogla	Ar-Stemim	0.5007	0.7297	-0.2291
Ar-Phesol	Ar-Schgra	0.2834	0.7110	-0.4277	Ar-Phesol	Ar-Schgra	0.4117	0.7297	-0.3181
Ar-Lepdec	Ar-Pseelo	0.3123	0.7116	-0.3993	Ar-Amytra	Ar-Galmel	0.2092	0.7311	-0.5219
Ar-Anogla	Ar-Eufmex	0.3019	0.7123	-0.4104	Mo-Halrub	Ar-Epacla	0.4938	0.7313	-0.2375
Ar-Censcu	Ar-Erilan	0.3690	0.7129	-0.3439	Ar-Lymdis	Ec-Ophspi	0.5777	0.7313	-0.1536
Ar-Osmbic	Ar-Pseelo	0.2441	0.7132	-0.4691	Ar-Epacla	Ar-Eufmex	0.4856	0.7321	-0.2465
Ar-Calmac	Ar-Lephet	0.3152	0.7145	-0.3993	Ar-Stemim	Ar-Tutabs	0.5523	0.7326	-0.1803
Ar-Amytra	Ar-Galmel	0.1704	0.7150	-0.5446	Ar-Censcu	Ar-Temlon	0.5003	0.7334	-0.2331
Ar-Lymdis	Ar-Sogfur	0.2740	0.7159	-0.4418	Mo-Mermer	Ar-Tutabs	0.5752	0.7336	-0.1584
Ar-Manjur	Ar-Sogfur	0.2855	0.7164	-0.4308	Ar-Ladful	Ar-Tutabs	0.4914	0.7341	-0.2426
Fn-Lobtra	Fn-Morsp.	0.1241	0.7178	-0.5937	Ar-Erilan	Ar-Limcal	0.4656	0.7349	-0.2693
Ar-Osmbic	Ar-Temlon	0.2674	0.7196	-0.4522	Ar-Lepdec	Ar-Lephet	0.4381	0.7359	-0.2978
Mo-Mermer	Ar-Neopin	0.3792	0.7204	-0.3412	Ar-Amytra	Ar-Eufmex	0.5184	0.7369	-0.2185
St-Pilapi	St-Pytoli	0.1688	0.7216	-0.5528	Ar-Anogla	Ar-Erilan	0.5362	0.7389	-0.2027
Ar-Censcu	Ar-Lepdec	0.3362	0.7228	-0.3866	Ar-Homvit	Ar-Lymdis	0.4406	0.7391	-0.2985

Ar-Erilan	Ar-Limcal	0.3402	0.7241	-0.3839	Ar-Glocon	Ar-Sogfur	0.4895	0.7402	-0.2507
Ar-Neopin	Ar-Tutabs	0.2631	0.7241	-0.4610	Fu-Lobtra	Fu-Morsp.	0.1182	0.7403	-0.6222
Ar-Lepdec	Ar-Limcal	0.2678	0.7248	-0.4570	Ar-Aulsol	Ar-Phesol	0.3738	0.7404	-0.3666
Mo-Halrub	Ar-Epacla	0.3728	0.7272	-0.3544	Ar-Stemim	Ar-Timgen	0.4457	0.7419	-0.2962
Ar-Lepdec	Ar-Lephet	0.2907	0.7285	-0.4378	Ar-Calmac	Ar-Lepdec	0.2681	0.7460	-0.4780
Ar-Homvit	Ar-Pluxyl	0.2562	0.7300	-0.4738	Mo-Limfor	Ar-Partep	0.5300	0.7473	-0.2173
Ar-Lymdis	Ec-Ophspi	0.3814	0.7313	-0.3499	Ar-Locmig	Ar-Pluxyl	0.5053	0.7478	-0.2425
Ar-Epacla	Ar-Eufmex	0.3342	0.7321	-0.3978	Mo-Halrub	Ar-Pluxyl	0.4850	0.7494	-0.2644
Ar-Anogla	Ar-Erilan	0.2954	0.7328	-0.4374	Ar-Epacla	Ar-Galmel	0.1908	0.7497	-0.5589
Ar-Chisup	Ar-Sogfur	0.2986	0.7338	-0.4353	Ar-Manjur	Ec-Ophspi	0.5608	0.7510	-0.1902
Ar-Homvit	Ar-Lymdis	0.2798	0.7342	-0.4544	Ar-Amytra	Ar-Partep	0.5830	0.7515	-0.1684
Ar-Homvit	Ar-Trivap	0.4203	0.7357	-0.3154	Ar-Censcu	Ar-Lepdec	0.5080	0.7522	-0.2442
Ar-Chisup	Ar-Homvit	0.3001	0.7363	-0.4362	Mo-Halrub	Mo-Mermer	0.3487	0.7522	-0.4035
Ar-Amytra	Ar-Eufmex	0.3297	0.7368	-0.4071	Ar-Adohon	Ar-Homvit	0.4657	0.7537	-0.2880
Ar-Chisup	Ar-Trivap	0.4190	0.7372	-0.3182	Ar-Glocon	Ar-Homvit	0.5031	0.7541	-0.2510
Ar-Chisup	Ar-Locmig	0.2903	0.7377	-0.4474	Ar-Nillug	Ec-Ophspi	0.5427	0.7543	-0.2115
Ar-Censcu	Ar-Temlon	0.4164	0.7391	-0.3228	Mo-Mermer	Ec-Ophspi	0.4673	0.7543	-0.2870
Ar-Helzea	Ec-Ophspi	0.3625	0.7393	-0.3769	Ar-Lephet	Ar-Tutabs	0.5019	0.7548	-0.2530
Mo-Mermer	Ar-Tutabs	0.3837	0.7399	-0.3562	Ar-Pluxyl	Ar-Trivap	0.6055	0.7560	-0.1506
Mo-Limfor	Ar-Partep	0.3576	0.7415	-0.3839	Ar-Limcal	Ar-Temlon	0.4293	0.7565	-0.3273
Ar-Amytra	Ar-Epacla	0.1768	0.7418	-0.5651	Ar-Amytra	Ar-Epacla	0.2092	0.7566	-0.5474
Mo-Halrub	Mo-Limfor	0.3642	0.7432	-0.3790	Ar-Lephet	Ar-Stemim	0.4445	0.7577	-0.3132
Ar-Stemim	Ar-Timgen	0.3528	0.7434	-0.3907	Ar-Bomman	Ar-Tutabs	0.2602	0.7582	-0.4980
Ar-Glocon	Ar-Sogfur	0.2436	0.7443	-0.5006	Ar-Stemim	Ar-Timshe	0.4462	0.7584	-0.3122
Ar-Ladful	Ar-Tutabs	0.3206	0.7448	-0.4242	Mo-Limfor	Ar-Lephet	0.5997	0.7588	-0.1591
Ar-Anogla	Ar-Stemim	0.3465	0.7464	-0.3999	Ar-Locmig	Ar-Sogfur	0.3582	0.7601	-0.4019
Ar-Chisup	Ar-Sitmis	0.3152	0.7473	-0.4321	Ar-Stemim	Ar-Timpop	0.4395	0.7609	-0.3214
Mo-Halrub	An-Hydele	0.4124	0.7476	-0.3352	Ar-Stemim	Ar-Timcri	0.4420	0.7612	-0.3192
Ar-Calmac	Ar-Lepdec	0.2256	0.7476	-0.5221	Mo-Mermer	Ar-Onttau	0.4971	0.7618	-0.2647
Mo-Mermer	Ar-Onttau	0.4256	0.7482	-0.3226	Ar-Locmig	Ar-Trivap	0.4623	0.7639	-0.3016
Ar-Stemim	Ar-Tutabs	0.3672	0.7497	-0.3825	Ar-Stemim	Ar-Timmon	0.4442	0.7641	-0.3199
Ar-Limcal	Ar-Temlon	0.3418	0.7498	-0.4080	Ar-Phesol	Ar-Pluxyl	0.6297	0.7646	-0.1349
Ar-Manjur	Ec-Ophspi	0.3826	0.7499	-0.3674	Ar-Eufmex	Ar-Tutabs	0.5197	0.7660	-0.2464
Ar-Epacla	Ar-Galmel	0.1920	0.7500	-0.5580	Ar-Anogla	Ar-Temlon	0.4447	0.7670	-0.3224
Ar-Calmac	Ar-Censcu	0.3818	0.7503	-0.3685	Ar-Onttau	Ar-Tutabs	0.4900	0.7675	-0.2775
Ar-Lephet	Ar-Stemim	0.3698	0.7509	-0.3811	Ar-Epacla	Ar-Neopin	0.4217	0.7681	-0.3464
Ar-Amytra	Ar-Partep	0.3625	0.7512	-0.3886	Mo-Halrub	Ar-Sipfla	0.5174	0.7694	-0.2520
Mo-Mermer	Ar-Sitmis	0.4170	0.7520	-0.3350	Ar-Homvit	Ar-Manjur	0.5620	0.7694	-0.2075
Mo-Halrub	Ar-Chisup	0.4176	0.7529	-0.3353	Ar-Stemim	Ar-Timtah	0.4394	0.7700	-0.3307
Ar-Eufmex	Ar-Vantam	0.3112	0.7533	-0.4421	Ar-Amytra	Ar-Stedum	0.5767	0.7703	-0.1936
Mo-Mermer	Ec-Ophspi	0.3554	0.7539	-0.3984	Ar-Stemim	Ar-Timbar	0.4394	0.7703	-0.3310
Ar-Locmig	Ar-Pluxyl	0.2969	0.7539	-0.4570	Ar-Limcal	Ar-Timgen	0.4107	0.7714	-0.3607
Ar-Neopin	Ar-Opebru	0.2857	0.7543	-0.4686	Mo-Mermer	Ar-Acypis	0.5564	0.7717	-0.2153
Ar-Amytra	Ar-Stedum	0.3690	0.7547	-0.3857	Ar-Censcu	Ar-Timgen	0.5073	0.7720	-0.2647
Mo-Limfor	Ar-Lephet	0.3561	0.7555	-0.3994	Ar-Adohon	Ar-Sogfur	0.4371	0.7720	-0.3349
Ar-Anogla	Ar-Temlon	0.3286	0.7558	-0.4272	Ar-Epacla	Ar-Stedum	0.5950	0.7721	-0.1771
Ar-Pluxyl	Ar-Trivap	0.4162	0.7560	-0.3398	Ar-Lephet	Ec-Ophspi	0.5572	0.7724	-0.2152
Ar-Stemim	Ar-Timpop	0.3532	0.7573	-0.4041	Ar-Timbar	Ec-Ophspi	0.4901	0.7726	-0.2825
Ar-Stemim	Ar-Timcri	0.3485	0.7576	-0.4091	Ar-Timtah	Ec-Ophspi	0.4903	0.7726	-0.2823
Mo-Halrub	Mo-Mermer	0.3516	0.7593	-0.4077	Ar-Bomman	Ar-Eufmex	0.4856	0.7732	-0.2875
Mo-Halrub	Ar-Pluxyl	0.3894	0.7595	-0.3700	Ar-Ladful	Ar-Neopin	0.3819	0.7755	-0.3937
Ar-Lephet	Ar-Tutabs	0.3043	0.7595	-0.4552	Ar-Helarm	Ar-Homvit	0.5118	0.7767	-0.2649
Mo-Mermer	Ar-Opebru	0.3960	0.7597	-0.3637	Ar-Locmig	Ar-Tutabs	0.4903	0.7767	-0.2865
Ar-Locmig	Ar-Sogfur	0.3107	0.7606	-0.4499	Ar-Timgen	Ec-Ophspi	0.4968	0.7774	-0.2806
Ar-Limcal	Ar-Timgen	0.3615	0.7611	-0.3996	Ar-Anogla	Ar-Timshe	0.4306	0.7778	-0.3473
Ar-Bomman	Ar-Tutabs	0.1817	0.7621	-0.5804	Mo-Halrub	Ar-Acypis	0.5357	0.7802	-0.2446
Ar-Eufmex	Ar-Opebru	0.3587	0.7624	-0.4036	Ar-Sipfla	Ec-Ophspi	0.5587	0.7803	-0.2216
Ar-Epacla	Ar-Neopin	0.2853	0.7625	-0.4772	Ar-Calmac	Ar-Limcal	0.3720	0.7808	-0.4088
Ar-Calmac	Ar-Limcal	0.2771	0.7636	-0.4864	Ar-Lephet	Ar-Osmbic	0.3151	0.7810	-0.4659

Ar-Opebru	Ar-Sitmis	0.3430	0.7637	-0.4207	Ar-Epacla	Ar-Ladful	0.4768	0.7818	-0.3050
Ar-Danmel	Ar-Sogfur	0.2689	0.7642	-0.4953	Ar-Anogla	Ar-Timcri	0.4288	0.7826	-0.3537
Ar-Locmig	Ar-Trivap	0.4190	0.7647	-0.3457	Ar-Helarm	Ar-Sogfur	0.4752	0.7826	-0.3074
Ar-Onttau	Ar-Opebru	0.3282	0.7649	-0.4368	Mo-Limfor	Ar-Limcal	0.5371	0.7829	-0.2458
Mo-Mermer	Ar-Chisup	0.4192	0.7651	-0.3458	Ar-Anogla	Ar-Timpop	0.4287	0.7829	-0.3542
Ar-Ladful	Ar-Opebru	0.3263	0.7681	-0.4418	Ar-Anogla	Ec-Ophspi	0.5653	0.7831	-0.2178
Ar-Sitmis	Ec-Ophspi	0.3852	0.7682	-0.3830	Ar-Glocon	Ar-Locmig	0.5360	0.7835	-0.2475
Ar-Adohon	Ar-Homvit	0.2611	0.7682	-0.5072	Ar-Anogla	Ar-Timmon	0.4545	0.7835	-0.3290
Ar-Phesol	Ar-Pluxyl	0.3605	0.7683	-0.4078	Ar-Eufmex	Ar-Trivap	0.4332	0.7844	-0.3512
Ar-Timgen	Ec-Ophspi	0.3998	0.7684	-0.3686	Ar-Anogla	Ar-Timbar	0.4188	0.7848	-0.3660
Ar-Censcu	Ar-Sitmis	0.3559	0.7694	-0.4136	Ar-Anogla	Ar-Timtah	0.4223	0.7848	-0.3625
Ar-Calmac	Ar-Pseelo	0.3244	0.7695	-0.4451	Ar-Homvit	Ar-Phesol	0.4796	0.7852	-0.3056
Ar-Sitmis	Ar-Tutabs	0.3378	0.7695	-0.4317	Ar-Timpop	Ec-Ophspi	0.4999	0.7854	-0.2856
Ar-Homvit	Ar-Manjur	0.2764	0.7700	-0.4936	Ar-Neopin	Ar-Onttau	0.4496	0.7863	-0.3367
Mo-Limfor	Ar-Chisup	0.3921	0.7704	-0.3784	Mo-Halrub	Ar-Ladful	0.5005	0.7863	-0.2859
Ar-Glocon	Ar-Homvit	0.2689	0.7714	-0.5025	Ar-Erilan	Ar-Lepdec	0.5448	0.7866	-0.2418
Ar-Onttau	Ar-Tutabs	0.3295	0.7727	-0.4432	Mo-Halrub	Ar-Eufmex	0.4674	0.7868	-0.3194
Ar-Neopin	Ar-Vantam	0.2786	0.7728	-0.4941	Ar-Limcal	Ar-Timpop	0.4060	0.7871	-0.3811
Ar-Bomman	Ar-Eufmex	0.3200	0.7732	-0.4531	Ar-Timmon	Ec-Ophspi	0.4939	0.7873	-0.2934
Mo-Limfor	Ar-Lymdis	0.3970	0.7739	-0.3769	Ar-Epacla	Ar-Onttau	0.4825	0.7875	-0.3051
Ar-Eufmex	Ar-Tutabs	0.3627	0.7741	-0.4114	Ar-Limcal	Ar-Timmon	0.4335	0.7879	-0.3545
Ar-Ladful	Ar-Neopin	0.3062	0.7752	-0.4690	Ar-Anogla	Ar-Timgen	0.4137	0.7883	-0.3746
Ar-Nillug	Ec-Ophspi	0.3431	0.7771	-0.4340	Ar-Timshe	Ec-Ophspi	0.4957	0.7886	-0.2929
Ar-Lephet	Ar-Sitmis	0.2892	0.7775	-0.4883	Mo-Limfor	Ar-Manjur	0.6163	0.7888	-0.1725
Ar-Erilan	Ar-Lepdec	0.2938	0.7779	-0.4841	Ar-Timcri	Ec-Ophspi	0.4939	0.7891	-0.2952
Mo-Limfor	Ar-Manjur	0.3933	0.7782	-0.3850	Mo-Limfor	Ar-Lymdis	0.6178	0.7895	-0.1717
Ar-Neopin	Ar-Onttau	0.2980	0.7784	-0.4803	Mo-Mermer	Ar-Trivap	0.5675	0.7896	-0.2221
Ar-Limcal	Ar-Timpop	0.3479	0.7784	-0.4306	Ar-Eufmex	Ar-Galmel	0.5342	0.7898	-0.2555
Mo-Halrub	Ar-Sipfla	0.4349	0.7784	-0.3436	Ar-Epacla	Ar-Partep	0.5611	0.7901	-0.2290
Ar-Helarm	Ar-Homvit	0.2661	0.7785	-0.5125	Ar-Bomman	Ar-Ladful	0.4582	0.7911	-0.3329
Ar-Timpop	Ec-Ophspi	0.4118	0.7786	-0.3668	Ar-Limcal	Ar-Timcri	0.4078	0.7913	-0.3835
Mo-Mermer	Ar-Trivap	0.4621	0.7789	-0.3168	Ar-Adohon	Ar-Locmig	0.4857	0.7918	-0.3061
Ar-Eufmex	Ar-Trivap	0.4519	0.7800	-0.3281	St-Aphast	St-Aphste	0.1086	0.7924	-0.6838
Ar-Helarm	Ar-Sitmis	0.3146	0.7811	-0.4664	Ar-Limcal	Ar-Timshe	0.4075	0.7937	-0.3862
Ar-Adohon	Ar-Sogfur	0.2438	0.7811	-0.5373	Ar-Calmac	Ar-Erilan	0.4996	0.7938	-0.2942
Ar-Helzea	Ar-Sitmis	0.3145	0.7815	-0.4670	Mo-Mermer	Ar-Lymdis	0.5556	0.7939	-0.2383
Ar-Glocon	Ar-Locmig	0.2962	0.7822	-0.4860	Ar-Limcal	Ar-Osmbic	0.4472	0.7946	-0.3474
Ar-Lephet	Ec-Ophspi	0.3889	0.7823	-0.3934	Ar-Limcal	Ar-Timtah	0.4078	0.7946	-0.3868
Ar-Timcri	Ec-Ophspi	0.3943	0.7823	-0.3880	Mo-Limfor	Ec-Ophspi	0.5453	0.7947	-0.2494
Ar-Limcal	Ar-Timcri	0.3499	0.7827	-0.4328	Ar-Glocon	Ec-Ophspi	0.5986	0.7948	-0.1963
Ar-Helarm	Ar-Sogfur	0.2460	0.7829	-0.5369	Ar-Acypis	Ec-Ophspi	0.5817	0.7949	-0.2132
Ar-Lymdis	Ar-Opebru	0.1779	0.7834	-0.6055	Ar-Limcal	Ar-Timbar	0.4059	0.7950	-0.3890
Ar-Tutabs	Ar-Vantam	0.1776	0.7836	-0.6060	Ar-Eupuro	Ar-Lephet	0.2746	0.7951	-0.5206
Ar-Lephet	Ar-Osmbic	0.2116	0.7847	-0.5732	Ar-Censcu	Ar-Stemim	0.3315	0.7953	-0.4638
Ar-Danmel	Ar-Homvit	0.2764	0.7856	-0.5092	Mo-Mermer	Ar-Nillug	0.5182	0.7954	-0.2772
Ar-Erilan	Ec-Ophspi	0.4046	0.7857	-0.3810	Ar-Sogfur	Ar-Trivap	0.4520	0.7955	-0.3434
Mo-Limfor	An-Hydele	0.3789	0.7861	-0.4072	Ar-Locmig	Ar-Manjur	0.5584	0.7962	-0.2378
Ar-Censcu	Ar-Timgen	0.3842	0.7873	-0.4032	Mo-Batpla	Ar-Pluxyl	0.6681	0.7969	-0.1288
St-Aphast	St-Aphste	0.1078	0.7874	-0.6796	Ar-Lephet	Ar-Timtah	0.4327	0.7978	-0.3651
Ar-Limcal	Ar-Osmbic	0.3142	0.7880	-0.4738	Ar-Lephet	Ar-Timcri	0.4349	0.7985	-0.3636
Ar-Calmac	Ar-Erilan	0.3141	0.7886	-0.4746	Ar-Lephet	Ar-Timgen	0.4256	0.7987	-0.3731
Ar-Epacla	Ar-Stedum	0.3628	0.7889	-0.4261	Ar-Partep	Ar-Tutabs	0.5692	0.7988	-0.2297
Ar-Anogla	Ar-Timcri	0.3675	0.7891	-0.4216	Mo-Limfor	Ar-Censcu	0.5624	0.7990	-0.2365
Ar-Helzea	Ar-Homvit	0.2632	0.7893	-0.5261	Mo-Mermer	Ar-Pluxyl	0.5873	0.7995	-0.2122
Mo-Halrub	Ar-Acypis	0.4151	0.7894	-0.3743	Ar-Eufmex	Ar-Lephet	0.3174	0.8003	-0.4829
Ar-Anogla	Ar-Timpop	0.3594	0.7894	-0.4301	Ar-Lephet	Ar-Timpop	0.4346	0.8003	-0.3658
Ar-Glocon	Ec-Ophspi	0.3632	0.7897	-0.4265	Ar-Lephet	Ar-Timbar	0.4343	0.8004	-0.3661
Ar-Eufmex	Ar-Galmel	0.3051	0.7897	-0.4845	Mo-Mermer	Ar-Epacla	0.5702	0.8008	-0.2306
Mo-Limfor	Ar-Limcal	0.4144	0.7897	-0.3753	Ar-Lymdis	Ar-Trivap	0.5612	0.8011	-0.2398

Ar-Sogfur	Ar-Trivap	0.4234	0.7898	-0.3665	Ar-Lephet	Ar-Timshe	0.4312	0.8011	-0.3699
Ar-Anogla	Ar-Timgen	0.3564	0.7899	-0.4335	Mo-Batpla	Mo-Limfor	0.4479	0.8014	-0.3536
Ar-Helzea	Ar-Sogfur	0.2440	0.7899	-0.5459	Ar-Epacla	Ar-Pluxyl	0.1890	0.8016	-0.6125
Ar-Censcu	Ar-Stemim	0.2335	0.7907	-0.5572	Mo-Mermer	Ar-Sipfla	0.5303	0.8018	-0.2715
Ar-Bomman	Ar-Ladful	0.3158	0.7908	-0.4750	Ar-Lephet	Ar-Timmon	0.4332	0.8021	-0.3689
Ar-Locmig	Ar-Manjur	0.2823	0.7918	-0.5095	Ar-Eufmex	Ar-Locmig	0.3707	0.8028	-0.4321
Ar-Chisup	Ar-Opebru	0.2222	0.7923	-0.5701	Ar-Eufmex	Ar-Helarm	0.5962	0.8030	-0.2067
Ar-Locmig	Ar-Tutabs	0.3060	0.7925	-0.4865	Ar-Adohon	Ec-Ophspi	0.6030	0.8030	-0.2001
Mo-Halrub	Ar-Sitmis	0.4187	0.7938	-0.3751	Ar-Adohon	Ar-Phesol	0.6004	0.8031	-0.2027
Ar-Epacla	Ar-Ladful	0.3197	0.7938	-0.4740	Mo-Mermer	Ar-Stedum	0.5119	0.8034	-0.2915
Ar-Lepdec	Ar-Stemim	0.3169	0.7938	-0.4769	Mo-Limfor	Ar-Nillug	0.5803	0.8036	-0.2232
Mo-Mermer	Ar-Acypis	0.4095	0.7948	-0.3853	Ar-Acypis	Ar-Eufmex	0.4571	0.8039	-0.3467
Mo-Limfor	Ar-Sogfur	0.3937	0.7955	-0.4018	Ar-Calmac	Ar-Stemim	0.4486	0.8045	-0.3559
Mo-Batpla	Ar-Pluxyl	0.3840	0.7957	-0.4117	Ar-Lepdec	Ec-Ophspi	0.5819	0.8048	-0.2229
Ar-Lymdis	Ar-Trivap	0.4180	0.7958	-0.3778	Ar-Acypis	Ar-Tutabs	0.5947	0.8053	-0.2106
Ar-Erilan	Ar-Sitmis	0.1297	0.7963	-0.6666	Ar-Censcu	Ar-Eufmex	0.4706	0.8064	-0.3357
Ar-Epacla	Ar-Onttau	0.3052	0.7968	-0.4916	Ar-Homvit	Ec-Ophspi	0.5357	0.8065	-0.2708
Ar-Pluxyl	Ar-Sitmis	0.3101	0.7968	-0.4868	Ar-Bomman	Ar-Partep	0.5495	0.8067	-0.2572
Mo-Limfor	Ar-Censcu	0.3280	0.7969	-0.4689	Ar-Eufmex	Ec-Ophspi	0.5683	0.8070	-0.2388
Ar-Adohon	Ar-Phesol	0.3729	0.7973	-0.4244	Ar-Glocon	Ar-Phesol	0.6168	0.8078	-0.1910
Ar-Glocon	Ar-Phesol	0.3860	0.7975	-0.4115	Ar-Censcu	Ar-Timtah	0.5046	0.8080	-0.3034
Ar-Pseelo	Ar-Stemim	0.3644	0.7976	-0.4332	Mo-Mermer	Ar-Bomman	0.5552	0.8081	-0.2529
Ar-Opebru	Ar-Vantam	0.1766	0.7984	-0.6218	Ar-Censcu	Ar-Timbar	0.5028	0.8083	-0.3055
Mo-Mermer	Ar-Pluxyl	0.3812	0.7984	-0.4172	Ar-Partep	Ec-Ophspi	0.5398	0.8087	-0.2689
Ar-Homvit	Ar-Phesol	0.3463	0.7986	-0.4524	Ar-Erilan	Ec-Ophspi	0.5576	0.8088	-0.2511
Ar-Partep	Ar-Tutabs	0.3549	0.7988	-0.4440	Mo-Limfor	Ar-Ampamp	0.6299	0.8089	-0.1790
Ar-Lephet	Ar-Timcri	0.2959	0.7988	-0.5029	Ar-Aulsol	Ec-Ophspi	0.5163	0.8092	-0.2928
Ar-Bomman	Ar-Vantam	0.1782	0.7993	-0.6211	Ar-Helarm	Ar-Trivap	0.6466	0.8098	-0.1632
Mo-Mermer	Ar-Stedum	0.3857	0.8004	-0.4147	Ar-Anogla	Ar-Schgra	0.5718	0.8098	-0.2380
Ar-Lephet	Ar-Timgen	0.2977	0.8005	-0.5029	Mo-Mermer	Ar-Phesol	0.5592	0.8099	-0.2507
Ar-Anogla	Ar-Schgra	0.3183	0.8006	-0.4823	Mo-Mermer	Ar-Adohon	0.5932	0.8102	-0.2170
Ar-Lephet	Ar-Timpop	0.2940	0.8007	-0.5067	Ar-Calmac	Ar-Eufmex	0.4371	0.8102	-0.3732
Mo-Batpla	Mo-Limfor	0.1670	0.8009	-0.6339	Ar-Limcal	Ar-Stemim	0.4469	0.8108	-0.3639
Ar-Adohon	Ec-Ophspi	0.3641	0.8010	-0.4369	Ar-Limcal	Ec-Ophspi	0.5718	0.8122	-0.2404
Mo-Mermer	Ar-Epacla	0.3834	0.8012	-0.4178	Mo-Mermer	Ar-Amytra	0.5600	0.8125	-0.2525
Ar-Onttau	Ar-Vantam	0.3050	0.8015	-0.4965	Ar-Censcu	Ar-Osmbic	0.4593	0.8126	-0.3533
Ar-Limcal	Ar-Opebru	0.3222	0.8024	-0.4802	Ar-Sipfla	Ar-Tutabs	0.6067	0.8132	-0.2065
Mo-Mermer	Ar-Lymdis	0.4006	0.8028	-0.4022	Ar-Manjur	Ar-Trivap	0.6685	0.8136	-0.1450
Ar-Chisup	Ar-Lephet	0.3037	0.8035	-0.4997	Ar-Lepdec	Ar-Stemim	0.4784	0.8140	-0.3356
Ar-Pseelo	Ar-Timgen	0.3358	0.8041	-0.4682	Mo-Halrub	Ar-Neopin	0.4727	0.8143	-0.3416
Ar-Bomman	Ar-Epacla	0.1910	0.8041	-0.6131	Ar-Ampamp	Ec-Ophspi	0.6059	0.8147	-0.2088
Ar-Eufmex	Ar-Lephet	0.2456	0.8042	-0.5586	Ar-Eufmex	Ar-Sipfla	0.4819	0.8149	-0.3330
Mo-Halrub	Ar-Eufmex	0.4703	0.8044	-0.3341	Ar-Amytra	Ar-Bomman	0.2663	0.8155	-0.5492
Ar-Epacla	Ar-Pluxyl	0.1642	0.8046	-0.6404	Mo-Limfor	Ar-Sogfur	0.5570	0.8159	-0.2589
Ar-Epacla	Ar-Sitmis	0.3200	0.8046	-0.4846	Mo-Limfor	Ar-Epacla	0.6407	0.8168	-0.1761
Ar-Censcu	Ar-Osmbic	0.3684	0.8053	-0.4369	Mo-Halrub	Ar-Bomman	0.4916	0.8176	-0.3259
Ar-Censcu	Ar-Eufmex	0.3657	0.8053	-0.4396	Ar-Neopin	Ar-Nillug	0.3722	0.8185	-0.4463
Ar-Anogla	Ar-Sitmis	0.3199	0.8053	-0.4854	Ar-Censcu	Ar-Timcri	0.5056	0.8187	-0.3131
Ar-Partep	Ec-Ophspi	0.3839	0.8054	-0.4215	Ar-Censcu	Ar-Timpop	0.4920	0.8191	-0.3270
Mo-Halrub	Ar-Ladful	0.4179	0.8062	-0.3884	Ar-Censcu	Ar-Timshe	0.4937	0.8191	-0.3254
Ar-Adohon	Ar-Locmig	0.3073	0.8063	-0.4990	Ar-Galmel	Ar-Lymdis	0.2294	0.8198	-0.5904
Mo-Mermer	Ar-Partep	0.3818	0.8064	-0.4245	Ar-Censcu	Ar-Timmon	0.5037	0.8200	-0.3162
Ar-Amytra	Ar-Bomman	0.1776	0.8068	-0.6292	Ar-Epacla	Ar-Locmig	0.4874	0.8201	-0.3327
Ar-Eufmex	Ar-Helarm	0.3529	0.8069	-0.4540	Ar-Galmel	Ar-Limcal	0.5312	0.8203	-0.2891
Ar-Eufmex	Ar-Helzea	0.3579	0.8069	-0.4490	Ar-Galmel	Ar-Pluxyl	0.2442	0.8206	-0.5764
Ar-Eufmex	Ec-Ophspi	0.4364	0.8070	-0.3706	Mo-Halrub	Ar-Nillug	0.4542	0.8211	-0.3669
Ar-Manjur	Ar-Trivap	0.4078	0.8073	-0.3996	Ar-Nillug	Ar-Tutabs	0.4368	0.8213	-0.3845
Ar-Eufmex	Ar-Locmig	0.2901	0.8073	-0.5172	Ar-Lymdis	Ar-Tutabs	0.2320	0.8218	-0.5898
Ar-Danmel	Ec-Ophspi	0.3655	0.8074	-0.4419	Ar-Eufmex	Ar-Onttau	0.4595	0.8228	-0.3633

Ar-Amytra	Ar-Ladful	0.3102	0.8075	-0.4972	Ar-Censcu	Ec-Ophspi	0.5419	0.8235	-0.2815
Ar-Acypis	Ar-Eufmex	0.3338	0.8077	-0.4739	Ar-Galmel	Ar-Sogfur	0.4506	0.8235	-0.3728
Ar-Calmac	Ar-Stemim	0.3576	0.8078	-0.4502	Ar-Amytra	Ar-Onttau	0.4870	0.8236	-0.3366
Ar-Chisup	Ar-Eufmex	0.3160	0.8079	-0.4919	Ar-Anogla	Ar-Locmig	0.4570	0.8241	-0.3671
Ar-Helarm	Ar-Trivap	0.4051	0.8084	-0.4033	Mo-Mermer	Ar-Manjur	0.6072	0.8245	-0.2173
Ar-Partep	Ar-Sitmis	0.3529	0.8093	-0.4564	Mo-Limfor	Ar-Lepdec	0.5801	0.8246	-0.2445
Ar-Pseelo	Ar-Timcri	0.3221	0.8094	-0.4873	Mo-Limfor	Ar-Glocon	0.6418	0.8249	-0.1831
Mo-Limfor	Ec-Ophspi	0.3666	0.8096	-0.4430	Ar-Homvit	Ar-Locmig	0.3225	0.8251	-0.5025
Ar-Pseelo	Ar-Timpop	0.3168	0.8098	-0.4930	Ar-Neopin	Ar-Partep	0.4946	0.8253	-0.3307
Mo-Mermer	Ar-Phesol	0.4146	0.8099	-0.3954	Ar-Lymdis	Ar-Phesol	0.5894	0.8258	-0.2364
Mo-Mermer	Ar-Nillug	0.3825	0.8103	-0.4278	Ar-Erilan	Ar-Timgen	0.4528	0.8268	-0.3740
Ar-Anogla	Ec-Ophspi	0.3883	0.8105	-0.4222	Ar-Amytra	Ar-Lephet	0.4758	0.8269	-0.3510
Ar-Pseelo	Ec-Ophspi	0.4187	0.8112	-0.3925	Ar-Phesol	Ar-Tutabs	0.5923	0.8271	-0.2347
Ar-Opebru	Ar-Pluxyl	0.1655	0.8116	-0.6460	Ar-Eupann	Ar-Lephet	0.2773	0.8272	-0.5498
Mo-Mermer	Ar-Bomman	0.4065	0.8119	-0.4054	Ar-Galmel	Ar-Phesol	0.6057	0.8273	-0.2216
Ar-Lephet	Ar-Opebru	0.3068	0.8120	-0.5052	Ar-Epacla	Ar-Schgra	0.5537	0.8273	-0.2736
Ar-Epacla	Ar-Partep	0.3580	0.8122	-0.4542	Mo-Limfor	Ar-Neopin	0.5670	0.8286	-0.2617
Ar-Opebru	Ar-Pseelo	0.3483	0.8126	-0.4643	Ar-Helarm	Ar-Locmig	0.5447	0.8291	-0.2844
Mo-Limfor	Ar-Ampamp	0.4273	0.8132	-0.3860	Ar-Epacla	Ar-Stemim	0.5367	0.8292	-0.2925
Mo-Mermer	Ar-Amytra	0.3853	0.8136	-0.4283	Ar-Amytra	Ar-Ladful	0.4953	0.8292	-0.3339
Mo-Mermer	Ar-Adohon	0.3885	0.8136	-0.4251	Ar-Eufmex	Ar-Phesol	0.4770	0.8292	-0.3522
Ar-Danmel	Ar-Trivap	0.4169	0.8138	-0.3969	Mo-Mermer	Ar-Schgra	0.5471	0.8295	-0.2824
Ar-Lepdec	Ec-Ophspi	0.3690	0.8140	-0.4450	Mo-Limfor	Ar-Helarm	0.6580	0.8297	-0.1717
Ar-Limcal	Ec-Ophspi	0.3566	0.8147	-0.4581	Ar-Galmel	Ar-Partep	0.5905	0.8299	-0.2394
Ar-Limcal	Ar-Stemim	0.3743	0.8150	-0.4407	Mo-Mermer	Ar-Partep	0.4618	0.8302	-0.3685
Mo-Limfor	Ar-Lepdec	0.3668	0.8159	-0.4491	Ar-Partep	Ar-Phesol	0.5059	0.8310	-0.3252
Mo-Limfor	Ar-Neopin	0.3840	0.8174	-0.4333	Ar-Limcal	Ar-Tutabs	0.4822	0.8312	-0.3490
Ar-Acypis	Ar-Tutabs	0.3300	0.8183	-0.4883	Ar-Eufmex	Ar-Neopin	0.2750	0.8313	-0.5563
Ar-Manjur	Ar-Opebru	0.1823	0.8184	-0.6361	Ar-Eufmex	Ar-Manjur	0.5133	0.8316	-0.3184
Ar-Galmel	Ar-Pluxyl	0.2024	0.8185	-0.6161	St-Phycam	St-Physyr	0.1118	0.8317	-0.7198
Mo-Limfor	Ar-Epacla	0.3889	0.8188	-0.4299	St-Aphinv	St-Physyr	0.3224	0.8320	-0.5096
Ar-Eufmex	Ar-Sipfla	0.3214	0.8188	-0.4974	Ar-Adohon	Ar-Lephet	0.5053	0.8323	-0.3270
Mo-Halrub	Ar-Bomman	0.4043	0.8193	-0.4150	Ar-Calmac	Ar-Phesol	0.5702	0.8326	-0.2624
Ar-Bomman	Ar-Partep	0.3575	0.8193	-0.4618	Ar-Aulsol	Ar-Tutabs	0.5386	0.8339	-0.2952
Ar-Neopin	Ar-Stedum	0.3098	0.8193	-0.5095	Ar-Censcu	Ar-Manjur	0.6502	0.8340	-0.1838
Ar-Calmac	Ar-Eufmex	0.3119	0.8202	-0.5082	Ar-Stedum	Ar-Tutabs	0.5978	0.8341	-0.2364
Mo-Mermer	Ar-Sipfla	0.4165	0.8202	-0.4037	Mo-Halrub	Ar-Onttau	0.5249	0.8347	-0.3098
Ar-Eufmex	Ar-Onttau	0.3026	0.8203	-0.5177	Mo-Limfor	Ar-Tutabs	0.6471	0.8353	-0.1882
Ar-Lymdis	Ar-Tutabs	0.1736	0.8205	-0.6469	Ar-Erilan	Ar-Timtah	0.4613	0.8353	-0.3740
Ar-Censcu	Ar-Timcri	0.3943	0.8206	-0.4263	Ar-Erilan	Ar-Timbar	0.4577	0.8356	-0.3779
Mo-Mermer	Ar-Glocon	0.3824	0.8206	-0.4382	Mo-Mermer	Ar-Lepdec	0.5325	0.8361	-0.3036
Ar-Limcal	Ar-Phesol	0.3663	0.8209	-0.4546	Ar-Manjur	Ar-Phesol	0.6076	0.8362	-0.2285
Ar-Galmel	Ar-Lymdis	0.1906	0.8209	-0.6303	Ar-Galmel	Ar-Stedum	0.5988	0.8366	-0.2378
Ar-Censcu	Ar-Timpop	0.3854	0.8209	-0.4355	Mo-Halrub	Ar-Censcu	0.4829	0.8367	-0.3539
Ar-Neopin	Ar-Pluxyl	0.2687	0.8210	-0.5523	Ar-Onttau	Ar-Stedum	0.5262	0.8369	-0.3107
Ar-Adohon	Ar-Sitmis	0.3296	0.8214	-0.4918	Ar-Galmel	Ar-Homvit	0.5037	0.8370	-0.3333
Ar-Adohon	Ar-Lephet	0.3020	0.8215	-0.5196	Ar-Neopin	Ar-Pluxyl	0.4448	0.8382	-0.3934
Ar-Ampamp	Ec-Ophspi	0.3713	0.8218	-0.4505	Ar-Schgra	Ar-Tutabs	0.6043	0.8386	-0.2343
Mo-Limfor	Ar-Nillug	0.3929	0.8223	-0.4294	Ar-Censcu	Ar-Tutabs	0.5752	0.8393	-0.2641
Ar-Sipfla	Ar-Tutabs	0.3509	0.8226	-0.4717	Ar-Eufmex	Ar-Stemim	0.4183	0.8396	-0.4213
Ar-Danmel	Ar-Locmig	0.2980	0.8227	-0.5247	Ar-Bomman	Ar-Epacla	0.2397	0.8400	-0.6002
Ar-Amytra	Ar-Onttau	0.2882	0.8228	-0.5346	Ar-Amytra	Ar-Censcu	0.5828	0.8401	-0.2573
Ar-Calmac	Ar-Phesol	0.3099	0.8229	-0.5130	Mo-Halrub	Ar-Calmac	0.4669	0.8405	-0.3736
Ar-Eupuro	Ar-Lephet	0.2375	0.8233	-0.5858	Ar-Censcu	Ar-Phesol	0.4790	0.8405	-0.3616
Ar-Lymdis	Ar-Sitmis	0.3044	0.8237	-0.5193	Mo-Mermer	Ar-Galmel	0.5884	0.8406	-0.2522
Ar-Manjur	Ar-Sitmis	0.3027	0.8238	-0.5211	Ar-Limcal	Ar-Phesol	0.5227	0.8407	-0.3180
Mo-Limfor	Ar-Tutabs	0.3863	0.8243	-0.4381	Mo-Mermer	Ar-Timshe	0.5187	0.8408	-0.3220
Ar-Galmel	Ar-Sogfur	0.2955	0.8246	-0.5292	Ar-Anogla	Ar-Aulsol	0.4812	0.8408	-0.3596
Ar-Erilan	Ar-Timgen	0.3234	0.8250	-0.5016	Ar-Adohon	Ar-Tutabs	0.1730	0.8412	-0.6682

Ar-Lymdis	Ar-Phesol	0.3304	0.8252	-0.4948	Mo-Halrub	Ar-Aulsol	0.5437	0.8412	-0.2976
Ar-Censcu	Ar-Manjur	0.3837	0.8258	-0.4421	Ar-Aulsol	Ar-Epacla	0.4959	0.8412	-0.3454
Mo-Limfor	Ar-Helzea	0.3893	0.8266	-0.4373	Mo-Halrub	Ec-Ophspi	0.4392	0.8414	-0.4022
Mo-Halrub	Ar-Neopin	0.4482	0.8267	-0.3786	Mo-Mermer	Ar-Limcal	0.5039	0.8415	-0.3376
Ar-Glocon	Ar-Opebru	0.2214	0.8270	-0.6056	Mo-Mermer	Ar-Timbar	0.5308	0.8418	-0.3109
Ar-Galmel	Ar-Phesol	0.3248	0.8278	-0.5030	Mo-Mermer	Ar-Timtah	0.5330	0.8418	-0.3088
Ar-Calmac	Ec-Ophspi	0.3935	0.8280	-0.4345	Ar-Stemim	Ec-Ophspi	0.4939	0.8422	-0.3483
St-Aphinv	St-Physyr	0.2405	0.8281	-0.5876	Mo-Mermer	Ar-Glocon	0.6149	0.8423	-0.2273
Ar-Chisup	Ar-Galmel	0.2140	0.8293	-0.6153	Ar-Censcu	Ar-Homvit	0.4702	0.8425	-0.3723
Ar-Amytra	Ar-Chisup	0.1802	0.8295	-0.6493	Mo-Limfor	Mo-Mermer	0.4545	0.8429	-0.3884
Mo-Limfor	Ar-Danmel	0.4143	0.8297	-0.4154	Ar-Erilan	Ar-Timmon	0.4589	0.8434	-0.3845
Mo-Mermer	Ar-Vantam	0.3971	0.8300	-0.4329	Ar-Lephet	Ar-Locmig	0.4302	0.8444	-0.4142
Mo-Limfor	Mo-Mermer	0.2957	0.8300	-0.5343	Mo-Mermer	Ar-Timpop	0.5187	0.8445	-0.3259
Ar-Homvit	Ar-Locmig	0.2957	0.8301	-0.5343	Ar-Erilan	Ar-Timcri	0.4571	0.8449	-0.3878
Mo-Mermer	Ar-Schgra	0.4027	0.8305	-0.4279	Ar-Galmel	Ar-Stemim	0.5534	0.8449	-0.2916
Ar-Epacla	Ar-Stemim	0.3522	0.8305	-0.4784	Ar-Neopin	Ar-Stedum	0.5077	0.8451	-0.3374
Ar-Opebru	Ar-Stedum	0.3711	0.8307	-0.4597	Mo-Limfor	Ar-Temlon	0.5528	0.8452	-0.2924
Ar-Eufmex	Ar-Neopin	0.2713	0.8313	-0.5600	Ar-Erilan	Ar-Timpop	0.4535	0.8453	-0.3918
Ar-Galmel	Ar-Partep	0.3556	0.8317	-0.4761	Mo-Limfor	Ar-Homvit	0.5059	0.8454	-0.3395
Ar-Anogla	Ar-Eupann	0.2895	0.8322	-0.5427	Ar-Galmel	Ar-Glocon	0.3706	0.8458	-0.4751
Ar-Galmel	Ar-Limcal	0.3189	0.8322	-0.5133	Ar-Bomman	Ar-Onttau	0.5097	0.8458	-0.3361
Mo-Limfor	Ar-Helarm	0.3894	0.8322	-0.4428	Mo-Mermer	Ar-Timmon	0.5150	0.8459	-0.3310
Ar-Bomman	Ar-Stemim	0.3429	0.8322	-0.4894	Ar-Ladful	Ar-Onttau	0.4301	0.8460	-0.4159
Ar-Neopin	Ar-Nillug	0.2780	0.8323	-0.5544	Mo-Mermer	Ar-Timgen	0.5343	0.8464	-0.3121
Ar-Onttau	Ar-Stedum	0.3322	0.8324	-0.5002	Ar-Eufmex	Ar-Lepdec	0.4971	0.8470	-0.3499
Ar-Nillug	Ar-Tutabs	0.2327	0.8324	-0.5997	Ar-Schgra	Ec-Ophspi	0.5810	0.8470	-0.2660
Ar-Neopin	Ar-Partep	0.3507	0.8325	-0.4818	Ar-Bomman	Ar-Stemim	0.5274	0.8472	-0.3198
Ar-Phesol	Ar-Tutabs	0.3786	0.8326	-0.4540	Ar-Anogla	Ar-Phesol	0.5745	0.8475	-0.2730
Ar-Sipfla	Ec-Ophspi	0.3793	0.8326	-0.4533	Ar-Adohon	Ar-Eufmex	0.5022	0.8475	-0.3453
Ar-Helzea	Ar-Trivap	0.4088	0.8327	-0.4239	Mo-Mermer	Ar-Timcri	0.5149	0.8478	-0.3330
Ar-Eufmex	Ar-Phesol	0.2950	0.8332	-0.5382	Ar-Bomman	Ar-Pluxyl	0.2639	0.8479	-0.5841
Mo-Limfor	Ar-Glocon	0.4038	0.8341	-0.4302	St-Aphinv	St-Aphste	0.1221	0.8482	-0.7261
Ar-Bomman	Ar-Opebru	0.1991	0.8344	-0.6353	Ar-Pluxyl	Ar-Stedum	0.5839	0.8483	-0.2644
Ar-Amytra	Ar-Censcu	0.3648	0.8346	-0.4698	Ar-Amytra	Ar-Lepdec	0.4962	0.8483	-0.3521
Ar-Homvit	Ec-Ophspi	0.3606	0.8347	-0.4741	Ar-Helarm	Ar-Tutabs	0.2768	0.8484	-0.5716
Mo-Limfor	Ar-Phesol	0.3698	0.8349	-0.4651	Ar-Sogfur	Ec-Ophspi	0.5329	0.8486	-0.3156
Mo-Batpla	Ar-Sitmis	0.3927	0.8349	-0.4422	Mo-Halrub	Ar-Amytra	0.5091	0.8487	-0.3396
Ar-Phesol	Ar-Pseelo	0.3345	0.8350	-0.5005	Mo-Limfor	Ar-Phesol	0.5935	0.8490	-0.2555
Ar-Eufmex	Ar-Manjur	0.3280	0.8350	-0.5070	Mo-Mermer	Ar-Lephet	0.5498	0.8491	-0.2993
Mo-Mermer	Ar-Manjur	0.4069	0.8350	-0.4281	Ar-Lephet	Ar-Manjur	0.5418	0.8493	-0.3075
Ar-Lephet	Ar-Lymdis	0.3112	0.8352	-0.5240	Ar-Aulsol	Ar-Pluxyl	0.5200	0.8495	-0.3296
Ar-Adohon	Ar-Galmel	0.2033	0.8352	-0.6319	Mo-Limfor	Ar-Acypis	0.5934	0.8496	-0.2562
Ar-Manjur	Ar-Phesol	0.3561	0.8352	-0.4791	Mo-Limfor	Ar-Adohon	0.6270	0.8497	-0.2227
Ar-Galmel	Ar-Sitmis	0.3103	0.8360	-0.5257	Mo-Halrub	Ar-Galmel	0.5163	0.8497	-0.3334
Ar-Censcu	Ec-Ophspi	0.4144	0.8362	-0.4218	Ar-Epacla	Ar-Phesol	0.5916	0.8498	-0.2582
Ar-Amytra	Ar-Lephet	0.2795	0.8368	-0.5573	Ar-Amytra	Ar-Pluxyl	0.2156	0.8499	-0.6342
Ar-Censcu	Ar-Homvit	0.3615	0.8368	-0.4753	Ar-Eufmex	Ar-Sogfur	0.3838	0.8500	-0.4663
Ar-Homvit	Ar-Sitmis	0.3013	0.8373	-0.5360	Mo-Batpla	Mo-Halrub	0.3660	0.8501	-0.4841
Mo-Limfor	Ar-Pseelo	0.3943	0.8380	-0.4437	Ar-Locmig	Ar-Thrpal	0.3923	0.8501	-0.4579
Ar-Onttau	Ar-Sitmis	0.3167	0.8383	-0.5217	Ar-Epacla	Ar-Manjur	0.2868	0.8504	-0.5636
Ar-Helarm	Ar-Locmig	0.2909	0.8386	-0.5477	Ar-Erilan	Ar-Timshe	0.4589	0.8506	-0.3917
Ar-Sitmis	Ar-Sogfur	0.3064	0.8387	-0.5322	Ar-Eufmex	Ar-Pluxyl	0.5336	0.8506	-0.3170
Ar-Erilan	Ar-Stemim	0.3353	0.8396	-0.5043	Ar-Neopin	Ar-Stemim	0.4389	0.8507	-0.4118
Ar-Lephet	Ar-Manjur	0.3004	0.8396	-0.5392	Ar-Lepdec	Ar-Timgen	0.3701	0.8514	-0.4813
Ar-Lepdec	Ar-Timgen	0.3333	0.8400	-0.5067	Ar-Erilan	Ar-Stemim	0.4440	0.8517	-0.4077
Ar-Lepdec	Ar-Sitmis	0.2928	0.8403	-0.5475	Ar-Lephet	Ar-Lymdis	0.4649	0.8518	-0.3868
Ar-Acypis	Ar-Chisup	0.3147	0.8404	-0.5256	Ar-Acypis	Ar-Pluxyl	0.6658	0.8520	-0.1861
Mo-Halrub	Ar-Censcu	0.4278	0.8405	-0.4127	Ar-Lymdis	Ar-Thrpal	0.4193	0.8522	-0.4329
Ar-Locmig	Ec-Ophspi	0.3909	0.8406	-0.4497	Ar-Anogla	Ar-Osmbic	0.5161	0.8527	-0.3366

Ar-Stedum	Ar-Tutabs	0.3665	0.8407	-0.4742	Ar-Pluxyl	Ar-Tutabs	0.1468	0.8529	-0.7061
Ar-Epacla	Ar-Locmig	0.2713	0.8415	-0.5702	Ar-Manjur	Ar-Tutabs	0.2569	0.8529	-0.5960
Mo-Halrub	Ar-Onttau	0.4465	0.8419	-0.3954	Mo-Halrub	Ar-Locmig	0.4885	0.8530	-0.3645
Ar-Adohon	Ar-Tutabs	0.1253	0.8420	-0.7167	Ar-Adohon	Ar-Galmel	0.2080	0.8534	-0.6455
Ar-Erilan	Ar-Timcri	0.3262	0.8423	-0.5161	Ar-Temlon	Ec-Ophspi	0.5418	0.8538	-0.3120
Mo-Halrub	Ar-Calmac	0.4336	0.8423	-0.4087	Ar-Epacla	Ar-Limcal	0.4719	0.8542	-0.3822
Ar-Eufmex	Ar-Sitmis	0.3323	0.8425	-0.5102	Ar-Locmig	Ec-Ophspi	0.5266	0.8543	-0.3278
Ar-Erilan	Ar-Timpop	0.3169	0.8427	-0.5257	Ar-Acypis	Ar-Epacla	0.5536	0.8546	-0.3011
Ar-Lepdec	Ar-Temlon	0.3411	0.8429	-0.5018	Ar-Lepdec	Ar-Temlon	0.4479	0.8550	-0.4071
Ar-Epacla	Ar-Limcal	0.3059	0.8432	-0.5373	Ar-Galmel	Ar-Manjur	0.2966	0.8551	-0.5585
Mo-Mermer	Ar-Lepdec	0.3725	0.8432	-0.4708	Ar-Stemim	Ar-Temlon	0.4488	0.8552	-0.4064
Ar-Lymdis	Ar-Thrpal	0.2896	0.8433	-0.5537	Mo-Halrub	Ar-Anogla	0.4451	0.8553	-0.4102
Ar-Bomman	Ar-Pluxyl	0.1819	0.8436	-0.6617	Ar-Tutabs	Ec-Ophspi	0.6150	0.8556	-0.2406
Ar-Danmel	Ar-Tutabs	0.1625	0.8438	-0.6813	Mo-Mermer	Ar-Anogla	0.5269	0.8557	-0.3288
Mo-Mermer	Ar-Galmel	0.3924	0.8439	-0.4515	Ar-Adohon	Ar-Anogla	0.4804	0.8558	-0.3753
Ar-Epacla	Ar-Phesol	0.3309	0.8440	-0.5130	Ar-Adohon	Ar-Trivap	0.6015	0.8562	-0.2546
Ar-Galmel	Ar-Stedum	0.3462	0.8441	-0.4979	Ar-Amytra	Ar-Manjur	0.2854	0.8563	-0.5709
Ar-Eufmex	Ar-Stemim	0.3526	0.8443	-0.4917	Ar-Galmel	Ar-Onttau	0.5284	0.8563	-0.3279
Mo-Limfor	Ar-Adohon	0.3840	0.8446	-0.4605	Ar-Anogla	Ar-Eupann	0.3954	0.8564	-0.4610
Ar-Epacla	Ar-Glocon	0.2211	0.8448	-0.6237	Ar-Nillug	Ar-Pluxyl	0.4738	0.8571	-0.3833
Ar-Eufmex	Ar-Pluxyl	0.3595	0.8451	-0.4856	Mo-Batpla	Ar-Manjur	0.6940	0.8575	-0.1635
Ar-Adohon	Ar-Eufmex	0.3760	0.8453	-0.4693	Ar-Limcal	Ar-Nillug	0.4945	0.8578	-0.3633
Ar-Temlon	Ec-Ophspi	0.4018	0.8458	-0.4439	Ar-Anogla	Ar-Manjur	0.4769	0.8579	-0.3810
Ar-Danmel	Ar-Galmel	0.1968	0.8460	-0.6492	Ar-Glocon	Ar-Tutabs	0.3304	0.8580	-0.5276
St-Phycam	St-Physyr	0.0483	0.8460	-0.7977	Ar-Glocon	Ar-Lephet	0.5551	0.8587	-0.3036
Mo-Limfor	Ar-Homvit	0.3778	0.8464	-0.4686	Mo-Batpla	Ar-Timbar	0.5854	0.8594	-0.2740
Ar-Pluxyl	Ar-Stedum	0.3542	0.8466	-0.4924	Mo-Batpla	Ar-Timtah	0.5831	0.8594	-0.2763
Ar-Anogla	Ar-Eupuro	0.2918	0.8468	-0.5550	Ar-Pluxyl	Ar-Schgra	0.6366	0.8598	-0.2233
Ar-Galmel	Ar-Homvit	0.2960	0.8472	-0.5512	Mo-Limfor	Ar-Timmon	0.5183	0.8600	-0.3416
Ar-Limcal	Ar-Tutabs	0.3057	0.8472	-0.5416	Mo-Limfor	Ar-Timpop	0.5271	0.8600	-0.3329
Ar-Lephet	Ar-Locmig	0.2940	0.8474	-0.5534	Mo-Halrub	Ar-Stedum	0.4754	0.8600	-0.3846
Ar-Opebru	Ar-Phesol	0.3943	0.8474	-0.4531	Ar-Manjur	Ar-Neopin	0.4774	0.8601	-0.3828
Ar-Censcu	Ar-Phesol	0.3390	0.8475	-0.5085	Mo-Mermer	Ar-Stemim	0.4111	0.8602	-0.4491
Ar-Anogla	Ar-Phesol	0.3328	0.8477	-0.5150	Mo-Limfor	Ar-Timshe	0.5291	0.8606	-0.3315
Ar-Anogla	Ar-Pluxyl	0.3372	0.8478	-0.5106	Ar-Homvit	Ar-Lephet	0.4220	0.8606	-0.4385
Ar-Glocon	Ar-Lephet	0.3068	0.8483	-0.5415	Ar-Epacla	Ar-Sipfla	0.5534	0.8606	-0.3072
Ar-Amytra	Ec-Ophspi	0.3817	0.8484	-0.4666	Ar-Lephet	Ar-Phesol	0.5097	0.8607	-0.3510
Mo-Batpla	An-Hydele	0.4096	0.8485	-0.4390	Ar-Adohon	Ar-Thrpal	0.4434	0.8609	-0.4175
Ar-Ladful	Ar-Onttau	0.3077	0.8486	-0.5410	Ar-Epacla	Ar-Glocon	0.3320	0.8612	-0.5292
Ar-Opebru	Ar-Partep	0.3684	0.8487	-0.4802	Ar-Lepdec	Ar-Phesol	0.5567	0.8613	-0.3046
Ar-Helzea	Ar-Locmig	0.2907	0.8488	-0.5581	Ar-Acypis	Ar-Lephet	0.4270	0.8614	-0.4344
Ar-Epacla	Ar-Schgra	0.3303	0.8495	-0.5191	Mo-Mermer	Ar-Locmig	0.5175	0.8622	-0.3448
Mo-Halrub	Ar-Locmig	0.4224	0.8502	-0.4277	Ar-Temlon	Ar-Timgen	0.3883	0.8630	-0.4747
Ar-Limcal	Ar-Sitmis	0.3352	0.8509	-0.5157	Ar-Anogla	Ar-Nillug	0.4720	0.8630	-0.3910
Mo-Limfor	Ar-Temlon	0.4240	0.8515	-0.4275	Ar-Lepdec	Ar-Tutabs	0.4543	0.8630	-0.4087
Ar-Amytra	Ar-Sitmis	0.3102	0.8528	-0.5427	Ar-Acypis	Ar-Manjur	0.5952	0.8634	-0.2681
Ar-Amytra	Ar-Vantam	0.1827	0.8529	-0.6701	Mo-Batpla	Ar-Timmon	0.5793	0.8636	-0.2843
Mo-Mermer	Ar-Timgen	0.3977	0.8531	-0.4554	Ar-Eufmex	Ar-Lymdis	0.4354	0.8637	-0.4282
Ar-Amytra	Ar-Lepdec	0.2653	0.8532	-0.5879	Ar-Limcal	Ar-Locmig	0.4284	0.8638	-0.4354
Ar-Sitmis	Ar-Stedum	0.3344	0.8532	-0.5189	Mo-Limfor	Ar-Bomman	0.5777	0.8641	-0.2864
Ar-Epacla	Ar-Manjur	0.1818	0.8535	-0.6717	Mo-Batpla	Ar-Timgen	0.5821	0.8641	-0.2820
Mo-Halrub	Ec-Ophspi	0.3883	0.8541	-0.4658	Ar-Anogla	Ar-Pluxyl	0.5042	0.8642	-0.3601
Ar-Trivap	Ec-Ophspi	0.4286	0.8541	-0.4255	Mo-Limfor	Ar-Timcri	0.5193	0.8644	-0.3451
Mo-Halrub	Ar-Opebru	0.3960	0.8543	-0.4583	Ar-Helarm	Ar-Neopin	0.4949	0.8646	-0.3696
Mo-Batpla	Ar-Locmig	0.3978	0.8544	-0.4566	Mo-Mermer	Ar-Ladful	0.5459	0.8647	-0.3188
Ar-Amytra	Ar-Limcal	0.2995	0.8546	-0.5551	Mo-Batpla	Ar-Tutabs	0.6976	0.8649	-0.1673
Ar-Adohon	Ar-Anogla	0.3422	0.8549	-0.5127	Ar-Aulsol	Ar-Eupann	0.3697	0.8649	-0.4952
Ar-Amytra	Ar-Manjur	0.1681	0.8549	-0.6868	Ar-Aulsol	Ar-Manjur	0.5919	0.8652	-0.2733
Ar-Adohon	Ar-Trivap	0.4567	0.8550	-0.3982	Ar-Locmig	Ar-Phesol	0.4775	0.8655	-0.3880

Ar-Stemim	Ar-Temlon	0.3850	0.8551	-0.4701	Ar-Onttau	Ec-Ophspi	0.5786	0.8658	-0.2872
Ar-Censcu	Ar-Tutabs	0.3929	0.8551	-0.4622	Mo-Batpla	Ar-Timpop	0.5768	0.8659	-0.2891
Ar-Lephet	Ar-Phesol	0.2938	0.8552	-0.5614	Mo-Limfor	Ar-Timbar	0.5234	0.8660	-0.3427
Ar-Acypis	Ar-Manjur	0.3054	0.8553	-0.5499	Mo-Limfor	Ar-Timtah	0.5256	0.8660	-0.3404
Ar-Galmel	Ar-Glocon	0.2655	0.8557	-0.5902	Mo-Batpla	Ar-Timcri	0.5811	0.8664	-0.2853
Ar-Acypis	Ec-Ophspi	0.3784	0.8558	-0.4774	Mo-Limfor	Ar-Osmbic	0.5456	0.8666	-0.3210
Mo-Mermer	Ar-Timpop	0.3832	0.8559	-0.4728	Mo-Batpla	Ar-Timshe	0.5772	0.8669	-0.2897
Mo-Halrub	Ar-Amytra	0.4185	0.8561	-0.4375	Ar-Calmac	Ar-Temlon	0.4651	0.8671	-0.4020
Ar-Anogla	Ar-Locmig	0.2940	0.8562	-0.5622	Mo-Limfor	Ar-Schgra	0.6005	0.8673	-0.2668
Ar-Amytra	Ar-Neopin	0.2843	0.8563	-0.5719	Mo-Limfor	Ar-Sipfla	0.6043	0.8678	-0.2635
Ar-Glocon	Ar-Tutabs	0.2148	0.8566	-0.6418	Ar-Amytra	Ar-Neopin	0.4502	0.8680	-0.4178
Mo-Limfor	Ar-Acypis	0.3935	0.8574	-0.4639	Ar-Epacla	Ar-Sogfur	0.4327	0.8685	-0.4357
An-Hydele	Ec-Ophspi	0.3643	0.8576	-0.4933	Ar-Anogla	Ar-Eupuro	0.3915	0.8685	-0.4770
Mo-Mermer	Ar-Limcal	0.4242	0.8577	-0.4335	Mo-Batpla	Ar-Acypis	0.5488	0.8686	-0.3198
Ar-Homvit	Ar-Pseelo	0.3064	0.8578	-0.5513	Ar-Amytra	Ar-Homvit	0.4914	0.8686	-0.3772
Mo-Mermer	Ar-Lephet	0.3853	0.8580	-0.4727	Ar-Aulsol	Ar-Locmig	0.3487	0.8687	-0.5200
Ar-Eufmex	Ar-Lepdec	0.3168	0.8581	-0.5412	Ar-Phesol	Ar-Sogfur	0.4481	0.8690	-0.4209
Ar-Locmig	Ar-Phesol	0.2930	0.8584	-0.5654	Ar-Amytra	Ar-Limcal	0.5100	0.8691	-0.3591
Ar-Pluxyl	Ar-Schgra	0.3317	0.8585	-0.5269	Ar-Adohon	Ar-Amytra	0.2275	0.8694	-0.6419
St-Aphinv	St-Aphste	0.1146	0.8586	-0.7440	Mo-Limfor	Ar-Aulsol	0.4826	0.8694	-0.3868
Ar-Anogla	Ar-Manjur	0.3147	0.8589	-0.5442	Mo-Limfor	Ar-Timgen	0.5311	0.8696	-0.3384
Ar-Neopin	Ar-Sitmis	0.2995	0.8590	-0.5595	Mo-Mermer	Ar-Sogfur	0.5097	0.8705	-0.3608
Mo-Mermer	Ar-Timcri	0.3933	0.8593	-0.4660	Ar-Homvit	Ar-Nillug	0.3237	0.8708	-0.5471
Ar-Limcal	Ar-Locmig	0.3269	0.8601	-0.5332	Ar-Galmel	Ar-Helarm	0.3175	0.8708	-0.5533
Ar-Tutabs	Ec-Ophspi	0.3522	0.8604	-0.5081	Mo-Batpla	Ar-Locmig	0.5165	0.8711	-0.3546
Ar-Censcu	Ar-Chisup	0.3819	0.8604	-0.4785	Mo-Halrub	Ar-Schgra	0.5357	0.8720	-0.3364
Mo-Mermer	Ar-Danmel	0.4120	0.8604	-0.4485	Ar-Locmig	Ar-Temlon	0.3984	0.8722	-0.4738
Ar-Galmel	Ar-Onttau	0.2919	0.8606	-0.5687	Ar-Epacla	Ar-Lephet	0.4644	0.8722	-0.4078
Ar-Epacla	Ar-Sogfur	0.2498	0.8606	-0.6108	Ar-Anogla	Ar-Sogfur	0.4296	0.8726	-0.4429
Mo-Limfor	Ar-Timpop	0.3882	0.8607	-0.4725	Ar-Manjur	Ar-Schgra	0.6127	0.8731	-0.2604
Mo-Halrub	Ar-Nillug	0.3799	0.8607	-0.4808	Ar-Galmel	Ar-Nillug	0.4985	0.8732	-0.3747
Ar-Osmbic	Ec-Ophspi	0.3958	0.8609	-0.4650	Ar-Homvit	Ar-Thrpal	0.3917	0.8733	-0.4816
Ar-Calmac	Ar-Sitmis	0.3229	0.8609	-0.5380	Mo-Halrub	Ar-Stemim	0.4265	0.8740	-0.4475
Mo-Halrub	Ar-Stedum	0.4008	0.8609	-0.4602	Ar-Aulsol	Ar-Sogfur	0.3806	0.8740	-0.4934
An-Hydele	Ar-Amytra	0.3570	0.8614	-0.5043	Ar-Osmbic	Ec-Ophspi	0.5590	0.8741	-0.3151
Mo-Limfor	Ar-Timgen	0.3823	0.8618	-0.4795	Ar-Lephet	Ar-Sipfla	0.4381	0.8744	-0.4363
Ar-Schgra	Ar-Tutabs	0.3411	0.8619	-0.5208	Ar-Partep	Ar-Pluxyl	0.5614	0.8746	-0.3133
Ar-Acypis	Ar-Pluxyl	0.3103	0.8619	-0.5515	Ar-Censcu	Ar-Schgra	0.5193	0.8747	-0.3554
Ar-Onttau	Ec-Ophspi	0.4173	0.8619	-0.4446	Ar-Locmig	Ar-Nillug	0.3586	0.8752	-0.5165
Ar-Pseelo	Ar-Tutabs	0.3324	0.8620	-0.5296	Mo-Batpla	Ar-Schgra	0.5492	0.8752	-0.3260
Ar-Eupann	Ar-Lephet	0.2456	0.8621	-0.6165	Mo-Batpla	Ar-Temlon	0.5570	0.8753	-0.3183
Ar-Stemim	Ec-Ophspi	0.3764	0.8622	-0.4858	Ar-Helarm	Ar-Phesol	0.6190	0.8753	-0.2563
Ar-Galmel	Ar-Helarm	0.1733	0.8627	-0.6894	Ar-Eufmex	Ar-Thrpal	0.4931	0.8760	-0.3829
Ar-Galmel	Ar-Helzea	0.1728	0.8627	-0.6898	Ar-Phesol	Ar-Trivap	0.4437	0.8770	-0.4333
Ar-Epacla	Ar-Vantam	0.1785	0.8628	-0.6842	Ar-Anogla	Ar-Homvit	0.4550	0.8773	-0.4223
Ar-Neopin	Ar-Stemim	0.3217	0.8628	-0.5411	Ar-Aulsol	Ar-Neopin	0.3607	0.8774	-0.5167
Mo-Limfor	Ar-Bomman	0.3646	0.8628	-0.4983	Ar-Acypis	Ar-Stedum	0.5444	0.8774	-0.3330
Ar-Bomman	Ar-Onttau	0.3145	0.8629	-0.5485	Ar-Limcal	Ar-Partep	0.5264	0.8776	-0.3512
Ar-Lepdec	Ar-Phesol	0.2922	0.8632	-0.5710	Ar-Lephet	Ar-Nillug	0.3964	0.8776	-0.4811
Ar-Helarm	Ar-Tutabs	0.1172	0.8632	-0.7460	Mo-Mermer	Ar-Aulsol	0.4748	0.8779	-0.4030
Ar-Helzea	Ar-Tutabs	0.1201	0.8632	-0.7431	Mo-Limfor	Ar-Aptruf	0.5690	0.8784	-0.3094
Mo-Batpla	Ar-Chisup	0.4022	0.8633	-0.4611	Ar-Galmel	Ar-Ladful	0.5033	0.8784	-0.3751
Mo-Batpla	Ar-Acypis	0.3824	0.8634	-0.4810	Mo-Limfor	Ar-Anogla	0.5404	0.8785	-0.3382
Ar-Locmig	Ar-Sitmis	0.3002	0.8637	-0.5636	Ar-Aulsol	Ar-Eufmex	0.3348	0.8787	-0.5438
Mo-Batpla	Mo-Halrub	0.3614	0.8639	-0.5024	Ar-Amytra	Ar-Glocon	0.3666	0.8788	-0.5122
Ar-Calmac	Ar-Temlon	0.3298	0.8641	-0.5343	Ar-Amytra	Ar-Sipfla	0.6440	0.8789	-0.2349
Ar-Sitmis	Ar-Trivap	0.3997	0.8645	-0.4648	Ar-Calmac	Ec-Ophspi	0.5545	0.8790	-0.3245
Ar-Homvit	Ar-Lephet	0.2958	0.8645	-0.5688	Mo-Limfor	Ar-Erilan	0.6064	0.8791	-0.2727
Ar-Partep	Ar-Phesol	0.3559	0.8647	-0.5088	Ar-Adohon	Ar-Epacla	0.1843	0.8791	-0.6948

Mo-Mermer	Ar-Anogla	0.4127	0.8649	-0.4521	Ar-Erilan	Ar-Sipfla	0.1243	0.8795	-0.7552
Ar-Manjur	Ar-Neopin	0.2809	0.8651	-0.5842	Ar-Lepdec	Ar-Osmbic	0.4991	0.8798	-0.3806
Mo-Limfor	Ar-Timcri	0.3825	0.8652	-0.4826	Ar-Aulsol	Ar-Lymdis	0.4585	0.8800	-0.4215
Ar-Pluxyl	Ar-Tutabs	0.1343	0.8656	-0.7314	Mo-Batpla	Ar-Lymdis	0.6279	0.8804	-0.2526
Mo-Batpla	Ar-Timgen	0.4152	0.8657	-0.4504	Mo-Batpla	Mo-Mermer	0.3111	0.8804	-0.5693
Ar-Censcu	Ar-Opebru	0.4095	0.8662	-0.4568	Ar-Limcal	Ar-Lymdis	0.4590	0.8806	-0.4216
Ar-Opebru	Ar-Schgra	0.3491	0.8663	-0.5172	Ar-Censcu	Ar-Helarm	0.6133	0.8807	-0.2674
Ar-Manjur	Ar-Tutabs	0.1792	0.8664	-0.6872	Ar-Bomman	Ar-Nillug	0.4931	0.8807	-0.3876
Ar-Temlon	Ar-Timgen	0.3499	0.8667	-0.5168	Ar-Homvit	Ar-Limcal	0.4857	0.8808	-0.3951
Ar-Calmac	Ar-Opebru	0.3129	0.8668	-0.5539	Mo-Batpla	Ar-Epacla	0.6757	0.8808	-0.2051
Ar-Eufmex	Ar-Thrpal	0.3824	0.8668	-0.4844	Ar-Aptruf	Ar-Timgen	0.3694	0.8809	-0.5115
Ar-Sitmis	Ar-Timgen	0.3211	0.8669	-0.5458	Ar-Calmac	Ar-Timgen	0.4053	0.8814	-0.4761
Ar-Amytra	Ar-Pluxyl	0.1654	0.8672	-0.7018	Mo-Limfor	Ar-Thrpal	0.5580	0.8816	-0.3235
Mo-Mermer	Ar-Locmig	0.3684	0.8672	-0.4988	Mo-Batpla	Ar-Nillug	0.5773	0.8816	-0.3043
Ar-Galmel	Ar-Manjur	0.1846	0.8673	-0.6827	Mo-Limfor	Ar-Eufmex	0.5452	0.8816	-0.3365
Ar-Chisup	Ar-Neopin	0.2999	0.8674	-0.5674	Mo-Batpla	Ar-Sipfla	0.5520	0.8819	-0.3299
An-Hydele	Ar-Sitmis	0.4111	0.8674	-0.4563	Mo-Mermer	Ar-Eupuro	0.4984	0.8823	-0.3839
Ar-Aptruf	Ar-Timgen	0.3113	0.8683	-0.5569	Mo-Limfor	Ar-Stedum	0.5756	0.8825	-0.3069
Ar-Eufmex	Ar-Sogfur	0.3549	0.8685	-0.5135	Ar-Nillug	Ar-Sogfur	0.1038	0.8830	-0.7791
Ar-Acypis	Ar-Lephet	0.2817	0.8688	-0.5871	Ar-Onttau	Ar-Partep	0.4926	0.8832	-0.3906
Mo-Limfor	Ar-Erilan	0.3872	0.8689	-0.4817	Ar-Lymdis	Ar-Stedum	0.5275	0.8833	-0.3558
Ar-Helarm	Ar-Neopin	0.2626	0.8694	-0.6067	Mo-Mermer	Ar-Homvit	0.5030	0.8836	-0.3805
Ar-Lepdec	Ar-Opebru	0.3025	0.8694	-0.5668	Ar-Amytra	Ar-Anogla	0.4871	0.8837	-0.3966
Ar-Amytra	Ar-Pseelo	0.3265	0.8699	-0.5434	Mo-Halrub	Ar-Limcal	0.5186	0.8838	-0.3652
Ar-Partep	Ar-Pluxyl	0.3521	0.8700	-0.5179	Mo-Mermer	Ar-Eupann	0.5037	0.8840	-0.3804
Ar-Lepdec	Ar-Timcri	0.3282	0.8702	-0.5420	Ar-Locmig	Ar-Schgra	0.3976	0.8841	-0.4865
Ar-Lepdec	Ar-Timpop	0.3240	0.8706	-0.5466	Mo-Batpla	Ar-Lepdec	0.5361	0.8844	-0.3483
Ar-Eufmex	Ar-Lymdis	0.3273	0.8707	-0.5434	Ar-Glocon	Ar-Limcal	0.5145	0.8846	-0.3700
Ar-Chisup	Ar-Phesol	0.3467	0.8710	-0.5243	Mo-Halrub	Ar-Trivap	0.5303	0.8849	-0.3546
Mo-Batpla	Ar-Tutabs	0.3991	0.8719	-0.4728	Ar-Limcal	Ar-Manjur	0.5001	0.8851	-0.3850
Mo-Batpla	Ar-Timpop	0.4136	0.8722	-0.4586	Ar-Glocon	Ar-Trivap	0.6083	0.8855	-0.2772
Ar-Phesol	Ar-Trivap	0.4003	0.8722	-0.4719	Mo-Limfor	Ar-Amytra	0.6301	0.8860	-0.2558
Mo-Mermer	Ar-Stemim	0.3893	0.8723	-0.4830	Ar-Trivap	Ec-Ophspi	0.5668	0.8864	-0.3196
Ar-Galmel	Ar-Ladful	0.2988	0.8724	-0.5736	Ar-Eufmex	Ar-Homvit	0.4024	0.8867	-0.4843
Mo-Batpla	Ar-Manjur	0.3941	0.8726	-0.4785	Mo-Batpla	Ar-Partep	0.4497	0.8867	-0.4371
Ar-Acypis	Ar-Opebru	0.3481	0.8726	-0.5245	St-Aphste	St-Physyr	0.2792	0.8868	-0.6075
Ar-Epacla	Ar-Lephet	0.2971	0.8726	-0.5755	Ar-Adohon	Ar-Censcu	0.5784	0.8868	-0.3085
Ar-Pluxyl	Ar-Pseelo	0.3285	0.8727	-0.5441	Mo-Limfor	Ar-Stemim	0.4237	0.8869	-0.4632
Mo-Batpla	Ar-Timcri	0.4172	0.8727	-0.4555	Ar-Censcu	Ar-Pluxyl	0.5724	0.8869	-0.3145
An-Hydele	Ar-Epacla	0.3254	0.8728	-0.5474	Ar-Bomman	Ar-Helarm	0.3313	0.8872	-0.5559
Ar-Danmel	Ar-Opebru	0.1854	0.8729	-0.6875	Ar-Censcu	Ar-Partep	0.3358	0.8875	-0.5517
Ar-Stemim	Ar-Trivap	0.4218	0.8734	-0.4516	Ar-Homvit	Ar-Sipfla	0.4102	0.8881	-0.4779
Mo-Limfor	Ar-Eufmex	0.3986	0.8734	-0.4748	Ar-Partep	Ar-Sogfur	0.4709	0.8883	-0.4174
An-Hydele	Ar-Censcu	0.4706	0.8735	-0.4029	Mo-Batpla	Ar-Lephet	0.5806	0.8884	-0.3078
Ar-Amytra	Ar-Glocon	0.2485	0.8735	-0.6250	Ar-Censcu	Ar-Glocon	0.5878	0.8884	-0.3006
Mo-Limfor	Ar-Trivap	0.4291	0.8735	-0.4444	Mo-Halrub	Ar-Sogfur	0.4746	0.8885	-0.4139
Mo-Batpla	Mo-Mermer	0.3068	0.8740	-0.5672	Ar-Amytra	Ar-Erilan	0.5884	0.8887	-0.3003
Ar-Ladful	Ar-Vantam	0.3058	0.8740	-0.5682	Mo-Batpla	Ar-Limcal	0.5370	0.8889	-0.3519
Ar-Glocon	Ar-Pseelo	0.3466	0.8741	-0.5275	Mo-Batpla	Ar-Stemim	0.3991	0.8892	-0.4900
Ar-Glocon	Ar-Sitmis	0.3129	0.8743	-0.5613	Ar-Amytra	Ec-Ophspi	0.5579	0.8896	-0.3317
Ar-Locmig	Ar-Thrpal	0.3138	0.8746	-0.5608	Mo-Limfor	Ar-Locmig	0.4844	0.8896	-0.4052
Ar-Chisup	Ar-Sipfla	0.3264	0.8752	-0.5489	Mo-Limfor	Ar-Trivap	0.5725	0.8897	-0.3173
Ar-Censcu	Ar-Helarm	0.3988	0.8755	-0.4767	Ar-Lephet	Ar-Pluxyl	0.4802	0.8901	-0.4098
Ar-Galmel	Ar-Stemim	0.3430	0.8756	-0.5326	Ar-Neopin	Ar-Schgra	0.4177	0.8903	-0.4727
Ar-Phesol	Ar-Sogfur	0.3596	0.8758	-0.5162	Ar-Phesol	Ar-Temlon	0.5091	0.8908	-0.3817
Ar-Chisup	Ar-Stedum	0.3414	0.8759	-0.5345	Ar-Censcu	Ar-Stedum	0.4319	0.8909	-0.4590
Ar-Amytra	Ar-Anogla	0.2971	0.8761	-0.5790	Ar-Stemim	Ar-Trivap	0.5351	0.8910	-0.3559
Ar-Sitmis	Ar-Timcri	0.3170	0.8762	-0.5592	Ar-Acypis	Ar-Sogfur	0.4188	0.8911	-0.4723
Ar-Anogla	Ar-Osmbic	0.3254	0.8763	-0.5509	Ar-Calmac	Ar-Pluxyl	0.4822	0.8912	-0.4090

Mo-Limfor	Ar-Sipfla	0.3889	0.8765	-0.4876	Ar-Amytra	Ar-Stemim	0.5528	0.8912	-0.3384
Ar-Sitmis	Ar-Vantam	0.3105	0.8767	-0.5662	Ar-Acypis	Ar-Neopin	0.4169	0.8913	-0.4744
Ar-Sitmis	Ar-Timpop	0.3061	0.8767	-0.5707	Ar-Galmel	Ec-Ophspi	0.6093	0.8915	-0.2822
Mo-Halrub	Ar-Stemim	0.4320	0.8768	-0.4448	Ar-Onttau	Ar-Phesol	0.5280	0.8916	-0.3636
Ar-Lepdec	Ar-Osmbic	0.2833	0.8770	-0.5937	Ar-Manjur	Ar-Partep	0.5536	0.8917	-0.3382
Mo-Batpla	Ar-Schgra	0.3804	0.8773	-0.4969	Ar-Sogfur	Ar-Timgen	0.3317	0.8921	-0.5604
Ar-Locmig	Ar-Opebru	0.3136	0.8774	-0.5637	Mo-Batpla	Ar-Helarm	0.6817	0.8930	-0.2112
Mo-Batpla	Ar-Lymdis	0.4057	0.8775	-0.4718	Ar-Adohon	Ar-Ladful	0.5017	0.8933	-0.3916
Mo-Halrub	Ar-Anogla	0.4710	0.8775	-0.4065	Ar-Manjur	Ar-Nillug	0.5113	0.8934	-0.3820
Ar-Opebru	Ec-Ophspi	0.3600	0.8779	-0.5178	Ar-Lephet	Ar-Neopin	0.3180	0.8939	-0.5759
Ar-Anogla	Ar-Helarm	0.3287	0.8784	-0.5497	Ar-Aulsol	Ar-Stedum	0.4963	0.8944	-0.3981
Mo-Batpla	Ec-Ophspi	0.3788	0.8784	-0.4996	Ar-Eufmex	Ar-Timcri	0.4151	0.8944	-0.4793
Ar-Anogla	Ar-Helzea	0.3296	0.8786	-0.5491	Ar-Eufmex	Ar-Timbar	0.4201	0.8944	-0.4743
Ar-Amytra	Ar-Homvit	0.2954	0.8787	-0.5834	Ar-Eufmex	Ar-Timtah	0.4253	0.8944	-0.4691
Ar-Opebru	Ar-Sipfla	0.3508	0.8788	-0.5280	Mo-Batpla	Ar-Trivap	0.5348	0.8949	-0.3600
Ar-Adohon	Ar-Amytra	0.1706	0.8790	-0.7084	Ar-Aptruf	Ec-Ophspi	0.5566	0.8951	-0.3386
Ar-Locmig	Ar-Temlon	0.3202	0.8792	-0.5590	Ar-Censcu	Ar-Epacla	0.5416	0.8952	-0.3537
Mo-Mermer	Ar-Ladful	0.3819	0.8792	-0.4974	Ar-Lepdec	Ar-Locmig	0.4668	0.8960	-0.4292
Ar-Danmel	Ar-Lephet	0.2826	0.8793	-0.5967	Mo-Halrub	Ar-Timbar	0.4386	0.8961	-0.4576
Mo-Limfor	Ar-Stedum	0.3772	0.8795	-0.5022	Mo-Halrub	Ar-Timtah	0.4386	0.8961	-0.4576
Ar-Locmig	Ar-Pseelo	0.2879	0.8795	-0.5916	Ar-Schgra	Ar-Sogfur	0.4256	0.8967	-0.4711
Ar-Limcal	Ar-Partep	0.3932	0.8796	-0.4864	Mo-Mermer	Ar-Helarm	0.6296	0.8970	-0.2674
Ar-Erilan	Ar-Sipfla	0.1324	0.8797	-0.7473	Ar-Eufmex	Ar-Osmbic	0.1985	0.8977	-0.6992
Ar-Galmel	Ar-Vantam	0.1886	0.8797	-0.6911	Ar-Aulsol	Ar-Eupuro	0.3646	0.8978	-0.5332
Ar-Aptruf	Ec-Ophspi	0.3548	0.8799	-0.5251	Ar-Lymdis	Ar-Schgra	0.5141	0.8978	-0.3838
Ar-Censcu	Ar-Glocon	0.4239	0.8799	-0.4560	Ar-Anogla	Ar-Helarm	0.5659	0.8981	-0.3321
Mo-Mermer	Ar-Eupann	0.3858	0.8799	-0.4941	Ar-Galmel	Ar-Neopin	0.4394	0.8981	-0.4587
Mo-Limfor	Ar-Osmbic	0.3960	0.8801	-0.4840	Ar-Neopin	Ar-Sipfla	0.4137	0.8984	-0.4847
Ar-Danmel	Ar-Sitmis	0.3069	0.8802	-0.5733	Ar-Adohon	Ar-Aulsol	0.5176	0.8985	-0.3809
Mo-Limfor	Ar-Stemim	0.3696	0.8802	-0.5107	Ar-Acypis	Ar-Amytra	0.6412	0.8986	-0.2574
Ar-Nillug	Ar-Opebru	0.2513	0.8808	-0.6294	Ar-Censcu	Ar-Sipfla	0.4985	0.8990	-0.4005
Ar-Helzea	Ar-Neopin	0.2659	0.8808	-0.6149	Mo-Batpla	Ar-Sogfur	0.5467	0.8991	-0.3524
Ar-Helarm	Ar-Pseelo	0.3410	0.8813	-0.5403	Ar-Manjur	Ar-Sipfla	0.5872	0.8992	-0.3120
Ar-Lepdec	Ar-Tutabs	0.2830	0.8814	-0.5984	Ar-Adohon	Ar-Schgra	0.6081	0.8993	-0.2912
Ar-Glocon	Ar-Trivap	0.4611	0.8814	-0.4203	Mo-Limfor	Ar-Calmac	0.5927	0.8995	-0.3068
Ar-Lymdis	Ar-Stedum	0.3427	0.8820	-0.5393	Ar-Eufmex	Ar-Timmon	0.4468	0.8996	-0.4527
Ar-Lephet	Ar-Sipfla	0.2973	0.8820	-0.5847	Ar-Eufmex	Ar-Timpop	0.4116	0.8996	-0.4880
Ar-Sogfur	Ar-Timgen	0.2958	0.8822	-0.5863	Ar-Eufmex	Ar-Timshe	0.4134	0.8996	-0.4862
Ar-Calmac	Ar-Locmig	0.2695	0.8824	-0.6129	Ar-Lephet	Ar-Partep	0.4877	0.8996	-0.4119
Mo-Mermer	Ar-Sogfur	0.3842	0.8829	-0.4987	Ar-Epacla	Ar-Helarm	0.2783	0.8998	-0.6215
Ar-Locmig	Ar-Nillug	0.3079	0.8831	-0.5752	Ar-Bomman	Ar-Stedum	0.5811	0.8998	-0.3187
Ar-Manjur	Ar-Pseelo	0.3174	0.8832	-0.5658	Mo-Halrub	Ar-Phesol	0.5100	0.8998	-0.3899
Ar-Adohon	Ar-Ladful	0.3279	0.8832	-0.5553	Ar-Anogla	Ar-Epacla	0.4245	0.9000	-0.4755
Ar-Anogla	Ar-Homvit	0.3248	0.8834	-0.5586	Ar-Eupuro	Ar-Phesol	0.4706	0.9001	-0.4294
Ar-Timgen	Ar-Trivap	0.4496	0.8834	-0.4338	Mo-Mermer	Ar-Censcu	0.4974	0.9001	-0.4027
Mo-Limfor	Ar-Anogla	0.3565	0.8835	-0.5270	Ar-Stedum	Ec-Ophspi	0.5627	0.9001	-0.3374
Ar-Adohon	Ar-Thrpal	0.2481	0.8838	-0.6357	Ar-Neopin	Ec-Ophspi	0.5474	0.9004	-0.3529
Ar-Onttau	Ar-Partep	0.3536	0.8840	-0.5304	Ar-Calmac	Ar-Timshe	0.4200	0.9006	-0.4807
Mo-Limfor	Ar-Opebru	0.3939	0.8843	-0.4904	Ar-Censcu	Ar-Sogfur	0.4982	0.9011	-0.4029
Mo-Batpla	Ar-Epacla	0.4145	0.8843	-0.4697	Ar-Epacla	Ar-Lepdec	0.4281	0.9015	-0.4734
Ar-Amytra	Ar-Stemim	0.3654	0.8844	-0.5190	Mo-Halrub	Ar-Timmon	0.4403	0.9019	-0.4616
Mo-Limfor	Ar-Locmig	0.3432	0.8845	-0.5413	Mo-Halrub	Ar-Timpop	0.4440	0.9019	-0.4579
Mo-Batpla	Ar-Partep	0.3798	0.8846	-0.5048	Ar-Eufmex	Ar-Timgen	0.4267	0.9020	-0.4753
Ar-Censcu	Ar-Partep	0.2961	0.8848	-0.5887	Ar-Lymdis	Ar-Neopin	0.4149	0.9024	-0.4875
Ar-Censcu	Ar-Pluxyl	0.3938	0.8849	-0.4910	Mo-Batpla	Ar-Homvit	0.5209	0.9024	-0.3815
Mo-Limfor	Ar-Schgra	0.3926	0.8849	-0.4922	Ar-Helarm	Ar-Lephet	0.5332	0.9024	-0.3693
Ar-Galmel	Ar-Pseelo	0.3221	0.8849	-0.5628	Ar-Calmac	Ar-Timtah	0.4070	0.9025	-0.4954
Ar-Anogla	Ar-Epacla	0.3251	0.8849	-0.5598	Ar-Calmac	Ar-Timmon	0.4200	0.9027	-0.4827
Ar-Chisup	Ar-Tutabs	0.1970	0.8849	-0.6880	Ar-Aulsol	Ar-Helarm	0.5696	0.9027	-0.3332

Ar-Adohon	Ar-Censcu	0.3986	0.8852	-0.4865	Ar-Calmac	Ar-Timbar	0.4037	0.9029	-0.4992
Ar-Anogla	Ar-Sogfur	0.3489	0.8854	-0.5365	Ar-Thrpal	Ar-Trivap	0.4845	0.9029	-0.4183
Mo-Halrub	Ar-Schgra	0.4338	0.8855	-0.4518	Ar-Calmac	Ar-Timpop	0.4148	0.9029	-0.4880
Ar-Galmel	Ar-Neopin	0.2856	0.8857	-0.6001	Ar-Acypis	Ar-Censcu	0.5267	0.9030	-0.3763
Mo-Limfor	Ar-Thrpal	0.4073	0.8858	-0.4785	Mo-Batpla	Ar-Aulsol	0.4827	0.9030	-0.4204
Mo-Limfor	Ar-Aptruf	0.3977	0.8858	-0.4881	Ar-Nillug	Ar-Onttau	0.5137	0.9032	-0.3895
Mo-Halrub	Ar-Trivap	0.5284	0.8859	-0.3575	Ar-Calmac	Ar-Timcri	0.4168	0.9033	-0.4865
Ar-Neopin	Ar-Schgra	0.2999	0.8861	-0.5862	Ar-Amytra	Ar-Helarm	0.2875	0.9035	-0.6160
Ar-Helarm	Ar-Phesol	0.3422	0.8864	-0.5442	Ar-Temlon	Ar-Timtah	0.3937	0.9037	-0.5100
Ar-Lephet	Ar-Vantam	0.2812	0.8867	-0.6054	Ar-Censcu	Ar-Galmel	0.5521	0.9041	-0.3519
Ar-Adohon	Ar-Epacla	0.1515	0.8873	-0.7359	Ar-Sipfla	Ar-Stedum	0.5154	0.9041	-0.3887
An-Hydele	Ar-Phesol	0.4292	0.8874	-0.4582	Ar-Temlon	Ar-Timbar	0.3920	0.9041	-0.5121
Ar-Danmel	Ar-Limcal	0.3040	0.8875	-0.5836	Ar-Locmig	Ar-Onttau	0.4488	0.9043	-0.4555
Ar-Bomman	Ar-Helzea	0.1649	0.8880	-0.7231	Ar-Sogfur	Ar-Stemim	0.4554	0.9043	-0.4489
Mo-Mermer	Ar-Homvit	0.3533	0.8882	-0.5349	Ar-Amytra	Ar-Lymdis	0.2283	0.9046	-0.6762
Mo-Batpla	Ar-Stemim	0.4276	0.8882	-0.4607	Ar-Epacla	Ar-Nillug	0.4139	0.9051	-0.4912
An-Hydele	Ar-Eufmex	0.4653	0.8884	-0.4231	Ar-Timgen	Ar-Trivap	0.4998	0.9051	-0.4053
Ar-Chisup	Ar-Thrpal	0.3185	0.8885	-0.5700	Ar-Helarm	Ar-Schgra	0.6461	0.9052	-0.2591
Ar-Bomman	Ar-Stedum	0.3478	0.8888	-0.5409	Ar-Calmac	Ar-Locmig	0.4545	0.9053	-0.4508
St-Aphinv	St-Phycam	0.2500	0.8893	-0.6393	Ar-Neopin	Ar-Phesol	0.5017	0.9054	-0.4036
Ar-Lymdis	Ar-Neopin	0.2781	0.8894	-0.6114	Ar-Galmel	Ar-Temlon	0.4638	0.9055	-0.4416
Ar-Limcal	Ar-Manjur	0.3045	0.8895	-0.5850	Ar-Ampamp	Ar-Eufmex	0.6225	0.9055	-0.2830
Ar-Adohon	Ar-Opebru	0.1553	0.8896	-0.7343	Ar-Lepdec	Ar-Onttau	0.3408	0.9057	-0.5649
Mo-Halrub	Ar-Galmel	0.4403	0.8896	-0.4494	Mo-Halrub	Ar-Timgen	0.4279	0.9062	-0.4783
Mo-Limfor	Ar-Amytra	0.3780	0.8897	-0.5117	Ar-Censcu	Ar-Eupuro	0.4579	0.9064	-0.4485
Ar-Bomman	Ar-Helarm	0.1654	0.8898	-0.7245	Ar-Helarm	Ar-Sipfla	0.6754	0.9065	-0.2312
Ar-Acypis	Ar-Galmel	0.3133	0.8899	-0.5765	Ar-Censcu	Ar-Locmig	0.4823	0.9066	-0.4243
Ar-Ladful	Ar-Partep	0.3381	0.8902	-0.5521	Ar-Pluxyl	Ar-Sipfla	0.6552	0.9069	-0.2517
Ar-Censcu	Ar-Epacla	0.3978	0.8902	-0.4924	Ar-Locmig	Ar-Partep	0.4852	0.9070	-0.4218
Ar-Lephet	Ar-Partep	0.3493	0.8902	-0.5409	Ar-Amytra	Ar-Temlon	0.4504	0.9072	-0.4568
Ar-Pseelo	Ar-Sitmis	0.3483	0.8904	-0.5421	Ar-Lymdis	Ar-Partep	0.4892	0.9073	-0.4181
Ar-Helarm	Ar-Lephet	0.3043	0.8904	-0.5861	Ar-Ladful	Ar-Partep	0.4593	0.9073	-0.4479
Ar-Galmel	Ec-Ophspi	0.3751	0.8906	-0.5155	Ar-Acypis	Ar-Erilan	0.1079	0.9076	-0.7996
Ar-Lephet	Ar-Pluxyl	0.2883	0.8907	-0.6024	Mo-Halrub	Ar-Aptruf	0.4753	0.9078	-0.4324
Mo-Batpla	Ar-Vantam	0.4041	0.8909	-0.4868	Ar-Eufmex	Ar-Schgra	0.4338	0.9080	-0.4742
Mo-Mermer	Ar-Eupuro	0.3914	0.8909	-0.4995	Ar-Partep	Ar-Stemim	0.2579	0.9080	-0.6501
Ar-Amytra	Ar-Erilan	0.3188	0.8910	-0.5722	Mo-Batpla	Ar-Anogla	0.5890	0.9083	-0.3193
Ar-Ampamp	Ar-Sitmis	0.4046	0.8911	-0.4865	Ar-Aulsol	Ar-Lephet	0.3802	0.9087	-0.5285
Ar-Eufmex	Ar-Homvit	0.3384	0.8913	-0.5529	Ar-Calmac	Ar-Tutabs	0.4541	0.9087	-0.4546
Ar-Sogfur	Ec-Ophspi	0.3567	0.8913	-0.5346	Mo-Halrub	Ar-Timcri	0.4403	0.9088	-0.4685
Ar-Galmel	Ar-Sipfla	0.3321	0.8915	-0.5594	Mo-Halrub	Ar-Timshe	0.4357	0.9088	-0.4731
Ar-Danmel	Ar-Eufmex	0.3435	0.8916	-0.5481	St-Aphinv	St-Phycam	0.3192	0.9088	-0.5897
Mo-Batpla	Ar-Lephet	0.4038	0.8918	-0.4880	Ar-Temlon	Ar-Timmon	0.3850	0.9089	-0.5239
Ar-Acypis	Ar-Sogfur	0.3000	0.8921	-0.5922	Ar-Epacla	Ar-Homvit	0.4681	0.9091	-0.4409
Ar-Stedum	Ec-Ophspi	0.3924	0.8922	-0.4999	Ar-Anogla	Ar-Onttau	0.3402	0.9093	-0.5691
Ar-Sitmis	Ar-Temlon	0.3762	0.8925	-0.5163	Ar-Anogla	Ar-Stedum	0.5801	0.9094	-0.3293
Ar-Acypis	Ar-Epacla	0.3227	0.8926	-0.5699	Ar-Lepdec	Ar-Timcri	0.3765	0.9095	-0.5329
Ar-Pseelo	Ar-Sogfur	0.3109	0.8928	-0.5820	Ar-Anogla	Ar-Neopin	0.4065	0.9095	-0.5031
Ar-Amytra	Ar-Helarm	0.1381	0.8928	-0.7547	Mo-Halrub	Ar-Partep	0.4217	0.9096	-0.4879
Ar-Locmig	Ar-Neopin	0.2671	0.8928	-0.6257	Ar-Anogla	Ar-Trivap	0.5798	0.9096	-0.3298
Ar-Eufmex	Ar-Pseelo	0.2531	0.8929	-0.6397	Ar-Erilan	Ar-Eufmex	0.4234	0.9097	-0.4864
Ar-Limcal	Ar-Lymdis	0.3294	0.8929	-0.5635	Ar-Lepdec	Ar-Timpop	0.3780	0.9099	-0.5319
Mo-Batpla	Ar-Nillug	0.4116	0.8929	-0.4813	Ar-Acypis	Ar-Partep	0.4829	0.9101	-0.4273
Mo-Batpla	Ar-Lepdec	0.3935	0.8932	-0.4998	Ar-Limcal	Ar-Schgra	0.4823	0.9103	-0.4279
Ar-Acypis	Ar-Neopin	0.3048	0.8933	-0.5885	Ar-Glocon	Ar-Neopin	0.4778	0.9105	-0.4326
Mo-Batpla	Ar-Sipfla	0.3805	0.8935	-0.5130	Ar-Phesol	Ec-Ophspi	0.5628	0.9105	-0.3477
Ar-Manjur	Ar-Schgra	0.3026	0.8938	-0.5912	Ar-Sipfla	Ar-Sogfur	0.4458	0.9106	-0.4648
Ar-Censcu	Ar-Schgra	0.3486	0.8942	-0.5456	Ar-Galmel	Ar-Lephet	0.5203	0.9106	-0.3903
Ar-Sitmis	Ar-Stemim	0.3355	0.8944	-0.5589	Ar-Helarm	Ar-Stedum	0.5587	0.9112	-0.3524

Ar-Phesol	Ar-Temlon	0.3660	0.8945	-0.5285	Ar-Nillug	Ar-Partep	0.4685	0.9113	-0.4428
Ar-Bomman	Ar-Nillug	0.2748	0.8946	-0.6198	Ar-Epacla	Ec-Ophspi	0.5462	0.9114	-0.3652
Ar-Opebru	Ar-Sogfur	0.2575	0.8951	-0.6376	Ar-Aulsol	Ar-Partep	0.4159	0.9115	-0.4956
Ar-Chisup	Ar-Schgra	0.3127	0.8953	-0.5826	Ar-Eupann	Ar-Phesol	0.4930	0.9116	-0.4185
Ar-Amytra	Ar-Helzea	0.1405	0.8954	-0.7550	Ar-Eufmex	Ar-Limcal	0.4370	0.9116	-0.4746
Ar-Homvit	Ar-Thrpal	0.2820	0.8957	-0.6137	Ar-Temlon	Ar-Timcri	0.3850	0.9122	-0.5272
Ar-Censcu	Ar-Locmig	0.3142	0.8961	-0.5819	Ar-Limcal	Ar-Pluxyl	0.5338	0.9124	-0.3787
Ar-Eupann	Ar-Sitmis	0.2806	0.8963	-0.6157	Ar-Amytra	Ar-Phesol	0.5862	0.9126	-0.3264
Mo-Halrub	Ar-Sogfur	0.3863	0.8964	-0.5101	Ar-Temlon	Ar-Timpop	0.3863	0.9126	-0.5263
Mo-Mermer	An-Hydele	0.3732	0.8966	-0.5234	Ar-Temlon	Ar-Timshe	0.3834	0.9126	-0.5292
Ar-Nillug	Ar-Sogfur	0.1103	0.8967	-0.7863	Ar-Lephet	Ar-Stedum	0.4742	0.9128	-0.4385
Ar-Manjur	Ar-Sipfla	0.3201	0.8968	-0.5767	Ar-Aulsol	Ar-Censcu	0.4751	0.9128	-0.4377
Ar-Schgra	Ec-Ophspi	0.3843	0.8968	-0.5125	Ar-Calmac	Ar-Helarm	0.5017	0.9130	-0.4112
Mo-Batpla	Ar-Trivap	0.4318	0.8972	-0.4654	Ar-Acypis	Ar-Homvit	0.4059	0.9130	-0.5071
Mo-Halrub	Ar-Limcal	0.4264	0.8975	-0.4711	Ar-Acypis	Ar-Galmel	0.6692	0.9133	-0.2441
Ar-Eupuro	Ar-Phesol	0.3050	0.8978	-0.5927	Ar-Lepdec	Ar-Timmon	0.4007	0.9135	-0.5128
Ar-Censcu	Ar-Galmel	0.3656	0.8981	-0.5325	Mo-Limfor	Ar-Galmel	0.6424	0.9138	-0.2713
Ar-Bomman	Ar-Chisup	0.2013	0.8986	-0.6972	Ar-Lepdec	Ar-Timshe	0.3731	0.9139	-0.5408
Mo-Batpla	Ar-Limcal	0.4580	0.8987	-0.4406	Ar-Homvit	Ar-Temlon	0.4185	0.9139	-0.4954
Mo-Batpla	Ar-Temlon	0.4333	0.8990	-0.4658	St-Aphast	St-Physyr	0.3004	0.9140	-0.6135
Ar-Lepdec	Ar-Partep	0.3385	0.8996	-0.5611	Ar-Lepdec	Ar-Timtah	0.3746	0.9141	-0.5395
Ar-Danmel	Ar-Phesol	0.3586	0.8999	-0.5413	Ar-Adohon	Ar-Limcal	0.5182	0.9144	-0.3962
Ar-Lephet	Ar-Neopin	0.2252	0.9000	-0.6747	Ar-Censcu	Ar-Eupann	0.4632	0.9144	-0.4512
Ar-Partep	Ar-Sogfur	0.3486	0.9002	-0.5515	Ar-Lepdec	Ar-Timbar	0.3748	0.9145	-0.5397
Ar-Helarm	Ar-Opebru	0.1448	0.9003	-0.7556	Ar-Galmel	Ar-Sipfla	0.6698	0.9150	-0.2452
Mo-Batpla	Ar-Helarm	0.4018	0.9004	-0.4986	Mo-Halrub	Ar-Lephet	0.4873	0.9150	-0.4277
Ar-Onttau	Ar-Phesol	0.2941	0.9004	-0.6063	Ar-Aulsol	Ar-Trivap	0.4419	0.9156	-0.4737
Ar-Danmel	Ar-Thrpal	0.2512	0.9006	-0.6493	Mo-Limfor	Ar-Onttau	0.5984	0.9156	-0.3173
Ar-Lymdis	Ar-Partep	0.3498	0.9006	-0.5508	Ar-Lepdec	Ar-Partep	0.4933	0.9157	-0.4224
Ar-Sogfur	Ar-Stemim	0.3705	0.9007	-0.5302	Ar-Epacla	Ar-Lymdis	0.2485	0.9157	-0.6672
Ar-Eufmex	Ar-Osmbic	0.1743	0.9013	-0.7270	Ar-Lymdis	Ar-Nillug	0.4210	0.9158	-0.4948
Ar-Epacla	Ar-Sipfla	0.3361	0.9013	-0.5653	Ar-Partep	Ar-Sipfla	0.4819	0.9158	-0.4339
Ar-Galmel	Ar-Nillug	0.2945	0.9014	-0.6068	Mo-Batpla	Ar-Phesol	0.5707	0.9163	-0.3455
An-Hydele	Ar-Locmig	0.4298	0.9018	-0.4719	Ar-Censcu	Ar-Neopin	0.5052	0.9165	-0.4112
Ar-Amytra	Ar-Sipfla	0.3268	0.9018	-0.5750	Ar-Manjur	Ar-Thrpal	0.4857	0.9168	-0.4311
Ar-Locmig	Ar-Onttau	0.3148	0.9019	-0.5870	Mo-Batpla	Ar-Censcu	0.4808	0.9169	-0.4361
Ar-Homvit	Ar-Limcal	0.3126	0.9021	-0.5895	Ar-Bomman	Ar-Homvit	0.4600	0.9170	-0.4570
Ar-Bomman	Ar-Sitmis	0.3272	0.9022	-0.5749	Ar-Phesol	Ar-Stemim	0.4748	0.9172	-0.4424
Mo-Batpla	Ar-Helzea	0.3993	0.9023	-0.5031	Ar-Locmig	Ar-Neopin	0.3754	0.9173	-0.5419
Ar-Anogla	Ar-Trivap	0.4693	0.9024	-0.4331	Ar-Eupuro	Ar-Partep	0.4891	0.9173	-0.4282
Ar-Amytra	Ar-Temlon	0.3183	0.9024	-0.5841	Ar-Nillug	Ar-Stemim	0.4774	0.9174	-0.4400
Ar-Glocon	Ar-Limcal	0.2984	0.9024	-0.6040	Ar-Sogfur	Ar-Tutabs	0.4442	0.9176	-0.4734
Ar-Chisup	Ar-Vantam	0.1959	0.9027	-0.7068	Ar-Manjur	Ar-Stedum	0.6685	0.9178	-0.2493
Ar-Anogla	Ar-Onttau	0.2701	0.9028	-0.6327	Ar-Homvit	Ar-Stemim	0.4091	0.9184	-0.5094
Ar-Amytra	Ar-Phesol	0.3257	0.9028	-0.5771	Ar-Aulsol	Ar-Limcal	0.4243	0.9185	-0.4941
An-Hydele	Ar-Neopin	0.3622	0.9029	-0.5407	Ar-Anogla	Ar-Glocon	0.4518	0.9187	-0.4668
Ar-Chisup	Ar-Epacla	0.1961	0.9030	-0.7070	Mo-Batpla	Ar-Erilan	0.5322	0.9187	-0.3866
Ar-Nillug	Ar-Pluxyl	0.2386	0.9031	-0.6645	Ar-Locmig	Ar-Sipfla	0.4241	0.9188	-0.4947
Ar-Neopin	Ar-Sipfla	0.3069	0.9031	-0.5962	Ar-Osmbic	Ar-Tutabs	0.5086	0.9195	-0.4109
Ar-Thrpal	Ar-Trivap	0.4280	0.9031	-0.4752	Mo-Mermer	Ar-Calmac	0.5152	0.9202	-0.4051
St-Aphste	St-Physyr	0.2302	0.9032	-0.6730	Ar-Homvit	Ar-Neopin	0.3708	0.9204	-0.5496
Ar-Anogla	Ar-Nillug	0.3550	0.9032	-0.5482	Ar-Ladful	Ar-Nillug	0.3387	0.9206	-0.5819
Ar-Limcal	Ar-Nillug	0.3014	0.9033	-0.6019	Ar-Stedum	Ar-Trivap	0.5753	0.9206	-0.3453
Mo-Mermer	Ar-Helzea	0.3798	0.9034	-0.5237	Ar-Locmig	Ar-Stemim	0.4183	0.9210	-0.5027
Ar-Censcu	Ar-Stedum	0.2390	0.9036	-0.6646	Ar-Calmac	Ar-Neopin	0.4437	0.9211	-0.4774
Ar-Censcu	Ar-Sogfur	0.3920	0.9036	-0.5116	Mo-Mermer	Ar-Thrpal	0.5185	0.9214	-0.4028
Ar-Helzea	Ar-Opebru	0.1477	0.9040	-0.7563	Ar-Calmac	Ar-Osmbic	0.4411	0.9225	-0.4813
Mo-Batpla	Ar-Pseelo	0.4046	0.9041	-0.4994	Ar-Pluxyl	Ar-Thrpal	0.4684	0.9225	-0.4541
Ar-Lymdis	Ar-Pseelo	0.3172	0.9045	-0.5873	Ar-Eufmex	Ar-Nillug	0.4623	0.9227	-0.4604

Ar-Acypis	Ar-Partep	0.3604	0.9046	-0.5442	Ar-Homvit	Ar-Schgra	0.4029	0.9233	-0.5204
Ar-Epacla	Ar-Lepdec	0.2720	0.9047	-0.6326	Ar-Erilan	Ar-Nillug	0.4620	0.9235	-0.4615
Ar-Eufmex	Ar-Timcri	0.3254	0.9047	-0.5793	Mo-Batpla	Ar-Neopin	0.5751	0.9236	-0.3485
Ar-Eufmex	Ar-Timgen	0.3277	0.9047	-0.5771	Ar-Homvit	Ar-Lepdec	0.4557	0.9239	-0.4682
Ar-Acypis	Ar-Stedum	0.3399	0.9048	-0.5648	Ar-Sogfur	Ar-Timshe	0.3217	0.9240	-0.6022
Ar-Schgra	Ar-Sogfur	0.3041	0.9050	-0.6008	Mo-Batpla	Ec-Ophspi	0.5094	0.9241	-0.4147
Ar-Adohon	Ar-Schgra	0.3442	0.9050	-0.5607	Ar-Acypis	Ar-Lymdis	0.5272	0.9241	-0.3970
Ar-Lephet	Ar-Nillug	0.3105	0.9050	-0.5946	Ar-Anogla	Ar-Lymdis	0.4346	0.9242	-0.4897
Ar-Pseelo	Ar-Schgra	0.3469	0.9056	-0.5587	Ar-Partep	Ar-Schgra	0.4563	0.9242	-0.4680
Ar-Partep	Ar-Vantam	0.3425	0.9056	-0.5631	Ar-Limcal	Ar-Stedum	0.5137	0.9243	-0.4106
Ar-Amytra	Ar-Lymdis	0.1576	0.9057	-0.7481	Mo-Batpla	Ar-Adohon	0.6649	0.9244	-0.2595
Ar-Manjur	Ar-Partep	0.3677	0.9057	-0.5379	Ar-Galmel	Ar-Locmig	0.5109	0.9245	-0.4137
Ar-Phesol	Ar-Stemim	0.3303	0.9060	-0.5757	Mo-Batpla	Ar-Osmbic	0.5043	0.9245	-0.4202
Ar-Vantam	Ec-Ophspi	0.3839	0.9060	-0.5220	Ar-Erilan	Ar-Phesol	0.4222	0.9248	-0.5025
Ar-Anogla	Ar-Stedum	0.3475	0.9060	-0.5585	Ar-Epacla	Ar-Timmon	0.4190	0.9248	-0.5058
Ar-Lepdec	Ar-Pluxyl	0.2733	0.9064	-0.6332	Ar-Acypis	Ar-Helarm	0.6714	0.9252	-0.2538
Ar-Anogla	Ar-Lymdis	0.3129	0.9066	-0.5936	Ar-Locmig	Ar-Osmbic	0.3868	0.9254	-0.5385
Ar-Limcal	Ar-Pluxyl	0.2914	0.9066	-0.6152	Mo-Limfor	Ar-Eupann	0.5709	0.9256	-0.3547
Ar-Eupuro	Ar-Sitmis	0.2862	0.9068	-0.6206	Ar-Lephet	Ar-Sogfur	0.3917	0.9259	-0.5342
Mo-Mermer	Ar-Censcu	0.3972	0.9068	-0.5096	Ar-Onttau	Ar-Pluxyl	0.5277	0.9259	-0.3983
Ar-Helzea	Ar-Lephet	0.3055	0.9070	-0.6015	St-Aphste	St-Phycam	0.2793	0.9260	-0.6467
Ar-Censcu	Ar-Helzea	0.3978	0.9073	-0.5094	Ar-Phesol	Ar-Osmbic	0.4769	0.9262	-0.4493
Ar-Helzea	Ar-Phesol	0.3362	0.9076	-0.5714	Ar-Aulsol	Ar-Timgen	0.3776	0.9264	-0.5488
Mo-Limfor	Ar-Calmac	0.3526	0.9076	-0.5550	Ar-Sogfur	Ar-Timtah	0.3288	0.9267	-0.5979
An-Hydele	Ar-Timgen	0.3929	0.9077	-0.5147	Ar-Aulsol	Ar-Erilan	0.1261	0.9268	-0.8008
Mo-Halrub	Ar-Phesol	0.4561	0.9077	-0.4516	Ar-Lepdec	Ar-Pluxyl	0.4984	0.9268	-0.4285
Ar-Helzea	Ar-Pseelo	0.3385	0.9080	-0.5696	Ar-Epacla	Ar-Timshe	0.4102	0.9269	-0.5167
Ar-Censcu	Ar-Sipfla	0.3627	0.9082	-0.5455	Ar-Sogfur	Ar-Timbar	0.3289	0.9271	-0.5982
Ar-Epacla	Ec-Ophspi	0.3744	0.9083	-0.5338	Mo-Limfor	Ar-Eupuro	0.5755	0.9277	-0.3522
Ar-Locmig	Ar-Schgra	0.2958	0.9083	-0.6125	Ar-Glocon	Ar-Temlon	0.4873	0.9279	-0.4406
Ar-Chisup	Ar-Partep	0.3503	0.9085	-0.5582	Ar-Erilan	Ar-Locmig	0.4106	0.9284	-0.5178
Ar-Limcal	Ar-Schgra	0.3345	0.9086	-0.5742	Ar-Helarm	Ar-Nillug	0.5493	0.9288	-0.3795
Mo-Halrub	Ar-Timgen	0.4402	0.9088	-0.4686	Ar-Lephet	Ar-Schgra	0.4302	0.9289	-0.4987
Ar-Calmac	Ar-Timgen	0.3303	0.9093	-0.5789	Ar-Sogfur	Ar-Timmon	0.3261	0.9290	-0.6028
Ar-Phesol	Ar-Vantam	0.3288	0.9095	-0.5807	Ar-Epacla	Ar-Timpop	0.4101	0.9290	-0.5190
Ar-Eufmex	Ar-Timpop	0.3125	0.9099	-0.5975	Ar-Sogfur	Ar-Timpop	0.3248	0.9295	-0.6047
Ar-Anogla	Ar-Chisup	0.3219	0.9102	-0.5883	Ar-Amytra	Ar-Calmac	0.4526	0.9297	-0.4770
Ar-Lepdec	Ar-Onttau	0.2605	0.9103	-0.6498	Ar-Acypis	Ar-Locmig	0.4074	0.9299	-0.5225
An-Hydele	Ar-Anogla	0.4227	0.9103	-0.4877	Ar-Neopin	Ar-Trivap	0.4915	0.9303	-0.4388
Ar-Calmac	Ar-Pluxyl	0.2905	0.9104	-0.6198	Ar-Schgra	Ar-Trivap	0.4224	0.9304	-0.5080
Ar-Anogla	Ar-Neopin	0.3064	0.9107	-0.6043	Ar-Partep	Ar-Timgen	0.4721	0.9307	-0.4586
Ar-Ampamp	Ar-Eufmex	0.4989	0.9107	-0.4118	Ar-Homvit	Ar-Timgen	0.3274	0.9315	-0.6041
Mo-Batpla	Ar-Sogfur	0.4215	0.9108	-0.4893	Ar-Aulsol	Ar-Homvit	0.3296	0.9316	-0.6020
An-Hydele	Ar-Partep	0.4149	0.9110	-0.4960	Ar-Eupann	Ar-Schgra	0.4310	0.9317	-0.5007
Ar-Epacla	Ar-Helarm	0.1380	0.9112	-0.7732	Mo-Halrub	Ar-Lepdec	0.4476	0.9317	-0.4841
Ar-Censcu	Ar-Eupuro	0.3576	0.9112	-0.5536	Ar-Homvit	Ar-Stedum	0.4891	0.9320	-0.4429
Ar-Homvit	Ar-Timgen	0.2853	0.9114	-0.6261	Ar-Bomman	Ar-Galmel	0.2938	0.9322	-0.6384
Mo-Mermer	Ar-Helarm	0.3832	0.9115	-0.5283	Mo-Limfor	Ar-Ladful	0.5458	0.9323	-0.3864
Ar-Osmbic	Ar-Stemim	0.3295	0.9116	-0.5821	Ar-Epacla	Ar-Timgen	0.4260	0.9326	-0.5066
Ar-Epacla	Ar-Lymdis	0.1786	0.9119	-0.7333	Ar-Calmac	Ar-Sipfla	0.5153	0.9327	-0.4174
An-Hydele	Ar-Homvit	0.3290	0.9120	-0.5830	Mo-Batpla	Ar-Glocon	0.6779	0.9329	-0.2550
Mo-Batpla	Ar-Censcu	0.3771	0.9121	-0.5350	Ar-Epacla	Ar-Timcri	0.4190	0.9331	-0.5141
Ar-Helarm	Ar-Schgra	0.3275	0.9121	-0.5846	Ar-Sogfur	Ar-Timcri	0.3261	0.9332	-0.6071
Ar-Censcu	Ar-Trivap	0.4537	0.9124	-0.4587	Ar-Erilan	Ar-Homvit	0.4024	0.9333	-0.5308
Ar-Lepdec	Ar-Lymdis	0.2724	0.9125	-0.6402	Ar-Glocon	Ar-Thrpal	0.4313	0.9334	-0.5021
Ar-Calmac	Ar-Neopin	0.3134	0.9128	-0.5994	Ar-Lymdis	Ar-Onttau	0.4632	0.9339	-0.4707
Ar-Homvit	Ar-Nillug	0.2333	0.9129	-0.6796	Mo-Mermer	Ar-Temlon	0.5183	0.9341	-0.4157
Ar-Osmbic	Ar-Timgen	0.3170	0.9129	-0.5959	Ar-Schgra	Ar-Stedum	0.5226	0.9346	-0.4120
Mo-Limfor	Ar-Onttau	0.3846	0.9130	-0.5284	Ar-Anogla	Ar-Sipfla	0.6169	0.9347	-0.3178

Ar-Epacla	Ar-Pseelo	0.3255	0.9131	-0.5876	Ar-Epacla	Ar-Timbar	0.4280	0.9348	-0.5068
Ar-Eufmex	Ar-Schgra	0.3276	0.9132	-0.5856	Ar-Epacla	Ar-Timtah	0.4281	0.9348	-0.5067
Ar-Acypis	Ar-Censcu	0.3518	0.9135	-0.5617	Ar-Calmac	Ar-Partep	0.4983	0.9349	-0.4367
Ar-Locmig	Ar-Partep	0.3182	0.9136	-0.5953	Ar-Aulsol	Ar-Onttau	0.4217	0.9353	-0.5136
Ar-Epacla	Ar-Timgen	0.3211	0.9138	-0.5927	Ar-Amytra	Ar-Schgra	0.6241	0.9355	-0.3115
Ar-Temlon	Ar-Timcri	0.3527	0.9145	-0.5618	Ar-Ladful	Ar-Manjur	0.5176	0.9355	-0.4180
Ar-Glocon	Ar-Vantam	0.2405	0.9145	-0.6740	Ar-Ladful	Ec-Ophspi	0.5579	0.9356	-0.3776
Mo-Halrub	Ar-Timpop	0.4568	0.9146	-0.4578	Ar-Ampamp	Ar-Phesol	0.5998	0.9356	-0.3358
Ar-Temlon	Ar-Timpop	0.3374	0.9149	-0.5775	Ar-Ampamp	Ar-Timgen	0.4397	0.9360	-0.4963
Ar-Chisup	Ar-Pseelo	0.3235	0.9150	-0.5915	Ar-Ladful	Ar-Phesol	0.4696	0.9361	-0.4665
Ar-Pluxyl	Ar-Sipfla	0.3426	0.9153	-0.5727	Ar-Bomman	Ar-Manjur	0.3056	0.9361	-0.6306
Ar-Homvit	Ar-Lepdec	0.2807	0.9153	-0.6346	Ar-Lepdec	Ar-Trivap	0.5945	0.9364	-0.3420
Ar-Pseelo	Ar-Trivap	0.4570	0.9154	-0.4583	Ar-Eupuro	Ar-Tutabs	0.4262	0.9365	-0.5104
Ar-Lephet	Ar-Sogfur	0.2943	0.9154	-0.6211	Ar-Schgra	Ar-Timgen	0.4371	0.9375	-0.5004
Ar-Eupann	Ar-Phesol	0.3027	0.9155	-0.6128	Mo-Halrub	Ar-Homvit	0.4291	0.9375	-0.5084
Ar-Ladful	Ar-Phesol	0.3395	0.9155	-0.5761	Ar-Locmig	Ar-Stedum	0.4713	0.9378	-0.4666
Mo-Limfor	Ar-Galmel	0.3762	0.9155	-0.5394	Mo-Halrub	Ar-Ampamp	0.4914	0.9383	-0.4469
Ar-Lepdec	Ar-Trivap	0.4108	0.9157	-0.5048	Ar-Bomman	Ar-Lymdis	0.2256	0.9384	-0.7128
Mo-Limfor	Ar-Vantam	0.3766	0.9157	-0.5391	Mo-Batpla	Ar-Calmac	0.5829	0.9387	-0.3558
Mo-Halrub	Ar-Aptruf	0.3934	0.9159	-0.5225	Ar-Lymdis	Ar-Sipfla	0.4966	0.9389	-0.4424
Ar-Acypis	Ar-Amytra	0.3150	0.9161	-0.6010	Ar-Adohon	Ar-Partep	0.5766	0.9390	-0.3624
Mo-Halrub	Ar-Lephet	0.4265	0.9161	-0.4896	Ar-Limcal	Ar-Sogfur	0.4605	0.9390	-0.4785
An-Hydele	Ar-Tutabs	0.3156	0.9161	-0.6005	Ar-Lephet	Ar-Trivap	0.5278	0.9396	-0.4119
Ar-Manjur	Ar-Nillug	0.2829	0.9161	-0.6332	Ar-Thrpal	Ec-Ophspi	0.5052	0.9397	-0.4345
St-Aphinv	Fn-Morsp.	0.3583	0.9165	-0.5582	Ar-Amytra	Ar-Timmon	0.4799	0.9398	-0.4599
Ar-Acypis	Ar-Erilan	0.1323	0.9166	-0.7843	Ar-Bomman	Ec-Ophspi	0.5770	0.9400	-0.3630
Ar-Amytra	Ar-Calmac	0.2888	0.9167	-0.6279	Ar-Calmac	Ar-Nillug	0.4449	0.9401	-0.4952
Ar-Helarm	Ar-Stedum	0.3647	0.9168	-0.5521	Ar-Erilan	Ar-Eupuro	0.4318	0.9402	-0.5084
Ar-Neopin	Ec-Ophspi	0.3840	0.9170	-0.5330	Ar-Acypis	Ar-Anogla	0.5947	0.9406	-0.3460
Ar-Partep	Ar-Stemim	0.2117	0.9174	-0.7057	Ar-Ampamp	Ar-Partep	0.6411	0.9407	-0.2996
Ar-Danmel	Ar-Schgra	0.3164	0.9177	-0.6013	Ar-Aptruf	Ar-Timbar	0.3681	0.9410	-0.5729
Mo-Halrub	Ar-Pseelo	0.4549	0.9178	-0.4628	Ar-Lymdis	Ar-Stemim	0.4788	0.9414	-0.4626
Ar-Epacla	Ar-Timpop	0.3075	0.9179	-0.6104	Ar-Aptruf	Ar-Timtah	0.3678	0.9415	-0.5736
Mo-Batpla	Ar-Homvit	0.3896	0.9181	-0.5285	Ar-Adohon	Ar-Stedum	0.5844	0.9416	-0.3572
Ar-Limcal	Ar-Sogfur	0.3141	0.9184	-0.6043	Ar-Bomman	Ar-Sipfla	0.6164	0.9417	-0.3253
Ar-Ladful	Ar-Manjur	0.3135	0.9186	-0.6051	Ar-Acypis	Ar-Calmac	0.5041	0.9417	-0.4376
Ar-Censcu	Ar-Neopin	0.3604	0.9187	-0.5583	St-Aphast	St-Phycam	0.2887	0.9419	-0.6532
Ar-Phesol	Ec-Ophspi	0.4185	0.9188	-0.5003	Ar-Anogla	Ar-Tutabs	0.4437	0.9419	-0.4982
Ar-Manjur	Ar-Stedum	0.3661	0.9188	-0.5527	Ar-Ampamp	Ar-Locmig	0.5374	0.9421	-0.4047
Ar-Galmel	Ar-Temlon	0.3375	0.9188	-0.5813	Ar-Eufmex	Ar-Glocon	0.5316	0.9425	-0.4109
Ar-Bomman	Ar-Galmel	0.1967	0.9193	-0.7226	Ar-Censcu	Ar-Lymdis	0.5397	0.9425	-0.4029
Ar-Nillug	Ar-Pseelo	0.3210	0.9194	-0.5984	Ar-Epacla	Ar-Trivap	0.5635	0.9426	-0.3791
Ar-Lymdis	Ar-Schgra	0.3167	0.9196	-0.6029	Ar-Nillug	Ar-Trivap	0.4331	0.9426	-0.5095
Ar-Censcu	Ar-Danmel	0.3825	0.9197	-0.5372	St-Pilapi	Mo-Limfor	0.7328	0.9430	-0.2102
Ar-Partep	Ar-Timgen	0.3540	0.9199	-0.5659	Ar-Osmbic	Ar-Timgen	0.4040	0.9438	-0.5397
Ar-Sipfla	Ar-Sogfur	0.3359	0.9200	-0.5841	Ar-Helarm	Ar-Limcal	0.5638	0.9438	-0.3800
Mo-Batpla	Ar-Phesol	0.4114	0.9201	-0.5087	Mo-Batpla	Ar-Ampamp	0.7350	0.9440	-0.2090
An-Hydele	Ar-Erilan	0.4411	0.9203	-0.4792	Ar-Erilan	Ar-Tutabs	0.6000	0.9442	-0.3442
Ar-Opebru	Ar-Stemim	0.3726	0.9205	-0.5479	Ar-Amytra	Ar-Timcri	0.4381	0.9447	-0.5067
Ar-Bomman	Ar-Homvit	0.2781	0.9206	-0.6425	Ar-Eupann	Ar-Tutabs	0.4259	0.9448	-0.5189
Ar-Glocon	Ar-Neopin	0.2813	0.9207	-0.6394	Ar-Limcal	Ar-Sipfla	0.4915	0.9452	-0.4537
Ar-Homvit	Ar-Sipfla	0.3165	0.9208	-0.6044	Ar-Amytra	Ar-Timshe	0.4326	0.9452	-0.5127
Ar-Epacla	Ar-Nillug	0.2725	0.9208	-0.6483	Ar-Amytra	Ar-Timpop	0.4395	0.9452	-0.5058
Ar-Neopin	Ar-Phesol	0.2982	0.9210	-0.6229	Ar-Bomman	Ar-Lephet	0.4851	0.9453	-0.4602
Ar-Erilan	Ar-Eufmex	0.3209	0.9211	-0.6003	Ar-Acypis	Ar-Adohon	0.6027	0.9456	-0.3430
St-Aphast	St-Physyr	0.2274	0.9212	-0.6938	Ar-Calmac	Ar-Epacla	0.4521	0.9462	-0.4942
An-Hydele	Ar-Lephet	0.3614	0.9213	-0.5599	Ar-Lepdec	Ar-Lymdis	0.4531	0.9463	-0.4932
Ar-Erilan	Ar-Eupuro	0.3024	0.9214	-0.6190	Ar-Homvit	Ar-Partep	0.4371	0.9465	-0.5093
Mo-Batpla	Ar-Osmbic	0.3928	0.9214	-0.5286	Ar-Anogla	Ar-Aptruf	0.4869	0.9469	-0.4600

Mo-Mermer	Ar-Pseelo	0.4234	0.9214	-0.4980	Ar-Homvit	Ar-Osmbic	0.3980	0.9475	-0.5494
Mo-Batpla	Ar-Erilan	0.3918	0.9215	-0.5297	Ar-Eupann	Ar-Partep	0.5017	0.9475	-0.4458
Mo-Halrub	Ar-Timcri	0.4423	0.9217	-0.4794	Ar-Censcu	Ar-Trivap	0.5681	0.9481	-0.3800
Ar-Erilan	Ar-Homvit	0.3074	0.9218	-0.6145	Ar-Sogfur	Ar-Stedum	0.5242	0.9481	-0.4239
Ar-Epacla	Ar-Timcri	0.3128	0.9220	-0.6092	Ar-Acypis	Ar-Limcal	0.4995	0.9483	-0.4488
Ar-Eufmex	Ar-Limcal	0.3503	0.9222	-0.5718	Ar-Onttau	Ar-Stemim	0.4396	0.9484	-0.5088
Ar-Anogla	Ar-Glocon	0.3277	0.9225	-0.5947	Ar-Lephet	Ar-Onttau	0.4511	0.9486	-0.4976
St-Aphste	St-Phycam	0.2489	0.9225	-0.6735	Ar-Acypis	Ar-Timgen	0.4335	0.9494	-0.5158
Ar-Nillug	Ar-Partep	0.3772	0.9227	-0.5454	Ar-Partep	Ar-Timcri	0.4707	0.9495	-0.4788
Ar-Eufmex	Ar-Nillug	0.3809	0.9227	-0.5418	Mo-Halrub	Ar-Adohon	0.4569	0.9497	-0.4928
Mo-Batpla	Ar-Glocon	0.4324	0.9230	-0.4906	Ar-Aulsol	Ar-Timbar	0.3834	0.9497	-0.5663
Ar-Ladful	Ar-Sitmis	0.3404	0.9233	-0.5828	Ar-Erilan	Ar-Eupann	0.4317	0.9498	-0.5180
Ar-Helarm	Ar-Sipfla	0.3373	0.9234	-0.5861	Ar-Partep	Ar-Timmon	0.4688	0.9499	-0.4811
Ar-Glocon	Ar-Lepdec	0.3109	0.9236	-0.6126	Ar-Glocon	Ar-Ladful	0.4897	0.9500	-0.4603
Ar-Partep	Ar-Sipfla	0.3546	0.9237	-0.5691	Ar-Phesol	Ar-Stedum	0.6031	0.9500	-0.3470
Ar-Sogfur	Ar-Timpop	0.3082	0.9241	-0.6159	Ar-Bomman	Ar-Locmig	0.4600	0.9501	-0.4901
Ar-Ampamp	Ar-Timgen	0.4422	0.9243	-0.4821	Ar-Aulsol	Ar-Timtah	0.3852	0.9502	-0.5650
Ar-Locmig	Ar-Sipfla	0.2869	0.9244	-0.6375	Ar-Erilan	Ar-Partep	0.4725	0.9503	-0.4778
Ar-Locmig	Ar-Osmbic	0.2828	0.9252	-0.6424	Ar-Calmac	Ar-Schgra	0.4912	0.9505	-0.4593
Mo-Mermer	Ar-Temlon	0.4154	0.9253	-0.5099	Ar-Nillug	Ar-Stedum	0.5293	0.9506	-0.4213
Ar-Osmbic	Ar-Tutabs	0.3239	0.9255	-0.6016	Ar-Nillug	Ar-Thrpal	0.4144	0.9513	-0.5369
Ar-Homvit	Ar-Opebru	0.2844	0.9260	-0.6415	Ar-Schgra	Ar-Stemim	0.4335	0.9514	-0.5178
Ar-Lephet	Ar-Onttau	0.2855	0.9261	-0.6406	Ar-Eupuro	Ar-Schgra	0.4218	0.9515	-0.5297
Ar-Helzea	Ar-Schgra	0.3241	0.9262	-0.6020	Ar-Pluxyl	Ar-Temlon	0.4474	0.9516	-0.5042
Mo-Batpla	Ar-Danmel	0.4318	0.9263	-0.4945	Ar-Ladful	Ar-Stedum	0.4640	0.9517	-0.4877
Ar-Danmel	Ar-Nillug	0.2818	0.9264	-0.6446	Ar-Lepdec	Ar-Neopin	0.4276	0.9518	-0.5241
Ar-Anogla	Ar-Opebru	0.3395	0.9264	-0.5870	Ar-Ampamp	Ar-Timtah	0.4346	0.9518	-0.5171
An-Hydele	Ar-Sipfla	0.4382	0.9265	-0.4883	Ar-Osmbic	Ar-Stemim	0.4226	0.9520	-0.5294
Mo-Limfor	Ar-Sitmis	0.3925	0.9267	-0.5342	Ar-Ampamp	Ar-Timbar	0.4379	0.9522	-0.5144
Ar-Bomman	Ar-Calmac	0.2982	0.9271	-0.6290	Ar-Lymdis	Ar-Timcri	0.4351	0.9524	-0.5173
Ar-Limcal	Ar-Stedum	0.3836	0.9274	-0.5438	Mo-Halrub	Ar-Lymdis	0.5024	0.9525	-0.4501
Ar-Helarm	Ar-Vantam	0.1622	0.9275	-0.7653	Ar-Anogla	Ar-Galmel	0.4727	0.9526	-0.4799
Ar-Lepdec	Ar-Locmig	0.2632	0.9275	-0.6644	Ar-Homvit	Ar-Timshe	0.3248	0.9527	-0.6279
Ar-Danmel	Ar-Partep	0.3467	0.9278	-0.5810	Ar-Ampamp	Ar-Timcri	0.4367	0.9528	-0.5161
Ar-Sogfur	Ar-Timcri	0.2961	0.9278	-0.6317	Ar-Bomman	Ar-Censcu	0.5283	0.9530	-0.4247
Ar-Censcu	Ar-Eupann	0.3452	0.9279	-0.5827	Mo-Mermer	Ar-Osmbic	0.5060	0.9530	-0.4471
Mo-Halrub	Ar-Lepdec	0.4362	0.9279	-0.4917	Ar-Amytra	Ar-Timbar	0.4294	0.9530	-0.5237
Mo-Batpla	Ar-Adohon	0.4193	0.9279	-0.5086	Ar-Amytra	Ar-Timtah	0.4296	0.9530	-0.5234
Ar-Lephet	Ar-Trivap	0.4007	0.9280	-0.5273	Ar-Partep	Ar-Timpop	0.4667	0.9534	-0.4868
Ar-Amytra	Ar-Schgra	0.3147	0.9281	-0.6134	Ar-Eupuro	Ar-Galmel	0.4400	0.9535	-0.5135
Ar-Neopin	Ar-Trivap	0.4152	0.9287	-0.5135	Ar-Lymdis	Ar-Timtah	0.4320	0.9536	-0.5217
Ar-Eupann	Ar-Opebru	0.2660	0.9287	-0.6627	Ar-Timtah	Ar-Trivap	0.5157	0.9538	-0.4381
Ar-Lephet	Ar-Stedum	0.3587	0.9287	-0.5700	Ar-Partep	Ar-Timshe	0.4704	0.9539	-0.4835
Mo-Batpla	Ar-Anogla	0.4025	0.9287	-0.5263	Ar-Partep	Ar-Timtah	0.4782	0.9539	-0.4757
Ar-Danmel	Ar-Pseelo	0.3144	0.9289	-0.6146	Ar-Lymdis	Ar-Timbar	0.4352	0.9542	-0.5190
Ar-Ladful	Ec-Ophspi	0.3917	0.9291	-0.5374	Mo-Mermer	Ar-Erilan	0.5128	0.9542	-0.4414
Ar-Homvit	Ar-Stemim	0.3503	0.9291	-0.5788	Ar-Neopin	Ar-Sogfur	0.3447	0.9542	-0.6096
Ar-Adohon	Ar-Lepdec	0.2951	0.9291	-0.6340	Ar-Amytra	Ar-Trivap	0.5918	0.9544	-0.3626
Ar-Calmac	Ar-Osmbic	0.2994	0.9291	-0.6297	Ar-Phesol	Ar-Timmon	0.5019	0.9544	-0.4526
Ar-Acypis	Ar-Anogla	0.3150	0.9293	-0.6143	Ar-Stedum	Ar-Timcri	0.5229	0.9544	-0.4315
Ar-Galmel	Ar-Locmig	0.2726	0.9294	-0.6568	Ar-Calmac	Ar-Manjur	0.5072	0.9547	-0.4476
Ar-Calmac	Ar-Helarm	0.2889	0.9296	-0.6406	Ar-Amytra	Ar-Nillug	0.4862	0.9548	-0.4686
Ar-Timcri	Ar-Trivap	0.4374	0.9296	-0.4922	Mo-Batpla	Ar-Eufmex	0.5221	0.9548	-0.4327
Ar-Anogla	Ar-Danmel	0.3341	0.9299	-0.5958	Ar-Aulsol	Ar-Timpop	0.3668	0.9548	-0.5880
Ar-Timpop	Ar-Trivap	0.4482	0.9300	-0.4818	Ar-Ladful	Ar-Schgra	0.4098	0.9549	-0.5450
Ar-Danmel	Ar-Lepdec	0.2903	0.9304	-0.6401	Ar-Stedum	Ar-Timpop	0.5224	0.9550	-0.4326
Ar-Erilan	Ar-Locmig	0.3185	0.9304	-0.6119	Ar-Aulsol	Ar-Timcri	0.3725	0.9553	-0.5828
Ar-Nillug	Ar-Onttau	0.3340	0.9309	-0.5969	Ar-Aulsol	Ar-Timmon	0.3744	0.9553	-0.5809
Ar-Glocon	Ar-Thrpal	0.2531	0.9316	-0.6785	Ar-Lymdis	Ar-Timpop	0.4417	0.9554	-0.5137

Ar-Calmac	Ar-Epacla	0.2955	0.9317	-0.6362	Ar-Onttau	Ar-Sipfla	0.5381	0.9555	-0.4174
Ar-Locmig	Ar-Stemim	0.2962	0.9317	-0.6355	Ar-Ampamp	Ar-Timmon	0.4604	0.9558	-0.4954
Ar-Calmac	Ar-Timpop	0.3280	0.9319	-0.6039	Ar-Lymdis	Ar-Timmon	0.4335	0.9559	-0.5224
Ar-Anogla	Ar-Sipfla	0.3190	0.9321	-0.6132	Ar-Eufmex	Ar-Ladful	0.3946	0.9559	-0.5614
Ar-Calmac	Ar-Timcri	0.3193	0.9323	-0.6130	Ar-Adohon	Ar-Erilan	0.5847	0.9560	-0.3713
Ar-Sipfla	Ar-Stedum	0.3334	0.9323	-0.5989	Ar-Stedum	Ar-Timshe	0.5313	0.9561	-0.4248
Ar-Bomman	Ar-Lephet	0.2975	0.9324	-0.6349	Ar-Stedum	Ar-Timmon	0.5229	0.9561	-0.4332
Ar-Osmbic	Ar-Timcri	0.3130	0.9325	-0.6195	Ar-Temlon	Ar-Tutabs	0.4663	0.9563	-0.4899
Ar-Helzea	Ar-Vantam	0.1638	0.9326	-0.7688	Ar-Amytra	Ar-Timgen	0.4326	0.9564	-0.5238
Ar-Onttau	Ar-Pluxyl	0.3063	0.9327	-0.6264	St-Aphinv	Fu-Morsp.	0.4890	0.9564	-0.4674
Ar-Calmac	Ar-Tutabs	0.2955	0.9328	-0.6374	Ar-Erilan	Ar-Schgra	0.1167	0.9565	-0.8398
Ar-Adohon	Ar-Stedum	0.3792	0.9329	-0.5536	Ar-Eupann	Ar-Lymdis	0.4095	0.9566	-0.5471
Ar-Bomman	Ar-Lymdis	0.1880	0.9329	-0.7448	Ar-Timbar	Ar-Trivap	0.5091	0.9567	-0.4476
Ar-Osmbic	Ar-Timpop	0.3050	0.9330	-0.6279	Ar-Aulsol	Ar-Timshe	0.3725	0.9568	-0.5844
Mo-Batpla	Ar-Opebru	0.4157	0.9331	-0.5174	Ar-Partep	Ar-Timbar	0.4801	0.9569	-0.4768
Ar-Manjur	Ar-Thrpal	0.2805	0.9331	-0.6526	Ar-Bomman	Ar-Schgra	0.5803	0.9571	-0.3768
Ar-Danmel	Ar-Neopin	0.2889	0.9331	-0.6442	Ar-Phesol	Ar-Timpop	0.4998	0.9576	-0.4577
Ar-Anogla	Ar-Aptruf	0.3359	0.9332	-0.5973	Ar-Erilan	Ar-Sogfur	0.4518	0.9579	-0.5061
Ar-Erilan	Ar-Eupann	0.3039	0.9335	-0.6296	Ar-Helarm	Ar-Partep	0.6245	0.9580	-0.3334
Ar-Ladful	Ar-Nillug	0.3383	0.9338	-0.5955	Ar-Lepdec	Ar-Schgra	0.5875	0.9580	-0.3706
Ar-Partep	Ar-Schgra	0.3479	0.9338	-0.5859	Ar-Erilan	Ar-Helarm	0.6119	0.9584	-0.3465
Ar-Epacla	Ar-Helzea	0.1380	0.9338	-0.7958	Ar-Timcri	Ar-Trivap	0.5043	0.9587	-0.4544
St-Pilapi	Mo-Limfor	0.5470	0.9339	-0.3869	Ar-Ampamp	Ar-Timshe	0.4279	0.9589	-0.5309
Ar-Adohon	Ar-Partep	0.3728	0.9344	-0.5616	Ar-Lymdis	Ar-Timshe	0.4348	0.9589	-0.5242
Ar-Stedum	Ar-Vantam	0.3563	0.9345	-0.5782	Ar-Aptruf	Ar-Timshe	0.3678	0.9589	-0.5911
An-Hydele	Ar-Pseelo	0.4174	0.9346	-0.5171	Ar-Eupann	Ar-Manjur	0.4571	0.9590	-0.5019
Ar-Stedum	Ar-Trivap	0.4180	0.9351	-0.5171	Ar-Homvit	Ar-Timpop	0.3263	0.9591	-0.6327
Ar-Censcu	Ar-Lymdis	0.3783	0.9351	-0.5569	Ar-Timpop	Ar-Trivap	0.5037	0.9591	-0.4555
Ar-Phesol	Ar-Stedum	0.3314	0.9351	-0.6038	Ar-Timshe	Ar-Trivap	0.5019	0.9591	-0.4572
Ar-Adohon	Ar-Limcal	0.3066	0.9352	-0.6286	Ar-Adohon	Ar-Sipfla	0.5996	0.9591	-0.3595
Ar-Ladful	Ar-Pseelo	0.3178	0.9353	-0.6176	Ar-Acypis	Ar-Trivap	0.4490	0.9592	-0.5103
Ar-Anogla	Ar-Galmel	0.2992	0.9355	-0.6363	Ar-Ampamp	Ar-Timpop	0.4332	0.9594	-0.5262
Ar-Ampamp	Ar-Phesol	0.4733	0.9355	-0.4623	Ar-Adohon	Ar-Lepdec	0.4398	0.9601	-0.5203
Ar-Helzea	Ar-Sipfla	0.3357	0.9355	-0.5999	Ar-Bomman	Ar-Calmac	0.4498	0.9601	-0.5103
Ar-Homvit	Ar-Schgra	0.3099	0.9357	-0.6258	Ar-Sipfla	Ar-Timgen	0.4356	0.9605	-0.5249
Ar-Eupann	Ar-Manjur	0.2618	0.9357	-0.6740	Ar-Homvit	Ar-Timmon	0.3276	0.9605	-0.6329
Ar-Opebru	Ar-Timcri	0.3162	0.9360	-0.6198	Ar-Phesol	Ar-Timbar	0.5093	0.9607	-0.4514
Mo-Batpla	Ar-Neopin	0.4199	0.9361	-0.5162	Ar-Phesol	Ar-Timtah	0.5091	0.9607	-0.4515
Ar-Lephet	Ar-Schgra	0.2918	0.9362	-0.6444	Ar-Aptruf	Ar-Timmon	0.3957	0.9613	-0.5656
Ar-Bomman	Ar-Locmig	0.2810	0.9364	-0.6553	Mo-Halrub	Ar-Temlon	0.4656	0.9615	-0.4959
Ar-Helzea	Ar-Stedum	0.3634	0.9364	-0.5730	Ar-Onttau	Ar-Schgra	0.5246	0.9616	-0.4370
Ar-Homvit	Ar-Neopin	0.2776	0.9366	-0.6591	Ar-Aptruf	Ar-Timcri	0.3632	0.9618	-0.5986
Ar-Erilan	Ar-Nillug	0.3188	0.9367	-0.6179	Ar-Amytra	Ar-Eupann	0.4262	0.9618	-0.5356
Ar-Erilan	Ar-Helarm	0.3353	0.9367	-0.6014	Ar-Phesol	Ar-Timshe	0.4979	0.9618	-0.4639
Ar-Nillug	Ar-Stemim	0.3693	0.9369	-0.5675	Ar-Amytra	Ar-Aulsol	0.5099	0.9619	-0.4520
Ar-Eupuro	Ar-Partep	0.3483	0.9370	-0.5886	Ar-Aptruf	Ar-Timpop	0.3660	0.9622	-0.5962
Ar-Pseelo	Ar-Vantam	0.3345	0.9371	-0.6026	Ar-Partep	Ar-Temlon	0.4929	0.9623	-0.4694
Ar-Bomman	Ar-Manjur	0.1891	0.9371	-0.7480	Ar-Acypis	Ar-Onttau	0.5267	0.9625	-0.4358
Ar-Homvit	Ar-Osmbic	0.3168	0.9372	-0.6203	Ar-Timmon	Ar-Trivap	0.5085	0.9626	-0.4541
Ar-Lymdis	Ar-Timgen	0.3247	0.9372	-0.6125	Ar-Adohon	Ar-Onttau	0.5155	0.9626	-0.4471
Ar-Glocon	Ar-Temlon	0.2913	0.9372	-0.6459	Ar-Schgra	Ar-Timtah	0.4444	0.9628	-0.5184
Ar-Adohon	Ar-Erilan	0.3600	0.9374	-0.5774	Ar-Manjur	Ar-Temlon	0.4858	0.9629	-0.4771
Ar-Lymdis	Ar-Onttau	0.2968	0.9375	-0.6408	Ar-Limcal	Ar-Neopin	0.3948	0.9631	-0.5683
Ar-Anogla	Ar-Tutabs	0.3275	0.9376	-0.6101	Mo-Halrub	Ar-Thrpal	0.4572	0.9632	-0.5060
Mo-Halrub	Ar-Partep	0.4272	0.9378	-0.5106	Ar-Trivap	Ar-Tutabs	0.6021	0.9632	-0.3611
Ar-Homvit	Ar-Temlon	0.2899	0.9381	-0.6482	Ar-Bomman	Ar-Trivap	0.5547	0.9632	-0.4085
Ar-Acypis	Ar-Lymdis	0.3122	0.9381	-0.6260	Ar-Censcu	Ar-Onttau	0.5169	0.9633	-0.4464
An-Hydele	Ar-Acypis	0.4131	0.9384	-0.5253	Ar-Censcu	Ar-Nillug	0.5044	0.9636	-0.4592
An-Hydele	Ar-Stemim	0.4409	0.9384	-0.4975	Ar-Ladful	Ar-Stemim	0.4168	0.9640	-0.5472

Ar-Opebru	Ar-Timpop	0.3178	0.9385	-0.6207	Ar-Stedum	Ar-Timbar	0.5210	0.9641	-0.4431
Ar-Amytra	Ar-Timcri	0.3122	0.9389	-0.6267	Ar-Stedum	Ar-Timtah	0.5212	0.9641	-0.4429
Ar-Onttau	Ar-Pseelo	0.3129	0.9389	-0.6260	Ar-Anogla	Ar-Bomman	0.4939	0.9644	-0.4705
Ar-Galmel	Ar-Lephet	0.3015	0.9390	-0.6375	Ar-Homvit	Ar-Timcri	0.3276	0.9646	-0.6369
Ar-Erilan	Ar-Phesol	0.3032	0.9391	-0.6359	Ar-Acypis	Ar-Bomman	0.6038	0.9648	-0.3609
Ar-Epacla	Ar-Homvit	0.2803	0.9392	-0.6590	Ar-Anogla	Ar-Partep	0.5132	0.9650	-0.4518
Mo-Halrub	Ar-Ampamp	0.4565	0.9393	-0.4828	Ar-Pluxyl	Ar-Timgen	0.4269	0.9651	-0.5382
Ar-Amytra	Ar-Timpop	0.3203	0.9394	-0.6191	Ar-Ladful	Ar-Lymdis	0.4572	0.9652	-0.5080
Ar-Opebru	Ar-Timgen	0.3272	0.9396	-0.6124	Ar-Adohon	Ar-Nillug	0.4324	0.9653	-0.5328
Mo-Limfor	Ar-Eupann	0.3640	0.9398	-0.5757	Ar-Epacla	Ar-Osmbic	0.4531	0.9654	-0.5123
Ar-Homvit	Ar-Stedum	0.3517	0.9399	-0.5882	Ar-Phesol	Ar-Timcri	0.5036	0.9655	-0.4619
Ar-Eupuro	Ar-Limcal	0.2901	0.9399	-0.6498	Ar-Epacla	Ar-Erilan	0.5588	0.9656	-0.4068
Ar-Ampamp	Ar-Locmig	0.4360	0.9399	-0.5039	Ar-Schgra	Ar-Timbar	0.4408	0.9658	-0.5250
Ar-Chisup	Ar-Nillug	0.3045	0.9400	-0.6355	Ar-Phesol	Ar-Thrpal	0.5421	0.9659	-0.4238
Ar-Ampamp	Ar-Timcri	0.4354	0.9404	-0.5050	Ar-Osmbic	Ar-Timshe	0.3974	0.9660	-0.5686
Ar-Sogfur	Ar-Tutabs	0.2382	0.9404	-0.7022	Ar-Eupuro	Ar-Limcal	0.3941	0.9662	-0.5721
Ar-Lepdec	Ar-Neopin	0.2692	0.9408	-0.6716	Ar-Phesol	Ar-Timgen	0.5076	0.9662	-0.4586
St-Aphast	St-Phycam	0.2475	0.9408	-0.6933	Ar-Erilan	Ar-Pluxyl	0.5999	0.9664	-0.3665
Ar-Erilan	Ar-Manjur	0.3278	0.9408	-0.6131	Ar-Acypis	Ar-Stemim	0.4572	0.9664	-0.5092
Ar-Danmel	Ar-Stedum	0.3743	0.9409	-0.5666	Ar-Lymdis	Ar-Timgen	0.4399	0.9666	-0.5267
Ar-Helarm	Ar-Nillug	0.2376	0.9409	-0.7033	Ar-Limcal	Ar-Onttau	0.3197	0.9667	-0.6471
Ar-Partep	Ar-Timcri	0.3375	0.9410	-0.6035	Ar-Homvit	Ar-Timtah	0.3321	0.9671	-0.6350
Mo-Batpla	Ar-Calmac	0.3807	0.9415	-0.5609	Ar-Galmel	Ar-Schgra	0.6360	0.9672	-0.3312
Ar-Acypis	Ar-Homvit	0.2858	0.9420	-0.6562	Ar-Schgra	Ar-Timpop	0.4370	0.9674	-0.5304
Ar-Ladful	Ar-Stedum	0.3109	0.9420	-0.6312	Ar-Helarm	Ar-Temlon	0.5235	0.9675	-0.4440
Ar-Amytra	Ar-Trivap	0.4239	0.9422	-0.5183	Ar-Homvit	Ar-Timbar	0.3320	0.9676	-0.6356
Ar-Pluxyl	Ar-Thrpal	0.2400	0.9424	-0.7025	Ar-Lepdec	Ar-Stedum	0.5387	0.9678	-0.4290
Ar-Osmbic	Ar-Sitmis	0.3274	0.9425	-0.6151	Ar-Schgra	Ar-Timcri	0.4422	0.9679	-0.5257
Ar-Pseelo	Ar-Stedum	0.3612	0.9428	-0.5816	Ar-Aulsol	Ar-Calmac	0.4789	0.9686	-0.4897
Ar-Partep	Ar-Pseelo	0.3450	0.9431	-0.5981	Ar-Amytra	Ar-Osmbic	0.5121	0.9690	-0.4568
Ar-Schgra	Ar-Timgen	0.3165	0.9432	-0.6268	Ar-Erilan	Ar-Neopin	0.4171	0.9690	-0.5518
Ar-Eufmex	Ar-Glocon	0.3733	0.9435	-0.5702	Ar-Sogfur	Ar-Thrpal	0.3692	0.9690	-0.5998
An-Hydele	Ar-Timpop	0.3877	0.9438	-0.5560	Ar-Osmbic	Ar-Timtah	0.4073	0.9691	-0.5617
Ar-Onttau	Ar-Stemim	0.3413	0.9440	-0.6027	Ar-Osmbic	Ar-Timcri	0.3991	0.9691	-0.5700
Ar-Danmel	Ar-Epacla	0.1763	0.9441	-0.7678	Ar-Eupuro	Ar-Lymdis	0.4059	0.9692	-0.5633
Mo-Mermer	Ar-Thrpal	0.3756	0.9442	-0.5686	Ar-Lymdis	Ar-Temlon	0.4403	0.9694	-0.5290
An-Hydele	Ar-Timcri	0.3869	0.9442	-0.5573	Ar-Helarm	Ar-Onttau	0.5828	0.9694	-0.3866
Ar-Acypis	Ar-Helarm	0.3184	0.9445	-0.6261	Ar-Schgra	Ar-Timmon	0.4664	0.9694	-0.5030
Ar-Homvit	Ar-Vantam	0.2888	0.9446	-0.6558	Ar-Schgra	Ar-Timshe	0.4423	0.9694	-0.5271
Ar-Eupuro	Ar-Tutabs	0.2544	0.9446	-0.6901	Ar-Osmbic	Ar-Timbar	0.4040	0.9696	-0.5656
Ar-Lymdis	Ar-Timcri	0.3169	0.9447	-0.6277	Ar-Osmbic	Ar-Timpop	0.4004	0.9696	-0.5692
Ar-Epacla	Ar-Trivap	0.4116	0.9447	-0.5331	Ar-Galmel	Ar-Lepdec	0.4812	0.9698	-0.4886
Ar-Partep	Ar-Timpop	0.3340	0.9450	-0.6110	Ar-Eupuro	Ar-Manjur	0.4604	0.9703	-0.5099
Ar-Amytra	Ar-Danmel	0.1777	0.9450	-0.7673	Ar-Helarm	Ar-Lepdec	0.5873	0.9705	-0.3833
Ar-Eupuro	Ar-Pseelo	0.2814	0.9453	-0.6639	Ar-Aptruf	Ar-Erilan	0.5189	0.9708	-0.4519
Ar-Homvit	Ar-Timpop	0.2907	0.9454	-0.6547	Ar-Ampamp	Ar-Thrpal	0.4876	0.9708	-0.4832
Ar-Aptruf	Ar-Timcri	0.3026	0.9458	-0.6432	Ar-Eufmex	Ar-Temlon	0.2460	0.9711	-0.7250
Ar-Anogla	Ar-Vantam	0.3083	0.9460	-0.6377	Ar-Stedum	Ar-Timgen	0.5181	0.9711	-0.4530
Mo-Mermer	Ar-Calmac	0.3929	0.9462	-0.5534	Ar-Osmbic	Ar-Timmon	0.4362	0.9712	-0.5349
Ar-Aptruf	Ar-Timpop	0.3035	0.9463	-0.6428	Ar-Aptruf	Ar-Phesol	0.5524	0.9712	-0.4188
Ar-Calmac	Ar-Partep	0.3326	0.9463	-0.6137	Ar-Glocon	Ar-Lepdec	0.4452	0.9712	-0.5260
Ar-Calmac	Ar-Sipfla	0.3099	0.9464	-0.6365	Ar-Ladful	Ar-Pluxyl	0.4782	0.9713	-0.4931
Ar-Erilan	Ar-Helzea	0.3318	0.9466	-0.6148	Ar-Eufmex	Ar-Eupann	0.2536	0.9718	-0.7183
Ar-Phesol	Ar-Timpop	0.3506	0.9466	-0.5960	Ar-Bomman	Ar-Limcal	0.5111	0.9726	-0.4616
Ar-Ampamp	Ar-Timpop	0.4240	0.9468	-0.5228	Mo-Mermer	Ar-Ampamp	0.6318	0.9728	-0.3410
Ar-Osmbic	Ar-Phesol	0.2991	0.9468	-0.6477	Ar-Ampamp	Ar-Neopin	0.4983	0.9729	-0.4746
Ar-Lymdis	Ar-Sipfla	0.3314	0.9469	-0.6156	Ar-Eupuro	Ar-Neopin	0.2665	0.9734	-0.7070
Ar-Aptruf	Ar-Phesol	0.3433	0.9471	-0.6037	St-Pytoli	Mo-Mermer	0.6953	0.9736	-0.2783
Mo-Limfor	Ar-Ladful	0.3454	0.9471	-0.6017	Ar-Lepdec	Ar-Sogfur	0.4153	0.9737	-0.5584

Ar-Amytra	Ar-Timgen	0.3282	0.9473	-0.6191	Mo-Halrub	Ar-Glocon	0.5247	0.9738	-0.4491
Ar-Epacla	Ar-Erilan	0.3548	0.9473	-0.5925	Ar-Nillug	Ar-Timgen	0.3372	0.9746	-0.6374
Ar-Erilan	Ar-Schgra	0.1299	0.9475	-0.8176	Ar-Eupuro	Ar-Nillug	0.3427	0.9748	-0.6321
An-Hydele	Ar-Schgra	0.4126	0.9476	-0.5349	Ar-Aulsol	Ar-Bomman	0.4584	0.9749	-0.5165
Ar-Homvit	Ar-Partep	0.3366	0.9476	-0.6110	Ar-Eupuro	Ar-Lepdec	0.4066	0.9749	-0.5684
Ar-Lymdis	Ar-Timpop	0.3117	0.9476	-0.6359	Ar-Stedum	Ar-Stemim	0.0983	0.9753	-0.8770
Ar-Ladful	Ar-Schgra	0.3372	0.9481	-0.6109	Ar-Erilan	Ar-Galmel	0.5864	0.9758	-0.3894
Ar-Calmac	Ar-Danmel	0.3033	0.9484	-0.6451	Ar-Eupuro	Ar-Sipfla	0.4261	0.9760	-0.5499
Ar-Helarm	Ar-Limcal	0.3180	0.9485	-0.6305	Ar-Manjur	Ar-Timcri	0.4955	0.9766	-0.4811
Ar-Chisup	Ar-Lepdec	0.2735	0.9491	-0.6756	Ar-Manjur	Ar-Timpop	0.4951	0.9771	-0.4819
Ar-Sogfur	Ar-Stedum	0.3783	0.9492	-0.5709	Ar-Erilan	Ar-Manjur	0.6012	0.9780	-0.3767
Ar-Lymdis	Ar-Stemim	0.3393	0.9495	-0.6102	Ar-Adohon	Ar-Neopin	0.4434	0.9781	-0.5348
Ar-Bomman	Ec-Ophspi	0.3839	0.9495	-0.5656	Ar-Eupann	Ar-Pluxyl	0.3983	0.9781	-0.5798
Ar-Phesol	Ar-Timgen	0.3651	0.9497	-0.5845	Ar-Bomman	Ar-Timtah	0.4180	0.9784	-0.5604
Ar-Acypis	Ar-Locmig	0.3125	0.9497	-0.6373	Ar-Ampamp	Ar-Sipfla	0.7134	0.9786	-0.2651
Ar-Bomman	Ar-Censcu	0.3566	0.9498	-0.5932	Mo-Batpla	Ar-Amytra	0.6672	0.9786	-0.3114
Ar-Calmac	Ar-Helzea	0.2879	0.9498	-0.6620	Mo-Batpla	Ar-Onttau	0.5069	0.9787	-0.4718
Ar-Lepdec	Ar-Sogfur	0.3133	0.9501	-0.6368	Ar-Neopin	Ar-Temlon	0.2758	0.9791	-0.7033
Mo-Batpla	Ar-Eufmex	0.4224	0.9501	-0.5277	Ar-Calmac	Ar-Galmel	0.4614	0.9791	-0.5177
Ar-Acypis	Ar-Calmac	0.3148	0.9504	-0.6356	Ar-Glocon	Ar-Nillug	0.4816	0.9793	-0.4977
Ar-Erilan	Ar-Partep	0.3532	0.9506	-0.5973	Ar-Pluxyl	Ar-Stemim	0.5442	0.9796	-0.4355
Ar-Homvit	Ar-Timcri	0.2875	0.9507	-0.6632	Mo-Halrub	Ar-Eupuro	0.4390	0.9797	-0.5407
Ar-Eupann	Ar-Pseelo	0.2825	0.9508	-0.6683	St-Pilapi	Mo-Mermer	0.6699	0.9798	-0.3100
Mo-Mermer	Ar-Osmbic	0.4261	0.9510	-0.5248	Ar-Aulsol	Ar-Lepdec	0.4930	0.9799	-0.4869
Ar-Neopin	Ar-Sogfur	0.2767	0.9510	-0.6743	Ar-Adohon	Ar-Temlon	0.4355	0.9801	-0.5446
Ar-Nillug	Ar-Stedum	0.3692	0.9514	-0.5822	Ar-Manjur	Ar-Stemim	0.5768	0.9801	-0.4033
Mo-Limfor	Ar-Eupuro	0.3687	0.9514	-0.5826	Ar-Manjur	Ar-Timshe	0.4953	0.9802	-0.4848
An-Hydele	Ar-Sogfur	0.2960	0.9514	-0.6554	Ar-Ladful	Ar-Lephet	0.4189	0.9803	-0.5613
Ar-Erilan	Ar-Sogfur	0.3181	0.9521	-0.6340	Ar-Eufmex	Ar-Eupuro	0.2547	0.9806	-0.7258
Ar-Erilan	Ar-Opebru	0.3610	0.9522	-0.5912	Ar-Epacla	Ar-Temlon	0.4296	0.9807	-0.5510
Mo-Halrub	Ar-Homvit	0.3738	0.9523	-0.5784	Ar-Eupann	Ar-Limcal	0.4031	0.9812	-0.5781
Ar-Chisup	Ar-Temlon	0.3280	0.9523	-0.6243	Ar-Acypis	Ar-Ampamp	0.6860	0.9813	-0.2952
Ar-Limcal	Ar-Sipfla	0.3377	0.9523	-0.6146	Ar-Manjur	Ar-Timmon	0.4934	0.9813	-0.4879
Ar-Ladful	Ar-Lephet	0.3250	0.9528	-0.6277	Ar-Stedum	Ar-Temlon	0.5160	0.9813	-0.4653
Ar-Acypis	Ar-Danmel	0.3011	0.9528	-0.6516	Ar-Bomman	Ar-Timbar	0.4161	0.9815	-0.5654
Ar-Pluxyl	Ar-Timgen	0.3101	0.9528	-0.6427	Ar-Acypis	Ar-Lepdec	0.5963	0.9815	-0.3852
Mo-Batpla	Ar-Ampamp	0.4548	0.9528	-0.4980	Mo-Batpla	Ar-Galmel	0.6936	0.9816	-0.2880
Ar-Nillug	Ar-Trivap	0.4205	0.9530	-0.5326	Mo-Halrub	Ar-Helarm	0.5597	0.9819	-0.4222
Ar-Bomman	Ar-Pseelo	0.3208	0.9531	-0.6322	Ar-Sipfla	Ar-Trivap	0.4269	0.9825	-0.5556
Ar-Opebru	Ar-Trivap	0.4327	0.9540	-0.5213	Ar-Bomman	Ar-Timshe	0.4055	0.9830	-0.5775
Ar-Chisup	Ar-Limcal	0.3095	0.9542	-0.6446	Ar-Aulsol	Ar-Ladful	0.3664	0.9831	-0.6167
Ar-Schgra	Ar-Stemim	0.3175	0.9542	-0.6367	Ar-Ampamp	Ar-Limcal	0.6139	0.9834	-0.3695
Ar-Phesol	Ar-Timcri	0.3638	0.9545	-0.5907	Ar-Pluxyl	Ar-Timtah	0.4254	0.9838	-0.5584
Ar-Phesol	Ar-Thrpal	0.3735	0.9549	-0.5814	Ar-Pluxyl	Ar-Timshe	0.4221	0.9839	-0.5617
Ar-Acypis	Ar-Pseelo	0.3611	0.9549	-0.5938	Ar-Adohon	Ar-Bomman	0.2747	0.9839	-0.7092
Ar-Erilan	Ar-Pluxyl	0.3371	0.9549	-0.6179	Ar-Ladful	Ar-Locmig	0.3446	0.9841	-0.6395
Ar-Calmac	Ar-Nillug	0.3389	0.9555	-0.6166	Ar-Pluxyl	Ar-Timbar	0.4269	0.9844	-0.5575
Ar-Anogla	Ar-Bomman	0.3204	0.9555	-0.6351	Ar-Pluxyl	Ar-Timcri	0.4293	0.9845	-0.5552
Ar-Schgra	Ar-Stedum	0.3161	0.9555	-0.6393	Ar-Bomman	Ar-Timcri	0.4125	0.9845	-0.5720
Ar-Bomman	Ar-Trivap	0.4019	0.9556	-0.5537	Ar-Eupann	Ar-Galmel	0.4463	0.9846	-0.5383
Ar-Danmel	Ar-Temlon	0.3196	0.9557	-0.6361	Ar-Eupann	Ar-Nillug	0.3522	0.9849	-0.6327
Ar-Onttau	Ar-Sipfla	0.3015	0.9559	-0.6545	Ar-Bomman	Ar-Timpop	0.4057	0.9851	-0.5793
Ar-Eufmex	Ar-Ladful	0.3220	0.9560	-0.6340	Ar-Amytra	Ar-Eupuro	0.4222	0.9852	-0.5630
Ar-Acypis	Ar-Helzea	0.3172	0.9561	-0.6389	Ar-Lepdec	Ar-Sipfla	0.6077	0.9852	-0.3775
Ar-Locmig	Ar-Stedum	0.2996	0.9562	-0.6566	Ar-Acypis	Ar-Eupuro	0.4103	0.9853	-0.5750
Ar-Amytra	Ar-Nillug	0.2687	0.9564	-0.6877	Mo-Batpla	Ar-Stedum	0.4975	0.9853	-0.4878
Ar-Stedum	Ar-Timgen	0.3596	0.9565	-0.5969	Ar-Eupann	Ar-Sipfla	0.4257	0.9854	-0.5597
Ar-Adohon	Ar-Neopin	0.2642	0.9566	-0.6924	Ar-Erilan	Ar-Glocon	0.6264	0.9855	-0.3591
Ar-Acypis	Ar-Limcal	0.3388	0.9569	-0.6181	Ar-Acypis	Ar-Timcri	0.4406	0.9861	-0.5455

Ar-Opebru	Ar-Osmbic	0.3337	0.9570	-0.6234	Ar-Acypis	Ar-Timtah	0.4459	0.9861	-0.5402
Ar-Eupann	Ar-Lymdis	0.2531	0.9570	-0.7040	Ar-Sogfur	Ar-Temlon	0.3918	0.9861	-0.5943
Ar-Adohon	Ar-Bomman	0.1855	0.9571	-0.7715	Ar-Bomman	Ar-Timmon	0.4451	0.9861	-0.5410
Ar-Ampamp	Ar-Partep	0.4449	0.9571	-0.5123	Ar-Acypis	Ar-Timpop	0.4336	0.9866	-0.5530
Ar-Acypis	Ar-Onttau	0.3278	0.9578	-0.6300	Ar-Aulsol	Ar-Galmel	0.5178	0.9868	-0.4689
Ar-Helarm	Ar-Temlon	0.3045	0.9579	-0.6534	Ar-Manjur	Ar-Timtah	0.4947	0.9869	-0.4922
Ar-Helarm	Ar-Partep	0.3470	0.9579	-0.6110	Ar-Eupann	Ar-Locmig	0.3657	0.9870	-0.6213
Ar-Eupuro	Ar-Manjur	0.2653	0.9580	-0.6927	Mo-Mermer	Ar-Aptruf	0.5240	0.9872	-0.4631
Ar-Manjur	Ar-Vantam	0.1584	0.9580	-0.7996	Ar-Ampamp	Ar-Osmbic	0.5850	0.9873	-0.4023
An-Hydele	Ar-Ladful	0.4181	0.9584	-0.5403	Ar-Ampamp	Ar-Epacla	0.4098	0.9873	-0.5775
Ar-Neopin	Ar-Pseelo	0.2710	0.9589	-0.6879	Ar-Manjur	Ar-Timbar	0.4968	0.9874	-0.4906
Ar-Calmac	Ar-Schgra	0.3051	0.9591	-0.6540	St-Pilapi	Ec-Ophspi	0.6532	0.9874	-0.3342
Ar-Stedum	Ar-Timcri	0.3579	0.9594	-0.6015	Ar-Pluxyl	Ar-Timpop	0.4272	0.9877	-0.5605
An-Hydele	Ar-Stedum	0.4797	0.9595	-0.4798	Ar-Sipfla	Ar-Timtah	0.4462	0.9877	-0.5415
Ar-Schgra	Ar-Trivap	0.4027	0.9595	-0.5569	Ar-Pluxyl	Ar-Timmon	0.4599	0.9883	-0.5285
Ar-Glocon	Ar-Ladful	0.3305	0.9599	-0.6294	Ar-Ampamp	Ar-Aulsol	0.5555	0.9885	-0.4330
Mo-Mermer	Ar-Erilan	0.4203	0.9599	-0.5396	Ar-Lepdec	Ar-Nillug	0.4944	0.9886	-0.4942
Ar-Stedum	Ar-Timpop	0.3612	0.9600	-0.5988	Ar-Bomman	Ar-Phesol	0.5747	0.9887	-0.4140
Ar-Bomman	Ar-Timcri	0.3310	0.9601	-0.6291	Ar-Acypis	Ar-Ladful	0.4369	0.9889	-0.5519
Ar-Eupann	Ar-Partep	0.3430	0.9602	-0.6172	Ar-Aulsol	Ar-Stemim	0.4387	0.9891	-0.5504
Ar-Lymdis	Ar-Nillug	0.2692	0.9603	-0.6911	Ar-Manjur	Ar-Timgen	0.4943	0.9892	-0.4949
Ar-Erilan	Ar-Tutabs	0.3360	0.9604	-0.6244	Ar-Acypis	Ar-Timbar	0.4388	0.9893	-0.5504
Ar-Adohon	Ar-Pseelo	0.3425	0.9604	-0.6179	Ar-Helarm	Ar-Osmbic	0.5596	0.9896	-0.4300
Ar-Helzea	Ar-Nillug	0.2344	0.9605	-0.7261	Mo-Halrub	Ar-Eupann	0.4465	0.9901	-0.5437
Ar-Glocon	Ar-Timgen	0.3100	0.9605	-0.6505	Ar-Acypis	Ar-Timshe	0.4388	0.9904	-0.5515
Ar-Bomman	Ar-Timpop	0.3409	0.9606	-0.6197	Ar-Acypis	Ar-Timmon	0.4621	0.9904	-0.5283
Ar-Timgen	Ar-Vantam	0.3196	0.9607	-0.6410	Ar-Ampamp	Ar-Lymdis	0.5162	0.9905	-0.4742
Ar-Eupuro	Ar-Lymdis	0.2596	0.9607	-0.7011	Ar-Eupann	Ar-Stemim	0.4862	0.9909	-0.5046
Ar-Epacla	Ar-Osmbic	0.2998	0.9609	-0.6611	Ar-Sipfla	Ar-Timbar	0.4461	0.9909	-0.5448
Ar-Helzea	Ar-Limcal	0.3177	0.9612	-0.6435	Ar-Glocon	Ar-Partep	0.5765	0.9912	-0.4147
Ar-Danmel	Ar-Sipfla	0.3298	0.9614	-0.6316	St-Pytoli	Mo-Limfor	0.7312	0.9913	-0.2601
Ar-Trivap	Ar-Tutabs	0.4418	0.9615	-0.5197	Ar-Aptruf	Ar-Limcal	0.4979	0.9921	-0.4942
Mo-Halrub	Ar-Eupuro	0.3826	0.9615	-0.5790	Ar-Calmac	Ar-Stedum	0.5225	0.9927	-0.4702
Ar-Ladful	Ar-Lymdis	0.3163	0.9617	-0.6455	Ar-Aulsol	Ar-Glocon	0.5472	0.9933	-0.4461
Ar-Acypis	Ar-Adohon	0.3231	0.9620	-0.6389	Ar-Eupuro	Ar-Locmig	0.3737	0.9940	-0.6203
Ar-Bomman	Ar-Danmel	0.1994	0.9626	-0.7632	Ar-Galmel	Ar-Timbar	0.4408	0.9942	-0.5535
Ar-Onttau	Ar-Schgra	0.3299	0.9627	-0.6328	Ar-Galmel	Ar-Timtah	0.4407	0.9942	-0.5535
Ar-Limcal	Ar-Neopin	0.3044	0.9627	-0.6584	Ar-Bomman	Ar-Glocon	0.3828	0.9943	-0.6115
An-Hydele	Ar-Nillug	0.3033	0.9628	-0.6594	Ar-Sipfla	Ar-Timshe	0.4470	0.9946	-0.5476
Ar-Lymdis	Ar-Temlon	0.3059	0.9628	-0.6569	Ar-Eupuro	Ar-Onttau	0.4456	0.9947	-0.5490
Ar-Stemim	Ar-Vantam	0.3572	0.9628	-0.6055	Ar-Erilan	Ar-Stedum	0.5252	0.9949	-0.4697
Ar-Erilan	Ar-Glocon	0.3444	0.9631	-0.6186	Ar-Ampamp	Ar-Bomman	0.5486	0.9949	-0.4464
Ar-Acypis	Ar-Timgen	0.3177	0.9632	-0.6455	Mo-Halrub	Ar-Erilan	0.5320	0.9950	-0.4630
Mo-Batpla	Ar-Onttau	0.4386	0.9637	-0.5251	Ar-Bomman	Ar-Erilan	0.5800	0.9952	-0.4152
Ar-Galmel	Ar-Schgra	0.3095	0.9638	-0.6543	Ar-Galmel	Ar-Trivap	0.6193	0.9952	-0.3759
Ar-Amytra	Ar-Eupann	0.2560	0.9640	-0.7080	Ar-Bomman	Ar-Timgen	0.4223	0.9967	-0.5745
Ar-Eupann	Ar-Stemim	0.3377	0.9641	-0.6265	Ar-Sipfla	Ar-Timcri	0.4437	0.9968	-0.5531
An-Hydele	Ar-Vantam	0.3586	0.9643	-0.6057	Ar-Partep	Ar-Trivap	0.5345	0.9969	-0.4624
Mo-Halrub	Ar-Glocon	0.3798	0.9648	-0.5851	Ar-Neopin	Ar-Timtah	0.3575	0.9969	-0.6394
Ar-Anogla	Ar-Partep	0.3688	0.9655	-0.5967	Ar-Sipfla	Ar-Timpop	0.4417	0.9973	-0.5556
Ar-Eupann	Ar-Tutabs	0.2572	0.9656	-0.7083	Ar-Nillug	Ar-Timtah	0.3373	0.9976	-0.6603
Ar-Pluxyl	Ar-Temlon	0.2875	0.9657	-0.6781	Ar-Ladful	Ar-Limcal	0.4290	0.9976	-0.5687
Ar-Opebru	Ar-Temlon	0.2998	0.9657	-0.6660	Ar-Sipfla	Ar-Stemim	0.4179	0.9977	-0.5798
Ar-Lepdec	Ar-Schgra	0.2950	0.9658	-0.6708	Ar-Homvit	Ar-Tutabs	0.4805	0.9979	-0.5174
Ar-Helzea	Ar-Onttau	0.3218	0.9659	-0.6441	Ar-Locmig	Ar-Timmon	0.2711	0.9980	-0.7269
Ar-Bomman	Ar-Schgra	0.3172	0.9661	-0.6489	Ar-Nillug	Ar-Timbar	0.3374	0.9981	-0.6607
Ar-Nillug	Ar-Timgen	0.3312	0.9662	-0.6350	St-Pilapi	Ar-Nillug	0.6813	0.9983	-0.3170
Ar-Bomman	Ar-Sipfla	0.3290	0.9663	-0.6373	Ar-Timpop	Ar-Tutabs	0.4340	0.9984	-0.5644
Mo-Halrub	Ar-Adohon	0.3803	0.9665	-0.5861	Ar-Timshe	Ar-Tutabs	0.4342	0.9984	-0.5642

Ar-Bomman	Ar-Timgen	0.3350	0.9666	-0.6316	Ar-Erilan	Ar-Lymdis	0.5298	0.9985	-0.4686
Mo-Mermer	Ar-Aptruf	0.3521	0.9668	-0.6147	Ar-Galmel	Ar-Timcri	0.4354	0.9990	-0.5635
An-Hydele	Ar-Limcal	0.3835	0.9668	-0.5833	Ar-Galmel	Ar-Timmon	0.4703	0.9990	-0.5287
Ar-Helzea	Ar-Partep	0.3507	0.9672	-0.6166	Ar-Adohon	Ar-Stemim	0.5507	0.9990	-0.4483
An-Hydele	Ar-Opebru	0.3121	0.9676	-0.6555	Ar-Galmel	Ar-Timgen	0.4371	0.9992	-0.5621
St-Pilapi	Mo-Mermer	0.4997	0.9679	-0.4681	Ar-Neopin	Ar-Timbar	0.3577	1.0001	-0.6424
Ar-Censcu	Ar-Onttau	0.3545	0.9681	-0.6136	St-Pytoli	Ec-Ophspi	0.6817	1.0001	-0.3184
Ar-Eupann	Ar-Limcal	0.2928	0.9682	-0.6754	Ar-Eupann	Ar-Timgen	0.3583	1.0004	-0.6421
Ar-Manjur	Ar-Temlon	0.3274	0.9685	-0.6411	Ar-Ampamp	Ar-Lephet	0.5917	1.0006	-0.4089
Ar-Ladful	Ar-Pluxyl	0.3243	0.9685	-0.6442	Ar-Ampamp	Ar-Tutabs	0.3851	1.0009	-0.6158
Ar-Lepdec	Ar-Sipfla	0.3013	0.9685	-0.6672	Ar-Sipfla	Ar-Timmon	0.4723	1.0012	-0.5289
An-Hydele	Ar-Calmac	0.4088	0.9691	-0.5602	Ar-Neopin	Ar-Timgen	0.3591	1.0016	-0.6425
Ar-Pseelo	Ar-Sipfla	0.3708	0.9694	-0.5986	Ar-Ladful	Ar-Timgen	0.3438	1.0016	-0.6579
Ar-Eupuro	Ar-Opebru	0.2650	0.9694	-0.7045	Ar-Eupann	Ar-Onttau	0.4462	1.0024	-0.5561
Ar-Helarm	Ar-Lepdec	0.2747	0.9696	-0.6949	Ar-Locmig	Ar-Timbar	0.2853	1.0025	-0.7172
Ar-Temlon	Ar-Tutabs	0.2814	0.9698	-0.6884	Ar-Locmig	Ar-Timtah	0.2866	1.0025	-0.7159
Ar-Eupuro	Ar-Lepdec	0.2661	0.9707	-0.7045	Ar-Calmac	Ar-Lymdis	0.4294	1.0025	-0.5731
St-Pytoli	Mo-Limfor	0.5416	0.9709	-0.4293	Ar-Eupuro	Ar-Stemim	0.4650	1.0027	-0.5377
Ar-Adohon	Ar-Onttau	0.3093	0.9710	-0.6617	Ar-Eupann	Ar-Neopin	0.2641	1.0027	-0.7387
Ar-Acypis	Ar-Stemim	0.3345	0.9710	-0.6365	Ar-Ladful	Ar-Sipfla	0.4399	1.0030	-0.5630
Ar-Ladful	Ar-Stemim	0.3054	0.9712	-0.6658	Ar-Osmbic	Ar-Stedum	0.4869	1.0030	-0.5162
Mo-Halrub	Ar-Temlon	0.4320	0.9716	-0.5396	Ar-Galmel	Ar-Timpop	0.4356	1.0032	-0.5676
Ar-Partep	Ar-Trivap	0.4347	0.9717	-0.5369	Ar-Galmel	Ar-Timshe	0.4324	1.0032	-0.5707
An-Hydele	Ar-Pluxyl	0.3160	0.9719	-0.6559	Ar-Anogla	Ar-Ladful	0.4848	1.0033	-0.5185
Ar-Danmel	Ar-Timgen	0.3381	0.9720	-0.6339	Ar-Epacla	Ar-Eupann	0.3924	1.0035	-0.6111
Ar-Chisup	Ar-Stemim	0.3367	0.9724	-0.6357	Ar-Temlon	Ar-Trivap	0.5083	1.0036	-0.4953
Ar-Eupuro	Ar-Locmig	0.2594	0.9731	-0.7137	Ar-Neopin	Ar-Timmon	0.3605	1.0039	-0.6434
Mo-Mermer	Ar-Ampamp	0.4125	0.9734	-0.5609	Ar-Timmon	Ar-Tutabs	0.4343	1.0041	-0.5698
Ar-Ampamp	Ar-Vantam	0.4160	0.9734	-0.5574	Ar-Timbar	Ar-Tutabs	0.4382	1.0041	-0.5659
Ar-Thrpal	Ec-Ophspi	0.3544	0.9740	-0.6196	Ar-Timtah	Ar-Tutabs	0.4350	1.0041	-0.5692
Mo-Halrub	Ar-Lymdis	0.4127	0.9743	-0.5616	Ar-Helarm	Ar-Timgen	0.5039	1.0047	-0.5008
Ar-Calmac	Ar-Galmel	0.2985	0.9743	-0.6758	Ar-Nillug	Ar-Timpop	0.3434	1.0050	-0.6616
Ar-Eupuro	Ar-Sipfla	0.2914	0.9743	-0.6829	Ar-Timcri	Ar-Tutabs	0.4359	1.0050	-0.5691
Ar-Limcal	Ar-Trivap	0.4317	0.9744	-0.5427	Ar-Neopin	Ar-Timshe	0.3512	1.0055	-0.6543
Ar-Partep	Ar-Temlon	0.3570	0.9746	-0.6175	Ar-Nillug	Ar-Timcri	0.3417	1.0055	-0.6638
Ar-Danmel	Ar-Timpop	0.3248	0.9746	-0.6498	Ar-Censcu	Ar-Ladful	0.4269	1.0056	-0.5787
Ar-Eupuro	Ar-Stemim	0.3459	0.9748	-0.6289	St-Pytoli	Ar-Phesol	0.7199	1.0059	-0.2859
Ar-Anogla	Ar-Ladful	0.3488	0.9750	-0.6262	Ar-Glocon	Ar-Stemim	0.5433	1.0060	-0.4627
Ar-Glocon	Ar-Partep	0.3840	0.9750	-0.5911	Ar-Stedum	Ar-Thrpal	0.5581	1.0063	-0.4482
Ar-Calmac	Ar-Chisup	0.2917	0.9750	-0.6833	Ar-Aptruf	Ar-Locmig	0.3934	1.0063	-0.6129
Ar-Danmel	Ar-Timcri	0.3225	0.9752	-0.6526	Ar-Calmac	Ar-Glocon	0.4705	1.0064	-0.5359
Ar-Lymdis	Ar-Vantam	0.1643	0.9752	-0.8109	Ar-Glocon	Ar-Timgen	0.4491	1.0065	-0.5574
Ar-Chisup	Ar-Erilan	0.3376	0.9757	-0.6381	Ar-Manjur	Ar-Onttau	0.5049	1.0065	-0.5016
Ar-Acypis	Ar-Lepdec	0.2911	0.9758	-0.6847	Ar-Locmig	Ar-Timcri	0.2698	1.0070	-0.7373
Ar-Eupann	Ar-Locmig	0.2588	0.9761	-0.7173	Ar-Locmig	Ar-Timpop	0.2754	1.0070	-0.7316
Ar-Epacla	Ar-Temlon	0.2948	0.9761	-0.6813	Ar-Neopin	Ar-Timcri	0.3591	1.0070	-0.6479
Ar-Erilan	Ar-Neopin	0.3185	0.9763	-0.6579	Ar-Neopin	Ar-Timpop	0.3558	1.0076	-0.6518
Ar-Eupann	Ar-Schgra	0.2892	0.9765	-0.6873	Ar-Limcal	Ar-Trivap	0.5678	1.0077	-0.4399
Ar-Ladful	Ar-Timgen	0.2938	0.9765	-0.6826	Ar-Calmac	Ar-Eupann	0.4212	1.0078	-0.5866
Ar-Sitmis	Ar-Thrpal	0.3171	0.9766	-0.6594	Ar-Acypis	Ar-Eupann	0.4146	1.0080	-0.5933
Ar-Danmel	Ar-Onttau	0.3193	0.9766	-0.6573	Ar-Lepdec	Ar-Manjur	0.5069	1.0089	-0.5021
Mo-Halrub	Ar-Eupann	0.3733	0.9767	-0.6034	Ar-Glocon	Ar-Sipfla	0.6058	1.0091	-0.4034
Ar-Amytra	Ar-Osmbic	0.3002	0.9770	-0.6768	Ar-Locmig	Ar-Timgen	0.2824	1.0095	-0.7271
Ar-Neopin	Ar-Timgen	0.3054	0.9771	-0.6717	Ar-Eupann	Ar-Lepdec	0.4127	1.0097	-0.5970
Ar-Limcal	Ar-Onttau	0.2878	0.9772	-0.6894	Ar-Helarm	Ar-Thrpal	0.5272	1.0097	-0.4825
Ar-Osmbic	Ar-Pluxyl	0.3170	0.9773	-0.6603	Ar-Helarm	Ar-Ladful	0.5426	1.0098	-0.4672
Ar-Sipfla	Ar-Timgen	0.3372	0.9773	-0.6402	Ar-Pluxyl	Ar-Osmbic	0.5304	1.0101	-0.4797
Ar-Pluxyl	Ar-Stemim	0.3536	0.9777	-0.6240	Ar-Ladful	Ar-Timcri	0.3410	1.0107	-0.6697
Ar-Lepdec	Ar-Manjur	0.2865	0.9778	-0.6913	Ar-Ladful	Ar-Timtah	0.3455	1.0107	-0.6652

Ar-Bomman	Ar-Erilan	0.3155	0.9778	-0.6624	Ar-Ladful	Ar-Timbar	0.3503	1.0113	-0.6610
Ar-Calmac	Ar-Manjur	0.2781	0.9780	-0.6999	Ar-Ladful	Ar-Timpop	0.3409	1.0113	-0.6704
Ar-Ladful	Ar-Locmig	0.2802	0.9782	-0.6980	Ar-Nillug	Ar-Timshe	0.3372	1.0114	-0.6742
Mo-Halrub	Ar-Thrpal	0.3970	0.9783	-0.5813	Ar-Eupann	Ar-Glocon	0.4642	1.0117	-0.5475
An-Hydele	Ar-Eupuro	0.3844	0.9785	-0.5941	Ar-Nillug	Ar-Timmon	0.3705	1.0120	-0.6415
Ar-Pluxyl	Ar-Vantam	0.1730	0.9787	-0.8057	Ar-Locmig	Ar-Timshe	0.2769	1.0124	-0.7355
Ar-Censcu	Ar-Nillug	0.3987	0.9789	-0.5802	Ar-Ladful	Ar-Timmon	0.3424	1.0125	-0.6700
Ar-Ampamp	Ar-Neopin	0.4169	0.9792	-0.5623	Ar-Erilan	Ar-Trivap	0.4391	1.0129	-0.5738
Ar-Adohon	Ar-Sipfla	0.3611	0.9793	-0.6182	Ar-Timgen	Ar-Tutabs	0.4385	1.0129	-0.5744
Ar-Bomman	Ar-Eupann	0.2739	0.9794	-0.7055	Ar-Ladful	Ar-Timshe	0.3425	1.0134	-0.6709
St-Pilapi	Ec-Ophspi	0.4468	0.9795	-0.5327	Ar-Acypis	Ar-Temlon	0.4472	1.0137	-0.5665
Ar-Erilan	Ar-Stedum	0.3340	0.9798	-0.6458	Ar-Eupuro	Ar-Pluxyl	0.3904	1.0139	-0.6235
Ar-Bomman	Ar-Limcal	0.3082	0.9800	-0.6717	Ar-Ampamp	Ar-Pluxyl	0.4653	1.0142	-0.5489
Mo-Halrub	Ar-Vantam	0.3987	0.9806	-0.5819	Ar-Nillug	Ar-Temlon	0.3934	1.0144	-0.6210
Ar-Helarm	Ar-Onttau	0.3246	0.9808	-0.6562	Ar-Osmbic	Ar-Sipfla	0.4949	1.0145	-0.5196
Ar-Galmel	Ar-Lepdec	0.2756	0.9810	-0.7054	Ar-Acypis	Ar-Glocon	0.6163	1.0146	-0.3983
St-Pytoli	Ar-Phesol	0.5593	0.9814	-0.4220	Ar-Helarm	Ar-Timtah	0.5003	1.0146	-0.5143
Ar-Helzea	Ar-Temlon	0.3053	0.9818	-0.6765	Ar-Helarm	Ar-Timbar	0.5020	1.0152	-0.5132
Ar-Calmac	Ar-Lymdis	0.2864	0.9818	-0.6954	Ar-Helarm	Ar-Timshe	0.4979	1.0152	-0.5173
Ar-Ampamp	Ar-Limcal	0.4132	0.9820	-0.5687	Ar-Aptruf	Ar-Stemim	0.4478	1.0169	-0.5690
Ar-Helarm	Ar-Osmbic	0.3144	0.9821	-0.6677	Ar-Bomman	Ar-Eupann	0.4190	1.0174	-0.5984
Ar-Ampamp	Ar-Lymdis	0.4266	0.9824	-0.5559	Mo-Batpla	Ar-Eupann	0.5384	1.0179	-0.4795
Mo-Batpla	Ar-Amytra	0.4044	0.9825	-0.5781	Ar-Bomman	Ar-Temlon	0.4378	1.0194	-0.5816
Ar-Eufmex	Ar-Temlon	0.2797	0.9825	-0.7029	Ar-Calmac	Ar-Eupuro	0.4259	1.0194	-0.5935
An-Hydele	Ar-Manjur	0.3450	0.9829	-0.6379	Ar-Adohon	Ar-Calmac	0.4804	1.0195	-0.5391
Ar-Ampamp	Ar-Osmbic	0.4504	0.9832	-0.5329	Ar-Schgra	Ar-Thrpal	0.5295	1.0196	-0.4902
Ar-Schgra	Ar-Timpop	0.3128	0.9835	-0.6708	Ar-Homvit	Ar-Ladful	0.3767	1.0199	-0.6433
Ar-Eupuro	Ar-Galmel	0.2828	0.9836	-0.7008	Ar-Calmac	Ar-Sogfur	0.4225	1.0208	-0.5983
Ar-Neopin	Ar-Timcri	0.3078	0.9836	-0.6758	Ar-Eupuro	Ar-Stedum	0.5203	1.0213	-0.5010
Ar-Neopin	Ar-Temlon	0.2638	0.9838	-0.7200	Mo-Batpla	Ar-Thrpal	0.5811	1.0213	-0.4402
An-Hydele	Ar-Eupann	0.3750	0.9840	-0.6090	Ar-Helarm	Ar-Timmon	0.5394	1.0215	-0.4821
Ar-Schgra	Ar-Timcri	0.3139	0.9841	-0.6702	Ar-Ampamp	Ar-Schgra	0.6731	1.0218	-0.3488
Ar-Neopin	Ar-Timpop	0.3125	0.9841	-0.6716	Ar-Sipfla	Ar-Temlon	0.4424	1.0219	-0.5795
Ar-Bomman	Ar-Phesol	0.3086	0.9844	-0.6758	Ar-Helarm	Ar-Timpop	0.5013	1.0221	-0.5208
Ar-Epacla	Ar-Eupann	0.2396	0.9845	-0.7449	Ar-Thrpal	Ar-Timgen	0.3580	1.0223	-0.6643
Ar-Ampamp	Ar-Thrpal	0.3479	0.9847	-0.6368	St-Pilapi	Ar-Phesol	0.7006	1.0226	-0.3220
Ar-Locmig	Ar-Timgen	0.2796	0.9848	-0.7052	Mo-Halrub	Ar-Osmbic	0.4840	1.0237	-0.5398
Mo-Batpla	Ar-Stedum	0.4361	0.9848	-0.5487	Ar-Amytra	Ar-Sogfur	0.4417	1.0242	-0.5825
Ar-Timpop	Ar-Vantam	0.3258	0.9851	-0.6593	Ar-Calmac	Ar-Homvit	0.4197	1.0243	-0.6046
Ar-Eupann	Ar-Sipfla	0.2844	0.9853	-0.7009	Ar-Thrpal	Ar-Tutabs	0.3986	1.0250	-0.6264
St-Pytoli	Mo-Mermer	0.5049	0.9857	-0.4808	Ar-Bomman	Ar-Lepdec	0.5135	1.0252	-0.5117
Ar-Chisup	Ar-Osmbic	0.2847	0.9858	-0.7011	Ar-Neopin	Ar-Osmbic	0.2558	1.0255	-0.7698
Ar-Manjur	Ar-Stemim	0.3502	0.9858	-0.6356	Ar-Helarm	Ar-Timcri	0.5053	1.0255	-0.5202
Ar-Eufmex	Ar-Eupann	0.2835	0.9864	-0.7028	Ar-Aptruf	Ar-Lephet	0.4453	1.0261	-0.5808
Ar-Acypis	Ar-Trivap	0.4100	0.9865	-0.5765	Ar-Eupuro	Ar-Timgen	0.3615	1.0261	-0.6646
Mo-Halrub	Ar-Helzea	0.3886	0.9870	-0.5984	Ar-Glocon	Ar-Timtah	0.4404	1.0264	-0.5861
St-Phycam	Fn-Morsp.	0.3809	0.9873	-0.6064	Ar-Glocon	Ar-Timbar	0.4423	1.0271	-0.5848
Mo-Batpla	Ar-Galmel	0.4214	0.9875	-0.5660	Mo-Batpla	Ar-Bomman	0.6477	1.0271	-0.3795
Ar-Helarm	Ar-Timgen	0.3044	0.9875	-0.6831	Mo-Batpla	Ar-Eupuro	0.5276	1.0273	-0.4997
Ar-Stedum	Ar-Temlon	0.3955	0.9879	-0.5924	Ar-Aulsol	Ar-Thrpal	0.4365	1.0285	-0.5919
Ar-Amytra	Ar-Eupuro	0.2472	0.9880	-0.7407	Ar-Eupuro	Ar-Temlon	0.2594	1.0288	-0.7694
Ar-Glocon	Ar-Timcri	0.3024	0.9880	-0.6857	Ar-Bomman	Ar-Osmbic	0.4984	1.0292	-0.5307
Ar-Timcri	Ar-Vantam	0.3124	0.9883	-0.6758	Ar-Aptruf	Ar-Thrpal	0.3289	1.0297	-0.7008
Ar-Eupann	Ar-Onttau	0.2778	0.9886	-0.7107	Ar-Manjur	Ar-Osmbic	0.5449	1.0301	-0.4852
Ar-Lepdec	Ar-Stedum	0.3175	0.9893	-0.6717	Ar-Aulsol	Ar-Temlon	0.3894	1.0303	-0.6409
Ar-Locmig	Ar-Timcri	0.2779	0.9893	-0.7114	Ar-Onttau	Ar-Timgen	0.4306	1.0303	-0.5997
Ar-Locmig	Ar-Timpop	0.2798	0.9893	-0.7095	Ar-Glocon	Ar-Timcri	0.4435	1.0309	-0.5874
Ar-Ampamp	Ar-Sipfla	0.4304	0.9894	-0.5590	Ar-Glocon	Ar-Timmon	0.4705	1.0309	-0.5604
Ar-Erilan	Ar-Lymdis	0.3222	0.9894	-0.6672	Ar-Glocon	Ar-Timshe	0.4417	1.0309	-0.5892

Ar-Aptruf	Ar-Erilan	0.3329	0.9899	-0.6570	Ar-Ampamp	Ar-Stedum	0.6592	1.0316	-0.3724
Ar-Locmig	Ar-Vantam	0.2918	0.9899	-0.6982	Ar-Anogla	Ar-Thrpal	0.4396	1.0320	-0.5925
Ar-Calmac	Ar-Stedum	0.3465	0.9899	-0.6434	Ar-Eupann	Ar-Timcri	0.3692	1.0323	-0.6631
Ar-Erilan	Ar-Trivap	0.4228	0.9903	-0.5675	Ar-Adohon	Ar-Aptruf	0.4580	1.0324	-0.5743
Ar-Nillug	Ar-Timpop	0.3225	0.9903	-0.6679	Ar-Erilan	Ar-Onttau	0.4911	1.0327	-0.5417
Ar-Acypis	Ar-Ampamp	0.4022	0.9905	-0.5883	Ar-Glocon	Ar-Stedum	0.6032	1.0328	-0.4296
Ar-Censcu	Ar-Ladful	0.3285	0.9907	-0.6621	Ar-Ampamp	Ar-Onttau	0.6527	1.0336	-0.3809
Ar-Nillug	Ar-Timcri	0.3221	0.9908	-0.6688	Ar-Ampamp	Ar-Ladful	0.5249	1.0338	-0.5088
Ar-Acypis	Ar-Bomman	0.3265	0.9914	-0.6649	Ar-Eupann	Ar-Timtah	0.3555	1.0345	-0.6790
Ar-Helzea	Ar-Lepdec	0.2686	0.9917	-0.7231	Ar-Glocon	Ar-Timpop	0.4415	1.0347	-0.5933
Ar-Eupann	Ar-Neopin	0.2225	0.9918	-0.7692	St-Aphinv	Fu-Lobtra	0.5383	1.0348	-0.4965
Ar-Erilan	Ar-Galmel	0.3141	0.9918	-0.6777	St-Pytoli	Mo-Batpla	0.7664	1.0349	-0.2685
Ar-Eufmex	Ar-Eupuro	0.2878	0.9923	-0.7046	Ar-Aptruf	Ar-Censcu	0.5350	1.0349	-0.4999
St-Pilapi	Ar-Phesol	0.5336	0.9926	-0.4590	Ar-Eupann	Ar-Timbar	0.3523	1.0350	-0.6827
Ar-Eupuro	Ar-Onttau	0.2833	0.9928	-0.7095	Ar-Ladful	Ar-Sogfur	0.3294	1.0350	-0.7056
Ar-Helzea	Ar-Osmbic	0.3115	0.9929	-0.6814	Ar-Bomman	Ar-Eupuro	0.4146	1.0361	-0.6215
Ar-Sogfur	Ar-Thrpal	0.2776	0.9933	-0.7157	Mo-Batpla	Ar-Ladful	0.5820	1.0361	-0.4541
Ar-Adohon	Ar-Temlon	0.2906	0.9938	-0.7032	Ar-Glocon	Ar-Schgra	0.6083	1.0363	-0.4279
Ar-Sipfla	Ar-Stemim	0.3346	0.9940	-0.6593	Ar-Aptruf	Ar-Calmac	0.4246	1.0363	-0.6117
Ar-Sogfur	Ar-Temlon	0.2748	0.9944	-0.7196	Ar-Aptruf	Ar-Temlon	0.4454	1.0373	-0.5919
Ar-Ladful	Ar-Timcri	0.2959	0.9944	-0.6985	Ar-Eupuro	Ar-Glocon	0.4635	1.0373	-0.5738
Ar-Nillug	Ar-Vantam	0.2884	0.9946	-0.7062	Ar-Onttau	Ar-Temlon	0.4633	1.0374	-0.5741
Ar-Ampamp	Ar-Lephet	0.3983	0.9946	-0.5963	St-Pytoli	Ar-Nillug	0.6723	1.0377	-0.3654
Ar-Ampamp	Ar-Epacla	0.3762	0.9948	-0.6186	Ar-Osmbic	Ar-Schgra	0.4529	1.0377	-0.5848
Ar-Manjur	Ar-Timgen	0.3263	0.9949	-0.6686	Mo-Halrub	Ar-Manjur	0.6065	1.0383	-0.4317
Ar-Ladful	Ar-Timpop	0.3018	0.9950	-0.6932	St-Aphinv	Mo-Halrub	0.5806	1.0383	-0.4578
Ar-Censcu	Ar-Vantam	0.3881	0.9951	-0.6071	St-Aphste	Mo-Halrub	0.5843	1.0383	-0.4540
Ar-Nillug	Ar-Thrpal	0.2774	0.9953	-0.7180	St-Pilapi	Mo-Halrub	0.5828	1.0388	-0.4560
Ar-Glocon	Ar-Timpop	0.3049	0.9953	-0.6904	Ar-Eupann	Ar-Temlon	0.2597	1.0391	-0.7794
Ar-Temlon	Ar-Trivap	0.4689	0.9956	-0.5266	Ar-Eupann	Ar-Timpop	0.3660	1.0393	-0.6734
Ar-Galmel	Ar-Trivap	0.4079	0.9959	-0.5879	St-Pilapi	Ar-Anogla	0.7094	1.0394	-0.3300
Ar-Danmel	Ar-Vantam	0.1683	0.9961	-0.8278	Ar-Ladful	Ar-Trivap	0.4852	1.0394	-0.5542
Ar-Acypis	Ar-Ladful	0.3434	0.9963	-0.6528	St-Pilapi	Ar-Limcal	0.7796	1.0402	-0.2606
Ar-Adohon	Ar-Aptruf	0.2670	0.9963	-0.7293	Ar-Ladful	Ar-Temlon	0.4153	1.0402	-0.6249
Ar-Aptruf	Ar-Lepdec	0.2862	0.9972	-0.7110	Ar-Eupann	Ar-Sogfur	0.3248	1.0404	-0.7156
Ar-Bomman	Ar-Lepdec	0.2851	0.9973	-0.7121	Ar-Censcu	Ar-Thrpal	0.5073	1.0405	-0.5331
Mo-Halrub	Ar-Helarm	0.3902	0.9978	-0.6076	Ar-Eupann	Ar-Timmon	0.3676	1.0412	-0.6736
Mo-Halrub	Ar-Danmel	0.3782	0.9979	-0.6198	Ar-Eupann	Ar-Timshe	0.3627	1.0415	-0.6788
Ar-Ladful	Ar-Limcal	0.3457	0.9988	-0.6531	Ar-Sipfla	Ar-Thrpal	0.5663	1.0415	-0.4752
Ar-Manjur	Ar-Timcri	0.3131	0.9991	-0.6859	Ar-Eupann	Ar-Stedum	0.5299	1.0416	-0.5117
Ar-Acypis	Ar-Eupuro	0.2935	0.9992	-0.7057	Ar-Aptruf	Ar-Lepdec	0.4897	1.0417	-0.5520
Ar-Danmel	Ar-Stemim	0.3756	0.9996	-0.6239	Ar-Nillug	Ar-Osmbic	0.4463	1.0419	-0.5956
Ar-Manjur	Ar-Timpop	0.3295	0.9996	-0.6701	Ar-Helarm	Ar-Stemim	0.6052	1.0438	-0.4387
Ar-Sogfur	Ar-Vantam	0.3031	0.9997	-0.6966	Ar-Thrpal	Ar-Timtah	0.3459	1.0443	-0.6983
Ar-Ampamp	Ar-Bomman	0.4052	0.9998	-0.5946	Ar-Epacla	Ar-Eupuro	0.3884	1.0447	-0.6563
Ar-Glocon	Ar-Stemim	0.3807	0.9999	-0.6192	Ar-Adohon	Ar-Eupuro	0.4202	1.0448	-0.6246
Ar-Lepdec	Ar-Nillug	0.3202	1.0001	-0.6799	Ar-Erilan	Ar-Thrpal	0.5188	1.0462	-0.5274
Ar-Adohon	Ar-Stemim	0.3666	1.0002	-0.6336	Ne-Caeele	Ar-Locmig	0.6066	1.0469	-0.4402
Ar-Adohon	Ar-Calmac	0.3023	1.0004	-0.6981	Ne-Caenig	Ar-Locmig	0.5877	1.0469	-0.4591
Ar-Eupann	Ar-Pluxyl	0.2525	1.0010	-0.7485	Ar-Thrpal	Ar-Timbar	0.3461	1.0475	-0.7014
Ar-Trivap	Ar-Vantam	0.3969	1.0014	-0.6045	Ar-Amytra	Ar-Locmig	0.5152	1.0476	-0.5323
Ar-Acypis	Ar-Timcri	0.3170	1.0015	-0.6846	Ar-Calmac	Ar-Trivap	0.5529	1.0477	-0.4948
Ar-Eupuro	Ar-Schgra	0.2950	1.0016	-0.7067	St-Pilapi	Mo-Batpla	0.7588	1.0482	-0.2894
Ar-Stedum	Ar-Stemim	0.0323	1.0019	-0.9696	Ar-Eupann	Ar-Helarm	0.5029	1.0486	-0.5457
Mo-Batpla	Ar-Eupann	0.3926	1.0020	-0.6094	Ar-Acypis	Ar-Thrpal	0.5370	1.0497	-0.5127
Ar-Ampamp	Ar-Pluxyl	0.3615	1.0020	-0.6405	St-Aphste	Ar-Pluxyl	0.5670	1.0501	-0.4831
Ar-Acypis	Ar-Timpop	0.3087	1.0021	-0.6934	Ar-Aptruf	Ar-Pluxyl	0.5116	1.0513	-0.5397
Ar-Epacla	Ar-Eupuro	0.2373	1.0023	-0.7651	Ar-Thrpal	Ar-Timpop	0.3543	1.0519	-0.6975
An-Hydele	Ar-Glocon	0.3036	1.0026	-0.6991	Ar-Bomman	Ar-Sogfur	0.4252	1.0519	-0.6267

Ar-Eupuro	Ar-Nillug	0.2775	1.0036	-0.7261	Ar-Thrpal	Ar-Timcri	0.3546	1.0524	-0.6978
St-Pytoli	Ec-Ophspi	0.4106	1.0038	-0.5932	Ar-Thrpal	Ar-Timshe	0.3530	1.0524	-0.6994
Ar-Eupann	Ar-Lepdec	0.2633	1.0042	-0.7409	Ar-Ladful	Ar-Osmbic	0.3965	1.0528	-0.6563
Ar-Stedum	Ar-Thrpal	0.3599	1.0047	-0.6448	Ar-Ampamp	Ar-Nillug	0.4843	1.0531	-0.5688
St-Pilapi	Ar-Sitmis	0.4631	1.0062	-0.5430	Ar-Galmel	Ar-Osmbic	0.5276	1.0533	-0.5257
Ar-Manjur	Ar-Onttau	0.2946	1.0064	-0.7118	Ar-Partep	Ar-Thrpal	0.5292	1.0541	-0.5248
Ar-Sipfla	Ar-Trivap	0.4204	1.0065	-0.5861	Ar-Onttau	Ar-Osmbic	0.4572	1.0543	-0.5970
Ar-Opebru	Ar-Thrpal	0.2626	1.0066	-0.7440	Ar-Ampamp	Ar-Anogla	0.5502	1.0547	-0.5045
Ar-Calmac	Ar-Eupann	0.2740	1.0072	-0.7331	Ar-Adohon	Ar-Eupann	0.4250	1.0554	-0.6303
Ar-Eupuro	Ar-Neopin	0.2254	1.0073	-0.7819	Mo-Limfor	Ne-Caeele	0.6775	1.0555	-0.3780
Ar-Chisup	Ar-Onttau	0.3056	1.0078	-0.7022	Mo-Limfor	Ne-Caenig	0.6690	1.0555	-0.3865
Ar-Nillug	Ar-Temlon	0.2960	1.0081	-0.7122	Ar-Aptruf	Ar-Osmbic	0.4883	1.0555	-0.5672
Ar-Danmel	Ar-Erilan	0.3147	1.0087	-0.6940	Ar-Eupuro	Ar-Helarm	0.4967	1.0564	-0.5597
Ar-Calmac	Ar-Vantam	0.2919	1.0090	-0.7171	St-Pytoli	Ar-Lymdis	0.6009	1.0565	-0.4557
St-Pilapi	Ar-Pseelo	0.5205	1.0092	-0.4886	Ar-Schgra	Ar-Temlon	0.4417	1.0571	-0.6154
An-Hydele	Ar-Bomman	0.3813	1.0097	-0.6285	Ar-Glocon	Ar-Osmbic	0.5206	1.0575	-0.5369
Ar-Sipfla	Ar-Timcri	0.3281	1.0102	-0.6821	Ar-Thrpal	Ar-Timmon	0.3932	1.0576	-0.6643
Ar-Eupuro	Ar-Stedum	0.3434	1.0104	-0.6670	St-Aphste	Fu-Morsp.	0.4721	1.0579	-0.5859
Ar-Glocon	Ar-Nillug	0.2492	1.0106	-0.7614	Ar-Osmbic	Ar-Trivap	0.4364	1.0588	-0.6224
Ar-Eupann	Ar-Timgen	0.3156	1.0106	-0.6950	Ar-Eupuro	Ar-Timcri	0.3724	1.0593	-0.6869
Ar-Sipfla	Ar-Timpop	0.3261	1.0108	-0.6847	Ar-Amytra	Ar-Thrpal	0.4647	1.0600	-0.5953
Ar-Galmel	Ar-Timgen	0.3096	1.0111	-0.7014	Ar-Eupann	Ar-Homvit	0.3511	1.0606	-0.7095
Ar-Aptruf	Ar-Sitmis	0.3051	1.0117	-0.7066	Ar-Eupuro	Ar-Sogfur	0.3152	1.0608	-0.7456
An-Hydele	Ar-Lepdec	0.4006	1.0119	-0.6114	Ar-Eupuro	Ar-Timtah	0.3586	1.0615	-0.7029
Ar-Ampamp	Ar-Tutabs	0.3454	1.0123	-0.6669	Ar-Eupuro	Ar-Timbar	0.3555	1.0621	-0.7066
Ar-Pluxyl	Ar-Timcri	0.2982	1.0127	-0.7145	Ar-Aulsol	Ar-Osmbic	0.3436	1.0622	-0.7186
Ar-Ladful	Ar-Sogfur	0.3307	1.0129	-0.6822	Ar-Galmel	Ar-Thrpal	0.4792	1.0629	-0.5837
Ar-Manjur	Ar-Osmbic	0.2989	1.0131	-0.7142	Ar-Eupuro	Ar-Timshe	0.3644	1.0633	-0.6989
Ar-Homvit	Ar-Tutabs	0.2596	1.0133	-0.7537	Ar-Eupann	Ec-Ophspi	0.5175	1.0636	-0.5461
Ar-Aptruf	Ar-Locmig	0.2708	1.0135	-0.7427	Ar-Homvit	Ar-Onttau	0.4671	1.0639	-0.5968
Ar-Calmac	Ar-Glocon	0.3188	1.0137	-0.6949	St-Pilapi	Ar-Lephet	0.6892	1.0654	-0.3762
Ar-Osmbic	Ar-Stedum	0.3325	1.0143	-0.6818	St-Aphste	Fu-Lobtra	0.4977	1.0655	-0.5679
Ar-Erilan	Ar-Onttau	0.3302	1.0146	-0.6844	Ar-Eupuro	Ar-Timpop	0.3677	1.0666	-0.6989
Fn-Lobtra	Mo-Halrub	0.4806	1.0151	-0.5345	Ar-Osmbic	Ar-Thrpal	0.4944	1.0666	-0.5723
Ar-Helarm	Ar-Ladful	0.3131	1.0151	-0.7020	Ar-Adohon	Ar-Timgen	0.4294	1.0671	-0.6376
Ar-Limcal	Ar-Vantam	0.3134	1.0152	-0.7018	Ar-Eupuro	Ar-Homvit	0.3627	1.0675	-0.7048
Ar-Eupann	Ar-Stedum	0.3341	1.0154	-0.6814	Ar-Eupuro	Ar-Osmbic	0.2543	1.0680	-0.8137
Ar-Ampamp	Ar-Chisup	0.4256	1.0156	-0.5901	Ar-Eupuro	Ec-Ophspi	0.5104	1.0681	-0.5577
Ar-Aptruf	Ar-Calmac	0.2954	1.0157	-0.7202	Ar-Epacla	Ar-Thrpal	0.4018	1.0683	-0.6665
Ar-Timpop	Ar-Tutabs	0.3023	1.0157	-0.7134	Ar-Limcal	Ar-Thrpal	0.4731	1.0684	-0.5953
Ar-Ladful	Ar-Trivap	0.4292	1.0158	-0.5866	Ar-Eupuro	Ar-Timmon	0.3708	1.0685	-0.6977
Ar-Galmel	Ar-Timcri	0.3135	1.0159	-0.7025	Ar-Partep	Ar-Osmbic	0.4519	1.0693	-0.6174
Ar-Bomman	Ar-Temlon	0.3057	1.0160	-0.7103	Ar-Osmbic	Ar-Sogfur	0.3715	1.0697	-0.6982
St-Aphste	Fn-Morsp.	0.3475	1.0160	-0.6685	St-Pytoli	Ar-Locmig	0.6880	1.0698	-0.3817
St-Pilapi	Ar-Nillug	0.4229	1.0160	-0.5931	Ar-Onttau	Ar-Trivap	0.5509	1.0698	-0.5188
Ar-Pluxyl	Ar-Timpop	0.2988	1.0161	-0.7172	Ar-Aptruf	Ar-Galmel	0.5394	1.0699	-0.5305
An-Hydele	Ar-Chisup	0.3865	1.0163	-0.6298	St-Pilapi	Ar-Timgen	0.6556	1.0709	-0.4153
Ar-Bomman	Ar-Eupuro	0.2656	1.0167	-0.7511	Ar-Acypis	Ar-Osmbic	0.4696	1.0712	-0.6015
Ar-Eupann	Ec-Ophspi	0.3628	1.0167	-0.6539	St-Pilapi	Ar-Timtah	0.6597	1.0715	-0.4118
Ar-Acypis	Ar-Vantam	0.3176	1.0170	-0.6994	Ar-Ampamp	Ar-Aptruf	0.5639	1.0725	-0.5086
Ar-Thrpal	Ar-Tutabs	0.2679	1.0174	-0.7495	Ar-Ampamp	Ar-Homvit	0.4934	1.0728	-0.5794
Fn-Morsp.	Mo-Halrub	0.5190	1.0177	-0.4987	St-Aphste	Ar-Ampamp	0.6058	1.0732	-0.4674
Ar-Glocon	Ar-Stedum	0.3885	1.0178	-0.6293	St-Phytub	Ec-Ophspi	0.6803	1.0732	-0.3929
Ar-Chisup	Ar-Ladful	0.3215	1.0178	-0.6963	Ar-Aptruf	Ar-Aulsol	0.4201	1.0739	-0.6538
Ar-Eupann	Ar-Galmel	0.2886	1.0179	-0.7294	St-Pilapi	Ar-Timshe	0.6503	1.0740	-0.4237
Ar-Ladful	Ar-Temlon	0.3572	1.0183	-0.6611	Ar-Eupann	Ar-Osmbic	0.2612	1.0740	-0.8129
Ar-Bomman	Ar-Osmbic	0.2921	1.0185	-0.7263	St-Aphinv	Mo-Limfor	0.7320	1.0746	-0.3426
Ar-Anogla	Ar-Thrpal	0.3702	1.0190	-0.6488	St-Pytoli	Ar-Pluxyl	0.5786	1.0746	-0.4960
Mo-Batpla	Ar-Eupuro	0.4033	1.0193	-0.6160	Ar-Eupann	Ar-Ladful	0.3790	1.0747	-0.6957

Ar-Onttau	Ar-Timgen	0.3399	1.0199	-0.6799	St-Pilapi	Ar-Lepdec	0.6859	1.0748	-0.3889
Mo-Batpla	Ar-Thrpal	0.4317	1.0200	-0.5883	St-Pilapi	Ar-Timbar	0.6593	1.0753	-0.4160
Ar-Ladful	Ar-Sipfla	0.3326	1.0201	-0.6875	Ar-Onttau	Ar-Timcri	0.4534	1.0758	-0.6224
Ar-Galmel	Ar-Timpop	0.3135	1.0202	-0.7066	Ar-Erilan	Ar-Ladful	0.4551	1.0759	-0.6208
Ar-Helarm	Ar-Timpop	0.2877	1.0208	-0.7331	Ar-Onttau	Ar-Timpop	0.4516	1.0764	-0.6249
Ar-Timgen	Ar-Tutabs	0.3217	1.0215	-0.6998	Ar-Onttau	Ar-Timshe	0.4512	1.0764	-0.6252
Ar-Sipfla	Ar-Vantam	0.3269	1.0218	-0.6949	Ar-Ampamp	Ar-Trivap	0.5919	1.0765	-0.4846
Ar-Calmac	Ar-Eupuro	0.2733	1.0219	-0.7486	Ar-Lymdis	Ar-Osmbic	0.4339	1.0767	-0.6428
Ar-Ampamp	Ar-Onttau	0.4584	1.0220	-0.5635	St-Pilapi	Ar-Sipfla	0.6945	1.0769	-0.3824
An-Hydele	Ar-Temlon	0.3509	1.0223	-0.6714	St-Pytoli	Ar-Acypis	0.7280	1.0770	-0.3489
Ar-Timcri	Ar-Tutabs	0.3132	1.0224	-0.7092	St-Pilapi	Ar-Timcri	0.6521	1.0771	-0.4250
Ar-Calmac	Ar-Trivap	0.4452	1.0229	-0.5777	Ar-Ampamp	Ar-Censcu	0.5952	1.0775	-0.4823
Mo-Batpla	Ar-Ladful	0.3845	1.0232	-0.6387	Ar-Onttau	Ar-Timtah	0.4419	1.0775	-0.6356
Ar-Glocon	Ar-Sipfla	0.3444	1.0233	-0.6789	St-Pilapi	Ar-Timpop	0.6478	1.0778	-0.4300
Ar-Eupuro	Ar-Timgen	0.3207	1.0234	-0.7027	St-Pilapi	Ar-Neopin	0.6764	1.0780	-0.4015
Ar-Acypis	Ar-Eupann	0.2916	1.0237	-0.7320	St-Pytoli	Ar-Anogla	0.6421	1.0780	-0.4359
Ar-Adohon	Ar-Nillug	0.2447	1.0237	-0.7790	Ar-Onttau	Ar-Timbar	0.4422	1.0781	-0.6360
St-Pytoli	Ar-Nillug	0.4060	1.0238	-0.6178	St-Pilapi	Ar-Censcu	0.6958	1.0782	-0.3824
Ar-Ampamp	Ar-Ladful	0.4511	1.0241	-0.5730	Ar-Ampamp	Ar-Amytra	0.5086	1.0783	-0.5697
Ar-Helarm	Ar-Timcri	0.2965	1.0242	-0.7277	Ar-Aptruf	Ar-Eufmex	0.4935	1.0797	-0.5863
Mo-Halrub	Ar-Erilan	0.4411	1.0245	-0.5834	St-Pytoli	Ar-Manjur	0.6182	1.0802	-0.4620
Ar-Thrpal	Ar-Timgen	0.3008	1.0247	-0.7238	St-Pilapi	Ar-Timmon	0.6876	1.0803	-0.3927
An-Hydele	Ar-Lymdis	0.3735	1.0251	-0.6516	St-Pilapi	Ar-Eufmex	0.6761	1.0804	-0.4043
Ar-Acypis	Ar-Glocon	0.3156	1.0251	-0.7096	Ar-Aptruf	Ar-Helarm	0.5798	1.0806	-0.5007
St-Aphast	Fn-Morsp.	0.3576	1.0252	-0.6676	Ar-Onttau	Ar-Timmon	0.4763	1.0809	-0.6045
Ar-Eupann	Ar-Nillug	0.2667	1.0253	-0.7586	Ar-Aptruf	Ar-Nillug	0.4877	1.0814	-0.5937
Ar-Helzea	Ar-Timgen	0.3088	1.0259	-0.7171	St-Aphinv	Ar-Locmig	0.6816	1.0826	-0.4011
An-Hydele	Ar-Helzea	0.3292	1.0261	-0.6969	Ar-Lepdec	Ar-Thrpal	0.4542	1.0829	-0.6287
Ar-Eupuro	Ec-Ophspi	0.3670	1.0265	-0.6595	Ar-Ampamp	Ar-Stemim	0.5842	1.0853	-0.5011
Ar-Eupuro	Ar-Pluxyl	0.2537	1.0267	-0.7730	St-Pilapi	Ar-Locmig	0.7002	1.0858	-0.3856
Ar-Eupann	Ar-Vantam	0.2464	1.0273	-0.7809	Ar-Adohon	Ar-Timtah	0.4315	1.0874	-0.6559
Ar-Aptruf	Ar-Lephet	0.2913	1.0273	-0.7360	Ar-Adohon	Ar-Timbar	0.4297	1.0881	-0.6584
St-Pilapi	Mo-Batpla	0.5278	1.0278	-0.5000	Ar-Temlon	Ar-Thrpal	0.4124	1.0886	-0.6762
Ar-Neopin	Ar-Osmbic	0.2409	1.0282	-0.7873	Ar-Adohon	Ar-Osmbic	0.4904	1.0893	-0.5989
Ar-Sipfla	Ar-Temlon	0.3660	1.0283	-0.6623	Ar-Adohon	Ar-Timpop	0.4251	1.0895	-0.6644
Ar-Acypis	Ar-Temlon	0.3677	1.0283	-0.6606	Ar-Adohon	Ar-Timshe	0.4220	1.0903	-0.6682
Ar-Helarm	Ar-Thrpal	0.2658	1.0290	-0.7631	St-Pilapi	Ar-Tutabs	0.6019	1.0915	-0.4896
Ar-Helzea	Ar-Ladful	0.3094	1.0291	-0.7197	St-Pytoli	Ar-Tutabs	0.5805	1.0926	-0.5121
Ar-Bomman	Ar-Glocon	0.2594	1.0294	-0.7700	Ar-Ampamp	Ar-Erilan	0.6382	1.0928	-0.4546
St-Pytoli	Mo-Batpla	0.5289	1.0294	-0.5005	Ar-Glocon	Ar-Onttau	0.5568	1.0936	-0.5368
St-Physyr	Fn-Morsp.	0.3305	1.0297	-0.6992	Ar-Adohon	Ar-Timcri	0.4238	1.0937	-0.6699
Ar-Aptruf	Ar-Limcal	0.3218	1.0297	-0.7079	Ar-Adohon	Ar-Timmon	0.4337	1.0937	-0.6600
Ar-Adohon	Ar-Timgen	0.3102	1.0306	-0.7204	St-Pytoli	Ar-Sipfla	0.7260	1.0947	-0.3687
An-Hydele	Ar-Helarm	0.3303	1.0308	-0.7006	St-Pilapi	Ar-Acypis	0.7146	1.0960	-0.3815
An-Hydele	Ar-Aptruf	0.3766	1.0312	-0.6546	St-Aphast	Fu-Morsp.	0.4749	1.0964	-0.6215
Ar-Eupann	Ar-Glocon	0.2598	1.0312	-0.7714	St-Phycam	Fu-Morsp.	0.4943	1.0966	-0.6023
Mo-Batpla	Ar-Bomman	0.4082	1.0313	-0.6231	Ar-Onttau	Ar-Sogfur	0.4518	1.0970	-0.6452
St-Aphste	Ar-Pluxyl	0.4404	1.0318	-0.5913	Ar-Eupuro	Ar-Ladful	0.3779	1.0971	-0.7192
Ar-Homvit	Ar-Ladful	0.3205	1.0323	-0.7118	Ar-Adohon	Ar-Ampamp	0.4204	1.0972	-0.6768
An-Hydele	Ar-Adohon	0.3060	1.0332	-0.7271	Fu-Morsp.	Ar-Ladful	0.6272	1.0976	-0.4704
Ar-Aptruf	Ar-Pluxyl	0.2670	1.0343	-0.7673	Ar-Lephet	Ar-Thrpal	0.4554	1.0986	-0.6432
Ar-Ampamp	Ar-Schgra	0.4067	1.0344	-0.6277	Ar-Ampamp	Ar-Helarm	0.5700	1.0990	-0.5290
Ar-Eupuro	Ar-Vantam	0.2479	1.0347	-0.7868	Fu-Lobtra	Mo-Halrub	0.5516	1.1000	-0.5484
An-Hydele	Ar-Galmel	0.3790	1.0348	-0.6558	Ar-Neopin	Ar-Thrpal	0.3782	1.1002	-0.7220
St-Aphinv	Fn-Lobtra	0.3816	1.0349	-0.6533	Ar-Ampamp	Ar-Sogfur	0.4750	1.1014	-0.6263
Ar-Eupuro	Ar-Glocon	0.2647	1.0353	-0.7706	Mo-Mermer	Ne-Caeele	0.6090	1.1017	-0.4928
Ar-Amytra	Ar-Sogfur	0.2734	1.0354	-0.7620	Mo-Mermer	Ne-Caenig	0.6234	1.1017	-0.4783
Ar-Aptruf	Ar-Stemim	0.3205	1.0357	-0.7152	Fu-Lobtra	Ar-Eufmex	0.6717	1.1024	-0.4307
St-Pilapi	Ar-Timgen	0.5055	1.0370	-0.5314	Fu-Morsp.	Mo-Halrub	0.5569	1.1035	-0.5466

Ar-Helzea	Ar-Thrpal	0.2620	1.0370	-0.7750	Ar-Onttau	Ar-Thrpal	0.4574	1.1037	-0.6463
Ar-Schgra	Ar-Thrpal	0.3159	1.0370	-0.7211	Ar-Ampamp	Ar-Eupann	0.5115	1.1037	-0.5922
Ar-Helarm	Ar-Stemim	0.3531	1.0371	-0.6841	St-Phytub	Mo-Halrub	0.5639	1.1046	-0.5408
Ar-Aptruf	Ar-Thrpal	0.1814	1.0374	-0.8560	St-Physyr	Fu-Morsp.	0.4785	1.1057	-0.6272
Ar-Ampamp	Ar-Stedum	0.4420	1.0375	-0.5955	Ar-Eupann	Ar-Trivap	0.4889	1.1061	-0.6172
Ar-Osmbic	Ar-Partep	0.3448	1.0375	-0.6927	Ar-Calmac	Ar-Onttau	0.3084	1.1069	-0.7985
St-Pytoli	Ar-Sitmis	0.4880	1.0380	-0.5500	Ar-Stemim	Ar-Thrpal	0.5157	1.1076	-0.5918
Ar-Erilan	Ar-Vantam	0.3265	1.0383	-0.7119	St-Physyr	Mo-Halrub	0.5681	1.1100	-0.5419
Ar-Glocon	Ar-Osmbic	0.3333	1.0387	-0.7054	Ar-Aptruf	Ar-Manjur	0.5117	1.1113	-0.5995
Ar-Schgra	Ar-Temlon	0.3544	1.0389	-0.6846	Fu-Morsp.	Ar-Tutabs	0.5635	1.1125	-0.5491
Mo-Limfor	Ne-Caeele	0.4388	1.0391	-0.6003	Fu-Morsp.	Ar-Pluxyl	0.5903	1.1132	-0.5228
Mo-Limfor	Ne-Caenig	0.4645	1.0391	-0.5746	Ar-Calmac	Ar-Ladful	0.4477	1.1132	-0.6654
Ar-Aptruf	Ar-Censcu	0.3781	1.0393	-0.6612	St-Phytub	Ar-Phesol	0.6919	1.1133	-0.4214
Ar-Chisup	Ar-Eupuro	0.2948	1.0398	-0.7450	St-Pilapi	Ar-Partep	0.6322	1.1139	-0.4817
An-Hydele	Ar-Onttau	0.3992	1.0399	-0.6407	Mo-Halrub	Ne-Caeele	0.5482	1.1140	-0.5658
An-Hydele	Ar-Trivap	0.4557	1.0401	-0.5844	Mo-Halrub	Ne-Caenig	0.5545	1.1140	-0.5595
Ar-Amytra	Ar-Thrpal	0.2791	1.0404	-0.7613	St-Pytoli	Ar-Eufmex	0.6956	1.1165	-0.4209
Ar-Osmbic	Ar-Schgra	0.3151	1.0408	-0.7258	St-Pytoli	Ar-Homvit	0.6438	1.1167	-0.4729
Ar-Chisup	Ar-Timgen	0.3267	1.0410	-0.7143	St-Pytoli	Ar-Ampamp	0.5821	1.1173	-0.5351
Ar-Calmac	Ar-Homvit	0.2866	1.0417	-0.7551	Ar-Ampamp	Ar-Temlon	0.5120	1.1194	-0.6074
Ar-Ampamp	Ar-Anogla	0.4478	1.0418	-0.5940	St-Aphste	Ar-Osmbic	0.6567	1.1196	-0.4629
Ar-Lepdec	Ar-Vantam	0.2867	1.0428	-0.7561	Ar-Ampamp	Ar-Manjur	0.4708	1.1197	-0.6489
Ar-Eupuro	Ar-Temlon	0.2608	1.0433	-0.7825	St-Pytoli	Ar-Osmbic	0.6981	1.1215	-0.4233
Ar-Ampamp	Ar-Aptruf	0.3841	1.0433	-0.6593	Fu-Morsp.	Ar-Ampamp	0.5977	1.1221	-0.5245
Ar-Ampamp	Ar-Opebru	0.3389	1.0435	-0.7046	St-Physyr	Ar-Pluxyl	0.5726	1.1227	-0.5501
Ar-Ampamp	Ar-Stemim	0.4650	1.0446	-0.5796	St-Pytoli	Ar-Epacla	0.5684	1.1233	-0.5548
Ar-Onttau	Ar-Temlon	0.3395	1.0451	-0.7056	St-Pilapi	Ar-Sogfur	0.6869	1.1236	-0.4367
Ar-Aptruf	Ar-Nillug	0.2926	1.0461	-0.7535	Ar-Ladful	Ar-Lepdec	0.4548	1.1237	-0.6690
St-Pytoli	Ar-Lymdis	0.5024	1.0466	-0.5442	St-Pytoli	Ar-Schgra	0.7485	1.1241	-0.3756
St-Pilapi	Mo-Halrub	0.5863	1.0466	-0.4603	St-Aphast	Fu-Lobtra	0.5215	1.1241	-0.6026
Ar-Eupuro	Ar-Helarm	0.2387	1.0471	-0.8084	Ne-Caeele	Ec-Ophspi	0.6444	1.1248	-0.4804
Mo-Halrub	Ar-Manjur	0.3966	1.0481	-0.6515	Ne-Caenig	Ec-Ophspi	0.6403	1.1248	-0.4845
Ar-Danmel	Ar-Eupann	0.2475	1.0481	-0.8006	St-Aphinv	Ar-Eufmex	0.6925	1.1258	-0.4333
Ar-Temlon	Ar-Vantam	0.3225	1.0486	-0.7261	St-Aphinv	Ar-Pluxyl	0.6092	1.1266	-0.5174
Ar-Danmel	Ar-Eupuro	0.2528	1.0492	-0.7965	St-Pilapi	Ar-Homvit	0.6620	1.1267	-0.4647
Ar-Bomman	Ar-Sogfur	0.2813	1.0497	-0.7684	St-Phycam	Mo-Limfor	0.7305	1.1274	-0.3969
Ar-Osmbic	Ar-Sipfla	0.3076	1.0498	-0.7422	Ar-Eupuro	Ar-Trivap	0.4607	1.1279	-0.6672
Ar-Osmbic	Ar-Trivap	0.4124	1.0502	-0.6377	Ar-Eupuro	Ar-Thrpal	0.3700	1.1280	-0.7580
Ar-Ampamp	Ar-Censcu	0.5069	1.0503	-0.5434	Ar-Aptruf	Ar-Schgra	0.5497	1.1286	-0.5790
Ar-Amytra	Ar-Locmig	0.2970	1.0503	-0.7534	St-Aphste	Ar-Manjur	0.6285	1.1290	-0.5004
Ar-Eupann	Ar-Sogfur	0.2738	1.0511	-0.7774	St-Pytoli	Mo-Halrub	0.5653	1.1300	-0.5648
St-Pilapi	Ar-Anogla	0.5140	1.0513	-0.5374	Ne-Caeele	Ar-Censcu	0.6275	1.1305	-0.5030
Ar-Eupann	Ar-Helarm	0.2448	1.0516	-0.8068	Ne-Caenig	Ar-Censcu	0.6581	1.1305	-0.4724
Ar-Helzea	Ar-Stemim	0.3568	1.0535	-0.6968	St-Pytoli	Ar-Eupann	0.5896	1.1312	-0.5416
Ar-Aptruf	Ar-Pseelo	0.3391	1.0536	-0.7146	Ar-Ampamp	Ar-Galmel	0.4996	1.1317	-0.6321
Ne-Caeele	Ar-Locmig	0.4664	1.0539	-0.5875	St-Aphste	Mo-Limfor	0.7079	1.1323	-0.4244
Ne-Caenig	Ar-Locmig	0.4753	1.0539	-0.5786	Ar-Aptruf	Ar-Epacla	0.4386	1.1323	-0.6937
Ar-Neopin	Ar-Thrpal	0.3017	1.0541	-0.7524	St-Pilapi	Ar-Trivap	0.6574	1.1330	-0.4756
Ar-Ampamp	Ar-Trivap	0.5154	1.0545	-0.5391	Ar-Aptruf	Ar-Sogfur	0.4083	1.1337	-0.7254
Ar-Calmac	Ar-Sogfur	0.3310	1.0551	-0.7241	Ar-Ampamp	Ar-Lepdec	0.5780	1.1338	-0.5559
Ar-Chisup	Ar-Timcri	0.3093	1.0554	-0.7461	St-Pilapi	Ar-Onttau	0.7406	1.1346	-0.3939
Ar-Ampamp	Ar-Sogfur	0.3076	1.0555	-0.7479	St-Pytoli	Ar-Eupuro	0.5851	1.1358	-0.5507
Ar-Helzea	Ar-Timpop	0.2936	1.0556	-0.7620	St-Aphste	Ec-Ophspi	0.7158	1.1361	-0.4203
An-Hydele	Ar-Danmel	0.3389	1.0559	-0.7170	Ne-Caeele	Ar-Amytra	0.5934	1.1362	-0.5427
Ar-Pseelo	Ar-Thrpal	0.3434	1.0564	-0.7130	Ne-Caenig	Ar-Amytra	0.5952	1.1362	-0.5410
Ar-Adohon	Ar-Eupuro	0.2756	1.0565	-0.7809	St-Pilapi	Ar-Osmbic	0.7109	1.1364	-0.4256
Mo-Halrub	Ar-Osmbic	0.4576	1.0568	-0.5992	St-Pytoli	Ar-Ladful	0.7217	1.1369	-0.4151
Ar-Eupuro	Ar-Sogfur	0.2827	1.0570	-0.7743	St-Phytub	Mo-Limfor	0.7054	1.1380	-0.4325
Ar-Ladful	Ar-Osmbic	0.2904	1.0574	-0.7670	Ar-Ladful	Ar-Thrpal	0.4041	1.1385	-0.7344

Ar-Adohon	Ar-Vantam	0.1832	1.0576	-0.8744	St-Aphast	Ar-Phesol	0.6800	1.1388	-0.4588
Ar-Erilan	Ar-Thrpal	0.3411	1.0577	-0.7165	St-Pilapi	Ar-Bomman	0.7349	1.1405	-0.4056
St-Pytoli	Ar-Locmig	0.5385	1.0580	-0.5194	Mo-Batpla	Ar-Aptruf	0.5669	1.1407	-0.5738
Ar-Eupann	Ar-Ladful	0.2954	1.0585	-0.7630	St-Pilapi	Ar-Ladful	0.7412	1.1412	-0.4001
Ar-Erilan	Ar-Ladful	0.3403	1.0585	-0.7182	Ne-Caeele	Ar-Eufmex	0.6165	1.1422	-0.5256
Ar-Onttau	Ar-Osmbic	0.2943	1.0585	-0.7643	Ne-Caenig	Ar-Eufmex	0.6192	1.1422	-0.5230
St-Aphste	Fn-Lobtra	0.3477	1.0585	-0.7109	Ar-Eupann	Ar-Thrpal	0.3665	1.1427	-0.7762
Ar-Helzea	Ar-Timcri	0.3013	1.0590	-0.7577	Ar-Aptruf	Ar-Tutabs	0.4554	1.1436	-0.6882
Ar-Nillug	Ar-Osmbic	0.3096	1.0591	-0.7495	St-Pilapi	Ar-Manjur	0.6471	1.1442	-0.4971
Ar-Ampamp	Ar-Nillug	0.3154	1.0592	-0.7438	Ar-Ampamp	Ar-Eupuro	0.5073	1.1454	-0.6381
St-Aphinv	Mo-Halrub	0.5674	1.0602	-0.4928	St-Pilapi	Ar-Stedum	0.6967	1.1454	-0.4487
Ar-Ampamp	Ar-Homvit	0.3594	1.0604	-0.7010	St-Aphste	St-Phytub	0.2892	1.1456	-0.8564
Ar-Chisup	Ar-Timpop	0.3231	1.0604	-0.7373	Fu-Morsp.	Ar-Anogla	0.6307	1.1457	-0.5150
Ar-Osmbic	Ar-Sogfur	0.3081	1.0604	-0.7522	Ar-Aptruf	Ar-Trivap	0.4600	1.1481	-0.6881
Ar-Thrpal	Ar-Timpop	0.3085	1.0604	-0.7519	St-Pilapi	Ar-Helarm	0.7132	1.1487	-0.4355
Ar-Onttau	Ar-Trivap	0.4227	1.0605	-0.6378	Fu-Morsp.	Ar-Adohon	0.5562	1.1488	-0.5925
Ar-Chisup	Ar-Eupann	0.2914	1.0606	-0.7692	Ne-Caeele	Ar-Galmel	0.6112	1.1497	-0.5385
Ar-Eupann	Ar-Temlon	0.2577	1.0606	-0.8030	Ne-Caenig	Ar-Galmel	0.6099	1.1497	-0.5398
Ar-Thrpal	Ar-Timcri	0.3005	1.0610	-0.7605	Ne-Caeele	Ar-Lymdis	0.5882	1.1498	-0.5616
Ar-Eupuro	Ar-Homvit	0.2700	1.0617	-0.7917	Ne-Caenig	Ar-Lymdis	0.5965	1.1498	-0.5532
Ar-Eupuro	Ar-Helzea	0.2344	1.0617	-0.8274	Fu-Lobtra	Ar-Anogla	0.6382	1.1507	-0.5125
Ar-Eupann	Ar-Timcri	0.3066	1.0622	-0.7556	St-Pytoli	Ar-Thrpal	0.5808	1.1507	-0.5699
St-Aphinv	Mo-Limfor	0.5895	1.0626	-0.4731	St-Pilapi	Ar-Calmac	0.7109	1.1509	-0.4400
Ar-Eupann	Ar-Helzea	0.2417	1.0631	-0.8215	St-Phytub	Ar-Pluxyl	0.5748	1.1520	-0.5772
An-Hydele	Ar-Osmbic	0.4116	1.0635	-0.6519	St-Pytoli	Ar-Amytra	0.6100	1.1528	-0.5428
Ar-Homvit	Ar-Onttau	0.3166	1.0648	-0.7483	St-Pilapi	Ar-Amytra	0.6825	1.1538	-0.4713
Ar-Ampamp	Ar-Helzea	0.3596	1.0654	-0.7058	St-Physyr	Mo-Limfor	0.7027	1.1551	-0.4525
Ar-Eupuro	Ar-Timcri	0.3080	1.0663	-0.7583	St-Aphste	Ar-Locmig	0.6976	1.1557	-0.4581
Ar-Sipfla	Ar-Thrpal	0.3259	1.0664	-0.7405	St-Pilapi	Ar-Erilan	0.6985	1.1560	-0.4575
Fn-Morsp.	Ar-Ampamp	0.4346	1.0665	-0.6319	Ar-Acypis	Ar-Aptruf	0.5784	1.1564	-0.5781
St-Aphinv	Ar-Pluxyl	0.4414	1.0670	-0.6256	St-Pilapi	Ar-Eupann	0.6447	1.1574	-0.5127
Ar-Danmel	Ar-Ladful	0.3197	1.0673	-0.7476	St-Aphinv	Ar-Amytra	0.6459	1.1583	-0.5124
Ar-Aptruf	Ar-Temlon	0.3266	1.0681	-0.7414	Ar-Aptruf	Ar-Neopin	0.4181	1.1590	-0.7408
Ar-Eupann	Ar-Timpop	0.3097	1.0695	-0.7598	St-Pilapi	Ar-Stemim	0.6241	1.1610	-0.5369
St-Pytoli	Ar-Manjur	0.5116	1.0696	-0.5580	Ar-Ampamp	Ar-Glocon	0.4603	1.1612	-0.7010
Ar-Glocon	Ar-Schgra	0.3167	1.0699	-0.7533	Fu-Morsp.	Ar-Epacla	0.5738	1.1620	-0.5881
Ar-Aptruf	Ar-Eufmex	0.3415	1.0701	-0.7286	Ar-Aptruf	Ar-Homvit	0.3831	1.1629	-0.7798
Ar-Galmel	Ar-Osmbic	0.2993	1.0711	-0.7718	Ne-Caeele	Ar-Phesol	0.6856	1.1631	-0.4775
Ar-Onttau	Ar-Timcri	0.3432	1.0715	-0.7283	Ne-Caenig	Ar-Phesol	0.6724	1.1631	-0.4907
Ar-Galmel	Ar-Thrpal	0.3099	1.0717	-0.7618	St-Aphste	Ar-Thrpal	0.6003	1.1636	-0.5633
St-Pytoli	Ar-Danmel	0.4564	1.0719	-0.6155	St-Pilapi	Ar-Ampamp	0.6295	1.1642	-0.5347
Ar-Onttau	Ar-Timpop	0.3497	1.0721	-0.7224	Ar-Ampamp	Ar-Calmac	0.5311	1.1657	-0.6346
Ar-Aptruf	Ar-Helzea	0.2545	1.0722	-0.8177	St-Pytoli	Ar-Helarm	0.6528	1.1659	-0.5130
Ar-Adohon	Ar-Timcri	0.3045	1.0727	-0.7682	Ar-Aptruf	Ar-Sipfla	0.6033	1.1672	-0.5639
Ar-Ampamp	Ar-Helarm	0.3616	1.0730	-0.7114	Ne-Caeele	Ar-Ladful	0.6260	1.1673	-0.5413
Ar-Adohon	Ar-Timpop	0.2994	1.0734	-0.7740	Ne-Caenig	Ar-Ladful	0.6343	1.1673	-0.5330
Ar-Eupuro	Ar-Timpop	0.3140	1.0738	-0.7597	St-Pilapi	Ar-Epacla	0.6193	1.1695	-0.5502
Ar-Lymdis	Ar-Osmbic	0.3003	1.0747	-0.7743	St-Pytoli	Ar-Sogfur	0.6609	1.1696	-0.5087
St-Pilapi	Ar-Lephet	0.4823	1.0747	-0.5924	St-Aphste	Ar-Limcal	0.6850	1.1698	-0.4849
Ar-Adohon	Ar-Eupann	0.2746	1.0758	-0.8012	St-Pytoli	Ar-Onttau	0.7180	1.1703	-0.4524
St-Pilapi	Ar-Eufmex	0.5447	1.0761	-0.5314	St-Aphast	Mo-Halrub	0.5842	1.1709	-0.5866
Ar-Partep	Ar-Thrpal	0.3384	1.0762	-0.7379	Fu-Morsp.	Ar-Lephet	0.6530	1.1727	-0.5197
Ar-Epacla	Ar-Thrpal	0.2815	1.0765	-0.7950	St-Phycam	Ec-Ophspi	0.6620	1.1730	-0.5110
St-Aphste	Ar-Ampamp	0.4646	1.0767	-0.6121	St-Aphste	Ar-Trivap	0.7073	1.1734	-0.4660
St-Pytoli	Ar-Pluxyl	0.4309	1.0767	-0.6458	Ar-Aptruf	Ar-Stedum	0.4951	1.1735	-0.6784
Ar-Osmbic	Ar-Thrpal	0.3415	1.0768	-0.7353	St-Pilapi	Ar-Temlon	0.6816	1.1740	-0.4924
Ar-Ampamp	Ar-Danmel	0.3875	1.0770	-0.6895	Fu-Morsp.	Ec-Ophspi	0.6756	1.1744	-0.4988
Fn-Lobtra	Mo-Limfor	0.4939	1.0770	-0.5831	St-Aphinv	Ar-Phesol	0.6496	1.1755	-0.5258
Ar-Aptruf	Ar-Helarm	0.2578	1.0771	-0.8193	St-Pytoli	Ar-Censcu	0.6807	1.1755	-0.4948

Ar-Limcal	Ar-Thrpal	0.3235	1.0782	-0.7547	St-Phycam	Ar-Ampamp	0.5134	1.1761	-0.6626
Ar-Eupuro	Ar-Ladful	0.2960	1.0784	-0.7823	St-Physyr	Mo-Batpla	0.7559	1.1762	-0.4203
St-Pilapi	Ar-Neopin	0.4890	1.0784	-0.5894	St-Aphinv	Ec-Ophspi	0.7386	1.1763	-0.4376
Fn-Lobtra	Ar-Eufmex	0.5158	1.0786	-0.5627	St-Pytoli	Ar-Neopin	0.6400	1.1773	-0.5373
Ar-Ampamp	Ar-Amytra	0.4075	1.0789	-0.6715	Ar-Aptruf	Ar-Partep	0.5135	1.1777	-0.6643
Ar-Ampamp	Ar-Erilan	0.4121	1.0793	-0.6672	St-Pytoli	Ar-Trivap	0.6838	1.1780	-0.4942
Mo-Mermer	Ne-Caeele	0.4626	1.0794	-0.6168	St-Pilapi	Ar-Eupuro	0.6259	1.1780	-0.5521
Mo-Mermer	Ne-Caenig	0.4940	1.0794	-0.5854	Fu-Morsp.	Ar-Limcal	0.6730	1.1780	-0.5050
St-Pilapi	Ar-Limcal	0.4677	1.0795	-0.6118	St-Aphste	Ar-Aulsol	0.6280	1.1794	-0.5514
St-Pilapi	Ar-Sipfla	0.4973	1.0795	-0.5822	St-Aphste	Ar-Temlon	0.6643	1.1806	-0.5164
St-Aphinv	Ar-Opebru	0.4719	1.0803	-0.6084	St-Pytoli	Ar-Lephet	0.6555	1.1819	-0.5264
Ar-Schgra	Ar-Vantam	0.3132	1.0820	-0.7688	Ne-Caeele	Ar-Bomman	0.5832	1.1819	-0.5987
Fn-Morsp.	Ar-Acypis	0.4493	1.0832	-0.6339	Ne-Caenig	Ar-Bomman	0.5920	1.1819	-0.5899
Fn-Morsp.	Ar-Anogla	0.4995	1.0834	-0.5839	Ne-Caeele	Ar-Neopin	0.5484	1.1832	-0.6348
Ar-Osmbic	Ar-Vantam	0.2964	1.0838	-0.7874	Ne-Caenig	Ar-Neopin	0.5440	1.1832	-0.6392
Fn-Morsp.	Ar-Pluxyl	0.3650	1.0839	-0.7189	St-Aphste	Ar-Schgra	0.7195	1.1838	-0.4643
Ar-Eupann	Ar-Homvit	0.2649	1.0842	-0.8193	St-Pilapi	Ar-Aulsol	0.6224	1.1845	-0.5621
St-Aphinv	Ar-Locmig	0.5580	1.0844	-0.5264	St-Pilapi	Ar-Pluxyl	0.6351	1.1846	-0.5495
St-Pytoli	Ar-Homvit	0.4511	1.0847	-0.6336	St-Phytub	Ar-Amytra	0.6256	1.1847	-0.5591
Ne-Caeele	Ar-Bomman	0.4093	1.0850	-0.6758	St-Aphste	Ar-Sogfur	0.6263	1.1847	-0.5584
Ne-Caenig	Ar-Bomman	0.4153	1.0850	-0.6697	St-Pilapi	Ar-Schgra	0.7157	1.1850	-0.4693
Fn-Morsp.	Ar-Sitmis	0.4503	1.0858	-0.6355	Fu-Morsp.	Ar-Neopin	0.6109	1.1854	-0.5745
Fn-Morsp.	Ar-Ladful	0.4667	1.0863	-0.6196	St-Aphste	Ar-Aptruf	0.6759	1.1864	-0.5106
St-Pytoli	Ar-Acypis	0.4859	1.0865	-0.6006	St-Aphinv	Mo-Batpla	0.7133	1.1865	-0.4732
St-Pytoli	Ar-Epacla	0.4588	1.0868	-0.6280	St-Pytoli	Ar-Temlon	0.6496	1.1866	-0.5370
Fn-Morsp.	Ar-Chisup	0.4437	1.0871	-0.6434	St-Aphinv	Ar-Anogla	0.6888	1.1870	-0.4982
St-Pilapi	Ar-Tutabs	0.4365	1.0872	-0.6507	St-Aphste	Ar-Homvit	0.6775	1.1873	-0.5098
Ar-Ladful	Ar-Lepdec	0.3207	1.0882	-0.7675	St-Pytoli	Ar-Aulsol	0.6583	1.1874	-0.5291
St-Aphste	Mo-Halrub	0.5792	1.0887	-0.5095	Ar-Amytra	Ar-Aptruf	0.5424	1.1875	-0.6451
St-Pilapi	Ar-Locmig	0.5128	1.0891	-0.5764	Fu-Morsp.	Ar-Lymdis	0.5956	1.1878	-0.5921
St-Pilapi	Ar-Timcri	0.5137	1.0902	-0.5765	St-Phytub	Ar-Locmig	0.6715	1.1878	-0.5163
Ar-Onttau	Ar-Thrpal	0.3523	1.0908	-0.7385	Ar-Aptruf	Ar-Glocon	0.4821	1.1892	-0.7071
St-Pilapi	Ar-Timpop	0.5116	1.0908	-0.5792	St-Aphinv	Ar-Tutabs	0.5884	1.1915	-0.6031
Fn-Morsp.	Ar-Sipfla	0.4706	1.0909	-0.6203	St-Aphste	Ar-Nillug	0.6384	1.1920	-0.5536
Ar-Ampamp	Ar-Lepdec	0.4230	1.0920	-0.6690	Fu-Morsp.	Ar-Timcri	0.5835	1.1930	-0.6095
Ar-Aptruf	Ar-Galmel	0.2954	1.0930	-0.7976	Ar-Aptruf	Ar-Lymdis	0.4585	1.1946	-0.7362
Ar-Calmac	Ar-Onttau	0.2821	1.0933	-0.8112	Ne-Caeele	Ar-Tutabs	0.5978	1.1954	-0.5976
Ar-Acypis	Ar-Thrpal	0.3138	1.0934	-0.7796	Ne-Caenig	Ar-Tutabs	0.5807	1.1954	-0.6147
St-Phytub	Ec-Ophspi	0.4714	1.0936	-0.6223	St-Physyr	Ar-Phesol	0.7194	1.1962	-0.4767
Fn-Morsp.	Ar-Epacla	0.3887	1.0936	-0.7050	St-Pytoli	Ar-Bomman	0.6356	1.1976	-0.5619
St-Pytoli	Ar-Anogla	0.5375	1.0946	-0.5570	St-Pytoli	Ar-Galmel	0.6043	1.1983	-0.5939
Ar-Adohon	Ar-Ampamp	0.3255	1.0947	-0.7692	Ne-Caeele	Ar-Sogfur	0.5689	1.1984	-0.6295
Ar-Adohon	Ar-Osmbic	0.3196	1.0949	-0.7753	Ne-Caenig	Ar-Sogfur	0.5617	1.1984	-0.6368
St-Pytoli	Ar-Tutabs	0.4322	1.0950	-0.6628	Mo-Batpla	Ne-Caeele	0.7098	1.1991	-0.4893
Ar-Eupuro	Ar-Osmbic	0.2401	1.0951	-0.8551	Mo-Batpla	Ne-Caenig	0.7129	1.1991	-0.4862
Fn-Morsp.	Ar-Calmac	0.4866	1.0960	-0.6094	Fu-Morsp.	Ar-Timpop	0.5708	1.1993	-0.6285
Ar-Censcu	Ar-Thrpal	0.4292	1.0964	-0.6672	St-Phytub	Ar-Epacla	0.5804	1.1994	-0.6190
St-Pytoli	Ar-Osmbic	0.5326	1.0972	-0.5646	St-Pilapi	Ar-Galmel	0.6778	1.1995	-0.5218
Ar-Eupann	Ar-Trivap	0.4115	1.0977	-0.6862	St-Aphinv	Ar-Galmel	0.6183	1.1997	-0.5814
Ar-Aptruf	Ar-Chisup	0.3037	1.0992	-0.7955	Fu-Morsp.	Ar-Timbar	0.5819	1.2005	-0.6186
Ne-Caeele	Ec-Ophspi	0.4217	1.0993	-0.6776	Fu-Morsp.	Ar-Timtah	0.5755	1.2005	-0.6251
Ne-Caenig	Ec-Ophspi	0.4397	1.0993	-0.6596	St-Aphinv	Ar-Onttau	0.7175	1.2009	-0.4834
Ar-Aptruf	Ar-Osmbic	0.3214	1.0995	-0.7781	St-Pytoli	Ar-Calmac	0.6962	1.2022	-0.5060
Ar-Acypis	Ar-Osmbic	0.3132	1.0996	-0.7864	St-Physyr	Ec-Ophspi	0.6545	1.2024	-0.5479
Fn-Morsp.	Ar-Adohon	0.3747	1.0999	-0.7252	Fu-Morsp.	Ar-Timmon	0.5842	1.2034	-0.6192
St-Aphinv	Ar-Eufmex	0.5549	1.1000	-0.5451	Ne-Caeele	Ar-Onttau	0.6142	1.2035	-0.5893
St-Pytoli	Ar-Sipfla	0.5050	1.1001	-0.5951	Ne-Caenig	Ar-Onttau	0.6240	1.2035	-0.5795
Ne-Caeele	Ar-Ladful	0.4554	1.1002	-0.6448	St-Aphast	Ar-Tutabs	0.5744	1.2043	-0.6299
Ne-Caenig	Ar-Ladful	0.4719	1.1002	-0.6283	Fu-Morsp.	Ar-Calmac	0.6691	1.2045	-0.5354

Ar-Aptruf	Ar-Trivap	0.4061	1.1009	-0.6948	St-Aphast	Ar-Epacla	0.6158	1.2056	-0.5899
Ar-Onttau	Ar-Sogfur	0.3276	1.1010	-0.7734	St-Aphast	Ec-Ophspi	0.7177	1.2065	-0.4887
Ar-Eupann	Ar-Osmbic	0.2371	1.1014	-0.8643	Ne-Caeele	Ar-Anogla	0.5815	1.2078	-0.6263
St-Pytoli	Ar-Pseelo	0.5321	1.1020	-0.5699	Ne-Caenig	Ar-Anogla	0.5769	1.2078	-0.6309
St-Pilapi	Ar-Lepdec	0.4548	1.1021	-0.6473	Ar-Calmac	Ar-Thrpal	0.4161	1.2080	-0.7920
Ne-Caeele	Ar-Amytra	0.4102	1.1022	-0.6921	Fu-Morsp.	Ar-Sipfla	0.6546	1.2081	-0.5535
Ne-Caenig	Ar-Amytra	0.4100	1.1022	-0.6922	Ar-Aptruf	Ar-Bomman	0.5280	1.2084	-0.6805
St-Pytoli	Ar-Schgra	0.4746	1.1025	-0.6279	St-Phycam	Mo-Halrub	0.5866	1.2085	-0.6219
St-Pytoli	Mo-Halrub	0.5351	1.1028	-0.5677	St-Aphast	Mo-Batpla	0.7543	1.2093	-0.4550
Fn-Morsp.	Ar-Manjur	0.4366	1.1035	-0.6669	Fu-Lobtra	Mo-Limfor	0.6838	1.2107	-0.5269
St-Aphste	Ar-Osmbic	0.5190	1.1046	-0.5856	Fu-Morsp.	Ar-Timgen	0.5811	1.2111	-0.6300
Ar-Thrpal	Ar-Vantam	0.2836	1.1048	-0.8212	Fu-Morsp.	Ar-Acypis	0.6694	1.2112	-0.5418
St-Pilapi	Ar-Trivap	0.5999	1.1050	-0.5050	Fu-Morsp.	Ar-Censcu	0.6928	1.2114	-0.5186
St-Pilapi	Ar-Acypis	0.4628	1.1063	-0.6435	Fu-Morsp.	Ar-Timshe	0.5731	1.2121	-0.6390
Ne-Caeele	Ar-Pseelo	0.4287	1.1072	-0.6785	Fu-Lobtra	Ar-Lymdis	0.6032	1.2123	-0.6091
Ne-Caenig	Ar-Pseelo	0.4325	1.1072	-0.6747	Fu-Morsp.	Mo-Batpla	0.7091	1.2128	-0.5036
Fn-Morsp.	Ar-Schgra	0.4574	1.1075	-0.6501	Ar-Aptruf	Ar-Onttau	0.5013	1.2134	-0.7121
St-Phytub	Mo-Halrub	0.5588	1.1078	-0.5490	Ne-Caeele	Ar-Trivap	0.6465	1.2134	-0.5669
St-Aphste	Ar-Limcal	0.4663	1.1078	-0.6414	Ne-Caenig	Ar-Trivap	0.6545	1.2134	-0.5589
Ar-Ampamp	Ar-Pseelo	0.4257	1.1080	-0.6822	St-Aphste	St-Pytoli	0.2640	1.2134	-0.9494
Ar-Aptruf	Ar-Manjur	0.2770	1.1090	-0.8320	St-Aphinv	Mo-Mermer	0.6839	1.2135	-0.5296
Ar-Calmac	Ar-Ladful	0.3244	1.1090	-0.7846	St-Phycam	Mo-Batpla	0.8031	1.2135	-0.4104
Ar-Danmel	Ar-Osmbic	0.3126	1.1090	-0.7964	Fu-Morsp.	Ar-Sogfur	0.5915	1.2137	-0.6222
Ar-Lepdec	Ar-Thrpal	0.3101	1.1097	-0.7996	St-Phycam	Ar-Locmig	0.7008	1.2137	-0.5130
Ar-Temlon	Ar-Thrpal	0.3256	1.1099	-0.7843	St-Physyr	Fu-Lobtra	0.4923	1.2152	-0.7229
St-Pytoli	Ar-Ampamp	0.3875	1.1107	-0.7231	Fu-Morsp.	Ar-Galmel	0.6090	1.2154	-0.6064
Ar-Ampamp	Ar-Glocon	0.3431	1.1109	-0.7679	St-Phycam	Ar-Aptruf	0.6691	1.2160	-0.5469
Fn-Morsp.	Mo-Batpla	0.4854	1.1111	-0.6257	St-Physyr	Ar-Anogla	0.6606	1.2165	-0.5559
Fn-Morsp.	Ar-Amytra	0.4281	1.1111	-0.6830	St-Aphste	Ar-Ladful	0.7303	1.2166	-0.4864
Ar-Glocon	Ar-Onttau	0.3517	1.1115	-0.7598	Fu-Morsp.	Mo-Limfor	0.6718	1.2177	-0.5460
Fn-Morsp.	Ar-Lymdis	0.4348	1.1115	-0.6767	Ne-Caeele	Ar-Lephet	0.5407	1.2179	-0.6772
Ar-Eupuro	Ar-Trivap	0.4124	1.1123	-0.6999	Ne-Caenig	Ar-Lephet	0.5450	1.2179	-0.6730
St-Pytoli	Ar-Eufmex	0.5738	1.1138	-0.5399	Ne-Caeele	Ar-Erilan	0.6518	1.2181	-0.5663
Ar-Lephet	Ar-Thrpal	0.2977	1.1143	-0.8166	Ne-Caenig	Ar-Erilan	0.6854	1.2181	-0.5327
An-Hydele	Ar-Ampamp	0.3063	1.1145	-0.8082	St-Phytub	Ar-Censcu	0.6845	1.2191	-0.5346
Ar-Ampamp	Ar-Manjur	0.4185	1.1160	-0.6975	St-Aphste	Mo-Batpla	0.7358	1.2196	-0.4838
Fn-Morsp.	Ar-Neopin	0.4263	1.1183	-0.6920	St-Pilapi	Ar-Glocon	0.6421	1.2196	-0.5775
Ne-Caeele	Ar-Eufmex	0.4665	1.1185	-0.6520	Fu-Morsp.	Ar-Manjur	0.6137	1.2199	-0.6063
Ne-Caenig	Ar-Eufmex	0.4910	1.1185	-0.6275	St-Pytoli	Ar-Lepdec	0.6818	1.2204	-0.5386
Ar-Ampamp	Ar-Temlon	0.3636	1.1189	-0.7553	St-Aphast	Ar-Pluxyl	0.5851	1.2207	-0.6356
St-Pilapi	Ar-Censcu	0.5903	1.1194	-0.5290	St-Aphste	Ar-Glocon	0.6122	1.2219	-0.6097
Ar-Ampamp	Ar-Galmel	0.4310	1.1215	-0.6906	Ne-Caeele	Ar-Lepdec	0.5966	1.2230	-0.6263
Ar-Aptruf	Ar-Danmel	0.2657	1.1218	-0.8562	Ne-Caenig	Ar-Lepdec	0.5888	1.2230	-0.6341
St-Aphste	Ec-Ophspi	0.4522	1.1229	-0.6707	Fu-Morsp.	Ar-Schgra	0.6573	1.2230	-0.5657
Fn-Morsp.	Ec-Ophspi	0.3768	1.1229	-0.7462	Fu-Morsp.	Ar-Amytra	0.6524	1.2240	-0.5716
St-Pilapi	Ar-Sogfur	0.4337	1.1248	-0.6911	St-Pytoli	Ar-Limcal	0.7218	1.2251	-0.5033
Fn-Lobtra	Ar-Anogla	0.4498	1.1251	-0.6752	Ne-Caeele	Ar-Osmbic	0.5913	1.2253	-0.6340
Ne-Caeele	Ar-Censcu	0.4643	1.1252	-0.6609	Ne-Caenig	Ar-Osmbic	0.5893	1.2253	-0.6361
Ne-Caenig	Ar-Censcu	0.4923	1.1252	-0.6329	St-Aphinv	Ar-Bomman	0.6798	1.2258	-0.5460
Fn-Morsp.	Ar-Opebru	0.3888	1.1256	-0.7368	St-Pilapi	Ar-Adohon	0.6036	1.2264	-0.6228
Ar-Ampamp	Ar-Eupann	0.4022	1.1262	-0.7240	Ne-Caeele	Ar-Nillug	0.5869	1.2271	-0.6402
Fn-Morsp.	Ar-Lephet	0.4617	1.1262	-0.6645	Ne-Caenig	Ar-Nillug	0.5675	1.2271	-0.6596
St-Aphste	Ar-Manjur	0.4820	1.1266	-0.6446	St-Aphast	Ar-Anogla	0.7099	1.2275	-0.5176
St-Aphste	Ar-Sogfur	0.4465	1.1271	-0.6806	St-Aphste	Ar-Bomman	0.6385	1.2283	-0.5897
Ar-Ampamp	Ar-Calmac	0.4259	1.1288	-0.7030	Ne-Caeele	Ar-Homvit	0.5751	1.2290	-0.6540
Fn-Morsp.	Ar-Tutabs	0.3516	1.1290	-0.7774	Ne-Caenig	Ar-Homvit	0.5763	1.2290	-0.6527
Ar-Stemim	Ar-Thrpal	0.3511	1.1294	-0.7783	Ne-Caeele	Ar-Partep	0.6331	1.2298	-0.5967
Ar-Aptruf	Ar-Glocon	0.2619	1.1296	-0.8677	Ne-Caenig	Ar-Partep	0.6667	1.2298	-0.5631
St-Phycam	Ar-Ampamp	0.4243	1.1302	-0.7059	St-Aphinv	Ar-Lymdis	0.6173	1.2305	-0.6132

Ar-Aptruf	Ar-Opebru	0.2924	1.1304	-0.8380	Ne-Caeele	Ar-Acypis	0.6576	1.2316	-0.5740
St-Pilapi	An-Hydele	0.4146	1.1316	-0.7170	Ne-Caenig	Ar-Acypis	0.6667	1.2316	-0.5649
St-Pytoli	Ar-Amytra	0.4784	1.1321	-0.6537	St-Aphast	Ar-Locmig	0.7037	1.2318	-0.5281
An-Hydele	Ar-Thrpal	0.3433	1.1325	-0.7892	Fu-Lobtra	Mo-Batpla	0.6635	1.2320	-0.5685
St-Pilapi	Ar-Erilan	0.4905	1.1328	-0.6422	Fu-Morsp.	Ar-Bomman	0.6141	1.2326	-0.6185
St-Pilapi	Ar-Eupann	0.4480	1.1342	-0.6861	Ar-Bomman	Ar-Thrpal	0.4645	1.2328	-0.7683
Ne-Caeele	Ar-Opebru	0.3771	1.1358	-0.7587	Ne-Caeele	Ar-Sipfla	0.6422	1.2337	-0.5915
Ne-Caenig	Ar-Opebru	0.3765	1.1358	-0.7593	Ne-Caenig	Ar-Sipfla	0.6595	1.2337	-0.5742
Fn-Morsp.	Ar-Limcal	0.4540	1.1358	-0.6818	Fu-Morsp.	Ar-Lepdec	0.6434	1.2337	-0.5903
Ne-Caeele	Ar-Galmel	0.4241	1.1359	-0.7119	St-Pilapi	Ar-Aptruf	0.6842	1.2338	-0.5496
Ne-Caenig	Ar-Galmel	0.4203	1.1359	-0.7157	St-Aphste	Ar-Lymdis	0.6057	1.2353	-0.6296
St-Pilapi	Ar-Onttau	0.5145	1.1361	-0.6217	Ne-Caeele	Ar-Calmac	0.6353	1.2378	-0.6025
Ar-Aptruf	Ar-Neopin	0.2920	1.1373	-0.8453	Ne-Caenig	Ar-Calmac	0.6332	1.2378	-0.6047
Ar-Aptruf	Ar-Epacla	0.2923	1.1373	-0.8451	St-Pytoli	Ar-Timgen	0.5980	1.2381	-0.6401
St-Aphast	Ar-Phesol	0.5577	1.1387	-0.5810	Ar-Aptruf	Ar-Ladful	0.4161	1.2384	-0.8223
Fn-Morsp.	Ar-Eupann	0.4256	1.1399	-0.7143	St-Aphste	Ar-Neopin	0.6567	1.2386	-0.5819
St-Pilapi	Ar-Temlon	0.5003	1.1403	-0.6400	St-Pilapi	Ar-Thrpal	0.6290	1.2400	-0.6110
St-Pilapi	Ar-Partep	0.5621	1.1421	-0.5800	St-Aphinv	Ar-Adohon	0.6093	1.2414	-0.6321
St-Aphinv	Ar-Anogla	0.5075	1.1434	-0.6359	St-Phytub	Fu-Morsp.	0.4924	1.2431	-0.7507
Ne-Caeele	Ar-Neopin	0.3942	1.1443	-0.7501	St-Physyr	Ar-Lymdis	0.6321	1.2450	-0.6129
Ne-Caenig	Ar-Neopin	0.4066	1.1443	-0.7378	St-Physyr	Ar-Locmig	0.7159	1.2454	-0.5295
St-Physyr	Ar-Pluxyl	0.4443	1.1452	-0.7009	St-Phycam	Ar-Nillug	0.6392	1.2456	-0.6064
Ar-Aptruf	Ar-Sogfur	0.2715	1.1454	-0.8738	Ne-Caeele	Ar-Temlon	0.5973	1.2458	-0.6484
Ne-Caeele	Ar-Sitmis	0.4321	1.1465	-0.7144	Ne-Caenig	Ar-Temlon	0.5871	1.2458	-0.6587
Ne-Caenig	Ar-Sitmis	0.4397	1.1465	-0.7068	St-Pilapi	Ar-Lymdis	0.6554	1.2475	-0.5921
Ne-Caeele	Ar-Lephet	0.4023	1.1466	-0.7443	St-Aphast	Ar-Eufmex	0.6981	1.2479	-0.5498
Ne-Caenig	Ar-Lephet	0.4046	1.1466	-0.7420	St-Pytoli	Ne-Caeele	0.6226	1.2481	-0.6255
Ne-Caeele	Ar-Erilan	0.4658	1.1469	-0.6811	St-Pytoli	Ne-Caenig	0.6180	1.2481	-0.6301
Ne-Caenig	Ar-Erilan	0.4799	1.1469	-0.6670	Fu-Morsp.	Ar-Helarm	0.6258	1.2485	-0.6227
St-Pilapi	Ar-Epacla	0.4513	1.1471	-0.6958	Ne-Caeele	Ar-Stemim	0.6041	1.2488	-0.6447
Ar-Aptruf	Ar-Schgra	0.3185	1.1474	-0.8289	Ne-Caenig	Ar-Stemim	0.6535	1.2488	-0.5953
St-Phytub	Ar-Phesol	0.5747	1.1475	-0.5727	Fu-Lobtra	Ar-Pluxyl	0.6198	1.2504	-0.6306
St-Physyr	Mo-Halrub	0.5481	1.1482	-0.6001	Fu-Morsp.	Ar-Aulsol	0.5863	1.2509	-0.6646
St-Pilapi	Ar-Stedum	0.5638	1.1483	-0.5845	St-Pytoli	Ar-Stemim	0.6678	1.2509	-0.5832
St-Pytoli	Ar-Thrpal	0.4371	1.1500	-0.7129	St-Aphinv	Ar-Ampamp	0.6493	1.2523	-0.6030
St-Aphinv	Ec-Ophspi	0.4460	1.1504	-0.7044	St-Aphast	Ar-Lymdis	0.6257	1.2529	-0.6272
Ne-Caeele	Ar-Stedum	0.4714	1.1506	-0.6792	St-Pytoli	Ar-Glocon	0.6316	1.2532	-0.6216
Ne-Caenig	Ar-Stedum	0.4763	1.1506	-0.6743	Ne-Caeele	Ar-Helarm	0.6529	1.2540	-0.6011
St-Pilapi	Ar-Osmbic	0.5075	1.1506	-0.6431	Ne-Caenig	Ar-Helarm	0.6516	1.2540	-0.6024
St-Pilapi	Ar-Eupuro	0.4538	1.1510	-0.6972	St-Aphinv	Ar-Osmbic	0.6820	1.2542	-0.5722
Fn-Morsp.	Mo-Mermer	0.4795	1.1519	-0.6725	St-Pytoli	Ar-Erilan	0.7354	1.2542	-0.5189
St-Pytoli	Ar-Sogfur	0.4371	1.1530	-0.7159	St-Phytub	Ar-Bomman	0.6742	1.2545	-0.5803
St-Aphast	Fn-Lobtra	0.3648	1.1535	-0.7887	Fu-Lobtra	Ar-Locmig	0.6572	1.2556	-0.5984
St-Aphste	Mo-Limfor	0.5843	1.1535	-0.5692	Fu-Morsp.	Ar-Nillug	0.6117	1.2556	-0.6440
St-Pytoli	Ar-Opebru	0.4558	1.1547	-0.6989	St-Pytoli	Ar-Adohon	0.5812	1.2558	-0.6746
Ne-Caeele	Ar-Temlon	0.3849	1.1547	-0.7698	St-Phycam	Mo-Mermer	0.7245	1.2560	-0.5316
Ne-Caenig	Ar-Temlon	0.3750	1.1547	-0.7797	St-Aphste	Ar-Lephet	0.6901	1.2563	-0.5662
Ar-Amytra	Ar-Aptruf	0.2775	1.1549	-0.8775	Fu-Morsp.	Mo-Mermer	0.6341	1.2570	-0.6229
St-Pytoli	Ar-Trivap	0.5760	1.1550	-0.5790	St-Pytoli	Ar-Aptruf	0.6551	1.2574	-0.6023
St-Aphast	Mo-Batpla	0.5872	1.1552	-0.5680	St-Phytub	Ar-Adohon	0.5936	1.2578	-0.6641
St-Pilapi	Ar-Opebru	0.4687	1.1554	-0.6867	St-Phycam	Ar-Thrpal	0.5947	1.2578	-0.6631
Ne-Caeele	Ar-Phesol	0.4812	1.1554	-0.6743	St-Aphinv	Ar-Ladful	0.7522	1.2578	-0.5056
Ne-Caenig	Ar-Phesol	0.5146	1.1554	-0.6409	St-Aphste	Ar-Adohon	0.5630	1.2583	-0.6953
St-Aphste	Ar-Locmig	0.5374	1.1555	-0.6181	St-Aphinv	Ar-Epacla	0.6222	1.2585	-0.6363
Ar-Aptruf	Ar-Bomman	0.3081	1.1558	-0.8477	St-Phycam	Ar-Pluxyl	0.5390	1.2587	-0.7197
St-Aphast	Ar-Pluxyl	0.4426	1.1563	-0.7137	St-Pytoli	Ar-Partep	0.6909	1.2589	-0.5681
St-Pilapi	Ar-Bomman	0.5140	1.1564	-0.6424	St-Phycam	Ar-Anogla	0.6632	1.2592	-0.5961
Ar-Aptruf	Ar-Lymdis	0.2702	1.1567	-0.8865	Ne-Caeele	Ar-Glocon	0.5797	1.2595	-0.6798
St-Aphinv	Mo-Batpla	0.5760	1.1568	-0.5807	Ne-Caenig	Ar-Glocon	0.5855	1.2595	-0.6739

St-Pytoli	Ar-Helzea	0.4584	1.1568	-0.6984	Fu-Morsp.	Ar-Locmig	0.6593	1.2614	-0.6021
Ne-Caeele	Ar-Danmel	0.3785	1.1571	-0.7786	Fu-Lobtra	Mo-Mermer	0.6106	1.2618	-0.6512
Ne-Caenig	Ar-Danmel	0.3963	1.1571	-0.7608	St-Aphast	Ar-Amytra	0.6329	1.2621	-0.6292
St-Phycam	Mo-Limfor	0.5905	1.1574	-0.5668	St-Aphast	Ar-Adohon	0.6064	1.2632	-0.6568
Ne-Caeele	Ar-Helzea	0.3728	1.1582	-0.7854	St-Aphste	Ar-Amytra	0.5955	1.2634	-0.6680
Ne-Caenig	Ar-Helzea	0.3691	1.1582	-0.7891	Ne-Caeele	Ar-Ampamp	0.5885	1.2637	-0.6752
St-Pytoli	Ar-Ladful	0.5166	1.1585	-0.6418	Ne-Caenig	Ar-Ampamp	0.5839	1.2637	-0.6798
St-Aphinv	Ar-Phesol	0.5523	1.1587	-0.6064	Fu-Morsp.	Ar-Temlon	0.6406	1.2640	-0.6234
Ar-Aptruf	Ar-Homvit	0.2590	1.1588	-0.8998	St-Phytub	Ar-Lymdis	0.6364	1.2641	-0.6277
St-Aphste	Ar-Trivap	0.5921	1.1591	-0.5671	Ne-Caeele	Ar-Stedum	0.6648	1.2648	-0.6000
St-Pilapi	Ar-Ampamp	0.4331	1.1594	-0.7263	Ne-Caenig	Ar-Stedum	0.6798	1.2648	-0.5851
Ar-Aptruf	Ar-Sipfla	0.3279	1.1594	-0.8315	Ne-Caeele	Ar-Adohon	0.5744	1.2666	-0.6922
St-Pilapi	Ar-Amytra	0.4731	1.1600	-0.6868	Ne-Caenig	Ar-Adohon	0.5698	1.2666	-0.6968
St-Aphste	Ar-Aptruf	0.5055	1.1601	-0.6547	St-Aphinv	Ar-Stedum	0.7318	1.2669	-0.5351
Ar-Aptruf	Ar-Partep	0.3396	1.1602	-0.8206	Ne-Caeele	Ar-Limcal	0.6211	1.2670	-0.6459
St-Physyr	Ec-Ophspi	0.4569	1.1603	-0.7034	Ne-Caenig	Ar-Limcal	0.6204	1.2670	-0.6466
Fn-Lobtra	Ar-Opebru	0.4181	1.1605	-0.7424	St-Phytub	Mo-Mermer	0.6636	1.2674	-0.6038
St-Physyr	Mo-Limfor	0.5848	1.1606	-0.5758	Fu-Lobtra	Ar-Epacla	0.6078	1.2692	-0.6614
St-Aphast	Ar-Opebru	0.4672	1.1607	-0.6934	St-Phycam	Ar-Epacla	0.5634	1.2694	-0.7061
Ne-Caeele	Ar-Sogfur	0.3788	1.1614	-0.7826	St-Phytub	Ar-Ladful	0.7105	1.2696	-0.5591
Ne-Caenig	Ar-Sogfur	0.3910	1.1614	-0.7704	St-Phycam	Fu-Lobtra	0.5265	1.2703	-0.7438
Fn-Morsp.	Ar-Temlon	0.4352	1.1619	-0.7267	St-Physyr	Ar-Erilan	0.7648	1.2706	-0.5058
Fn-Morsp.	Ar-Pseelo	0.4759	1.1624	-0.6865	Ne-Caeele	Ar-Manjur	0.6305	1.2711	-0.6407
Ne-Caeele	Ar-Onttau	0.4489	1.1629	-0.7141	Ne-Caenig	Ar-Manjur	0.6247	1.2711	-0.6464
Ne-Caenig	Ar-Onttau	0.4617	1.1629	-0.7013	St-Aphast	Ar-Stedum	0.7309	1.2715	-0.5406
Fn-Morsp.	Ar-Galmel	0.4460	1.1631	-0.7171	Ne-Caeele	Ar-Pluxyl	0.5761	1.2743	-0.6982
Ar-Aptruf	Ar-Tutabs	0.2763	1.1632	-0.8869	Ne-Caenig	Ar-Pluxyl	0.5615	1.2743	-0.7127
St-Pilapi	Ar-Ladful	0.5163	1.1635	-0.6471	Fu-Morsp.	Ar-Eupann	0.5871	1.2744	-0.6873
St-Pilapi	Ar-Schgra	0.4626	1.1642	-0.7016	St-Physyr	Ar-Ampamp	0.5482	1.2752	-0.7270
St-Pilapi	Ar-Homvit	0.4664	1.1643	-0.6979	St-Aphast	Ar-Trivap	0.7087	1.2761	-0.5674
St-Pilapi	Ar-Manjur	0.4851	1.1645	-0.6794	St-Aphast	St-Pytoli	0.2723	1.2762	-1.0039
Ar-Aptruf	Ar-Stedum	0.3325	1.1650	-0.8326	Ar-Aptruf	Ar-Eupann	0.3997	1.2762	-0.8765
St-Pytoli	Ar-Aptruf	0.4662	1.1653	-0.6990	St-Aphast	Ar-Osmbic	0.6920	1.2765	-0.5844
Ne-Caeele	Ar-Helarm	0.3698	1.1658	-0.7961	St-Phycam	Ar-Helarm	0.6435	1.2766	-0.6331
Ne-Caenig	Ar-Helarm	0.3674	1.1658	-0.7984	St-Aphste	Ar-Phesol	0.6526	1.2768	-0.6241
St-Pytoli	Ar-Temlon	0.4728	1.1663	-0.6935	St-Physyr	Ar-Tutabs	0.5634	1.2769	-0.7135
Fn-Morsp.	Ar-Eupuro	0.4253	1.1663	-0.7410	Fu-Morsp.	Ar-Eufmex	0.6781	1.2770	-0.5990
Ne-Caeele	Ar-Anogla	0.4652	1.1664	-0.7012	Ne-Caeele	Ar-Epacla	0.5929	1.2771	-0.6842
Ne-Caenig	Ar-Anogla	0.4762	1.1664	-0.6902	Ne-Caenig	Ar-Epacla	0.5815	1.2771	-0.6956
Fn-Lobtra	Ec-Ophspi	0.3916	1.1667	-0.7751	St-Phycam	Ar-Manjur	0.6094	1.2774	-0.6680
St-Pilapi	Ar-Stemim	0.5523	1.1668	-0.6145	Fu-Morsp.	Ar-Osmbic	0.7003	1.2775	-0.5772
St-Phytub	Mo-Limfor	0.5798	1.1669	-0.5870	St-Phytub	Ar-Helarm	0.6336	1.2778	-0.6442
St-Pytoli	Ar-Eupann	0.4553	1.1674	-0.7121	Fu-Lobtra	Ar-Phesol	0.6895	1.2783	-0.5889
St-Pilapi	Ar-Vantam	0.4808	1.1684	-0.6876	St-Phytub	Ar-Manjur	0.6276	1.2791	-0.6515
St-Phycam	Ec-Ophspi	0.4507	1.1686	-0.7179	St-Aphinv	Ar-Calmac	0.6904	1.2793	-0.5889
Fn-Morsp.	Ar-Helarm	0.3784	1.1686	-0.7902	Ne-Caeele	Ar-Timcri	0.5965	1.2796	-0.6831
St-Pytoli	Ar-Censcu	0.5818	1.1688	-0.5869	Ne-Caenig	Ar-Timcri	0.5765	1.2796	-0.7031
St-Pilapi	Ar-Calmac	0.4960	1.1693	-0.6734	St-Physyr	Ar-Osmbic	0.6726	1.2797	-0.6071
Ne-Caeele	Ar-Partep	0.4386	1.1694	-0.7308	Fu-Lobtra	Ar-Amytra	0.6514	1.2797	-0.6283
Ne-Caenig	Ar-Partep	0.4732	1.1694	-0.6962	St-Aphinv	Ar-Erilan	0.7023	1.2815	-0.5792
Mo-Batpla	Ar-Aptruf	0.4405	1.1696	-0.7291	Ne-Caeele	Ar-Timshe	0.5896	1.2816	-0.6919
St-Pytoli	Ar-Helarm	0.4538	1.1697	-0.7158	Ne-Caenig	Ar-Timshe	0.5739	1.2816	-0.7077
St-Phytub	Ar-Pseelo	0.5472	1.1709	-0.6237	St-Phytub	Ar-Neopin	0.5901	1.2818	-0.6917
Ar-Acypis	Ar-Aptruf	0.3066	1.1710	-0.8644	St-Physyr	Ar-Manjur	0.6097	1.2822	-0.6726
Fn-Morsp.	Ar-Eufmex	0.5602	1.1713	-0.6110	Ne-Caeele	Ar-Timpop	0.5921	1.2826	-0.6905
St-Pilapi	Ar-Helzea	0.4710	1.1714	-0.7005	Ne-Caenig	Ar-Timpop	0.5804	1.2826	-0.7023
Fn-Morsp.	Ar-Locmig	0.4816	1.1719	-0.6903	St-Phytub	Ar-Eufmex	0.6928	1.2827	-0.5899
Ne-Caeele	Ar-Lymdis	0.4156	1.1720	-0.7564	St-Aphste	St-Pilapi	0.2539	1.2829	-1.0290
Ne-Caenig	Ar-Lymdis	0.4148	1.1720	-0.7572	Fu-Morsp.	Ar-Aptruf	0.6172	1.2832	-0.6660

St-Aphste	Ar-Pseelo	0.5215	1.1723	-0.6508	St-Pytoli	Ar-Timshe	0.5961	1.2846	-0.6885
Fn-Morsp.	Ar-Sogfur	0.3995	1.1726	-0.7731	St-Pytoli	Ar-Timtah	0.5903	1.2846	-0.6943
Fn-Morsp.	Ar-Trivap	0.5328	1.1747	-0.6419	St-Aphinv	Ar-Timbar	0.6399	1.2852	-0.6453
Fn-Morsp.	Ar-Helzea	0.3776	1.1752	-0.7976	St-Aphinv	Ar-Timtah	0.6375	1.2852	-0.6476
St-Pytoli	Ar-Galmel	0.5202	1.1752	-0.6550	St-Aphast	Ar-Bomman	0.6854	1.2852	-0.5998
St-Pytoli	Ar-Lephet	0.4876	1.1761	-0.6885	St-Pytoli	Ar-Timcri	0.6044	1.2856	-0.6812
Fn-Morsp.	Ar-Timcri	0.4619	1.1778	-0.7159	St-Aphinv	St-Phytub	0.3233	1.2864	-0.9630
St-Aphinv	Ar-Amytra	0.4768	1.1785	-0.7016	St-Pytoli	Ar-Timpop	0.5977	1.2866	-0.6889
Ne-Caeele	Ar-Lepdec	0.4322	1.1785	-0.7463	Ne-Caeele	Ar-Timbar	0.6090	1.2867	-0.6777
Ne-Caenig	Ar-Lepdec	0.4717	1.1785	-0.7069	Ne-Caenig	Ar-Timbar	0.5970	1.2867	-0.6897
Ar-Eupuro	Ar-Thrpal	0.2794	1.1787	-0.8993	St-Pytoli	Ar-Stedum	0.7349	1.2868	-0.5519
St-Aphinv	Ar-Galmel	0.5159	1.1787	-0.6628	Ne-Caeele	Ar-Timgen	0.5971	1.2871	-0.6900
Fn-Lobtra	Ar-Lymdis	0.4154	1.1788	-0.7634	Ne-Caenig	Ar-Timgen	0.5771	1.2871	-0.7100
St-Pilapi	Ar-Helarm	0.4680	1.1792	-0.7113	Fu-Morsp.	Ar-Eupuro	0.5872	1.2872	-0.7000
St-Aphste	Ar-Thrpal	0.4536	1.1793	-0.7256	Ne-Caeele	Ar-Timmon	0.5966	1.2874	-0.6908
St-Aphinv	Ar-Chisup	0.4708	1.1793	-0.7085	Ne-Caenig	Ar-Timmon	0.5766	1.2874	-0.7107
St-Aphste	Ar-Bomman	0.4948	1.1806	-0.6858	St-Aphste	Ar-Erilan	0.7305	1.2880	-0.5575
St-Pilapi	Ar-Galmel	0.5043	1.1814	-0.6771	St-Pytoli	Fu-Morsp.	0.4559	1.2882	-0.8323
St-Phycam	Ar-Sitmis	0.5250	1.1821	-0.6571	Fu-Lobtra	Ar-Thrpal	0.5478	1.2888	-0.7410
St-Aphast	Mo-Halrub	0.5879	1.1822	-0.5943	St-Aphste	Ar-Timbar	0.6363	1.2888	-0.6525
St-Phytub	Ar-Pluxyl	0.4313	1.1834	-0.7521	St-Aphste	Ar-Timtah	0.6367	1.2888	-0.6521
Ar-Ladful	Ar-Thrpal	0.3340	1.1841	-0.8501	Ne-Caeele	Ar-Thrpal	0.5733	1.2891	-0.7158
Fn-Morsp.	Ar-Timpop	0.4620	1.1843	-0.7222	Ne-Caenig	Ar-Thrpal	0.5728	1.2891	-0.7164
Mo-Halrub	Ne-Caeele	0.4520	1.1849	-0.7329	St-Pytoli	Ar-Timbar	0.5900	1.2897	-0.6997
Mo-Halrub	Ne-Caenig	0.4274	1.1849	-0.7575	St-Pytoli	Ar-Timmon	0.6442	1.2897	-0.6455
St-Aphste	Ar-Homvit	0.4910	1.1849	-0.6939	St-Phytub	Ar-Stedum	0.7615	1.2910	-0.5295
Fn-Morsp.	Ar-Timgen	0.4794	1.1855	-0.7061	St-Physyr	Ar-Schgra	0.7746	1.2913	-0.5166
Fn-Morsp.	Ar-Nillug	0.3553	1.1857	-0.8305	St-Physyr	Ar-Epacla	0.5822	1.2919	-0.7097
St-Pytoli	Ar-Limcal	0.4696	1.1861	-0.7164	Ne-Caeele	Ar-Timtah	0.6071	1.2920	-0.6848
Fn-Morsp.	Ar-Censcu	0.5507	1.1877	-0.6370	Ne-Caenig	Ar-Timtah	0.5952	1.2920	-0.6968
Fn-Morsp.	Ar-Phesol	0.4987	1.1894	-0.6908	St-Aphste	Ar-Helarm	0.6700	1.2927	-0.6227
Ne-Caeele	Ar-Osmbic	0.4510	1.1894	-0.7384	St-Aphinv	Ar-Timmon	0.6863	1.2928	-0.6065
Ne-Caenig	Ar-Osmbic	0.4704	1.1894	-0.7190	Fu-Morsp.	Ar-Trivap	0.6508	1.2930	-0.6422
St-Pytoli	Ar-Eupuro	0.4560	1.1894	-0.7334	St-Aphste	Ar-Timcri	0.6392	1.2930	-0.6538
St-Aphste	Ar-Chisup	0.4803	1.1897	-0.7094	St-Aphste	Ar-Timmon	0.6412	1.2930	-0.6519
Ar-Ampamp	Ar-Eupuro	0.4059	1.1898	-0.7838	St-Aphinv	Ar-Timpop	0.6489	1.2939	-0.6450
St-Pytoli	Ar-Bomman	0.5087	1.1901	-0.6814	St-Aphast	Ar-Ampamp	0.6173	1.2943	-0.6770
St-Aphinv	Ar-Onttau	0.4891	1.1902	-0.7012	St-Aphast	Ar-Neopin	0.6634	1.2943	-0.6309
St-Pytoli	Ar-Onttau	0.5323	1.1908	-0.6585	St-Phycam	St-Pytoli	0.2426	1.2943	-1.0518
St-Pytoli	Ar-Stemim	0.5640	1.1931	-0.6290	Fu-Morsp.	Ar-Erilan	0.6629	1.2956	-0.6328
St-Aphste	Ar-Nillug	0.4294	1.1935	-0.7641	St-Aphast	Mo-Limfor	0.7525	1.2957	-0.5432
Fn-Lobtra	Mo-Batpla	0.4888	1.1942	-0.7054	St-Aphinv	Ar-Timcri	0.6492	1.2972	-0.6480
Ar-Eupann	Ar-Thrpal	0.2829	1.1943	-0.9113	St-Aphste	Ar-Timgen	0.6472	1.2976	-0.6505
St-Aphste	Ar-Temlon	0.4933	1.1945	-0.7012	St-Phytub	Ar-Temlon	0.6570	1.2982	-0.6412
St-Phycam	Ar-Locmig	0.5549	1.1950	-0.6402	St-Aphinv	Ar-Timshe	0.6422	1.2983	-0.6562
Ne-Caeele	Ar-Calmac	0.4597	1.1952	-0.7355	St-Phytub	Ar-Tutabs	0.5700	1.2990	-0.7290
Ne-Caenig	Ar-Calmac	0.4483	1.1952	-0.7469	St-Physyr	Ar-Aptruf	0.6973	1.3000	-0.6027
Ar-Bomman	Ar-Thrpal	0.3011	1.1953	-0.8942	St-Aphste	Ar-Timpop	0.6411	1.3001	-0.6590
Mo-Batpla	Ne-Caeele	0.4681	1.1956	-0.7275	St-Aphste	Ar-Timshe	0.6386	1.3001	-0.6616
Mo-Batpla	Ne-Caenig	0.4877	1.1956	-0.7078	St-Aphinv	Ar-Timgen	0.6466	1.3001	-0.6536
St-Physyr	Ar-Anogla	0.5696	1.1962	-0.6266	Ar-Aptruf	Ar-Eupuro	0.3991	1.3019	-0.9027
Ne-Caeele	Ar-Homvit	0.4126	1.1969	-0.7842	St-Aphinv	Ar-Lephet	0.7098	1.3024	-0.5925
Ne-Caenig	Ar-Homvit	0.3963	1.1969	-0.8006	Fu-Morsp.	Ar-Partep	0.6305	1.3026	-0.6722
Ar-Calmac	Ar-Thrpal	0.2938	1.1974	-0.9036	St-Aphste	Ar-Calmac	0.7156	1.3031	-0.5875
St-Aphast	Ar-Epacla	0.4788	1.1976	-0.7187	St-Aphast	Mo-Mermer	0.7129	1.3040	-0.5910
St-Pytoli	Ar-Calmac	0.5180	1.1981	-0.6801	St-Aphinv	Ar-Stemim	0.6455	1.3043	-0.6588
St-Aphste	Ar-Schgra	0.4692	1.1984	-0.7291	St-Aphast	St-Phytub	0.2832	1.3046	-1.0214
Fn-Lobtra	Ar-Locmig	0.4584	1.1993	-0.7409	St-Aphast	Ar-Onttau	0.7534	1.3053	-0.5518
St-Aphast	Ec-Ophspi	0.4488	1.2010	-0.7522	Fu-Morsp.	Ar-Glocon	0.5977	1.3057	-0.7080

St-Phytub	Ar-Sitmis	0.4985	1.2011	-0.7026	Fu-Morsp.	Ar-Phesol	0.7035	1.3058	-0.6023
St-Physyr	Mo-Batpla	0.5739	1.2012	-0.6273	St-Aphast	Ar-Galmel	0.6080	1.3060	-0.6980
Ne-Caeele	Ar-Ampamp	0.4016	1.2012	-0.7996	St-Aphinv	Ar-Aptruf	0.6992	1.3063	-0.6071
Ne-Caenig	Ar-Ampamp	0.3816	1.2012	-0.8197	St-Phycam	Ar-Limcal	0.7566	1.3067	-0.5501
Fn-Morsp.	Ar-Partep	0.5071	1.2017	-0.6946	St-Aphste	Mo-Mermer	0.6837	1.3073	-0.6235
St-Aphste	Ar-Opebru	0.4676	1.2020	-0.7344	St-Aphinv	Ar-Thrpal	0.6536	1.3084	-0.6548
St-Aphste	Ar-Danmel	0.4490	1.2021	-0.7532	St-Phytub	St-Pytoli	0.2187	1.3086	-1.0899
St-Pytoli	An-Hydele	0.3976	1.2024	-0.8048	St-Aphinv	Ar-Trivap	0.7146	1.3087	-0.5941
St-Aphinv	Mo-Mermer	0.5505	1.2025	-0.6519	St-Phycam	Ar-Sipfla	0.8090	1.3099	-0.5009
St-Aphste	Ar-Ladful	0.4983	1.2026	-0.7043	St-Physyr	Ar-Adohon	0.5726	1.3106	-0.7381
Ne-Caeele	Ar-Limcal	0.4231	1.2030	-0.7799	St-Phytub	Ar-Trivap	0.6674	1.3108	-0.6434
Ne-Caenig	Ar-Limcal	0.4419	1.2030	-0.7611	St-Aphast	Ar-Glocon	0.6649	1.3131	-0.6482
Ne-Caeele	Ar-Manjur	0.4098	1.2040	-0.7942	St-Aphast	Ar-Thrpal	0.6673	1.3132	-0.6459
Ne-Caenig	Ar-Manjur	0.4122	1.2040	-0.7918	St-Physyr	Mo-Mermer	0.6605	1.3146	-0.6541
St-Pytoli	Ar-Neopin	0.4618	1.2043	-0.7424	St-Aphast	Ar-Nillug	0.6860	1.3147	-0.6288
St-Phycam	Ar-Thrpal	0.4404	1.2044	-0.7640	St-Phytub	Ar-Homvit	0.6383	1.3154	-0.6772
Ne-Caeele	Ar-Trivap	0.5250	1.2048	-0.6798	St-Aphinv	Ar-Helarm	0.6760	1.3159	-0.6398
Ne-Caenig	Ar-Trivap	0.5401	1.2048	-0.6648	St-Phycam	Ar-Onttau	0.7741	1.3171	-0.5430
St-Phytub	Fn-Morsp.	0.3388	1.2049	-0.8661	St-Physyr	Ar-Helarm	0.6521	1.3173	-0.6652
St-Aphinv	Ar-Ladful	0.4944	1.2056	-0.7112	St-Physyr	Ar-Bomman	0.6534	1.3183	-0.6649
St-Aphinv	Ar-Bomman	0.4966	1.2057	-0.7091	St-Aphinv	Ar-Glocon	0.6668	1.3187	-0.6520
St-Aphste	Ar-Glocon	0.4596	1.2058	-0.7461	Fu-Lobtra	Ar-Manjur	0.6311	1.3195	-0.6884
Fn-Morsp.	Ar-Erilan	0.4925	1.2074	-0.7149	St-Phytub	Ar-Partep	0.6459	1.3199	-0.6739
St-Pilapi	Ar-Pluxyl	0.4223	1.2074	-0.7851	Fu-Lobtra	Ec-Ophspi	0.6433	1.3204	-0.6771
St-Phytub	Ar-Epacla	0.4561	1.2085	-0.7524	St-Physyr	Ar-Neopin	0.6162	1.3208	-0.7046
St-Aphinv	Ar-Sitmis	0.4773	1.2089	-0.7316	Fu-Lobtra	Ar-Homvit	0.6309	1.3227	-0.6918
St-Phytub	Ar-Amytra	0.4832	1.2091	-0.7259	St-Aphinv	Ar-Homvit	0.6551	1.3228	-0.6677
St-Aphste	Ar-Lymdis	0.4865	1.2095	-0.7230	Fu-Lobtra	Ar-Tutabs	0.5978	1.3234	-0.7257
St-Pytoli	Ar-Timgen	0.5209	1.2101	-0.6892	St-Phytub	Ar-Calmac	0.7148	1.3248	-0.6100
St-Phytub	Ar-Censcu	0.5878	1.2115	-0.6236	St-Phycam	Ar-Acypis	0.7860	1.3256	-0.5397
Ne-Caeele	Ar-Tutabs	0.3569	1.2116	-0.8546	St-Phytub	Ar-Galmel	0.6150	1.3270	-0.7120
Ne-Caenig	Ar-Tutabs	0.3664	1.2116	-0.8452	St-Physyr	Ar-Nillug	0.6560	1.3281	-0.6721
St-Aphinv	Ar-Stedum	0.5726	1.2116	-0.6390	St-Aphste	Ne-Caeele	0.6940	1.3288	-0.6348
St-Aphinv	Ar-Adohon	0.4564	1.2118	-0.7553	St-Aphste	Ne-Caenig	0.6966	1.3288	-0.6322
St-Aphste	Ar-Adohon	0.4436	1.2128	-0.7693	St-Phycam	Ar-Osmbic	0.7197	1.3289	-0.6092
Ar-Aptruf	Ar-Onttau	0.3263	1.2135	-0.8872	St-Phytub	Ar-Sogfur	0.6495	1.3292	-0.6798
Ne-Caeele	Ar-Nillug	0.3521	1.2138	-0.8617	Fu-Lobtra	Ar-Sipfla	0.6479	1.3306	-0.6827
Ne-Caenig	Ar-Nillug	0.3609	1.2138	-0.8530	Fu-Lobtra	Ar-Aulsol	0.5935	1.3314	-0.7379
Ne-Caeele	Ar-Stemim	0.4531	1.2140	-0.7609	Fu-Lobtra	Ar-Schgra	0.6702	1.3323	-0.6622
Ne-Caenig	Ar-Stemim	0.4697	1.2140	-0.7442	St-Aphinv	Ar-Sipfla	0.6954	1.3327	-0.6373
Ar-Aptruf	Ar-Ladful	0.3116	1.2142	-0.9026	St-Aphste	Ar-Acypis	0.6941	1.3339	-0.6398
Ne-Caeele	Ar-Adohon	0.3640	1.2145	-0.8505	St-Phycam	Ar-Adohon	0.5482	1.3344	-0.7861
Ne-Caenig	Ar-Adohon	0.3518	1.2145	-0.8627	Fu-Morsp.	Ar-Stemim	0.6732	1.3358	-0.6626
Fn-Morsp.	Ar-Glocon	0.3681	1.2150	-0.8469	St-Aphinv	Ar-Lepdec	0.6544	1.3362	-0.6818
Fn-Lobtra	Ar-Sitmis	0.4067	1.2156	-0.8089	Fu-Lobtra	Ar-Ladful	0.6306	1.3368	-0.7061
St-Aphste	Ar-Lephet	0.4836	1.2173	-0.7337	St-Physyr	Ar-Lephet	0.6520	1.3372	-0.6851
St-Physyr	Ar-Phesol	0.5825	1.2181	-0.6356	St-Physyr	Ar-Timgen	0.6119	1.3374	-0.7255
St-Phytub	Ar-Opebru	0.4696	1.2200	-0.7504	St-Physyr	Ar-Eufmex	0.6934	1.3374	-0.6441
Fn-Morsp.	Ar-Bomman	0.4267	1.2205	-0.7938	St-Aphinv	Ar-Nillug	0.6849	1.3376	-0.6527
Ne-Caeele	Ar-Pluxyl	0.3709	1.2206	-0.8498	St-Aphast	Ar-Schgra	0.7437	1.3381	-0.5944
Ne-Caenig	Ar-Pluxyl	0.3481	1.2206	-0.8725	St-Aphste	Ar-Stemim	0.6491	1.3385	-0.6894
St-Pilapi	Ar-Adohon	0.4405	1.2217	-0.7812	Fu-Morsp.	Ar-Onttau	0.6585	1.3398	-0.6813
St-Pytoli	Ar-Erilan	0.4990	1.2228	-0.7238	St-Phycam	Ar-Homvit	0.6590	1.3399	-0.6809
St-Phycam	Mo-Halrub	0.5937	1.2231	-0.6294	Fu-Lobtra	Ar-Neopin	0.5962	1.3400	-0.7438
St-Pytoli	Ar-Chisup	0.5081	1.2237	-0.7156	St-Phycam	Ar-Neopin	0.6299	1.3414	-0.7115
St-Pilapi	Ar-Danmel	0.4497	1.2258	-0.7762	St-Phytub	Ar-Lephet	0.6790	1.3437	-0.6647
St-Aphste	Ar-Timgen	0.5183	1.2261	-0.7077	St-Aphste	Ar-Partep	0.6382	1.3458	-0.7076
St-Aphinv	Ar-Erilan	0.4906	1.2268	-0.7361	St-Aphinv	Ar-Censcu	0.6757	1.3465	-0.6708
St-Aphste	St-Phytub	0.2488	1.2271	-0.9783	St-Aphast	Ar-Ladful	0.7551	1.3472	-0.5920

St-Phytub	Ar-Danmel	0.4522	1.2273	-0.7750	St-Aphste	Ar-Sipfla	0.7121	1.3473	-0.6352
Fn-Morsp.	Mo-Limfor	0.4817	1.2279	-0.7462	St-Phycam	St-Pilapi	0.2492	1.3476	-1.0984
Fn-Lobtra	Ar-Pluxyl	0.3812	1.2281	-0.8469	St-Aphast	Ar-Aptruf	0.7163	1.3486	-0.6324
St-Aphste	Ar-Amytra	0.4642	1.2286	-0.7644	St-Phytub	Ar-Limcal	0.7476	1.3489	-0.6013
St-Phycam	Mo-Batpla	0.5693	1.2290	-0.6597	Fu-Lobtra	Ar-Galmel	0.6181	1.3493	-0.7311
St-Aphast	Ar-Eufmex	0.5494	1.2296	-0.6802	St-Phycam	Ar-Phesol	0.7316	1.3493	-0.6176
St-Aphast	Ar-Tutabs	0.4486	1.2297	-0.7811	St-Aphinv	Ar-Schgra	0.7197	1.3493	-0.6296
St-Pilapi	Ar-Glocon	0.4451	1.2302	-0.7850	Fu-Lobtra	Ar-Bomman	0.6353	1.3506	-0.7153
Ar-Aptruf	Ar-Vantam	0.2788	1.2302	-0.9513	St-Aphinv	Ar-Neopin	0.6306	1.3508	-0.7202
St-Aphste	Mo-Batpla	0.5764	1.2303	-0.6539	St-Aphast	Ar-Lephet	0.7064	1.3523	-0.6459
St-Physyr	Ar-Erilan	0.5595	1.2312	-0.6717	St-Aphinv	Ar-Partep	0.6264	1.3546	-0.7282
Ne-Caeele	Ar-Eupann	0.4146	1.2329	-0.8183	St-Aphast	Ar-Erilan	0.7325	1.3547	-0.6222
Ne-Caenig	Ar-Eupann	0.4286	1.2329	-0.8043	St-Phycam	Ar-Stemim	0.6852	1.3548	-0.6696
Fn-Lobtra	Mo-Mermer	0.4779	1.2334	-0.7555	St-Aphste	Ar-Anogla	0.6472	1.3552	-0.7080
St-Aphinv	Ar-Calmac	0.5188	1.2336	-0.7149	St-Phytub	Ar-Nillug	0.6409	1.3552	-0.7144
St-Pytoli	Ar-Partep	0.5573	1.2341	-0.6768	Fu-Lobtra	Ar-Lepdec	0.6329	1.3554	-0.7225
St-Aphste	St-Pytoli	0.2052	1.2347	-1.0295	St-Aphste	Ar-Lepdec	0.6323	1.3555	-0.7232
St-Pytoli	Ne-Caeele	0.4392	1.2348	-0.7957	St-Phytub	Mo-Batpla	0.7509	1.3561	-0.6053
St-Pytoli	Ne-Caenig	0.4255	1.2348	-0.8093	Fu-Lobtra	Ar-Trivap	0.6220	1.3569	-0.7350
St-Aphast	Ar-Sitmis	0.4790	1.2349	-0.7559	Fu-Lobtra	Ar-Ampamp	0.6192	1.3571	-0.7379
St-Aphste	Ar-Neopin	0.4832	1.2351	-0.7519	St-Aphast	Ar-Helarm	0.6761	1.3576	-0.6815
St-Aphast	Ar-Locmig	0.5640	1.2356	-0.6715	St-Phycam	Ar-Eufmex	0.7350	1.3576	-0.6226
Fn-Morsp.	Ar-Onttau	0.4898	1.2361	-0.7463	St-Aphast	Ar-Stemim	0.6631	1.3587	-0.6956
St-Aphinv	Ar-Lymdis	0.5071	1.2365	-0.7294	St-Phytub	Ar-Erilan	0.7511	1.3598	-0.6087
Ar-Aptruf	Ar-Eupann	0.2758	1.2367	-0.9609	St-Phycam	Ar-Glocon	0.5835	1.3598	-0.7763
St-Phycam	Ar-Nillug	0.4029	1.2384	-0.8355	St-Aphinv	Ar-Aulsol	0.6304	1.3598	-0.7294
St-Aphast	Ar-Anogla	0.5155	1.2385	-0.7230	Ne-Caeele	Ar-Eupuro	0.5385	1.3599	-0.8214
St-Aphinv	Ar-Ampamp	0.4578	1.2385	-0.7807	Ne-Caenig	Ar-Eupuro	0.5227	1.3599	-0.8373
St-Aphinv	Ar-Tutabs	0.4614	1.2388	-0.7774	St-Aphinv	Ar-Sogfur	0.6546	1.3601	-0.7055
St-Phycam	Mo-Mermer	0.5483	1.2388	-0.6905	St-Aphast	Ar-Calmac	0.7150	1.3603	-0.6453
St-Physyr	Ar-Locmig	0.5747	1.2405	-0.6657	St-Aphast	Ar-Timgen	0.6553	1.3604	-0.7051
Fn-Lobtra	Ar-Phesol	0.4847	1.2419	-0.7571	Ne-Caeele	Ar-Eupann	0.5430	1.3624	-0.8194
Ne-Caeele	Ar-Eupuro	0.4148	1.2419	-0.8271	Ne-Caenig	Ar-Eupann	0.5304	1.3624	-0.8319
Ne-Caenig	Ar-Eupuro	0.4265	1.2419	-0.8154	St-Phytub	Ar-Eupuro	0.5994	1.3626	-0.7632
St-Physyr	Ar-Ampamp	0.3976	1.2425	-0.8449	St-Aphste	Ar-Galmel	0.5958	1.3628	-0.7670
St-Physyr	Fn-Lobtra	0.3743	1.2438	-0.8696	Fu-Lobtra	Ar-Helarm	0.6510	1.3635	-0.7125
Fn-Lobtra	Ar-Amytra	0.4200	1.2446	-0.8245	St-Phytub	St-Pilapi	0.2262	1.3642	-1.1380
Ar-Aptruf	Ar-Eupuro	0.2721	1.2450	-0.9729	St-Aphast	Ar-Timbar	0.6487	1.3643	-0.7156
St-Aphste	Ar-Vantam	0.4844	1.2456	-0.7612	St-Aphast	Ar-Timtah	0.6491	1.3643	-0.7151
St-Pilapi	Ar-Thrpal	0.4399	1.2459	-0.8060	St-Aphste	Ar-Onttau	0.6971	1.3647	-0.6676
St-Aphast	Ar-Chisup	0.4860	1.2461	-0.7601	St-Phycam	Ar-Temlon	0.6604	1.3655	-0.7050
St-Pytoli	Ar-Lepdec	0.4820	1.2466	-0.7646	Fu-Morsp.	Ar-Homvit	0.6284	1.3668	-0.7384
St-Aphinv	Ar-Timgen	0.5258	1.2468	-0.7210	St-Phycam	Ar-Bomman	0.6742	1.3669	-0.6926
St-Phytub	Ar-Locmig	0.5507	1.2468	-0.6962	St-Physyr	Ar-Temlon	0.6369	1.3691	-0.7323
Fn-Morsp.	Ar-Lepdec	0.4461	1.2475	-0.8014	St-Aphinv	Ar-Temlon	0.6698	1.3702	-0.7004
St-Phycam	Fn-Lobtra	0.3657	1.2481	-0.8824	St-Physyr	Ar-Timbar	0.6093	1.3713	-0.7620
St-Aphste	Ar-Timcri	0.5158	1.2482	-0.7324	St-Physyr	Ar-Timtah	0.6074	1.3713	-0.7639
St-Aphast	Ar-Trivap	0.5981	1.2496	-0.6515	St-Physyr	Ar-Lepdec	0.6695	1.3717	-0.7021
Fn-Lobtra	Ar-Galmel	0.4284	1.2499	-0.8215	St-Phytub	Ar-Stemim	0.6508	1.3718	-0.7211
St-Phycam	Ar-Pluxyl	0.4383	1.2502	-0.8119	St-Aphste	Ar-Epacla	0.5904	1.3724	-0.7819
St-Pytoli	Ar-Vantam	0.5105	1.2504	-0.7398	St-Aphinv	Ar-Manjur	0.6384	1.3724	-0.7339
Fn-Lobtra	Ar-Chisup	0.4276	1.2516	-0.8240	St-Aphinv	Ar-Limcal	0.7146	1.3728	-0.6582
St-Pytoli	Ar-Timcri	0.5218	1.2524	-0.7306	St-Physyr	Ar-Calmac	0.6941	1.3734	-0.6793
St-Pilapi	Ar-Lymdis	0.4827	1.2533	-0.7706	St-Phytub	Ar-Ampamp	0.5827	1.3735	-0.7908
St-Pytoli	Ar-Timpop	0.5367	1.2533	-0.7167	Fu-Lobtra	Ar-Acypis	0.6591	1.3737	-0.7146
St-Aphste	An-Hydele	0.4236	1.2547	-0.8310	St-Pilapi	Ne-Caeele	0.6815	1.3749	-0.6934
St-Aphste	Ar-Timpop	0.5233	1.2547	-0.7315	St-Pilapi	Ne-Caenig	0.6820	1.3749	-0.6929
Ne-Caeele	Ar-Timgen	0.4228	1.2559	-0.8331	St-Physyr	Ar-Ladful	0.7007	1.3750	-0.6743
Ne-Caenig	Ar-Timgen	0.4328	1.2559	-0.8230	St-Phycam	Ar-Trivap	0.7045	1.3752	-0.6708

St-Aphinv	Ar-Epacla	0.4742	1.2570	-0.7828	Fu-Lobtra	Ar-Partep	0.6304	1.3755	-0.7451
St-Aphast	Ar-Adohon	0.4474	1.2573	-0.8099	St-Phycam	Ar-Timgen	0.5895	1.3763	-0.7868
St-Aphinv	Ar-Stemim	0.5428	1.2590	-0.7162	St-Physyr	Ar-Aulsol	0.6540	1.3765	-0.7225
St-Aphste	Ar-Stemim	0.5537	1.2596	-0.7059	St-Aphast	Ar-Timpop	0.6557	1.3772	-0.7215
An-Hydele	Ne-Caeele	0.3697	1.2598	-0.8902	St-Physyr	Ar-Glocon	0.6255	1.3778	-0.7523
An-Hydele	Ne-Caenig	0.3712	1.2598	-0.8886	St-Phycam	Ar-Timshe	0.5893	1.3788	-0.7895
St-Physyr	Ar-Opebru	0.4640	1.2599	-0.7959	Ne-Caeele	Ar-Aulsol	0.5878	1.3789	-0.7910
St-Phytub	Ar-Temlon	0.4922	1.2602	-0.7680	Ne-Caenig	Ar-Aulsol	0.5963	1.3789	-0.7825
St-Aphste	Ar-Helarm	0.4515	1.2603	-0.8088	St-Aphinv	Ar-Acypis	0.6987	1.3790	-0.6803
St-Aphste	Ar-Helzea	0.4591	1.2603	-0.8012	St-Physyr	Ar-Timmon	0.6621	1.3791	-0.7170
St-Phycam	Ar-Epacla	0.4591	1.2617	-0.8025	St-Phycam	Ar-Timpop	0.5909	1.3799	-0.7891
Ne-Caeele	Ar-Chisup	0.4402	1.2619	-0.8217	St-Physyr	Ar-Sipfla	0.7619	1.3801	-0.6181
Ne-Caenig	Ar-Chisup	0.4312	1.2619	-0.8307	St-Phytub	Ar-Aptruf	0.6515	1.3808	-0.7293
St-Aphast	Ar-Lymdis	0.4967	1.2625	-0.7658	St-Phycam	Ar-Timmon	0.6353	1.3811	-0.7458
St-Aphast	Mo-Limfor	0.6023	1.2637	-0.6613	St-Aphste	Ar-Censcu	0.6751	1.3819	-0.7068
Ne-Caeele	Ar-Vantam	0.4131	1.2637	-0.8507	St-Aphast	Ar-Timcri	0.6606	1.3826	-0.7220
Ne-Caenig	Ar-Vantam	0.4146	1.2637	-0.8492	St-Aphast	Ar-Timshe	0.6489	1.3827	-0.7338
St-Pytoli	Fn-Morsp.	0.3067	1.2650	-0.9582	St-Phycam	Ar-Aulsol	0.6957	1.3847	-0.6890
St-Phycam	Ar-Anogla	0.5631	1.2657	-0.7026	St-Physyr	Ar-Timcri	0.6226	1.3848	-0.7623
St-Aphinv	Ar-Osmbic	0.5272	1.2659	-0.7387	St-Physyr	Ar-Onttau	0.7558	1.3852	-0.6294
St-Phytub	Mo-Mermer	0.5289	1.2661	-0.7372	St-Phycam	Ar-Timcri	0.5949	1.3857	-0.7908
St-Pilapi	Ar-Aptruf	0.4589	1.2661	-0.8072	St-Physyr	Ar-Timpop	0.6175	1.3860	-0.7684
St-Pytoli	Ar-Glocon	0.4429	1.2663	-0.8234	St-Aphast	Ar-Timmon	0.6964	1.3878	-0.6914
St-Aphste	Ar-Erilan	0.4815	1.2667	-0.7852	Ne-Caeele	Ar-Schgra	0.6555	1.3912	-0.7357
Ne-Caeele	Ar-Epacla	0.4014	1.2677	-0.8663	Ne-Caenig	Ar-Schgra	0.6724	1.3912	-0.7188
Ne-Caenig	Ar-Epacla	0.3985	1.2677	-0.8692	St-Physyr	Ar-Timshe	0.6108	1.3913	-0.7806
St-Phycam	Ar-Aptruf	0.4863	1.2685	-0.7822	St-Phycam	Ar-Timbar	0.5833	1.3915	-0.8082
Ne-Caeele	Ar-Acypis	0.4401	1.2692	-0.8291	St-Phycam	Ar-Timtah	0.5837	1.3915	-0.8078
Ne-Caenig	Ar-Acypis	0.4472	1.2692	-0.8219	St-Physyr	Ar-Stemim	0.6664	1.3947	-0.7283
St-Phytub	Ar-Manjur	0.5109	1.2693	-0.7584	St-Phytub	Ar-Eupann	0.6117	1.3965	-0.7849
Fn-Morsp.	Ar-Stemim	0.5385	1.2697	-0.7312	St-Phytub	Ar-Timgen	0.5893	1.3972	-0.8079
St-Pytoli	Ar-Stedum	0.5751	1.2714	-0.6962	St-Physyr	Ar-Amytra	0.6255	1.3975	-0.7720
St-Aphinv	Ar-Glocon	0.4943	1.2720	-0.7777	St-Aphast	Ar-Manjur	0.6469	1.3979	-0.7509
St-Phycam	Ar-Opebru	0.4665	1.2721	-0.8056	St-Physyr	Ar-Stedum	0.7377	1.4004	-0.6626
Fn-Lobtra	Ar-Schgra	0.4070	1.2724	-0.8654	St-Phytub	Ar-Thrpal	0.5654	1.4004	-0.8350
Ne-Caeele	Ar-Sipfla	0.4297	1.2725	-0.8428	St-Phycam	Ar-Lephet	0.6749	1.4016	-0.7267
Ne-Caenig	Ar-Sipfla	0.4661	1.2725	-0.8064	St-Phytub	Ar-Glocon	0.6187	1.4019	-0.7833
St-Aphast	Ar-Stedum	0.6006	1.2725	-0.6719	St-Physyr	St-Phytub	0.1086	1.4022	-1.2936
St-Phycam	Ar-Chisup	0.5264	1.2727	-0.7463	St-Phytub	Ar-Acypis	0.7454	1.4026	-0.6572
St-Phycam	Ar-Onttau	0.5579	1.2731	-0.7153	St-Physyr	Ar-Thrpal	0.5787	1.4028	-0.8242
St-Aphinv	Ar-Partep	0.5404	1.2734	-0.7329	St-Aphast	Ar-Lepdec	0.6865	1.4040	-0.7175
St-Aphinv	Ar-Trivap	0.5775	1.2736	-0.6961	St-Phycam	St-Phytub	0.0881	1.4073	-1.3193
Fn-Lobtra	Ar-Ampamp	0.4054	1.2765	-0.8712	St-Aphast	Ar-Limcal	0.7386	1.4075	-0.6689
Fn-Morsp.	Ar-Osmbic	0.4999	1.2774	-0.7775	Fu-Lobtra	Ar-Adohon	0.5637	1.4080	-0.8443
St-Phycam	Ar-Helarm	0.4494	1.2777	-0.8282	St-Phytub	Ar-Osmbic	0.6819	1.4082	-0.7263
St-Physyr	Ar-Sitmis	0.5325	1.2777	-0.7452	St-Physyr	Ar-Limcal	0.7208	1.4087	-0.6879
St-Aphinv	Ar-Danmel	0.4637	1.2786	-0.8149	St-Phytub	Fu-Lobtra	0.5189	1.4088	-0.8899
St-Phycam	Ar-Helzea	0.4507	1.2798	-0.8290	St-Phycam	Ar-Schgra	0.7959	1.4096	-0.6137
Ne-Caeele	Ar-Glocon	0.3859	1.2800	-0.8941	St-Physyr	Ar-Galmel	0.6067	1.4097	-0.8030
Ne-Caenig	Ar-Glocon	0.3787	1.2800	-0.9013	St-Phycam	Ar-Lymdis	0.6486	1.4114	-0.7628
Fn-Morsp.	Ar-Vantam	0.4297	1.2802	-0.8505	St-Aphast	Ar-Aulsol	0.6625	1.4120	-0.7495
St-Phycam	Ar-Manjur	0.5128	1.2809	-0.7681	St-Aphast	Ar-Sipfla	0.7215	1.4130	-0.6916
St-Aphinv	Ar-Aptruf	0.5040	1.2812	-0.7771	St-Phytub	Ar-Aulsol	0.6468	1.4155	-0.7687
St-Aphast	Mo-Mermer	0.5501	1.2813	-0.7312	St-Phycam	Ar-Stedum	0.7783	1.4161	-0.6378
St-Aphast	Ar-Vantam	0.4902	1.2813	-0.7911	St-Physyr	Ar-Acypis	0.7531	1.4165	-0.6634
St-Aphast	Ar-Onttau	0.4945	1.2819	-0.7874	St-Aphinv	St-Pytoli	0.2893	1.4175	-1.1281
St-Aphinv	Ar-Helarm	0.4709	1.2821	-0.8111	St-Phytub	Ar-Timtah	0.5837	1.4185	-0.8348
St-Aphinv	Ar-Helzea	0.4716	1.2821	-0.8105	St-Phytub	Ar-Timbar	0.5855	1.4199	-0.8344
St-Phytub	Ar-Stemim	0.5658	1.2828	-0.7170	Ne-Caeele	Ar-Aptruf	0.6112	1.4201	-0.8090

Fn-Morsp.	Ar-Stedum	0.5472	1.2835	-0.7362	Ne-Caenig	Ar-Aptruf	0.6284	1.4201	-0.7917
Fn-Morsp.	An-Hydele	0.3633	1.2844	-0.9212	St-Physyr	Ar-Sogfur	0.6553	1.4215	-0.7662
St-Physyr	Ar-Epacla	0.4657	1.2847	-0.8190	Fu-Lobtra	Ar-Nillug	0.6216	1.4222	-0.8006
St-Aphinv	Ar-Lephet	0.4911	1.2852	-0.7941	St-Physyr	Ar-Censcu	0.7015	1.4224	-0.7209
Fn-Lobtra	Ar-Timgen	0.4482	1.2856	-0.8374	Fu-Lobtra	Ar-Sogfur	0.6135	1.4236	-0.8101
St-Physyr	Ar-Lymdis	0.5288	1.2867	-0.7579	St-Phytub	Ar-Schgra	0.7458	1.4241	-0.6783
St-Aphste	Ar-Epacla	0.4560	1.2869	-0.8309	Fu-Lobtra	Ar-Lephet	0.6685	1.4243	-0.7558
St-Aphste	Ar-Sipfla	0.4868	1.2886	-0.8017	St-Pytoli	Fu-Lobtra	0.4901	1.4263	-0.9362
St-Pilapi	Ar-Chisup	0.5045	1.2889	-0.7845	St-Physyr	St-Pytoli	0.2409	1.4266	-1.1857
St-Aphast	Ar-Stemim	0.5624	1.2892	-0.7268	St-Aphast	Ar-Censcu	0.6839	1.4278	-0.7438
Fn-Lobtra	Ar-Trivap	0.4977	1.2893	-0.7915	Fu-Morsp.	Ar-Thrpal	0.5439	1.4291	-0.8852
St-Phytub	Ar-Lymdis	0.5153	1.2893	-0.7740	Fu-Lobtra	Ar-Stedum	0.6773	1.4296	-0.7522
St-Aphste	Ar-Calmac	0.5219	1.2901	-0.7682	St-Physyr	Ar-Trivap	0.6848	1.4315	-0.7467
St-Aphinv	Ar-Timpop	0.5354	1.2906	-0.7552	Fu-Lobtra	Ar-Timgen	0.5829	1.4324	-0.8495
St-Aphast	Ar-Glocon	0.4720	1.2919	-0.8199	St-Phytub	Ar-Timshe	0.5802	1.4327	-0.8525
St-Physyr	Ar-Osmbic	0.5527	1.2921	-0.7395	St-Aphste	Ar-Tutabs	0.5733	1.4332	-0.8599
St-Aphast	Ar-Ampamp	0.4379	1.2924	-0.8545	Fu-Lobtra	Ar-Erilan	0.6435	1.4334	-0.7899
Fn-Lobtra	Ar-Stemim	0.4960	1.2926	-0.7965	St-Aphast	Ar-Acypis	0.7352	1.4353	-0.7000
St-Phytub	Ar-Eufmex	0.5909	1.2933	-0.7024	St-Phytub	Ar-Anogla	0.6478	1.4374	-0.7896
St-Phycam	Ar-Pseelo	0.5513	1.2936	-0.7423	St-Phycam	Ar-Tutabs	0.5421	1.4388	-0.8967
St-Aphinv	Ar-Timcri	0.5298	1.2939	-0.7641	Fu-Lobtra	Ar-Eupann	0.5942	1.4390	-0.8448
St-Phycam	Ar-Sipfla	0.5457	1.2946	-0.7489	St-Physyr	Ar-Partep	0.6794	1.4396	-0.7602
St-Phycam	Ar-Phesol	0.5804	1.2956	-0.7152	St-Phytub	Ar-Timpop	0.5819	1.4408	-0.8589
Fn-Morsp.	Ar-Aptruf	0.4265	1.2963	-0.8699	Fu-Lobtra	Ar-Limcal	0.6768	1.4412	-0.7644
Fn-Lobtra	Ar-Bomman	0.4313	1.2980	-0.8667	St-Phycam	Ar-Calmac	0.7089	1.4414	-0.7325
St-Phytub	Ar-Adohon	0.4201	1.2984	-0.8783	St-Phytub	Ar-Timmon	0.6311	1.4422	-0.8111
St-Aphast	Ar-Galmel	0.5158	1.2994	-0.7836	Fu-Lobtra	Ar-Glocon	0.6038	1.4432	-0.8393
St-Phytub	Mo-Batpla	0.5582	1.3003	-0.7421	St-Aphste	Ar-Eufmex	0.6787	1.4433	-0.7646
St-Aphste	Ar-Sitmis	0.4744	1.3008	-0.8263	St-Pilapi	Fu-Morsp.	0.4767	1.4434	-0.9667
St-Physyr	Mo-Mermer	0.5481	1.3009	-0.7528	St-Phycam	Ar-Ladful	0.7283	1.4435	-0.7151
St-Phycam	Ar-Limcal	0.4790	1.3016	-0.8226	St-Phycam	Ar-Amytra	0.5871	1.4449	-0.8578
St-Aphste	Ar-Galmel	0.4931	1.3017	-0.8086	Fu-Lobtra	Ar-Osmbic	0.6784	1.4454	-0.7670
St-Aphste	Ar-Phesol	0.5357	1.3018	-0.7661	Fu-Lobtra	Ar-Eupuro	0.5943	1.4486	-0.8544
St-Aphste	Ar-Censcu	0.5554	1.3023	-0.7469	Fu-Lobtra	Ar-Censcu	0.6510	1.4489	-0.7979
Fn-Lobtra	Ar-Sogfur	0.4077	1.3034	-0.8957	St-Phytub	Ar-Timcri	0.5905	1.4490	-0.8584
St-Physyr	Ar-Manjur	0.5020	1.3046	-0.8025	St-Aphast	Ar-Partep	0.6574	1.4492	-0.7918
St-Aphast	Ar-Bomman	0.5068	1.3051	-0.7983	St-Physyr	Ar-Homvit	0.6358	1.4501	-0.8143
St-Pytoli	Ar-Adohon	0.4278	1.3055	-0.8777	Fu-Lobtra	Ar-Timtah	0.5772	1.4502	-0.8730
St-Aphast	Ar-Amytra	0.4753	1.3055	-0.8302	St-Aphast	St-Pilapi	0.2804	1.4503	-1.1699
St-Physyr	Ar-Schgra	0.5263	1.3056	-0.7793	Fu-Morsp.	Ne-Caeele	0.6168	1.4511	-0.8343
Ne-Caeele	Ar-Schgra	0.4503	1.3064	-0.8561	Fu-Morsp.	Ne-Caenig	0.5956	1.4511	-0.8555
Ne-Caenig	Ar-Schgra	0.4526	1.3064	-0.8538	St-Aphinv	Ar-Eupann	0.6359	1.4517	-0.8158
St-Phytub	Ar-Osmbic	0.5355	1.3065	-0.7710	St-Aphast	Ar-Temlon	0.6779	1.4538	-0.7758
St-Aphast	Ar-Neopin	0.4789	1.3065	-0.8276	St-Phytub	Ar-Sipfla	0.7536	1.4538	-0.7002
Fn-Lobtra	Ar-Epacla	0.3939	1.3067	-0.9127	Fu-Lobtra	Ar-Timshe	0.5552	1.4546	-0.8993
Fn-Lobtra	Ar-Adohon	0.3646	1.3069	-0.9424	St-Phycam	Ar-Galmel	0.5678	1.4547	-0.8869
Fn-Lobtra	Ar-Danmel	0.4014	1.3073	-0.9059	Fu-Lobtra	Ar-Timbar	0.5777	1.4558	-0.8782
St-Aphste	St-Pilapi	0.2153	1.3084	-1.0931	Fu-Lobtra	Ar-Onttau	0.6878	1.4657	-0.7779
St-Physyr	Ar-Timgen	0.5322	1.3090	-0.7768	Fu-Lobtra	Ar-Timpop	0.5568	1.4659	-0.9090
Fn-Morsp.	Ar-Homvit	0.4110	1.3094	-0.8984	Fu-Lobtra	Ar-Timcri	0.5674	1.4671	-0.8998
St-Aphste	Ar-Anogla	0.5047	1.3107	-0.8060	Fu-Lobtra	Ar-Temlon	0.6283	1.4683	-0.8401
St-Aphste	Ar-Lepdec	0.4544	1.3107	-0.8563	Fu-Lobtra	Ar-Stemim	0.6450	1.4695	-0.8244
St-Aphast	An-Hydele	0.4183	1.3112	-0.8930	St-Aphste	Ar-Eupuro	0.6035	1.4700	-0.8665
St-Aphast	Ar-Helarm	0.4492	1.3113	-0.8622	Fu-Lobtra	Ar-Timmon	0.5681	1.4702	-0.9021
St-Physyr	Ar-Neopin	0.4874	1.3115	-0.8241	St-Aphinv	St-Pilapi	0.2961	1.4708	-1.1747
St-Phytub	Ar-Neopin	0.4904	1.3122	-0.8218	St-Phycam	Ar-Lepdec	0.6729	1.4723	-0.7994
St-Aphinv	Ar-Homvit	0.4898	1.3132	-0.8234	St-Physyr	St-Pilapi	0.2412	1.4725	-1.2313
St-Aphste	Ar-Partep	0.5256	1.3135	-0.7879	St-Physyr	Ne-Caeele	0.6679	1.4751	-0.8072
Ne-Caeele	Ar-Timpop	0.4328	1.3136	-0.8809	St-Physyr	Ne-Caenig	0.6573	1.4751	-0.8178

Ne-Caenig	Ar-Timpop	0.4444	1.3136	-0.8692	St-Phycam	Ar-Censcu	0.7111	1.4757	-0.7646
St-Aphinv	Ar-Vantam	0.4934	1.3137	-0.8203	St-Phycam	Ar-Sogfur	0.6533	1.4765	-0.8232
St-Aphast	Ar-Helzea	0.4582	1.3140	-0.8558	Fu-Morsp.	Ar-Stedum	0.6994	1.4783	-0.7789
St-Phytub	Ar-Bomman	0.5106	1.3141	-0.8035	St-Phytub	Ar-Onttau	0.7313	1.4816	-0.7504
St-Phycam	Ar-Adohon	0.4128	1.3150	-0.9022	St-Phycam	Ar-Partep	0.6953	1.4863	-0.7911
St-Aphinv	Ar-Sipfla	0.4845	1.3160	-0.8315	St-Aphast	Ar-Homvit	0.6650	1.4907	-0.8257
Ne-Caeele	Ar-Timcri	0.4192	1.3170	-0.8978	Fu-Lobtra	Ar-Calmac	0.6671	1.4934	-0.8263
Ne-Caenig	Ar-Timcri	0.4327	1.3170	-0.8843	St-Phycam	Ar-Eupann	0.6285	1.4934	-0.8649
St-Phytub	Ar-Stedum	0.5785	1.3174	-0.7389	St-Aphste	Ar-Eupann	0.6101	1.4990	-0.8889
St-Phycam	Ar-Acypis	0.5209	1.3177	-0.7968	St-Phycam	Ar-Erilan	0.7962	1.5011	-0.7049
St-Aphste	Ar-Acypis	0.4678	1.3178	-0.8500	St-Aphast	Ar-Sogfur	0.6661	1.5017	-0.8356
St-Aphinv	Ar-Sogfur	0.4632	1.3191	-0.8559	St-Aphste	Ar-Stedum	0.7420	1.5065	-0.7645
St-Phytub	Ar-Helarm	0.4428	1.3194	-0.8766	St-Aphinv	Ne-Caeele	0.7128	1.5081	-0.7953
St-Aphste	Mo-Mermer	0.5353	1.3198	-0.7845	St-Aphinv	Ne-Caenig	0.6982	1.5081	-0.8099
Ne-Caeele	Ar-Aptruf	0.4101	1.3200	-0.9099	Fu-Lobtra	Ar-Aptruf	0.6068	1.5142	-0.9073
Ne-Caenig	Ar-Aptruf	0.4209	1.3200	-0.8991	St-Pilapi	Fu-Lobtra	0.5163	1.5149	-0.9986
Fn-Morsp.	Ar-Danmel	0.3848	1.3206	-0.9358	Fu-Lobtra	Ne-Caeele	0.6243	1.5245	-0.9001
St-Aphast	Ar-Schgra	0.4722	1.3212	-0.8490	Fu-Lobtra	Ne-Caenig	0.6204	1.5245	-0.9040
St-Phytub	Ar-Lephet	0.4960	1.3219	-0.8259	St-Aphast	Ne-Caeele	0.6912	1.5260	-0.8347
Fn-Lobtra	Ar-Stedum	0.5102	1.3219	-0.8116	St-Aphast	Ne-Caenig	0.6908	1.5260	-0.8352
St-Physyr	Ar-Bomman	0.5281	1.3220	-0.7939	St-Phytub	Ar-Lepdec	0.6644	1.5261	-0.8617
St-Phycam	Ar-Osmbic	0.5468	1.3226	-0.7759	St-Aphast	Ar-Eupuro	0.6150	1.5284	-0.9135
St-Aphast	Ar-Calmac	0.5249	1.3231	-0.7983	St-Aphinv	Ar-Eupuro	0.6374	1.5364	-0.8990
Fn-Lobtra	Ar-Neopin	0.4022	1.3234	-0.9213	St-Phycam	Ar-Eupuro	0.6263	1.5385	-0.9122
St-Aphinv	An-Hydele	0.4513	1.3236	-0.8722	St-Physyr	Ar-Eupann	0.6053	1.5640	-0.9587
St-Physyr	Ar-Aptruf	0.4929	1.3237	-0.8307	St-Aphast	Ar-Eupann	0.6192	1.5723	-0.9531
St-Physyr	An-Hydele	0.4083	1.3242	-0.9160	St-Physyr	Ar-Eupuro	0.6129	1.6033	-0.9904
St-Aphinv	Ar-Thrpal	0.4735	1.3249	-0.8514	St-Phytub	Ne-Caeele	0.6705	1.6173	-0.9468
St-Phycam	Ar-Stemim	0.5729	1.3249	-0.7520	St-Phytub	Ne-Caenig	0.6663	1.6173	-0.9510
St-Aphinv	Ar-Manjur	0.4922	1.3250	-0.8328	St-Phycam	Ne-Caeele	0.6822	1.6274	-0.9452
Fn-Lobtra	Ar-Helarm	0.3941	1.3251	-0.9309	St-Phycam	Ne-Caenig	0.6805	1.6274	-0.9469
St-Aphinv	Ar-Limcal	0.4825	1.3264	-0.8439					
St-Physyr	Ar-Temlon	0.4791	1.3267	-0.8477					
St-Phycam	An-Hydele	0.3949	1.3283	-0.9334					
St-Aphste	Ar-Onttau	0.4904	1.3285	-0.8380					
St-Aphast	Ar-Limcal	0.4865	1.3288	-0.8423					
St-Physyr	Ar-Eufmex	0.6108	1.3292	-0.7184					
St-Physyr	Ar-Calmac	0.5515	1.3292	-0.7777					
St-Aphast	Ar-Ladful	0.5144	1.3294	-0.8151					
St-Aphinv	Ar-Neopin	0.4702	1.3298	-0.8596					
Fn-Lobtra	Ar-Timpop	0.4501	1.3299	-0.8798					
St-Aphinv	Ar-Pseelo	0.5226	1.3300	-0.8074					
St-Aphast	St-Pytoli	0.2171	1.3300	-1.1129					
St-Phycam	St-Pytoli	0.1574	1.3301	-1.1727					
St-Aphinv	Ar-Lepdec	0.4793	1.3304	-0.8511					
St-Phycam	Ar-Temlon	0.4743	1.3304	-0.8562					
St-Phytub	Ar-Helzea	0.4489	1.3309	-0.8820					
Fn-Lobtra	Ar-Timcri	0.4432	1.3309	-0.8878					
St-Aphinv	Ar-Acypis	0.4678	1.3322	-0.8644					
St-Phytub	Ar-Galmel	0.5203	1.3324	-0.8121					
St-Aphast	Ar-Erilan	0.5017	1.3325	-0.8308					
St-Phytub	Ar-Aptruf	0.4765	1.3327	-0.8562					
Fn-Lobtra	Ar-Ladful	0.4571	1.3344	-0.8773					
St-Aphast	Ar-Osmbic	0.5220	1.3345	-0.8125					
St-Phytub	Ar-Trivap	0.6039	1.3353	-0.7314					
St-Aphinv	Ar-Censcu	0.5531	1.3357	-0.7826					
Fn-Lobtra	Ar-Helzea	0.3942	1.3363	-0.9422					
St-Physyr	Ar-Stedum	0.5787	1.3365	-0.7579					
St-Pilapi	Ne-Caeele	0.4729	1.3377	-0.8648					

St-Pilapi	Ne-Caenig	0.4846	1.3377	-0.8531
St-Phytub	Ar-Erilan	0.5350	1.3383	-0.8032
St-Phytub	St-Pytoli	0.1609	1.3385	-1.1775
Fn-Lobtra	Ar-Tutabs	0.3779	1.3393	-0.9615
St-Phycam	Ar-Neopin	0.5259	1.3401	-0.8142
St-Aphast	Ar-Danmel	0.4619	1.3404	-0.8785
St-Phytub	Ar-Tutabs	0.4385	1.3406	-0.9020
St-Aphast	Ar-Aptruf	0.5018	1.3409	-0.8392
Fn-Lobtra	Ar-Partep	0.4869	1.3410	-0.8541
St-Physyr	Ar-Nillug	0.4262	1.3414	-0.9152
St-Aphast	Ar-Lephet	0.4869	1.3421	-0.8552
Fn-Lobtra	Ar-Erilan	0.4418	1.3427	-0.9009
St-Phytub	Ar-Nillug	0.4134	1.3444	-0.9310
St-Physyr	Ar-Pseelo	0.5442	1.3451	-0.8009
St-Aphinv	Ar-Schgra	0.4720	1.3453	-0.8732
St-Physyr	Ar-Lephet	0.5098	1.3465	-0.8367
St-Phycam	Ar-Vantam	0.5140	1.3467	-0.8327
St-Physyr	Ar-Adohon	0.4133	1.3482	-0.9349
St-Aphast	Ar-Nillug	0.4274	1.3485	-0.9211
Fn-Lobtra	Ar-Sipfla	0.4398	1.3487	-0.9089
St-Phycam	Ar-Eufmex	0.6110	1.3495	-0.7386
St-Aphast	Ar-Timgen	0.5196	1.3498	-0.8301
St-Phycam	Ar-Homvit	0.4744	1.3501	-0.8757
St-Phytub	Ar-Homvit	0.4791	1.3525	-0.8735
St-Aphinv	Ar-Nillug	0.4534	1.3537	-0.9004
Fn-Lobtra	Ar-Lepdec	0.4081	1.3544	-0.9463
St-Aphinv	Ar-Temlon	0.5033	1.3548	-0.8515
St-Physyr	Ar-Sipfla	0.5572	1.3555	-0.7983
St-Phytub	Ar-Ladful	0.5416	1.3558	-0.8142
St-Aphast	Ar-Thrpal	0.4719	1.3563	-0.8844
Fn-Lobtra	Ar-Limcal	0.4398	1.3567	-0.9170
St-Phycam	Ar-Trivap	0.6410	1.3588	-0.7178
St-Aphast	Ar-Sipfla	0.5056	1.3590	-0.8534
St-Phytub	Ar-Partep	0.5467	1.3600	-0.8132
Fn-Lobtra	Ar-Homvit	0.4100	1.3600	-0.9500
St-Aphast	St-Phytub	0.2426	1.3600	-1.1174
St-Aphast	Ar-Acypis	0.4766	1.3612	-0.8846
Fn-Lobtra	Ar-Acypis	0.4098	1.3613	-0.9515
St-Aphast	Ar-Manjur	0.4989	1.3619	-0.8630
St-Phytub	Ar-Acypis	0.4917	1.3620	-0.8703
Fn-Lobtra	Ar-Glocon	0.3657	1.3632	-0.9975
St-Physyr	Ar-Helzea	0.4557	1.3635	-0.9078
Fn-Lobtra	Ar-Thrpal	0.3942	1.3643	-0.9700
St-Phytub	Ar-Timgen	0.5240	1.3649	-0.8410
St-Physyr	Ar-Tutabs	0.4425	1.3651	-0.9226
St-Phycam	Ar-Glocon	0.4313	1.3663	-0.9350
St-Physyr	Ar-Limcal	0.4939	1.3669	-0.8729
Fn-Morsp.	Ar-Thrpal	0.3729	1.3674	-0.9945
Fn-Lobtra	Ar-Manjur	0.4141	1.3684	-0.9543
St-Physyr	Ar-Helarm	0.4546	1.3691	-0.9145
St-Phycam	Ar-Schgra	0.5211	1.3704	-0.8493
St-Aphste	Ar-Tutabs	0.4410	1.3724	-0.9314
St-Aphinv	St-Phytub	0.2546	1.3732	-1.1185
St-Physyr	Ar-Amytra	0.4963	1.3743	-0.8780
Ne-Caeele	Ar-Thrpal	0.3948	1.3749	-0.9801
Ne-Caenig	Ar-Thrpal	0.3839	1.3749	-0.9911
St-Physyr	Ar-Chisup	0.5351	1.3755	-0.8404
St-Aphinv	Ar-Eupann	0.4508	1.3756	-0.9248
St-Physyr	Ar-Lepdec	0.5211	1.3776	-0.8564

St-Physyr	Ar-Acypis	0.5267	1.3778	-0.8511
St-Phycam	Ar-Timgen	0.5233	1.3788	-0.8555
St-Phytub	Ar-Chisup	0.5224	1.3799	-0.8574
St-Phytub	Ar-Eupuro	0.4654	1.3815	-0.9161
St-Physyr	Ar-Onttau	0.5548	1.3824	-0.8276
St-Phycam	Ar-Bomman	0.5163	1.3825	-0.8662
Fn-Lobtra	Ar-Nillug	0.3724	1.3834	-1.0110
St-Phytub	Ar-Calmac	0.5284	1.3835	-0.8551
St-Aphste	Ar-Stedum	0.5823	1.3854	-0.8031
St-Phytub	Ar-Limcal	0.4762	1.3857	-0.9095
St-Phytub	Ar-Anogla	0.5598	1.3864	-0.8267
St-Aphste	Ne-Caeele	0.4369	1.3888	-0.9519
St-Aphste	Ne-Caenig	0.4585	1.3888	-0.9303
St-Phycam	St-Pilapi	0.1845	1.3898	-1.2053
St-Phycam	Ar-Lephet	0.5204	1.3906	-0.8702
St-Phytub	St-Pilapi	0.1569	1.3923	-1.2354
St-Phytub	Ar-Sogfur	0.4337	1.3924	-0.9587
St-Phycam	Ar-Timpop	0.5204	1.3936	-0.8733
St-Physyr	Ar-Stemim	0.5712	1.3951	-0.8239
Fn-Lobtra	Ar-Lephet	0.4506	1.3956	-0.9450
St-Pilapi	Fn-Morsp.	0.3731	1.3964	-1.0233
St-Physyr	Ar-Censcu	0.6133	1.3974	-0.7841
St-Phycam	Ar-Timcri	0.5220	1.3995	-0.8775
St-Phytub	An-Hydele	0.4097	1.4012	-0.9914
St-Physyr	Ar-Timcri	0.5311	1.4017	-0.8707
St-Phycam	Ar-Stedum	0.5920	1.4026	-0.8106
St-Physyr	Ar-Timpop	0.5320	1.4029	-0.8709
St-Aphast	Ar-Lepdec	0.4742	1.4032	-0.9290
St-Physyr	St-Pytoli	0.1620	1.4067	-1.2446
Fn-Lobtra	Ar-Calmac	0.4530	1.4073	-0.9543
St-Aphast	Ar-Partep	0.5318	1.4078	-0.8760
St-Aphast	Ar-Timpop	0.5287	1.4081	-0.8794
St-Phytub	Ar-Ampamp	0.4053	1.4116	-1.0063
Fn-Lobtra	Ar-Osmbic	0.4755	1.4131	-0.9376
St-Physyr	Ar-Danmel	0.4651	1.4131	-0.9480
St-Aphast	Ar-Timcri	0.5192	1.4138	-0.8946
Fn-Morsp.	Ne-Caeele	0.3991	1.4158	-1.0167
Fn-Morsp.	Ne-Caenig	0.3968	1.4158	-1.0190
St-Phytub	Ar-Timpop	0.5119	1.4162	-0.9044
St-Aphste	Ar-Eufmex	0.5492	1.4176	-0.8684
St-Phycam	St-Phytub	0.0578	1.4178	-1.3600
St-Physyr	Ar-Galmel	0.5366	1.4181	-0.8815
Fn-Lobtra	Ar-Eupuro	0.4182	1.4183	-1.0001
St-Phytub	Ar-Timcri	0.5120	1.4254	-0.9134
St-Physyr	Ar-Thrpal	0.4422	1.4261	-0.9838
Fn-Lobtra	Ar-Censcu	0.5080	1.4271	-0.9191
Fn-Lobtra	Ar-Temlon	0.4277	1.4274	-0.9997
St-Phytub	Ar-Sipfla	0.5280	1.4275	-0.8995
St-Physyr	Ne-Caeele	0.4606	1.4285	-0.9678
St-Physyr	Ne-Caenig	0.4458	1.4285	-0.9826
Fn-Lobtra	Ar-Eupann	0.4227	1.4285	-1.0059
Fn-Lobtra	Ar-Onttau	0.4697	1.4285	-0.9588
St-Phytub	Ar-Glocon	0.4362	1.4286	-0.9925
St-Phycam	Ar-Calmac	0.5431	1.4308	-0.8877
St-Phytub	Ar-Onttau	0.5548	1.4349	-0.8801
St-Phytub	Ar-Eupann	0.4754	1.4351	-0.9597
St-Phycam	Ar-Galmel	0.5307	1.4357	-0.9050
St-Physyr	Ar-Ladful	0.5401	1.4361	-0.8960
Fn-Lobtra	Ar-Aptruf	0.4090	1.4375	-1.0286

St-Phycam	Ar-Censcu	0.6068	1.4408	-0.8341
St-Phytub	Ar-Schgra	0.4971	1.4409	-0.9438
St-Physyr	St-Phytub	0.0769	1.4414	-1.3645
St-Aphinv	St-Pytoli	0.2221	1.4445	-1.2224
St-Physyr	St-Pilapi	0.1679	1.4451	-1.2772
St-Phycam	Ar-Lepdec	0.5031	1.4451	-0.9421
Fn-Lobtra	Ar-Pseelo	0.4636	1.4483	-0.9847
St-Phycam	Ar-Amytra	0.4982	1.4484	-0.9502
St-Physyr	Ar-Sogfur	0.4349	1.4488	-1.0140
St-Phycam	Ar-Tutabs	0.4320	1.4503	-1.0183
St-Aphast	Ar-Sogfur	0.4538	1.4509	-0.9970
St-Pytoli	Fn-Lobtra	0.3240	1.4516	-1.1275
St-Phytub	Fn-Lobtra	0.3530	1.4516	-1.0987
St-Phycam	Ar-Sogfur	0.4196	1.4534	-1.0339
St-Phytub	Ar-Lepdec	0.4802	1.4545	-0.9743
St-Aphast	Ar-Censcu	0.5710	1.4556	-0.8846
Fn-Lobtra	An-Hydele	0.3716	1.4572	-1.0856
St-Phytub	Ar-Vantam	0.5085	1.4574	-0.9489
St-Aphinv	Ne-Caeele	0.4757	1.4604	-0.9847
St-Aphinv	Ne-Caenig	0.4815	1.4604	-0.9789
St-Phycam	Ar-Ladful	0.5463	1.4629	-0.9166
St-Phycam	Ar-Eupann	0.4823	1.4634	-0.9811
St-Phycam	Ar-Danmel	0.4587	1.4653	-1.0066
Fn-Lobtra	Ar-Vantam	0.4270	1.4665	-1.0395
St-Aphast	Ar-Temlon	0.4985	1.4669	-0.9685
St-Phycam	Ar-Erilan	0.5570	1.4679	-0.9109
St-Physyr	Ar-Glocon	0.4415	1.4693	-1.0278
St-Phycam	Ar-Partep	0.5649	1.4699	-0.9050
St-Aphinv	Ar-Eupuro	0.4511	1.4712	-1.0201
St-Pilapi	Fn-Lobtra	0.3512	1.4792	-1.1279
St-Physyr	Ar-Vantam	0.5134	1.4799	-0.9665
St-Physyr	Ar-Trivap	0.6100	1.4845	-0.8745
St-Aphast	St-Pilapi	0.2231	1.4861	-1.2631
St-Aphast	Ne-Caeele	0.4588	1.4890	-1.0302
St-Aphast	Ne-Caenig	0.4678	1.4890	-1.0212
St-Physyr	Ar-Partep	0.5653	1.4921	-0.9268
St-Aphinv	St-Pilapi	0.2310	1.4934	-1.2624
St-Phycam	Ar-Lymdis	0.5089	1.4937	-0.9848
St-Aphast	Ar-Pseelo	0.5203	1.4950	-0.9748
St-Aphste	Ar-Eupuro	0.4526	1.4996	-1.0471
Fn-Lobtra	Ne-Caeele	0.4152	1.5048	-1.0896
Fn-Lobtra	Ne-Caenig	0.4492	1.5048	-1.0556
St-Phycam	Ar-Eupuro	0.4767	1.5098	-1.0331
St-Physyr	Ar-Eupann	0.4874	1.5220	-1.0346
St-Aphast	Ar-Eupuro	0.4665	1.5234	-1.0569
St-Aphste	Ar-Eupann	0.4433	1.5280	-1.0847
St-Phytub	Ar-Thrpal	0.4435	1.5286	-1.0851
St-Aphast	Ar-Homvit	0.5112	1.5306	-1.0194
St-Aphast	Ar-Eupann	0.4590	1.5437	-1.0847
St-Physyr	Ar-Homvit	0.4965	1.5537	-1.0572
St-Physyr	Ar-Eupuro	0.4863	1.5590	-1.0727
St-Phytub	Ne-Caeele	0.4655	1.6081	-1.1426
St-Phytub	Ne-Caenig	0.4770	1.6081	-1.1311
St-Phycam	Ne-Caeele	0.4590	1.6357	-1.1767
St-Phycam	Ne-Caenig	0.4600	1.6357	-1.1757
