# Supplementary figures and images for: Prokaryotic and Eukaryotic Horizontal Transfer of Sailor (DD82E), a New Superfamily of IS630-Tc1-Mariner DNA Transposons
Source: Biology (Basel). 2021 Oct 7;10(10):1005. doi: 10.3390/biology10101005 (PMC8533490; doi:10.3390/biology10101005)

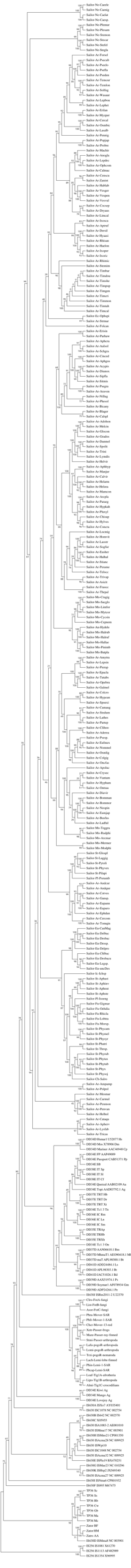

Supplement: Supplementary file 1 [file biology-10-01005-s001.zip › Supplementary Material/Supplementary Figure S3.PDF]
